# Supplementary material for: Oncogenic PKA signaling increases c-MYC protein expression through multiple targetable mechanisms
Source: eLife. 2023 Jan 24;12:e69521. doi: 10.7554/eLife.69521 (PMC9925115; doi:10.7554/eLife.69521)
Supplement: Figure 1—source data 3. [file elife-69521-fig1-data3.zip › 1D left panel/1D mark up.pdf]

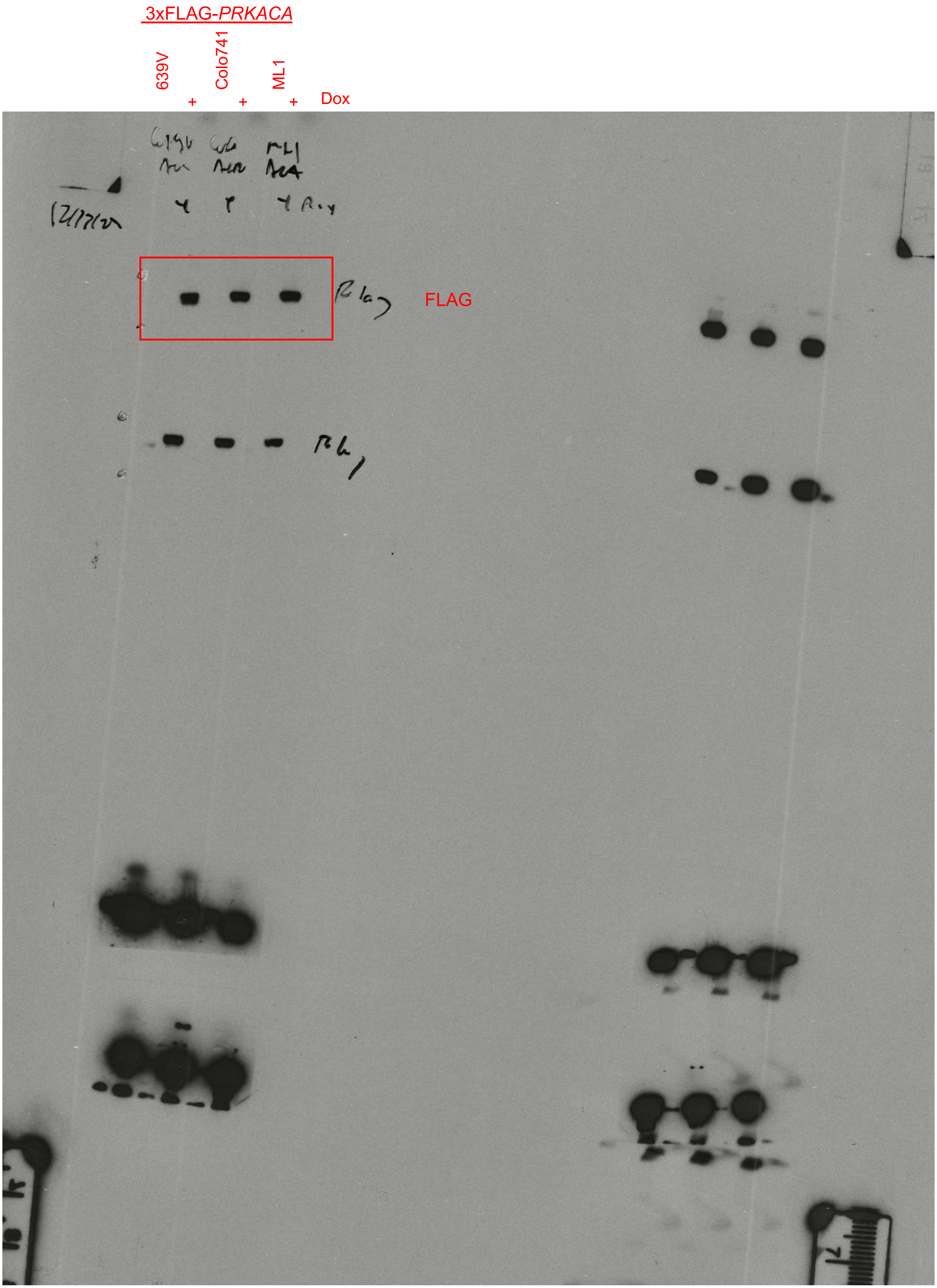
3xFLAG-PRKACA
639V
Colo741
ML1
+
+
+
Dox
FLAG

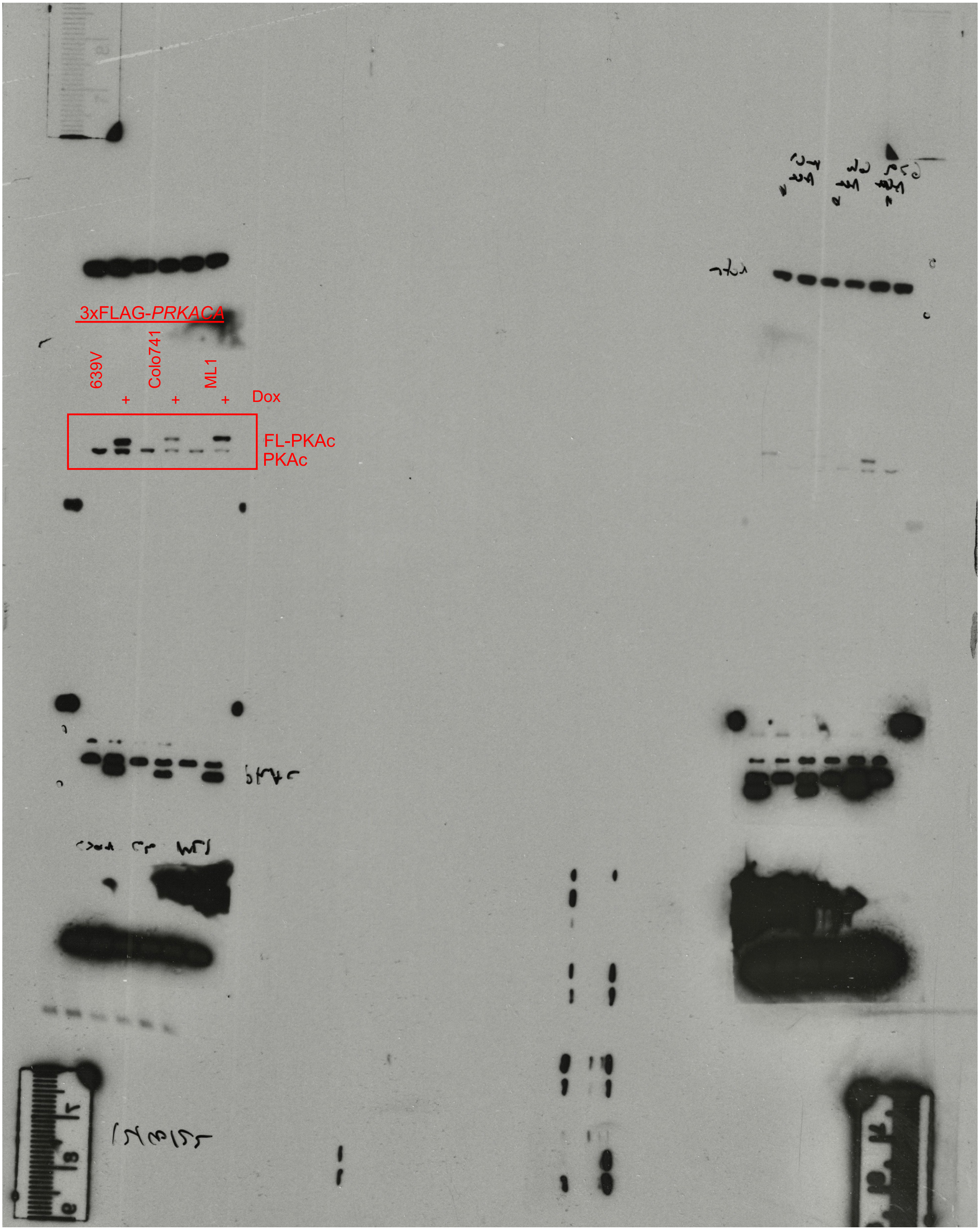

3xFLAG-PRKACA
639V
Colo741
ML1
+
+
+
Dox
FL-PKAc
PKAc

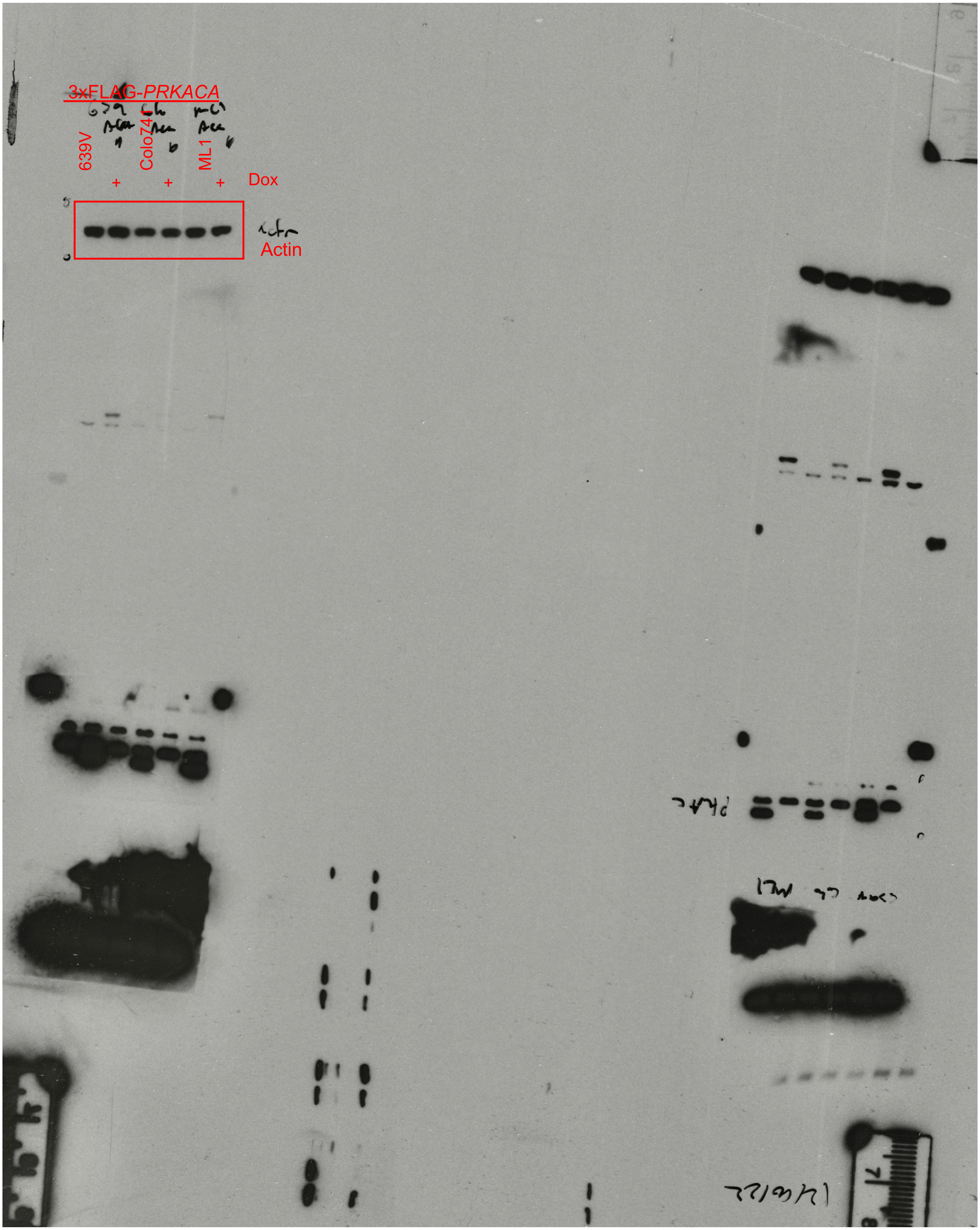
3xFLAG-PRKACA
639V
Colo741
ML1
+
+
+
Dox
Actin

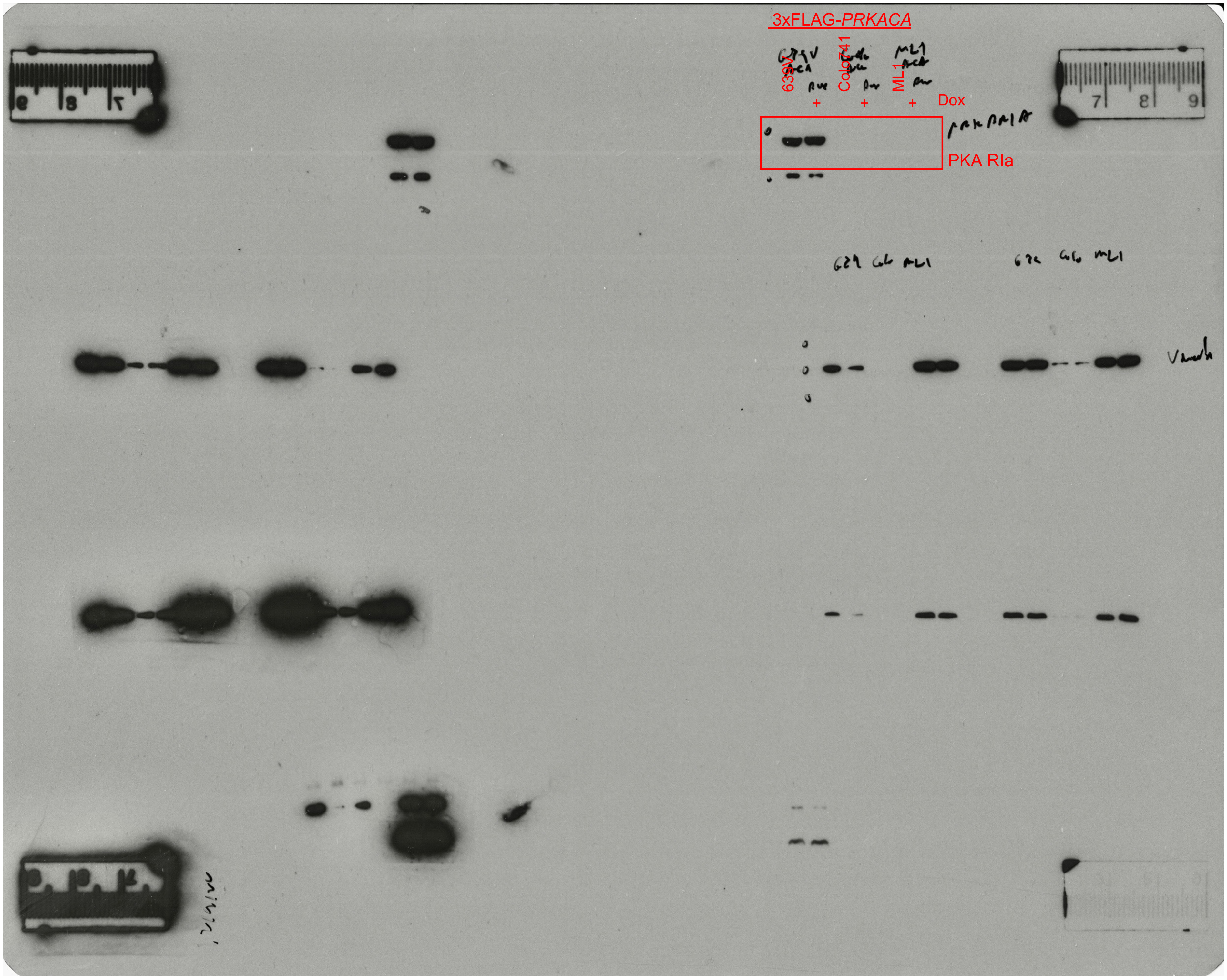
3xFLAG-PRKACA
639V
Colo741
ML1
+
+
+
Dox
PKA RIa
