# Supplementary figures and images for: Oncogenic PKA signaling increases c-MYC protein expression through multiple targetable mechanisms (part 1 of 2)
Source: eLife. 2023 Jan 24;12:e69521. doi: 10.7554/eLife.69521 (PMC9925115; doi:10.7554/eLife.69521)

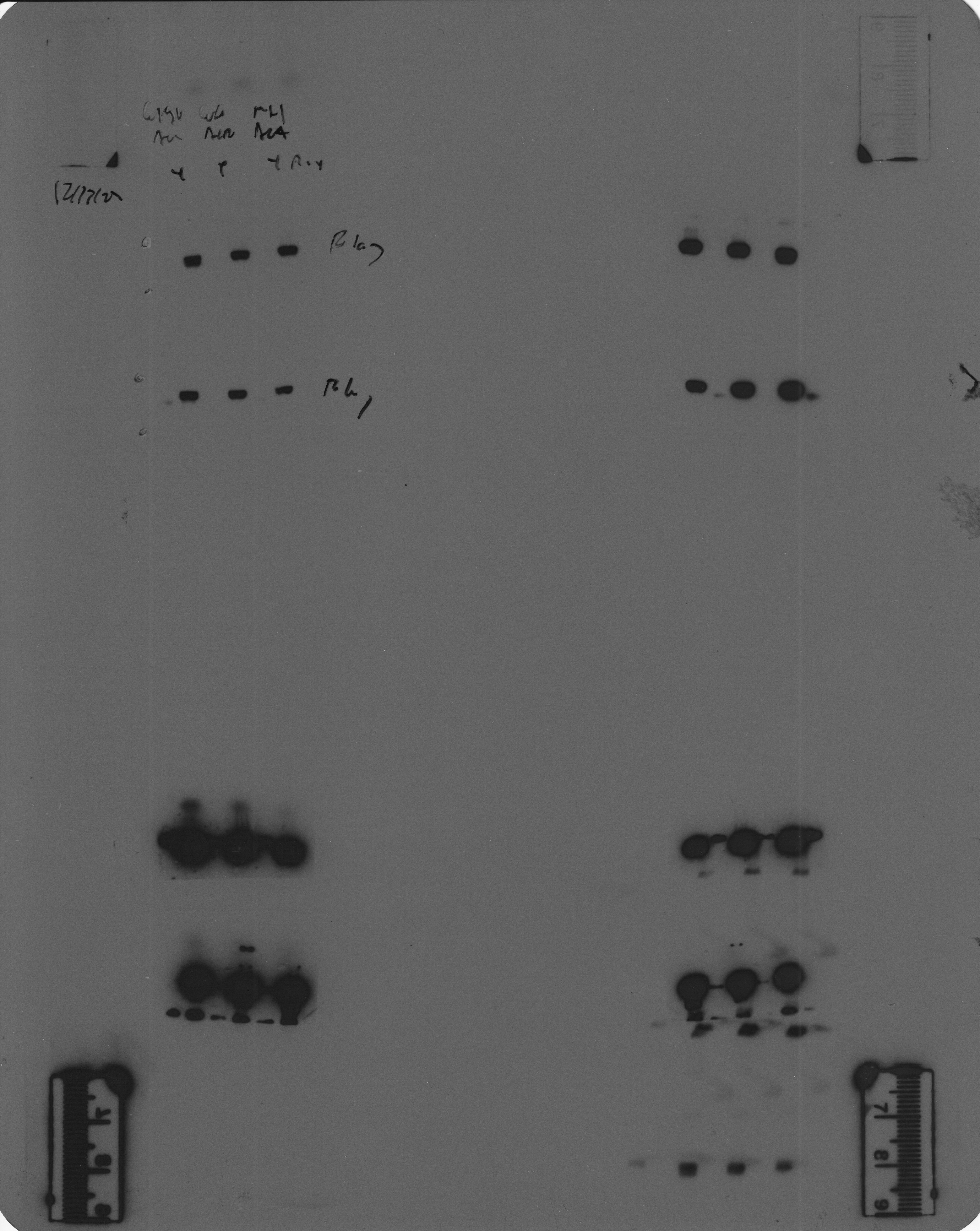

Supplement: Figure 1—source data 3. [file elife-69521-fig1-data3.zip › 1D left panel/1D flag.tif]

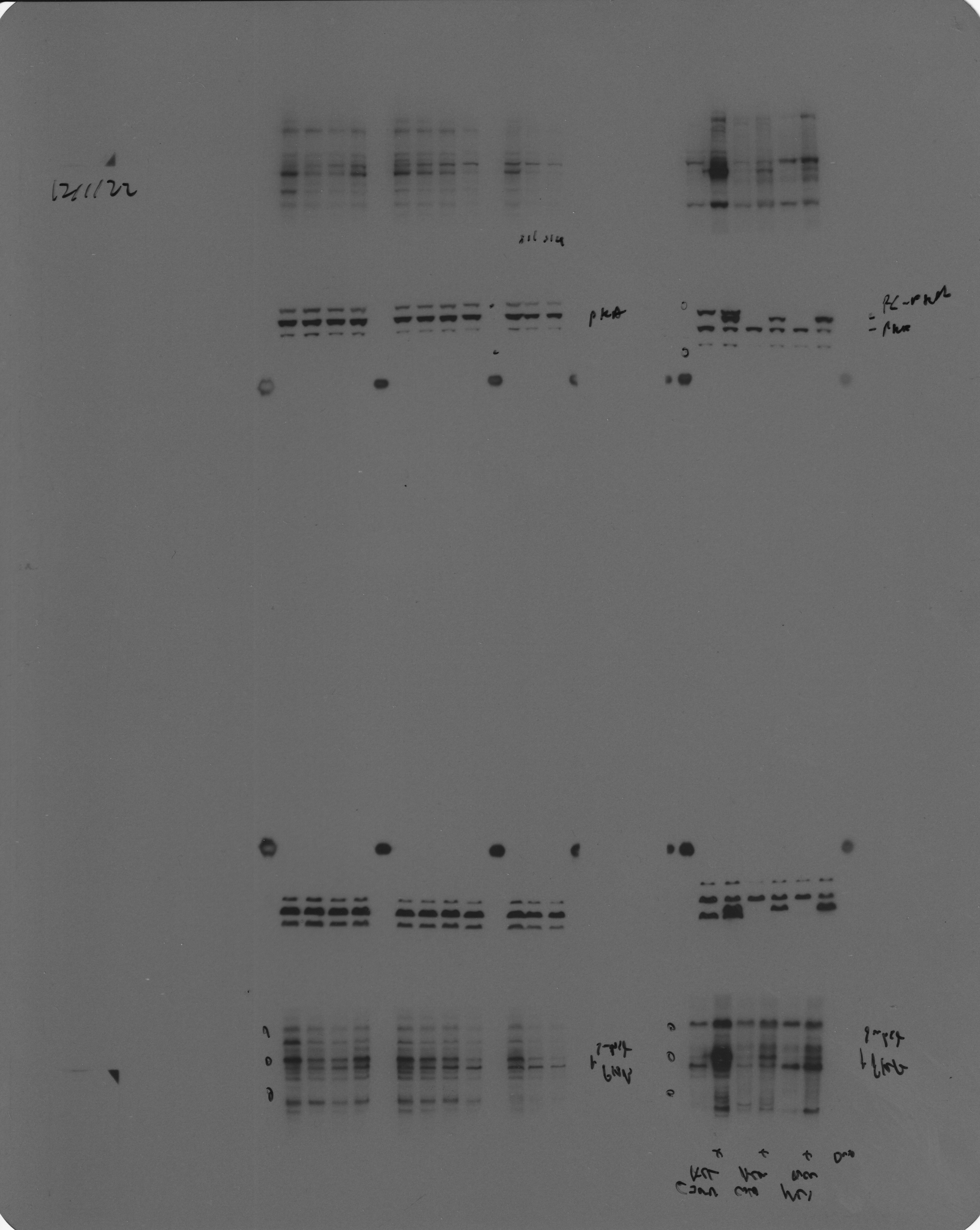

Supplement: Figure 1—source data 3. [file elife-69521-fig1-data3.zip › 1D left panel/1D p-pka subst raw.tif]

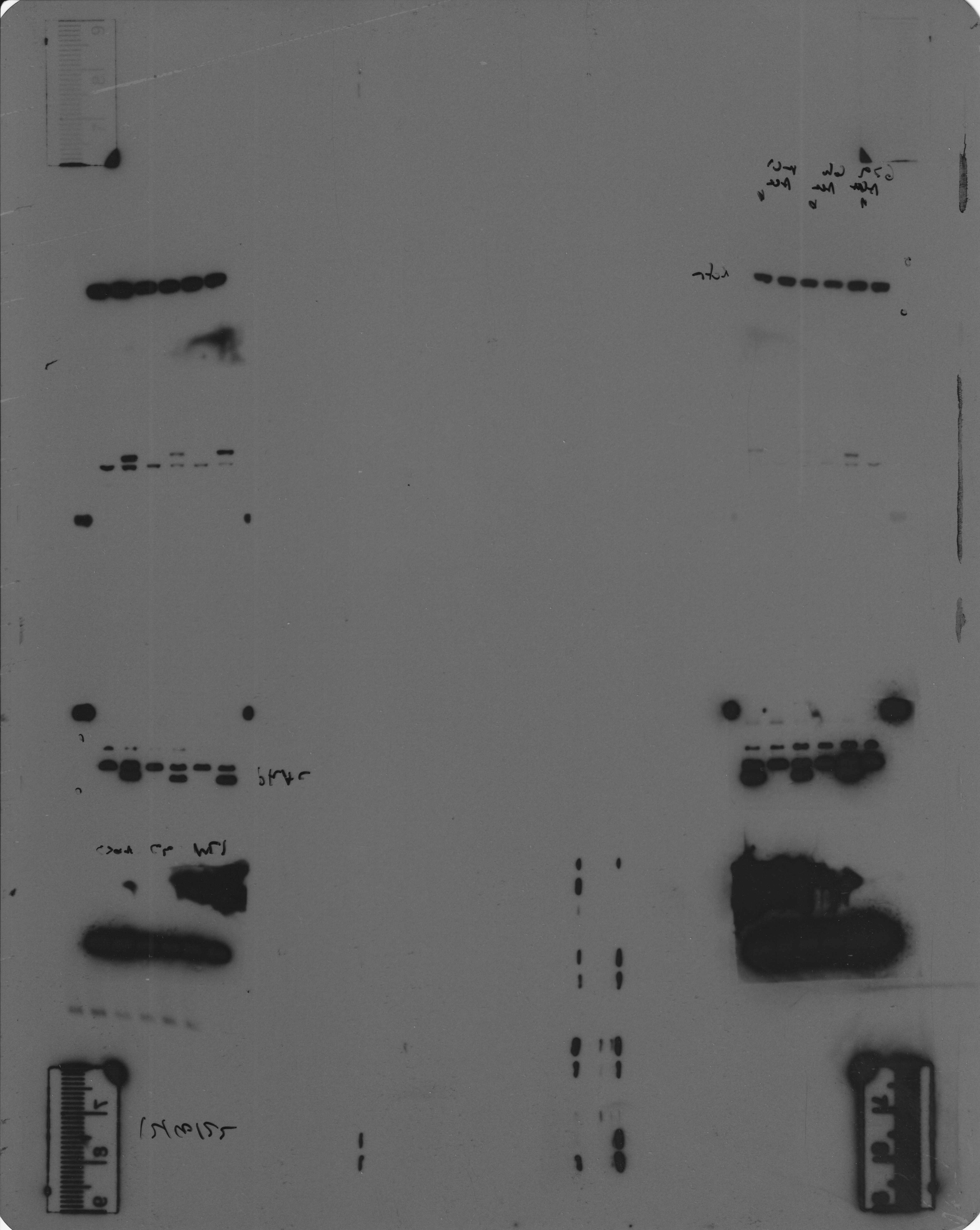

Supplement: Figure 1—source data 3. [file elife-69521-fig1-data3.zip › 1D left panel/1D PKAc.tif]

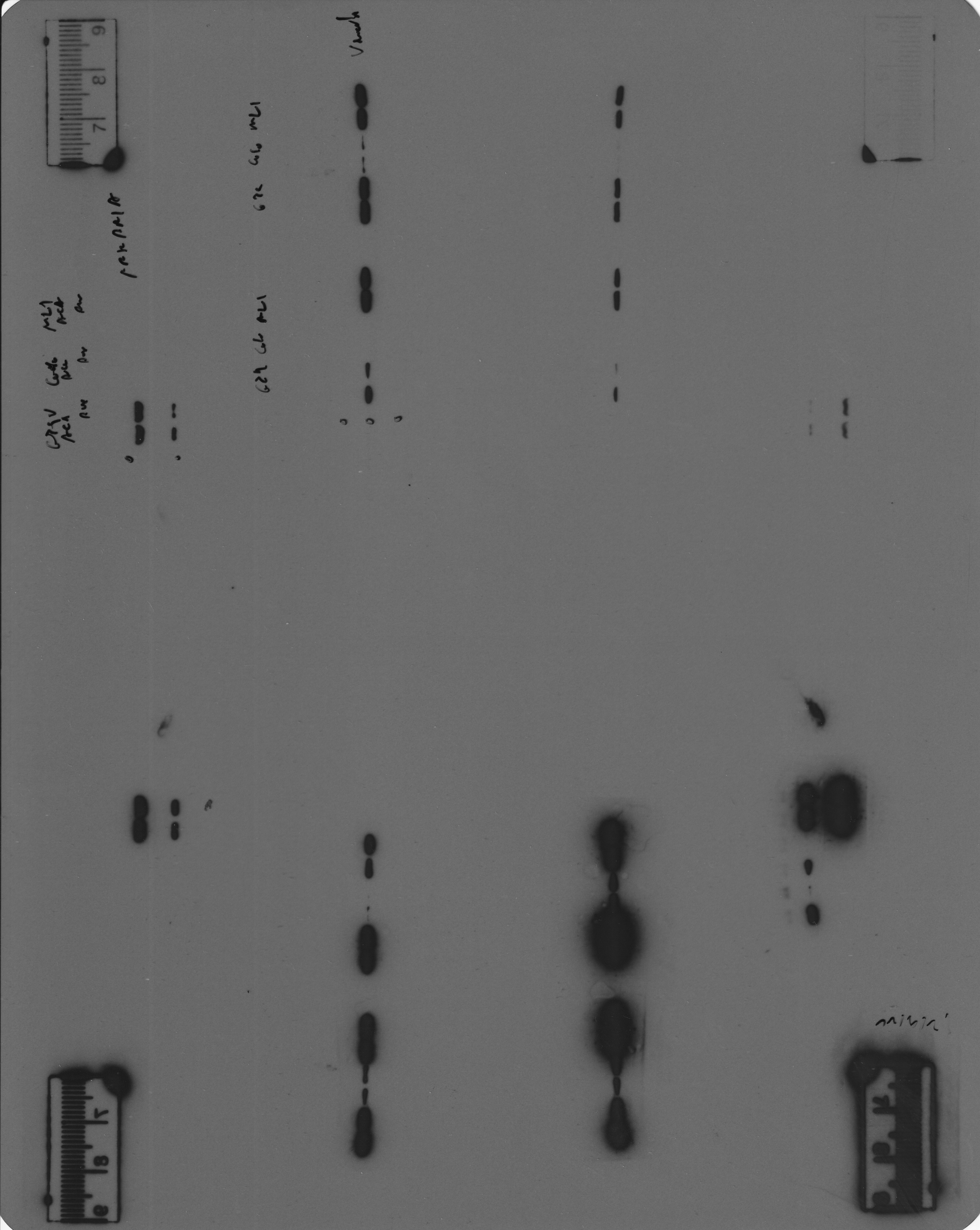

Supplement: Figure 1—source data 3. [file elife-69521-fig1-data3.zip › 1D left panel/1D vinculin AR1.tif]

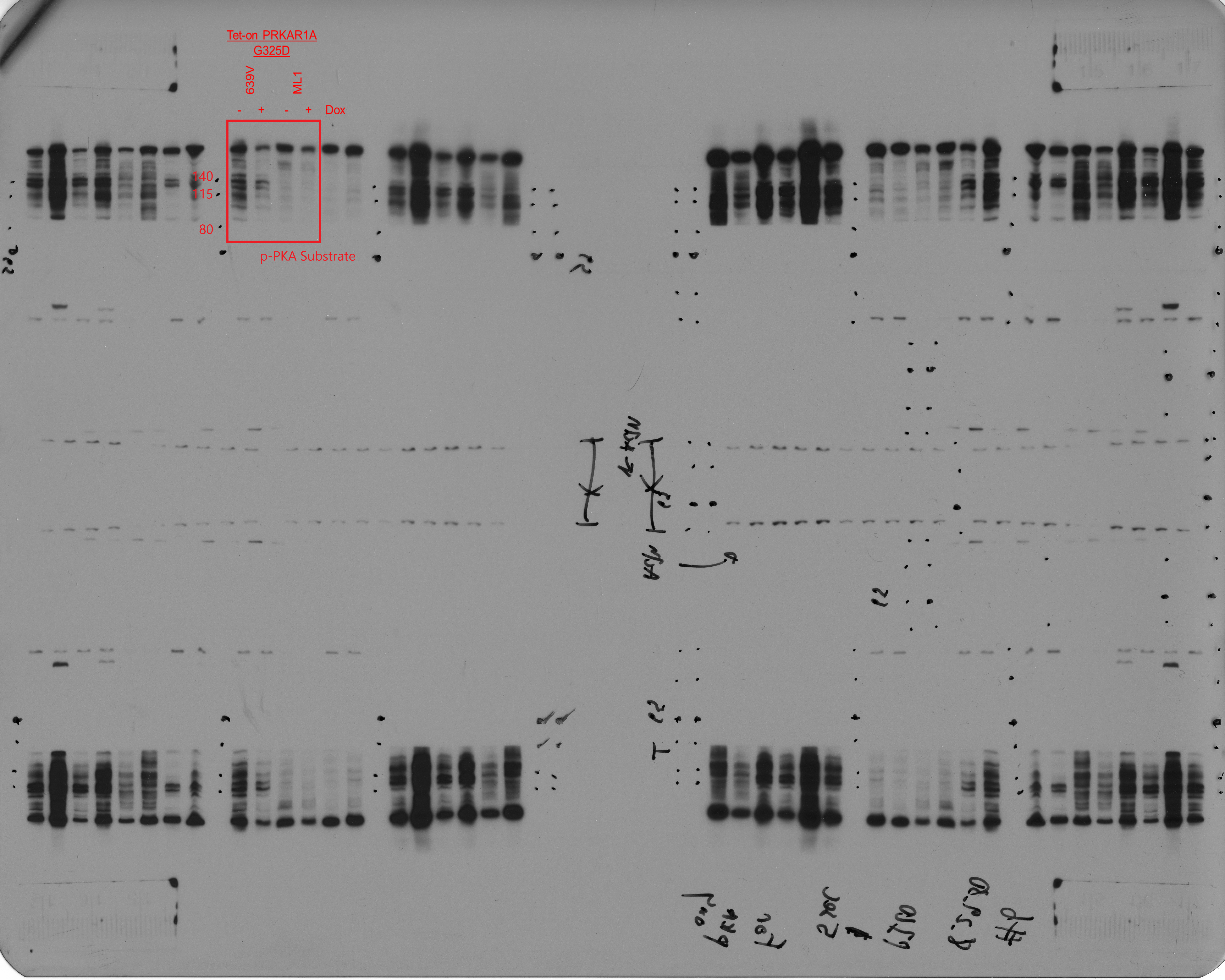

Supplement: Figure 1—source data 4. [file elife-69521-fig1-data4.zip › Figure 1D pPKA Subtrate labelled.tif]

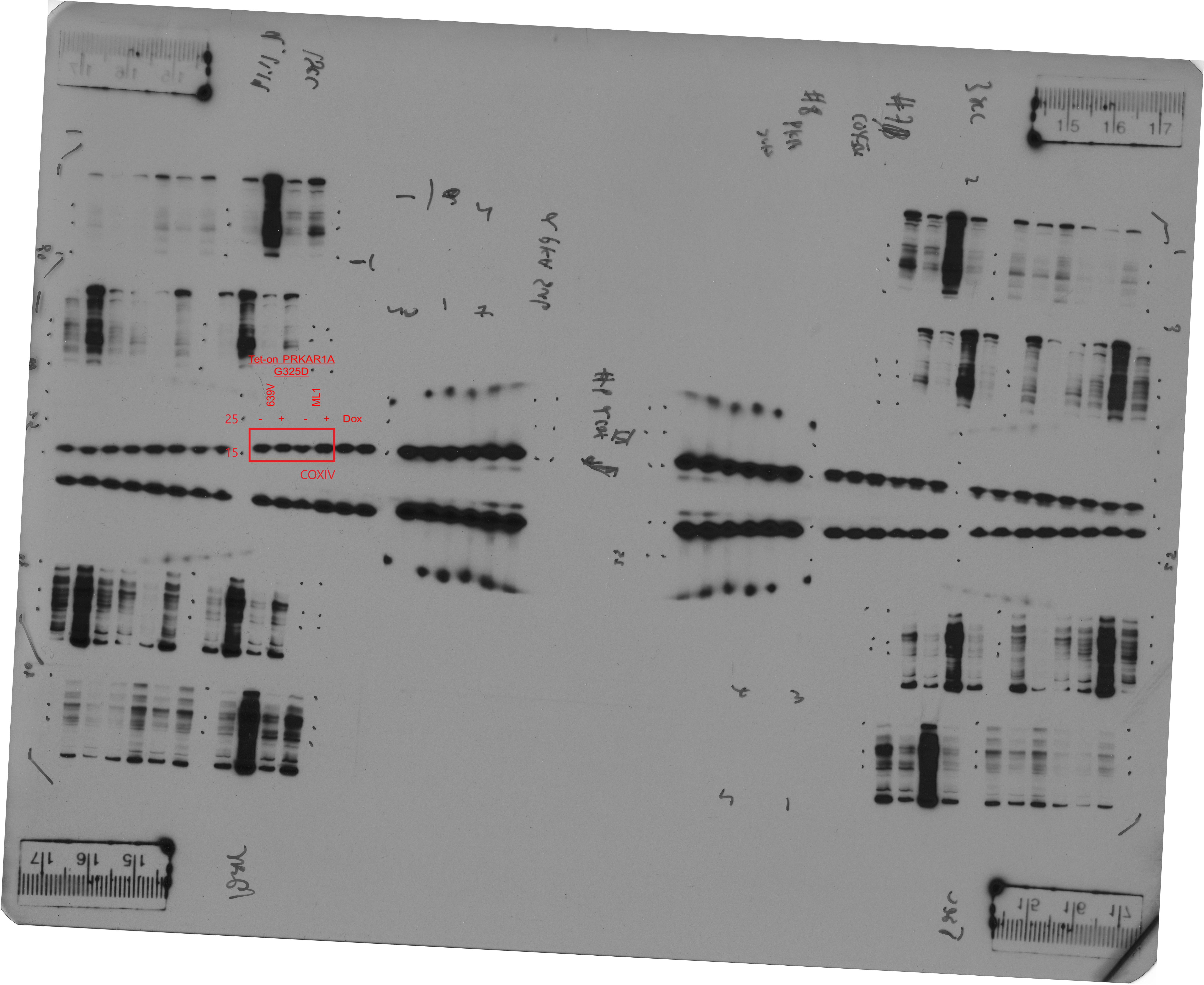

Supplement: Figure 1—source data 4. [file elife-69521-fig1-data4.zip › Figure 1D COXIV labelled.tif]

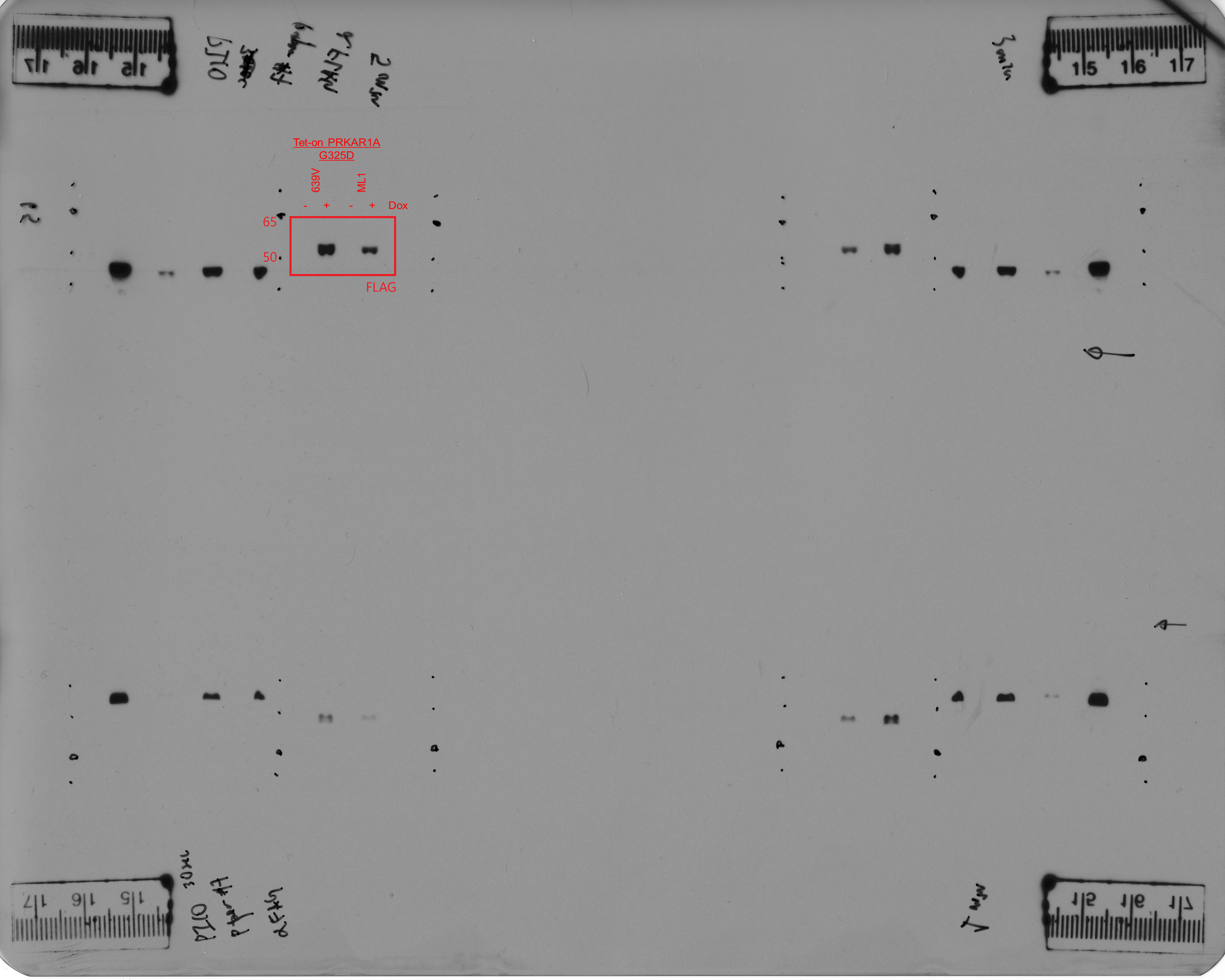

Supplement: Figure 1—source data 4. [file elife-69521-fig1-data4.zip › Figure 1D FLAG Labelled.tif]

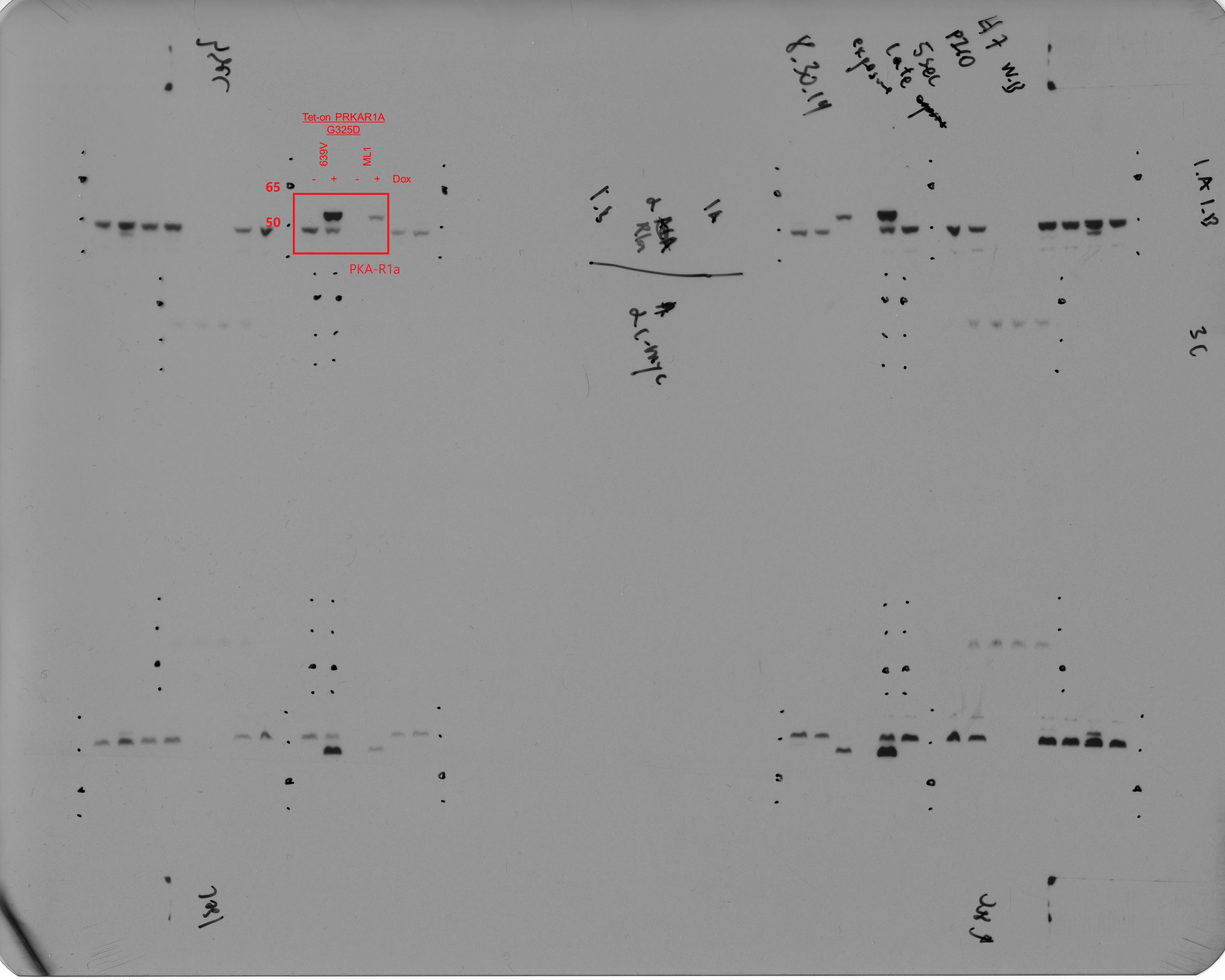

Supplement: Figure 1—source data 4. [file elife-69521-fig1-data4.zip › Figure 1D PKAR1a Labelled.tif]

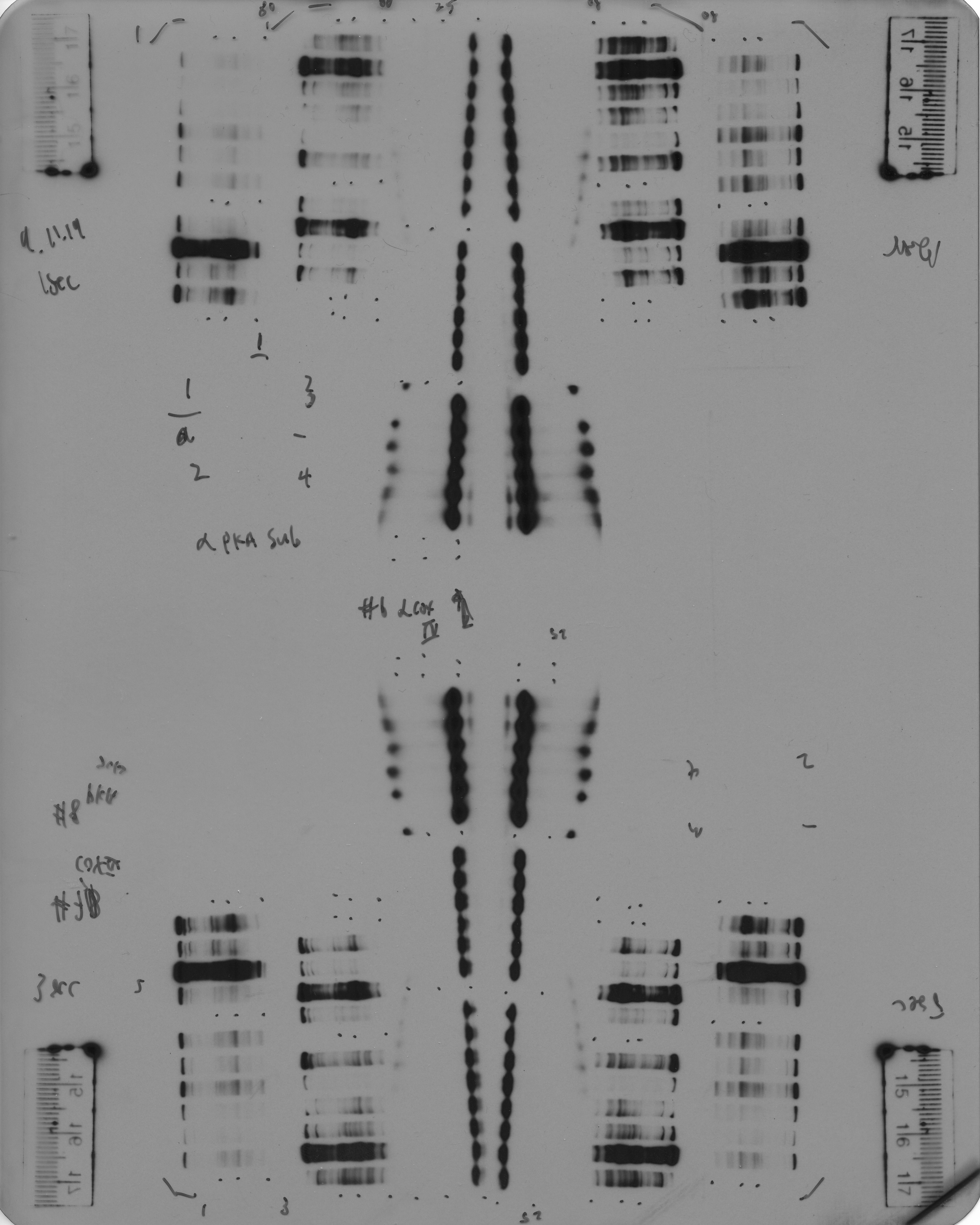

Supplement: Figure 1—source data 5. [file elife-69521-fig1-data5.zip › Figure 1D COXIV Raw.tif]

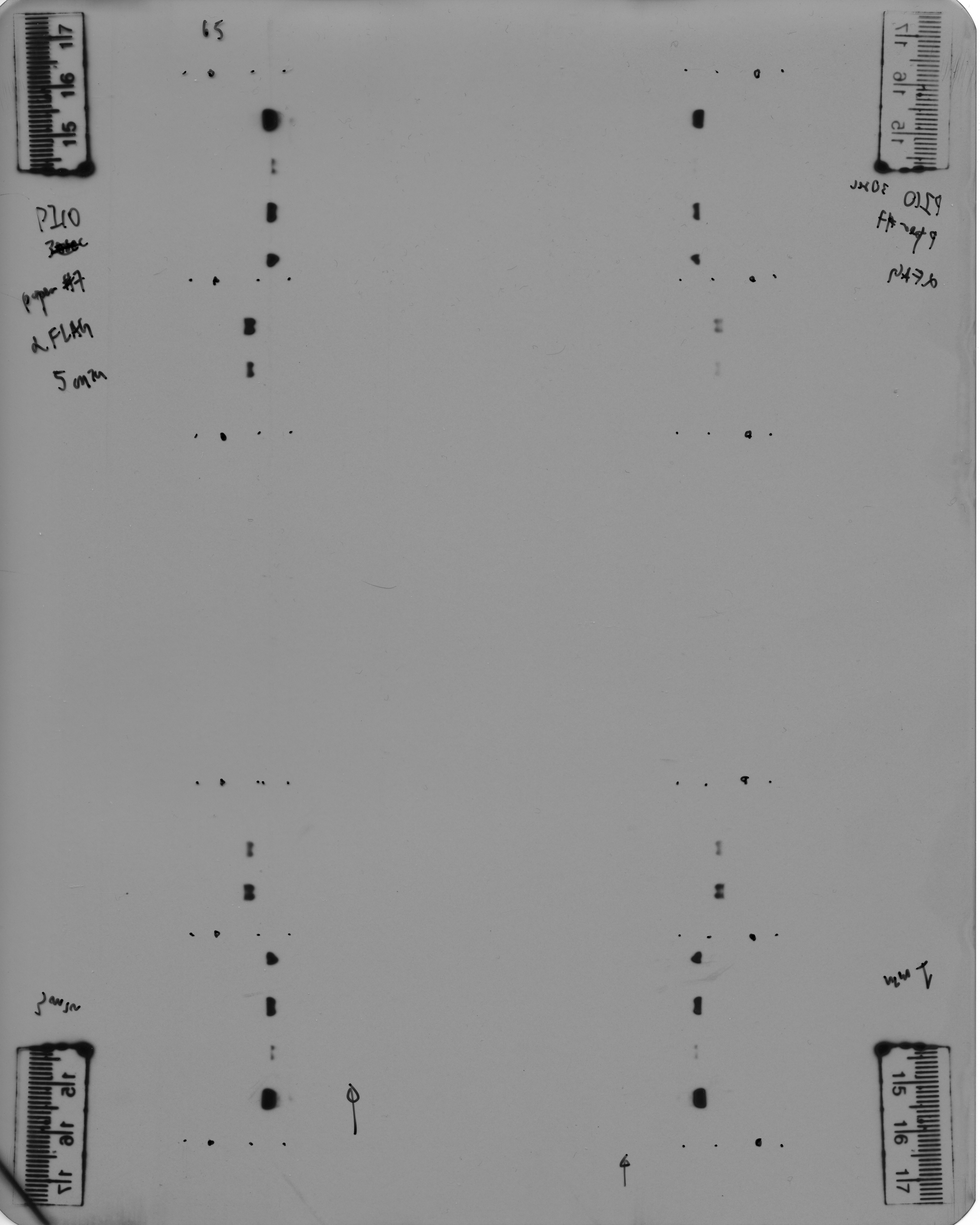

Supplement: Figure 1—source data 5. [file elife-69521-fig1-data5.zip › Figure 1D FLAG Raw.tif]

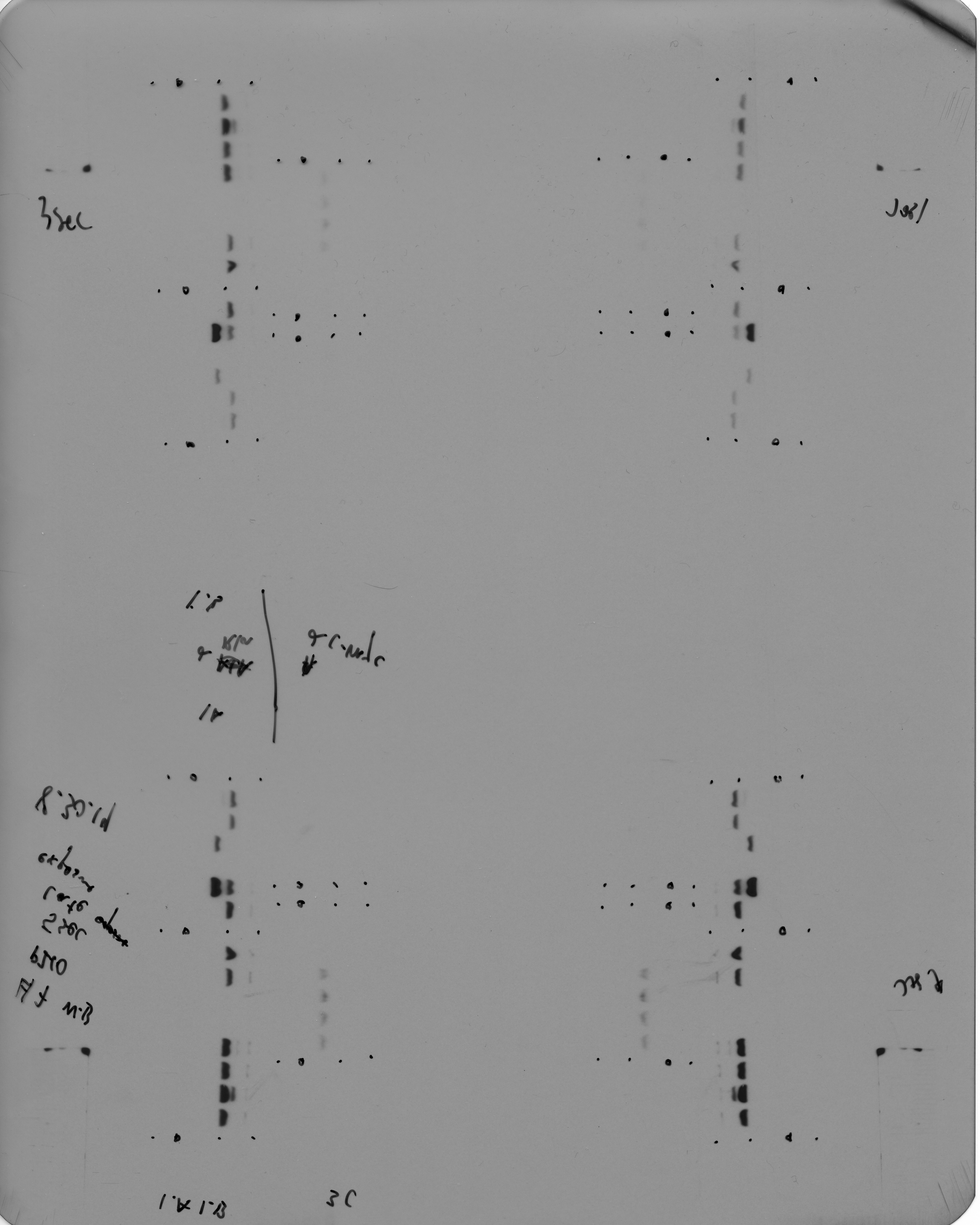

Supplement: Figure 1—source data 5. [file elife-69521-fig1-data5.zip › Figure 1D PKAR1a Raw.tif]

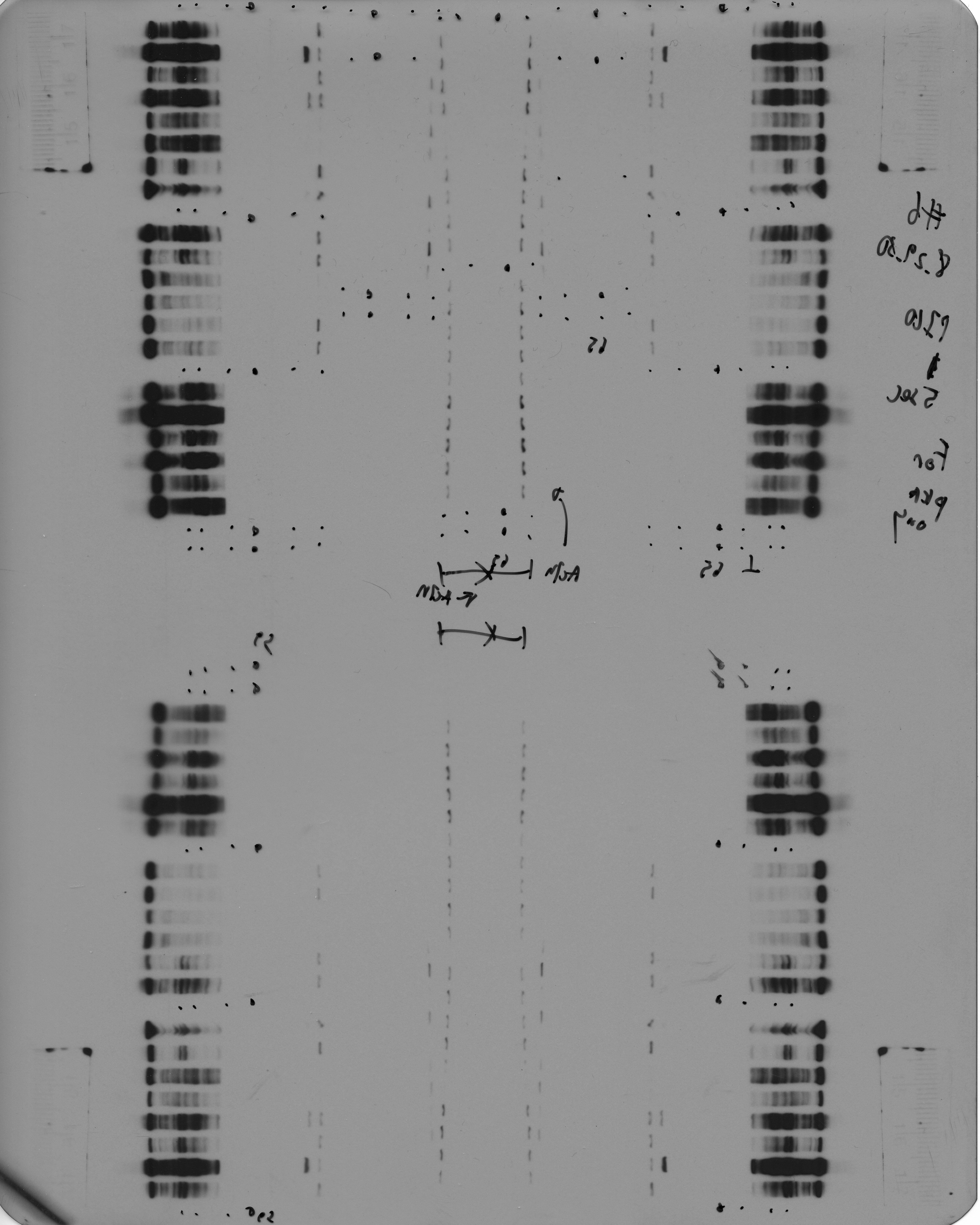

Supplement: Figure 1—source data 5. [file elife-69521-fig1-data5.zip › Figure 1D pPKA Subtrate Raw.tif]

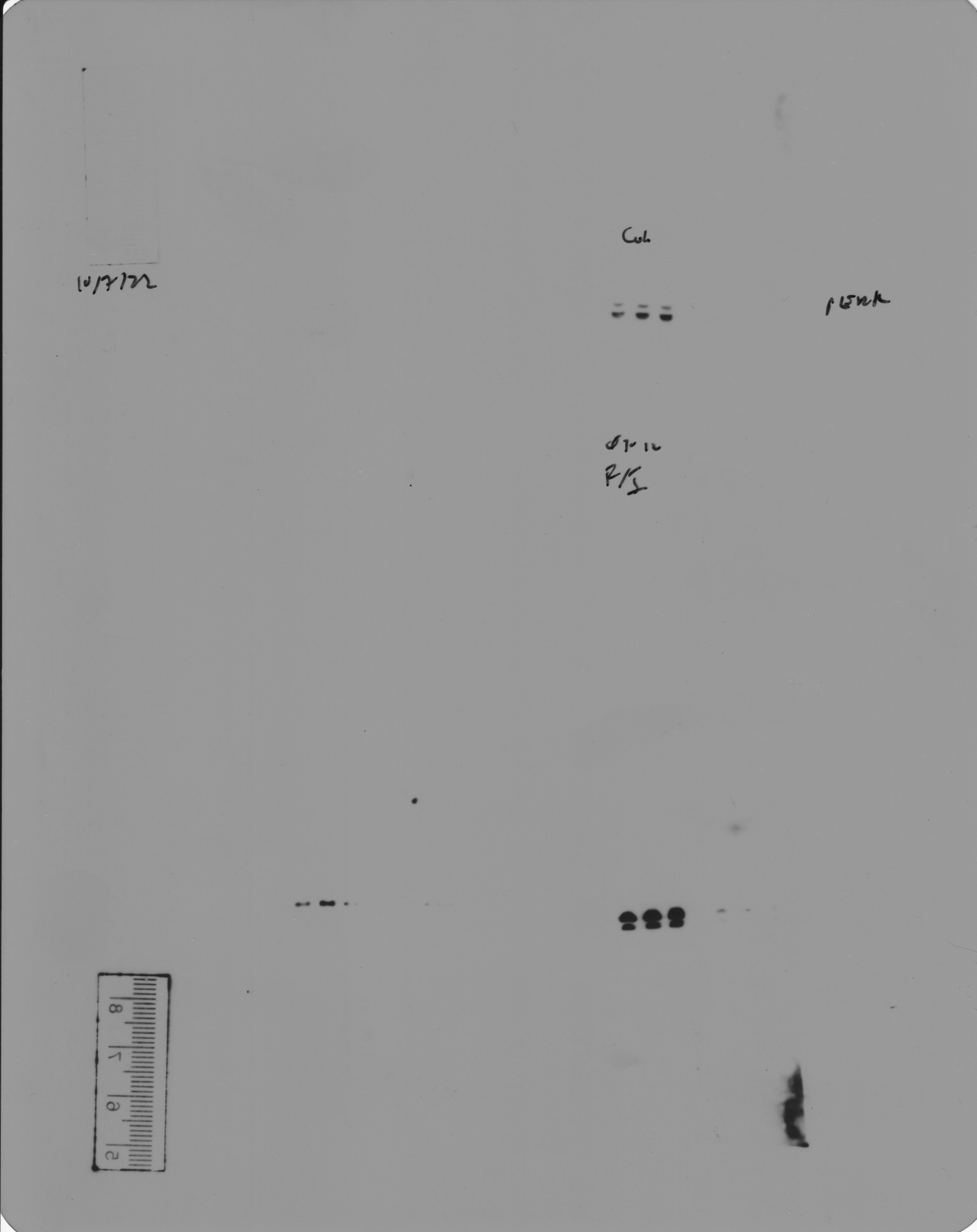

Supplement: Figure 2—source data 6. [file elife-69521-fig2-data6.zip › 2F/2F colo pERK raw.tif]

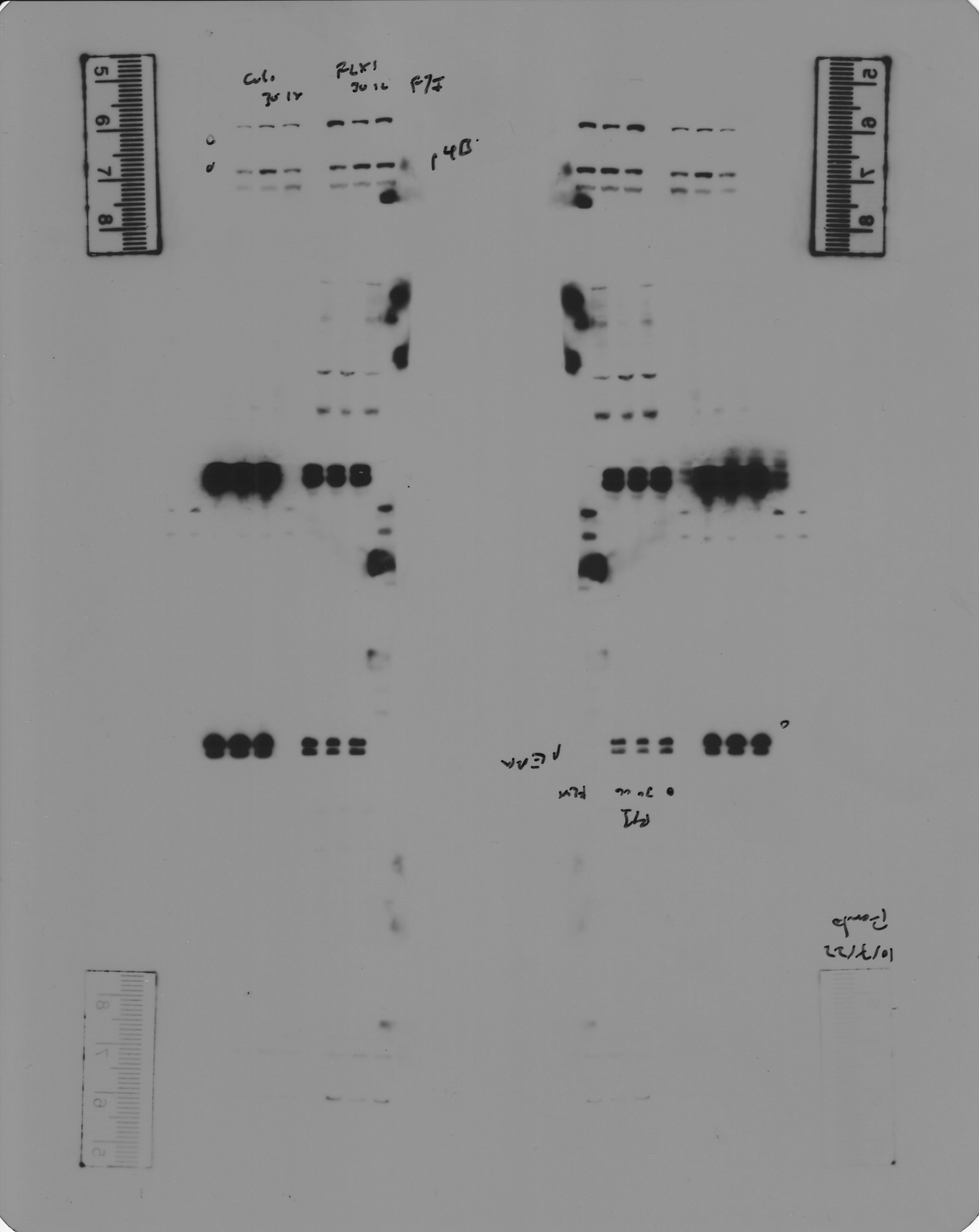

Supplement: Figure 2—source data 6. [file elife-69521-fig2-data6.zip › 2F/2F FLX1 pERK raw.tif]

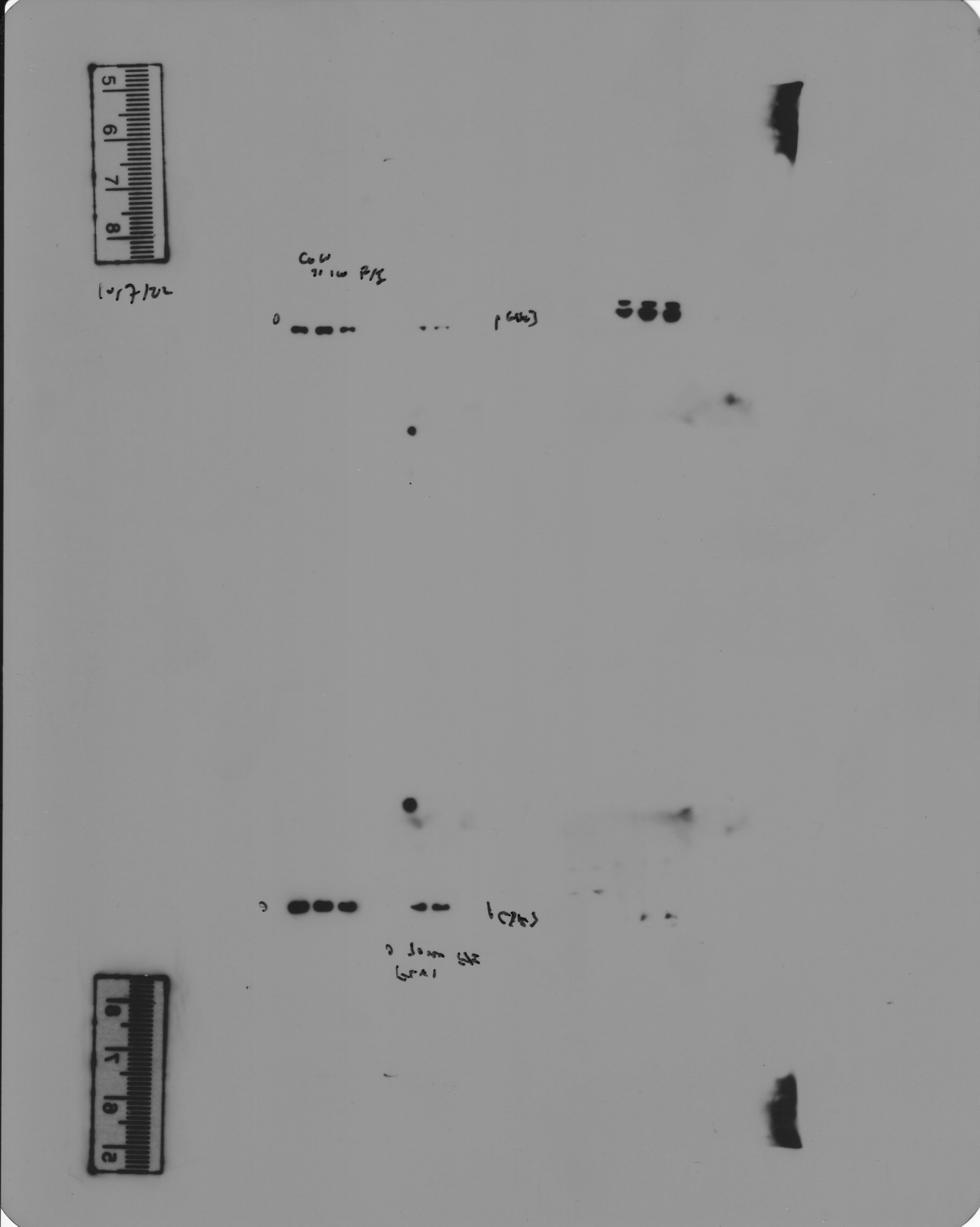

Supplement: Figure 2—source data 6. [file elife-69521-fig2-data6.zip › 2F/2F pGSK3 raw.tif]

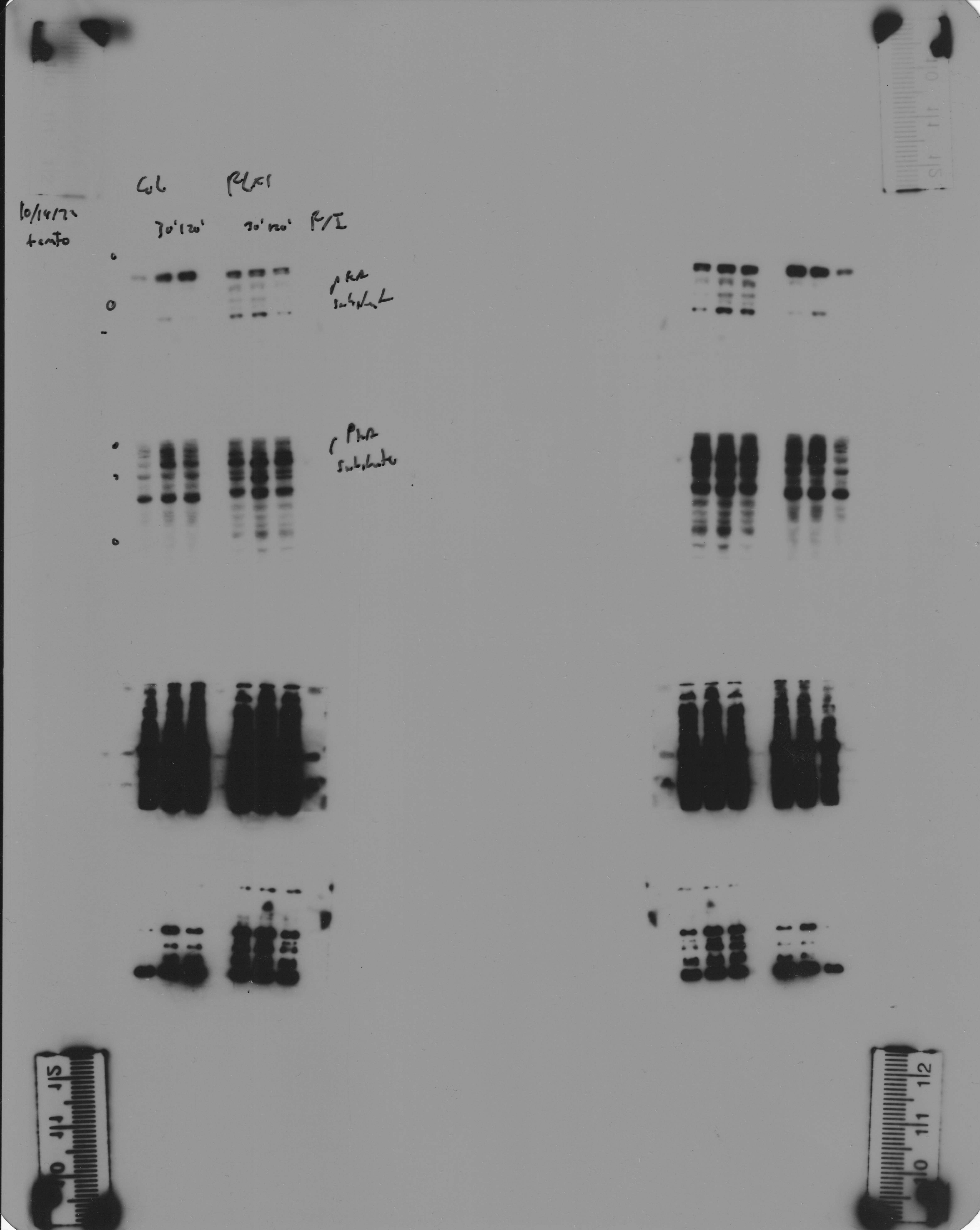

Supplement: Figure 2—source data 6. [file elife-69521-fig2-data6.zip › 2F/2F ppka substrate raw.tif]

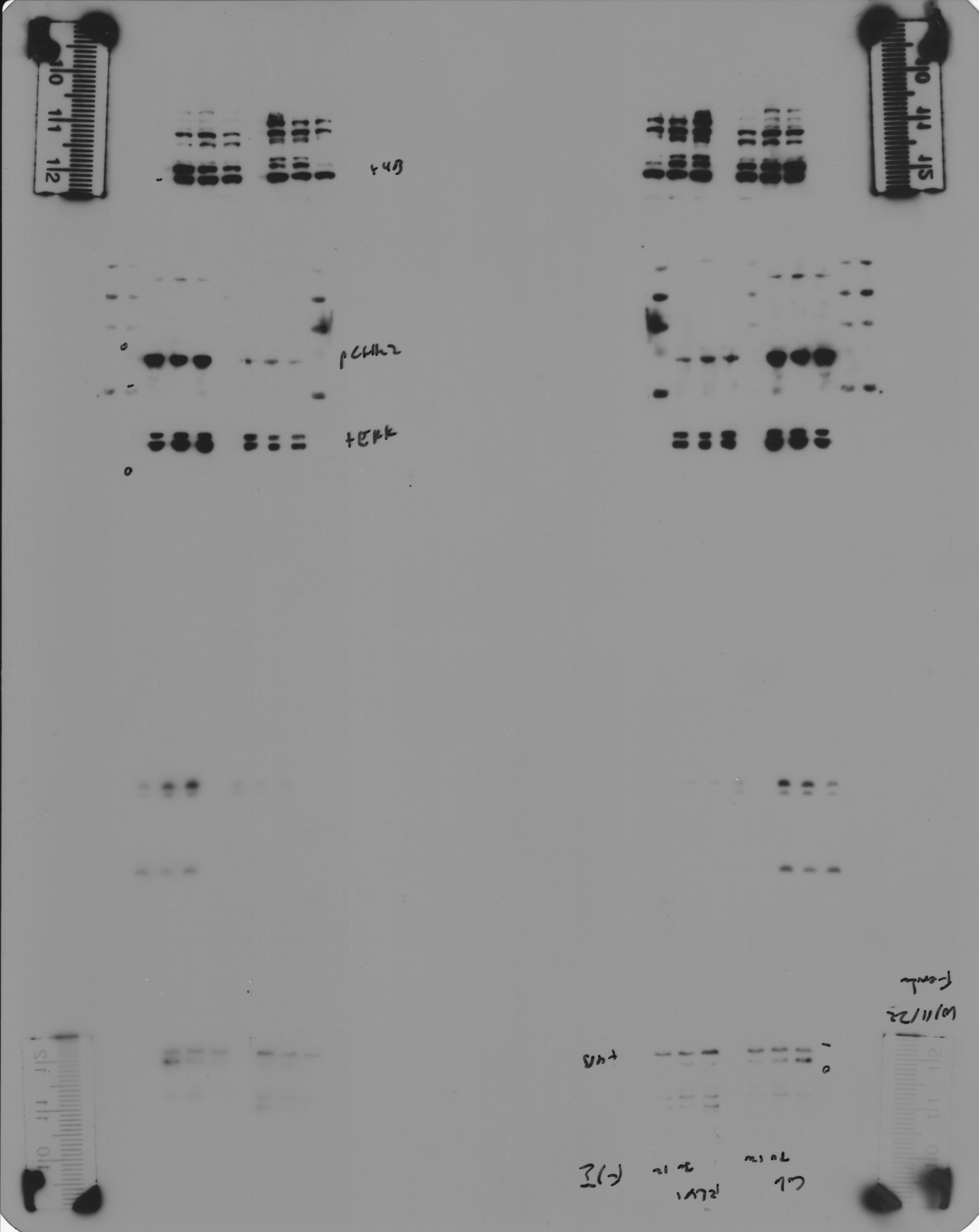

Supplement: Figure 2—source data 6. [file elife-69521-fig2-data6.zip › 2F/2F tERK raw.tif]

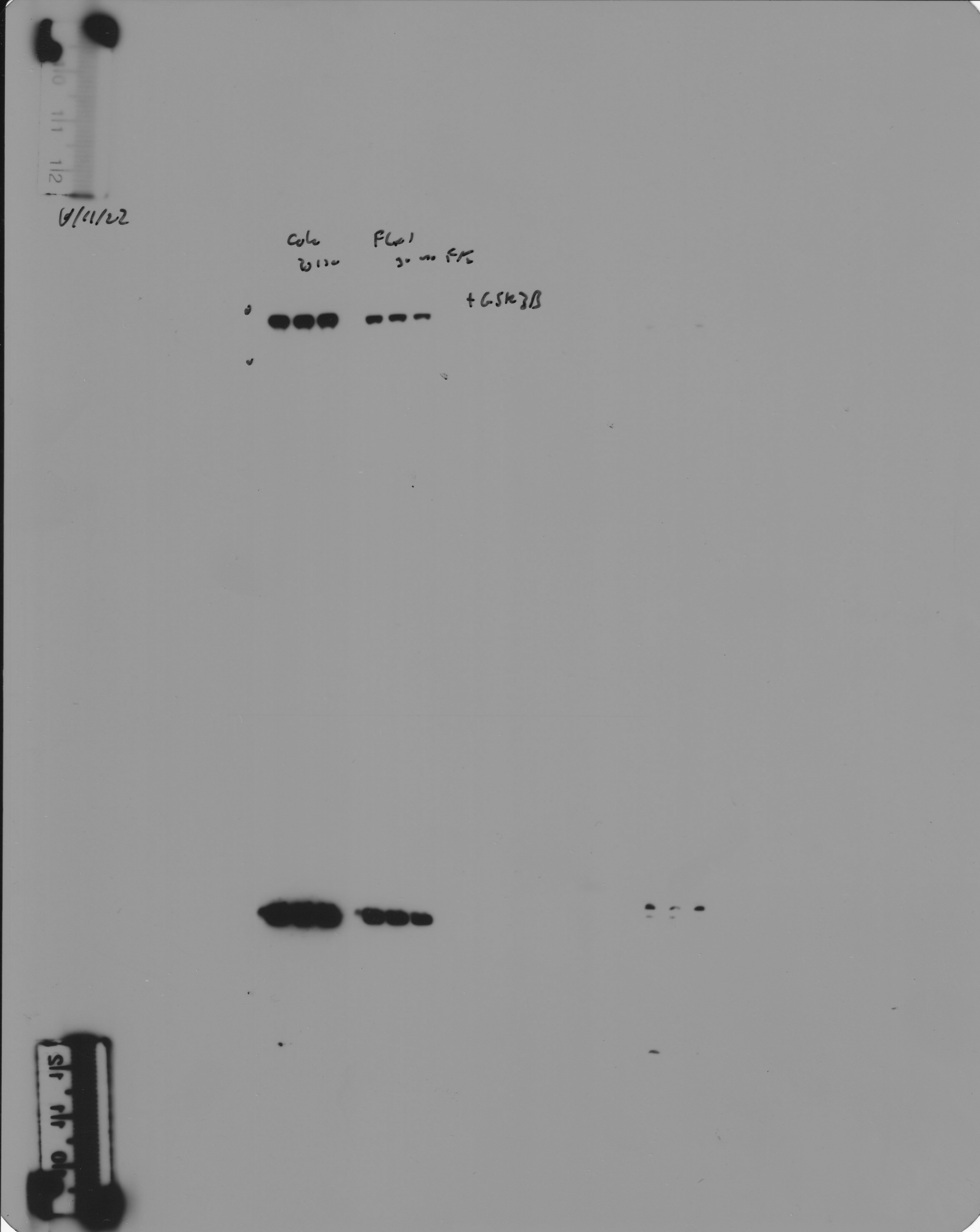

Supplement: Figure 2—source data 6. [file elife-69521-fig2-data6.zip › 2F/2F tGSK raw.tif]

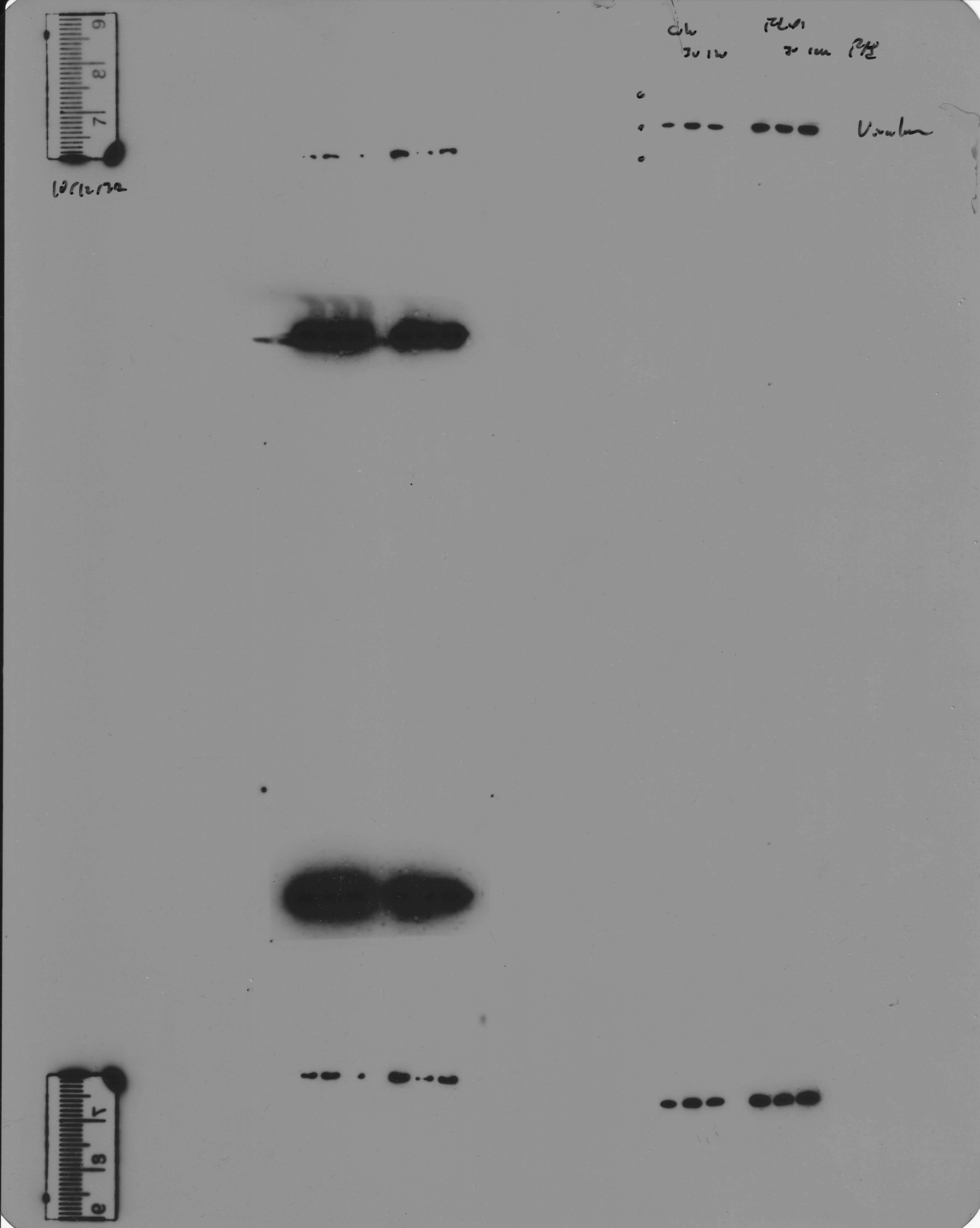

Supplement: Figure 2—source data 6. [file elife-69521-fig2-data6.zip › 2F/2F vinculin raw.tif]

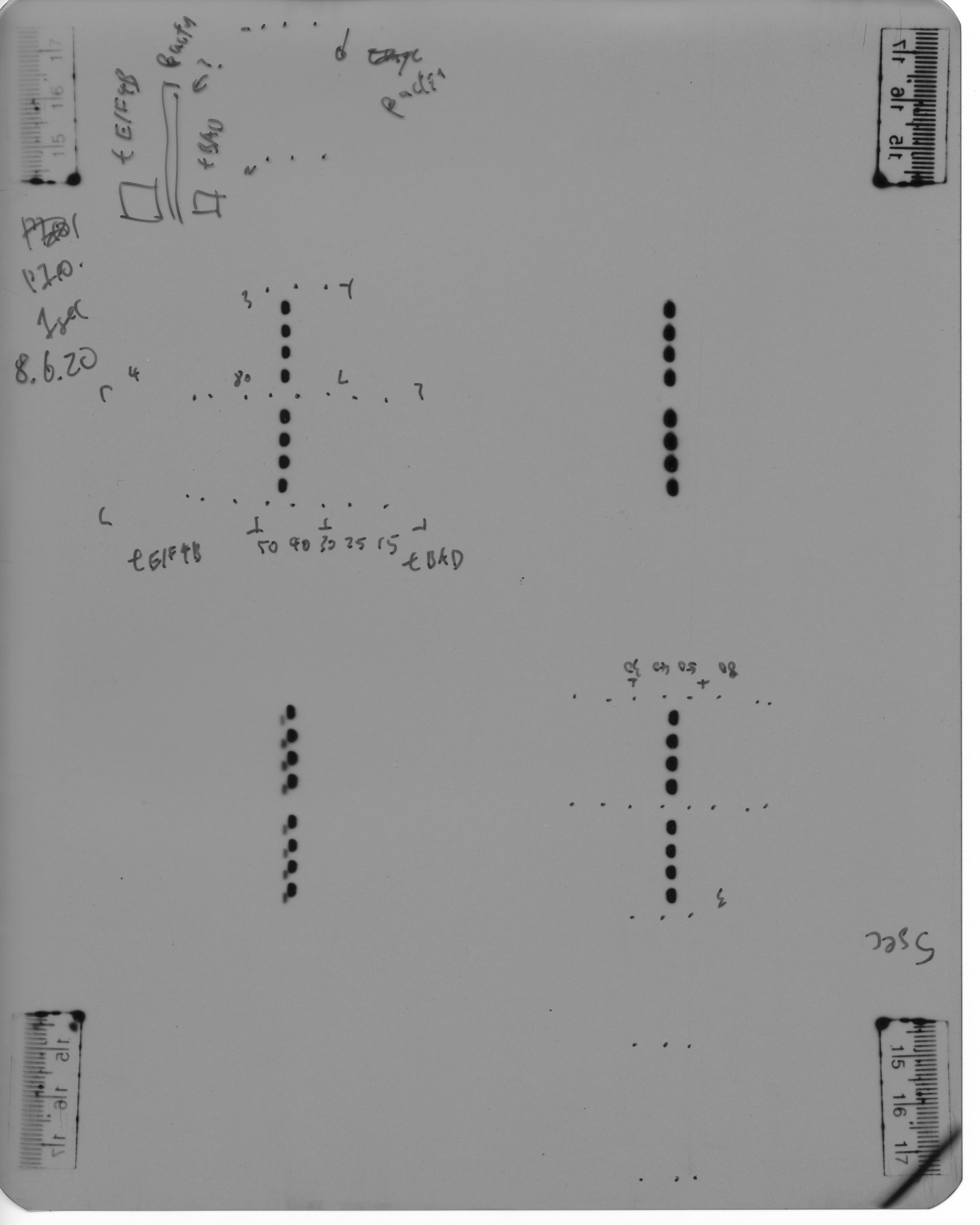

Supplement: Figure 3—source data 1. [file elife-69521-fig3-data1.zip › 3A/Colo741/Figure 3A Colo741 Actin Raw.tif]

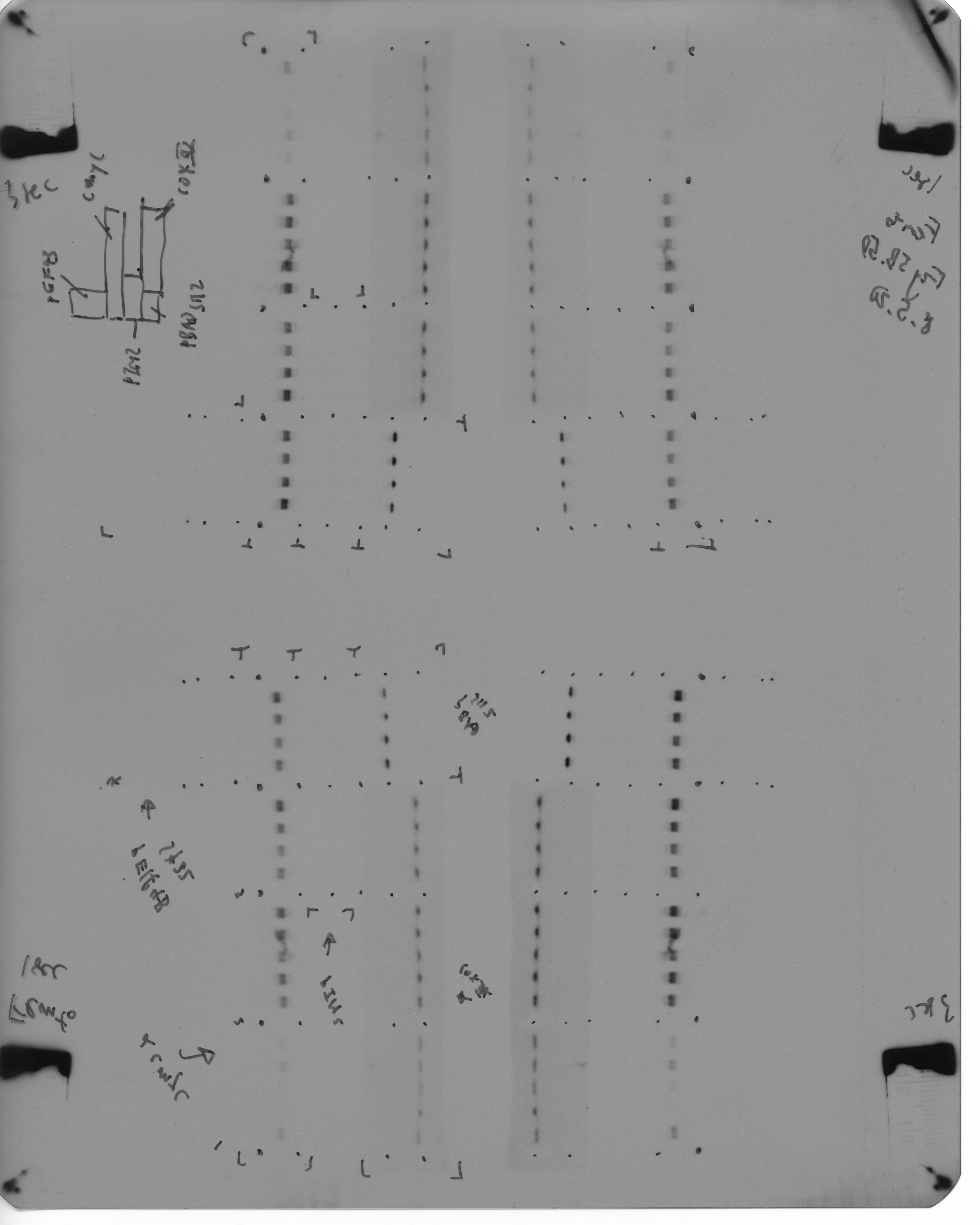

Supplement: Figure 3—source data 1. [file elife-69521-fig3-data1.zip › 3A/Colo741/Figure 3A Colo741 c-MYC Raw.tif]

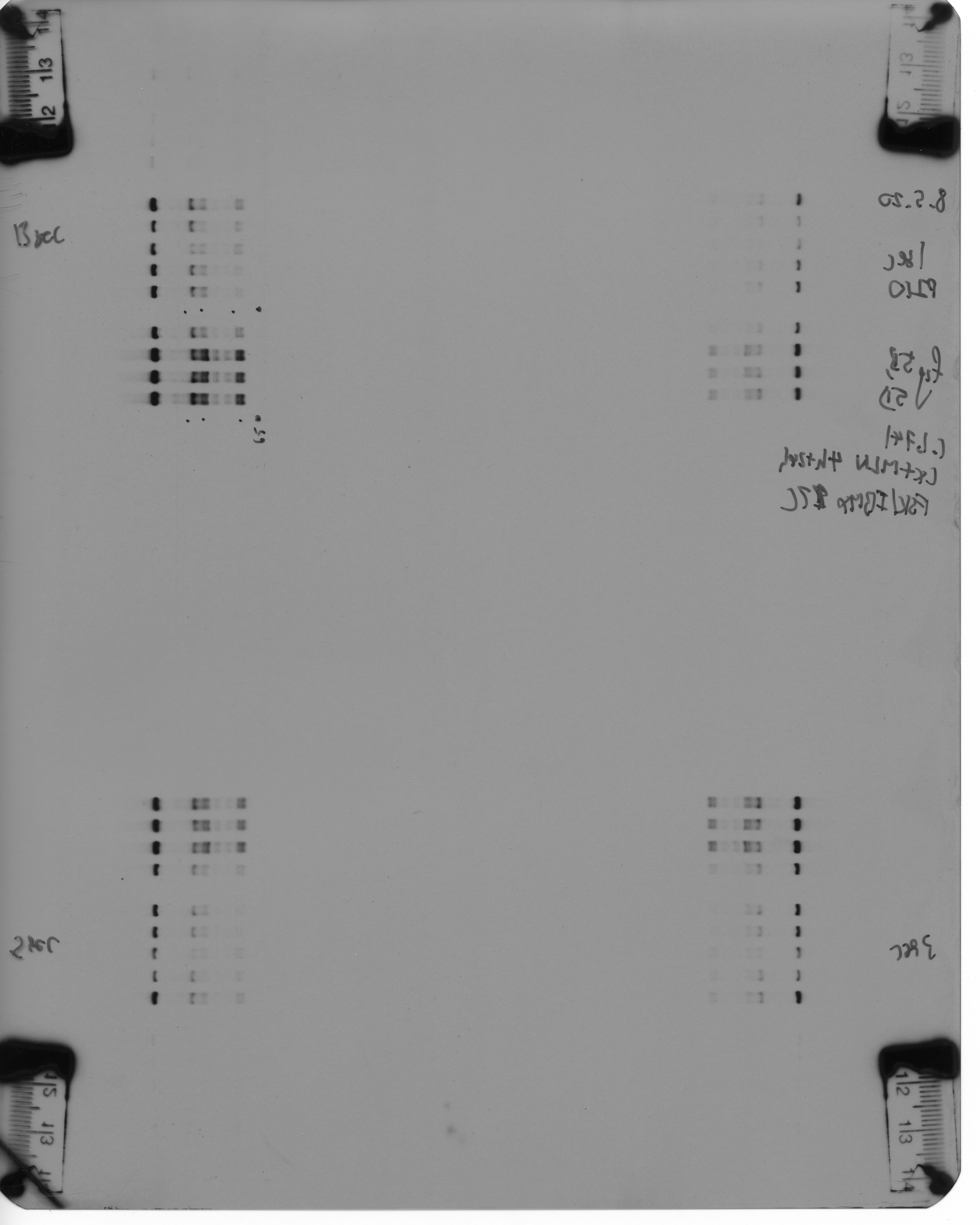

Supplement: Figure 3—source data 1. [file elife-69521-fig3-data1.zip › 3A/Colo741/Figure 3A Colo741 pPKA Sub Raw.tif]

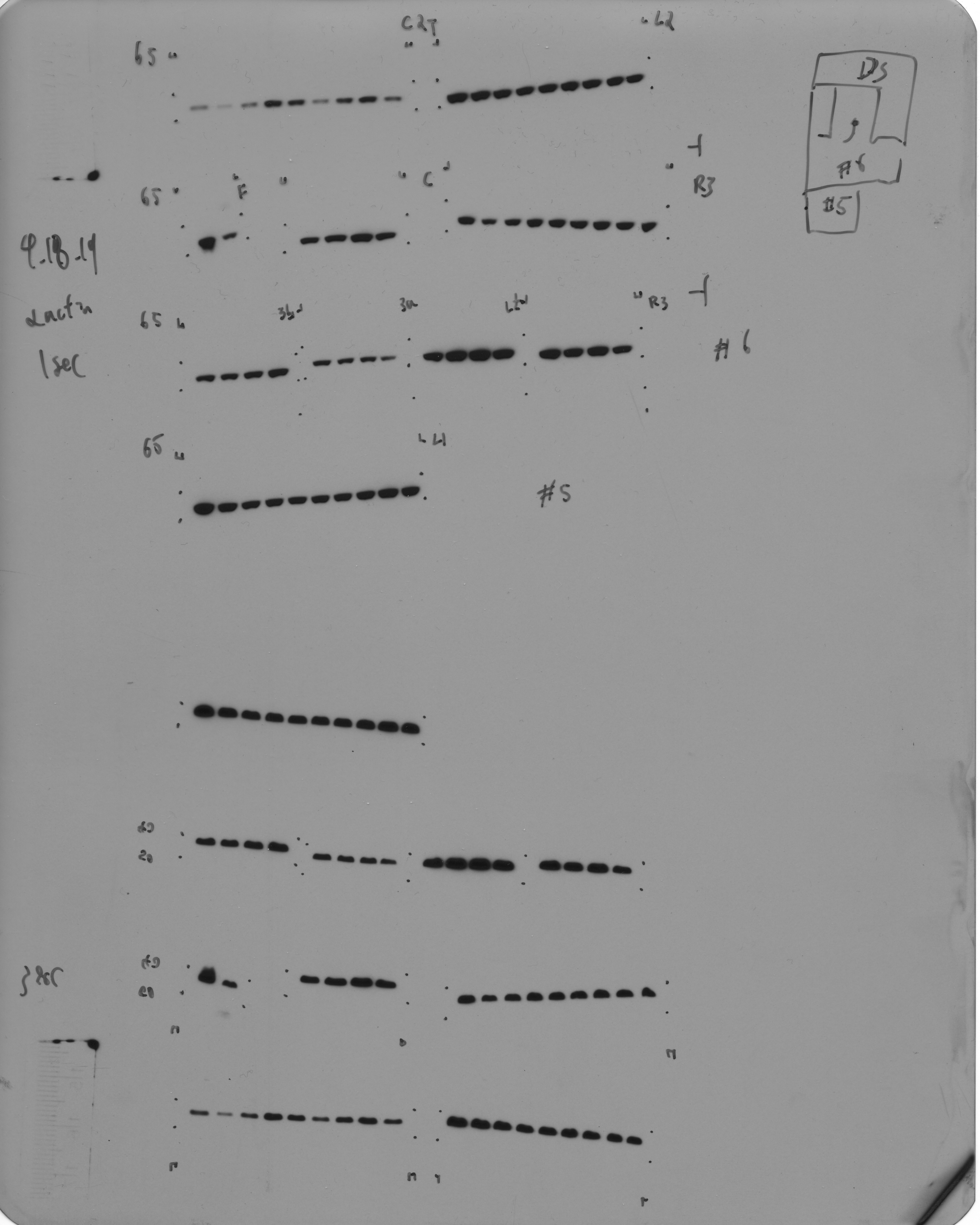

Supplement: Figure 3—source data 1. [file elife-69521-fig3-data1.zip › 3A/FLX1/Figure 3A FLX11 Actin Raw.tif]

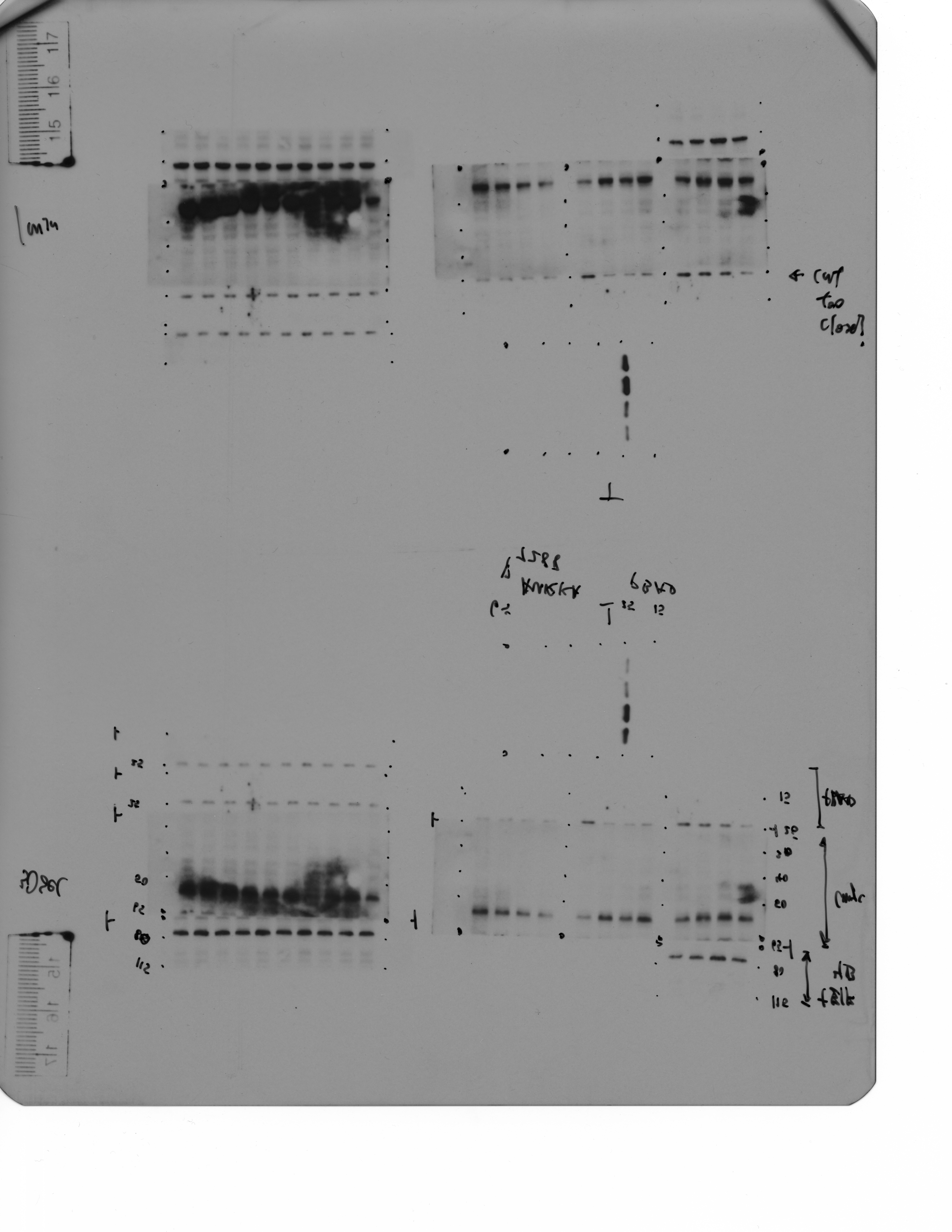

Supplement: Figure 3—source data 1. [file elife-69521-fig3-data1.zip › 3A/FLX1/Figure 3A FLX11 c-MYC Raw.tif]

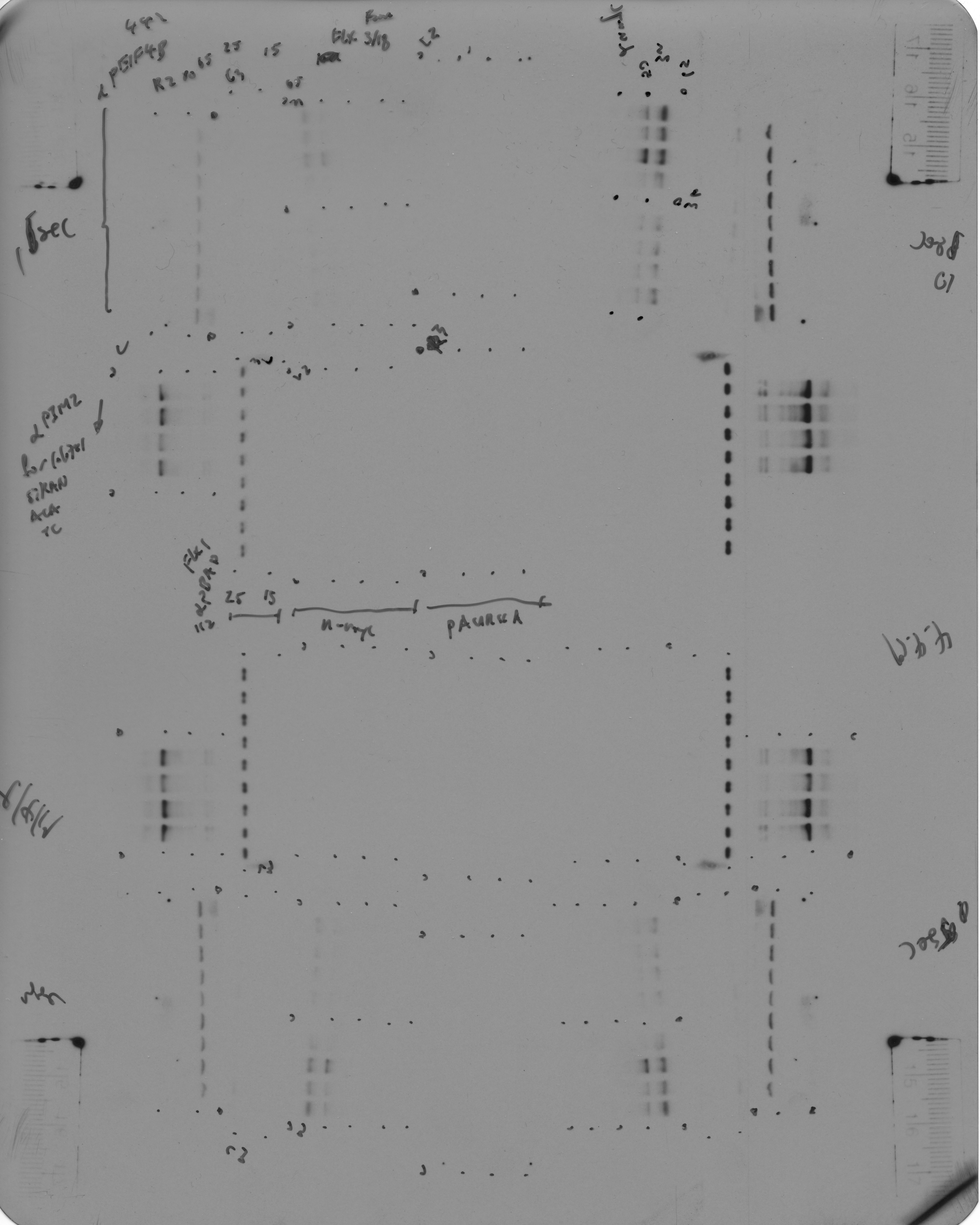

Supplement: Figure 3—source data 1. [file elife-69521-fig3-data1.zip › 3A/FLX1/Figure 3A FLX11 n-MYC Raw.tif]

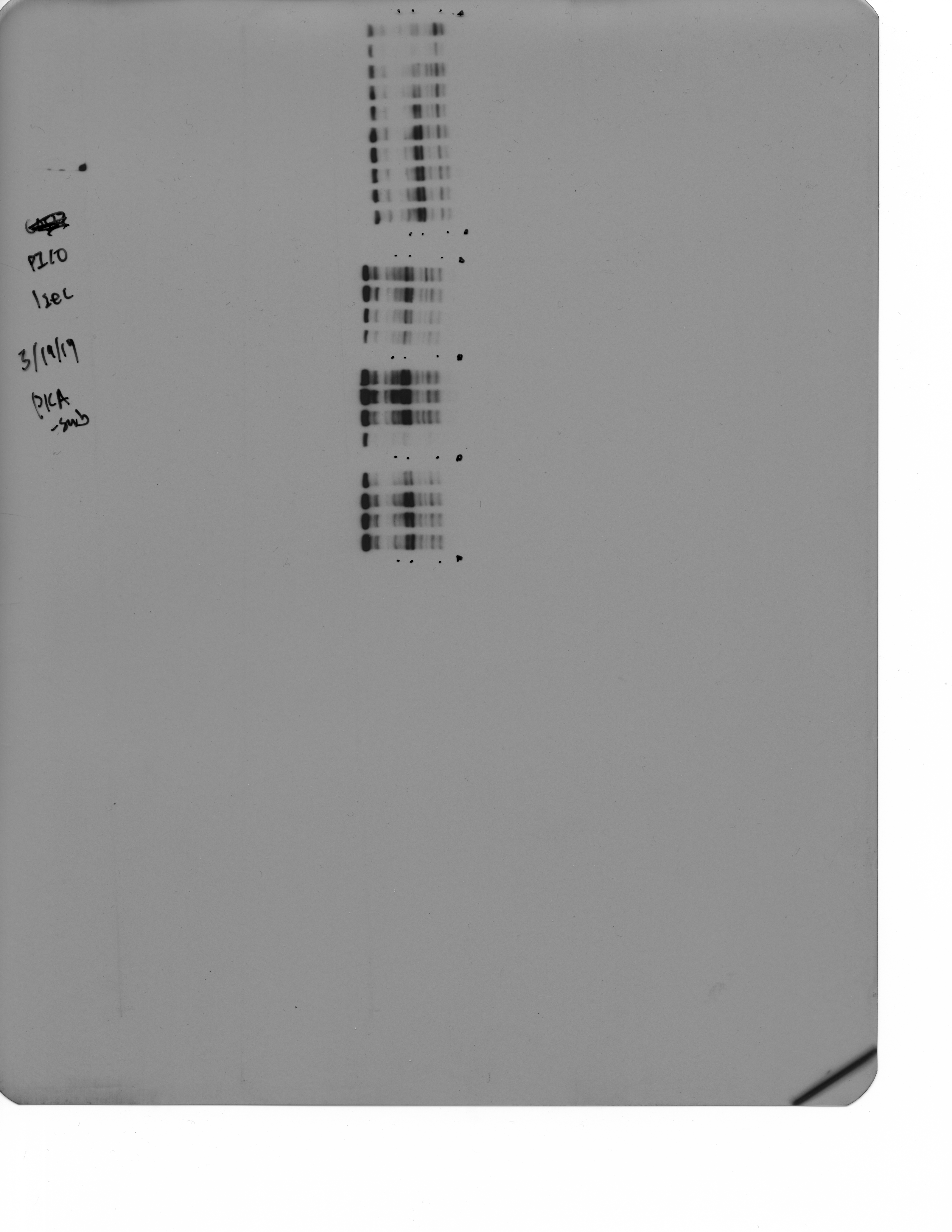

Supplement: Figure 3—source data 1. [file elife-69521-fig3-data1.zip › 3A/FLX1/Figure 3A FLX11 pPKA Substrate Raw.tif]

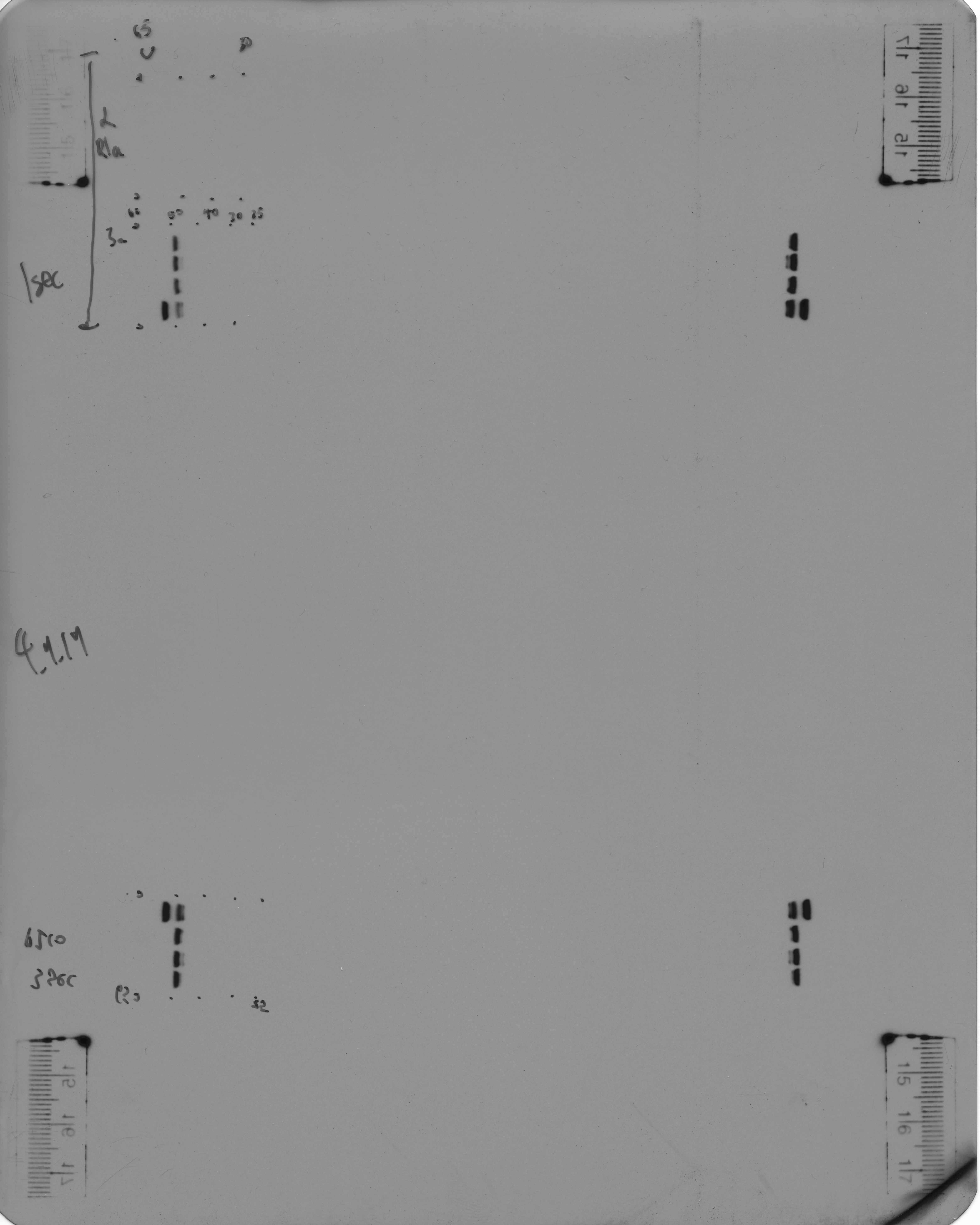

Supplement: Figure 3—source data 3. [file elife-69521-fig3-data3.zip › 3C/Figure 3C FLX1 PKAR1a Raw.tif]

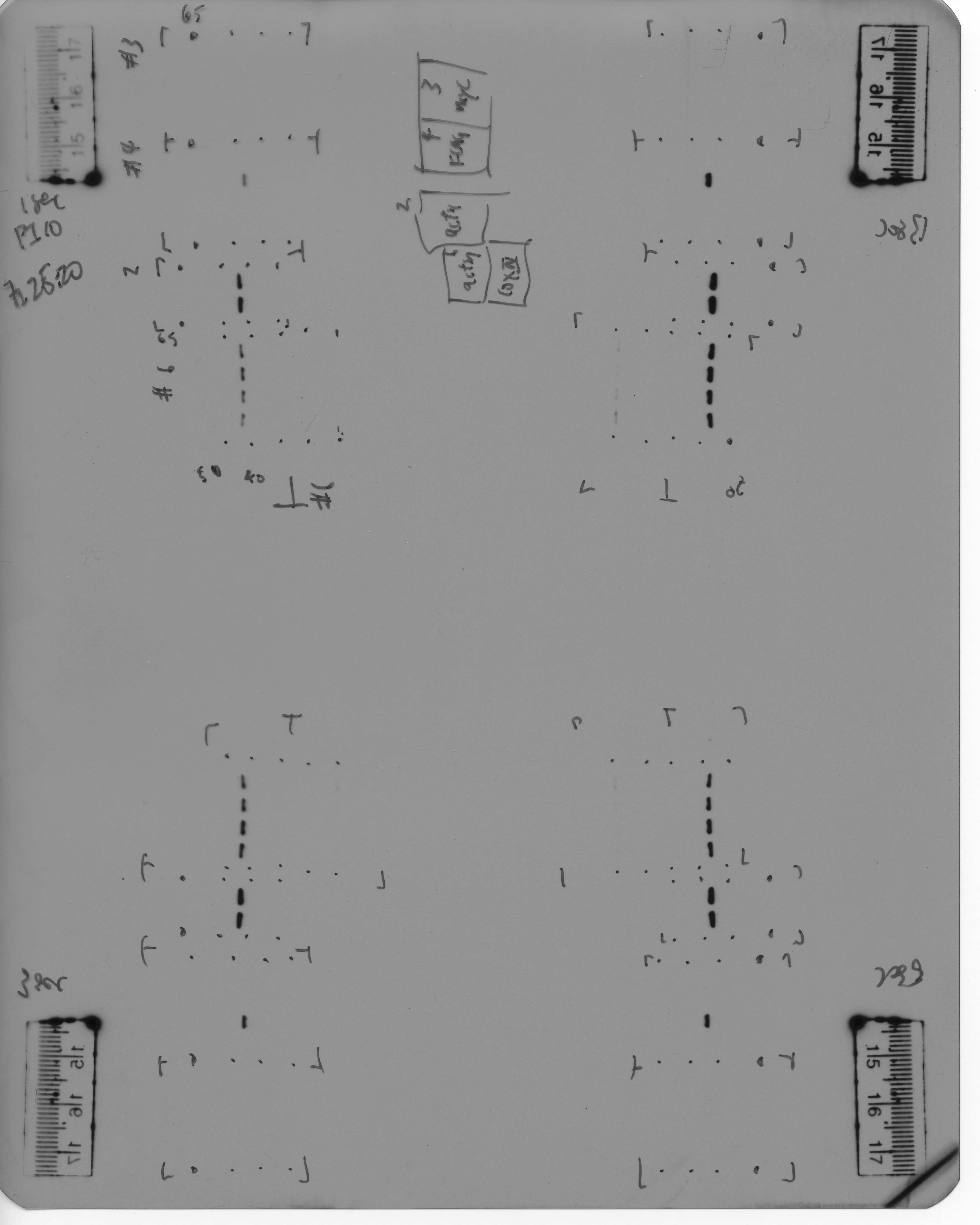

Supplement: Figure 3—source data 4. [file elife-69521-fig3-data4.zip › 3D/Figure 3D AML12 Actin Raw.tif]

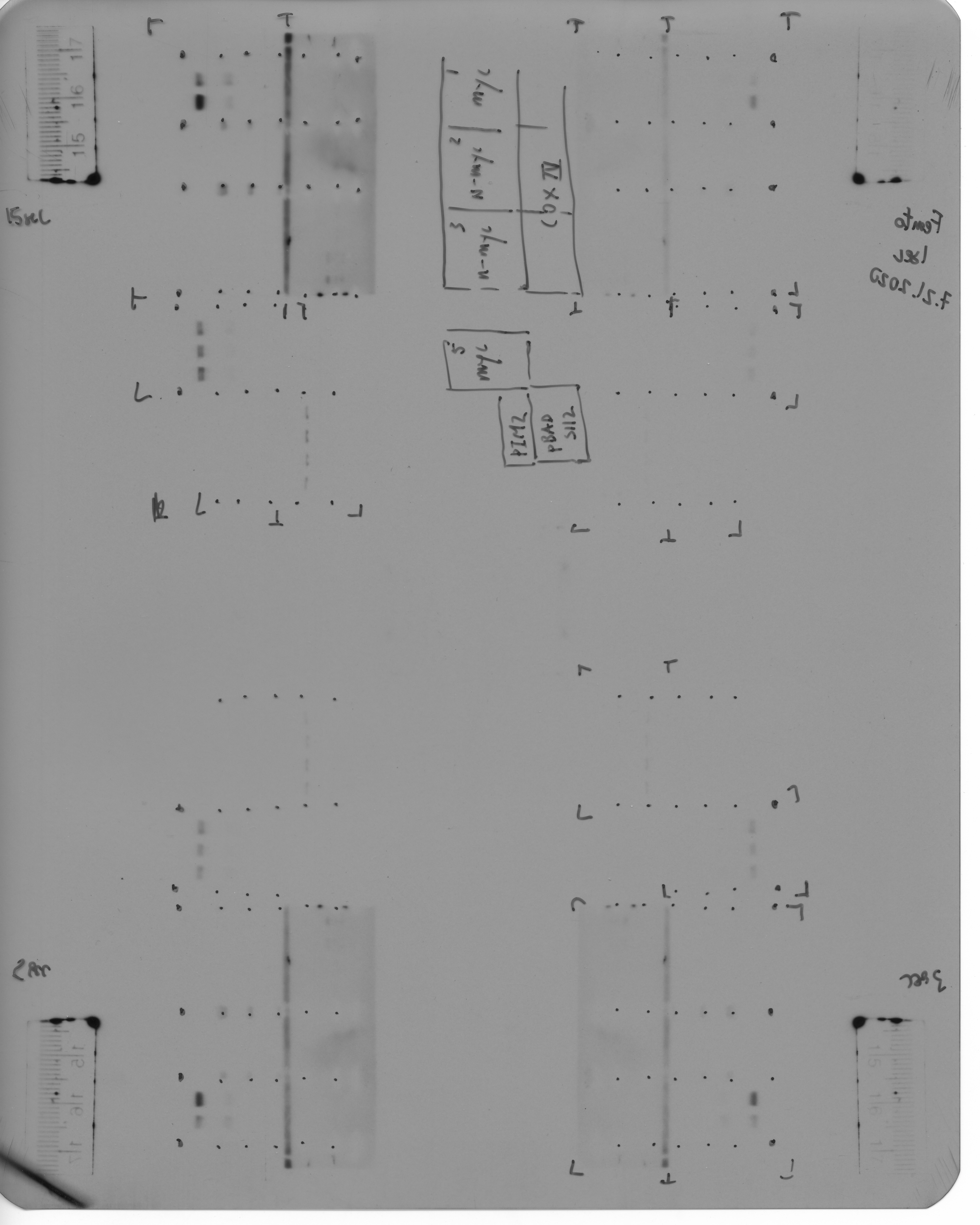

Supplement: Figure 3—source data 4. [file elife-69521-fig3-data4.zip › 3D/Figure 3D AML12 c-MYC Raw.tif]

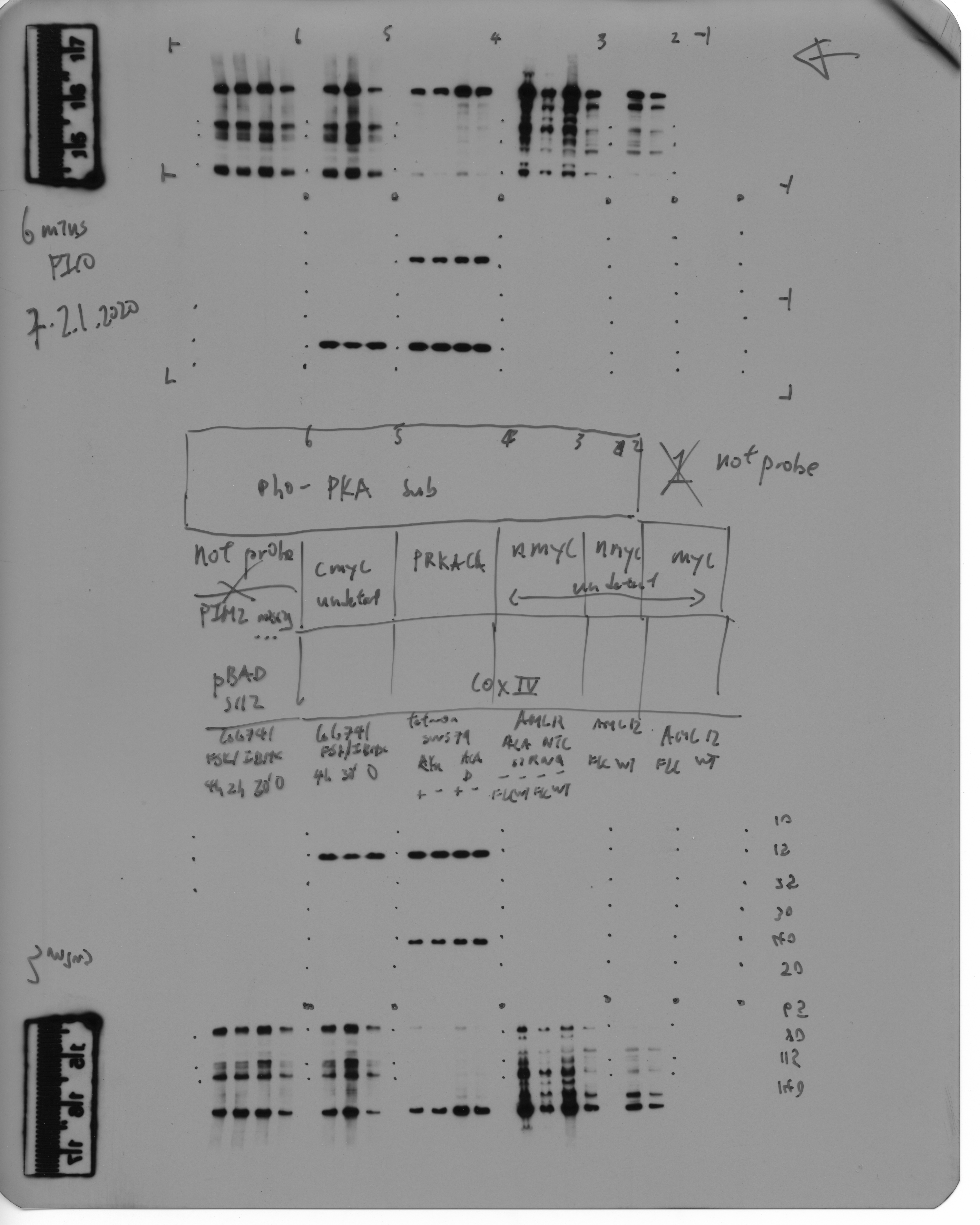

Supplement: Figure 3—source data 4. [file elife-69521-fig3-data4.zip › 3D/Figure 3D AML12 pPKA Substrate Raw.tif]

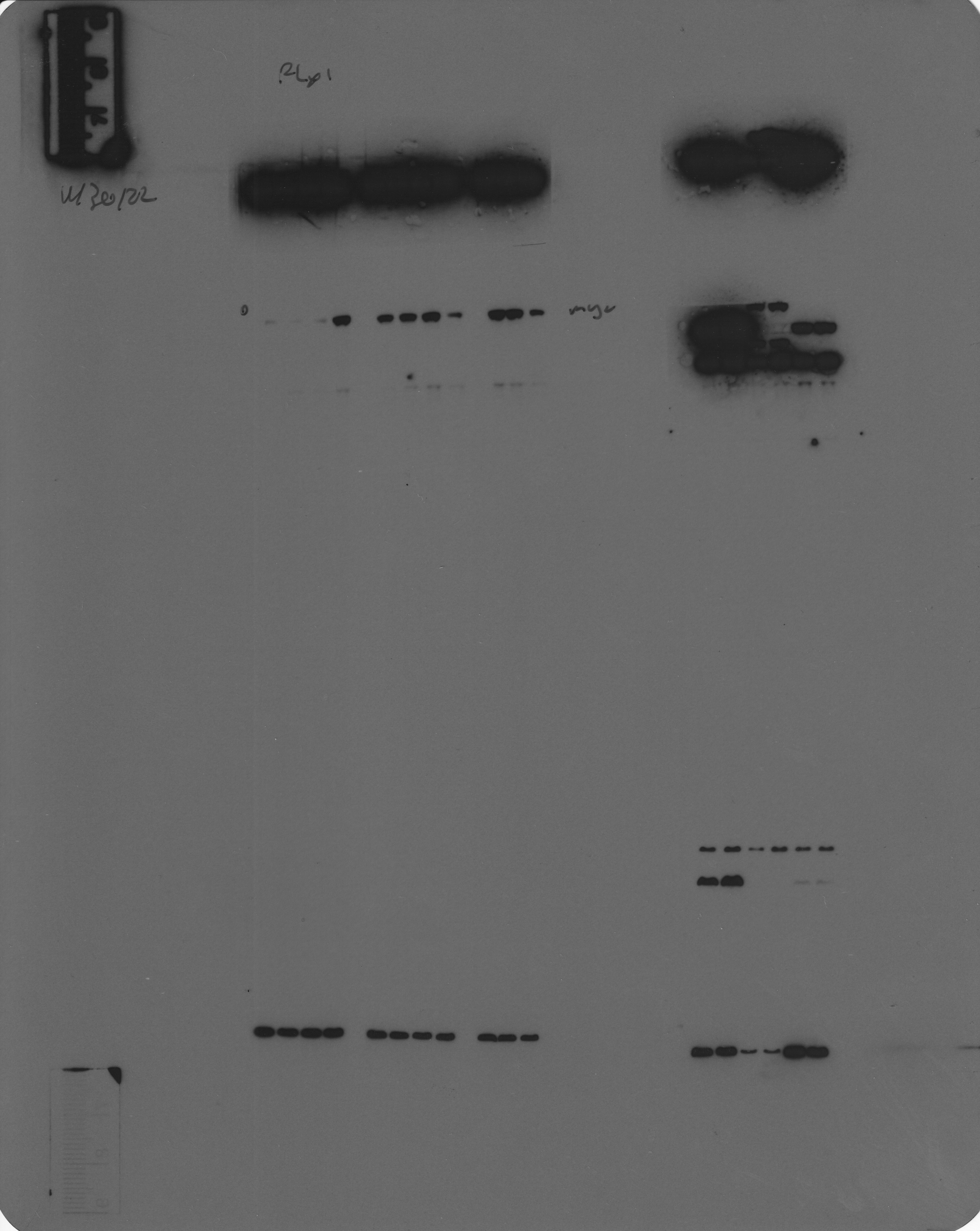

Supplement: Figure 3—source data 5. [file elife-69521-fig3-data5.zip › 3E/3E MYC.tif]

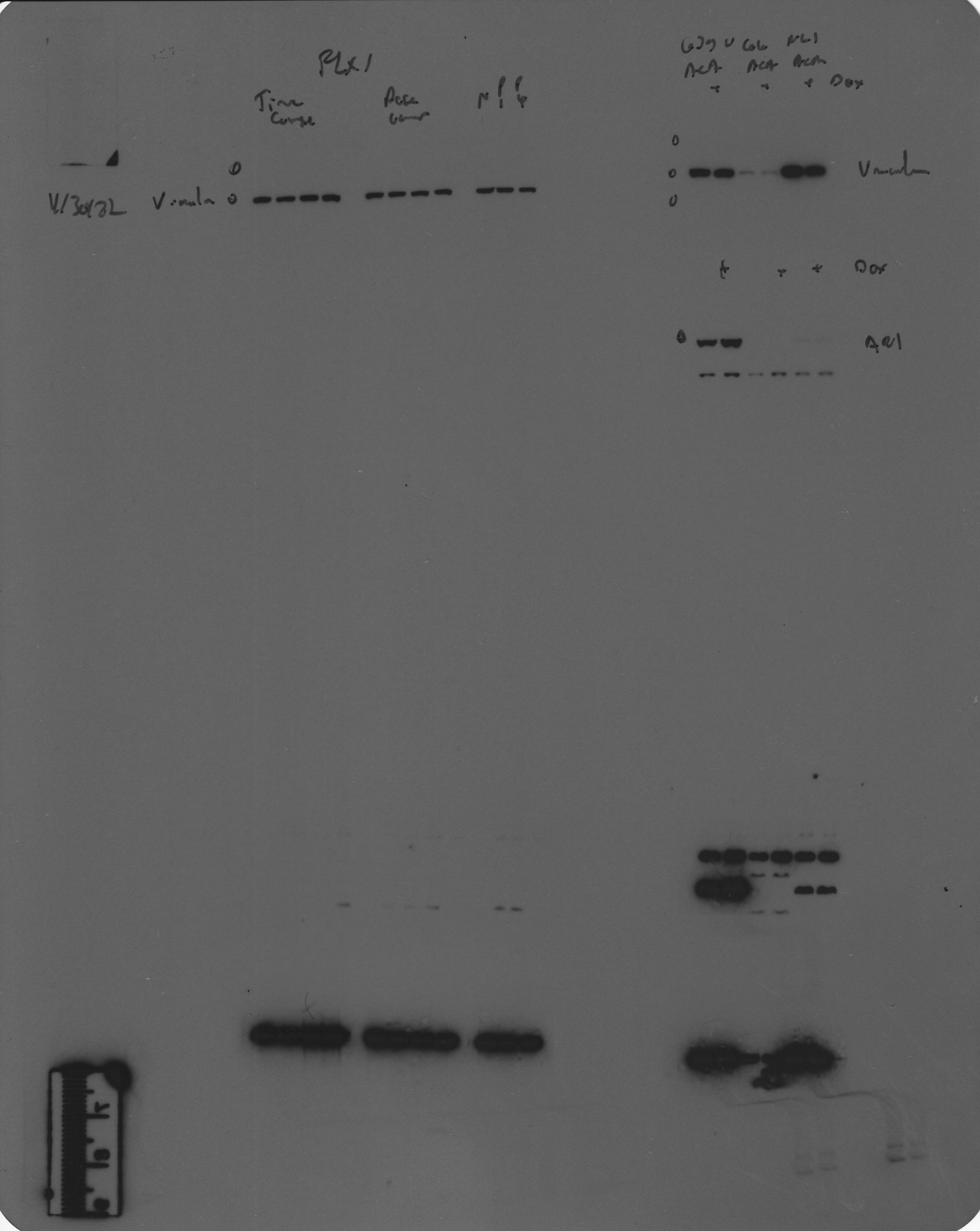

Supplement: Figure 3—source data 5. [file elife-69521-fig3-data5.zip › 3E/3E vinculin raw.tif]

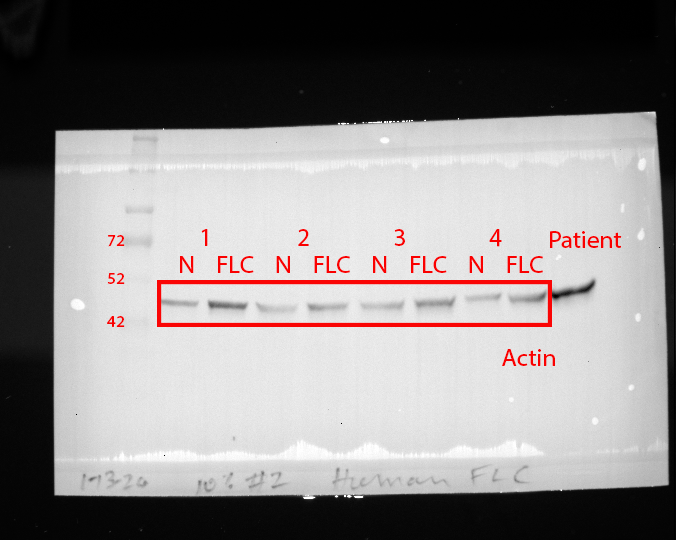

Supplement: Figure 3—source data 6. [file elife-69521-fig3-data6.zip › 3F/Figure 3E Actin Labelled.tif]

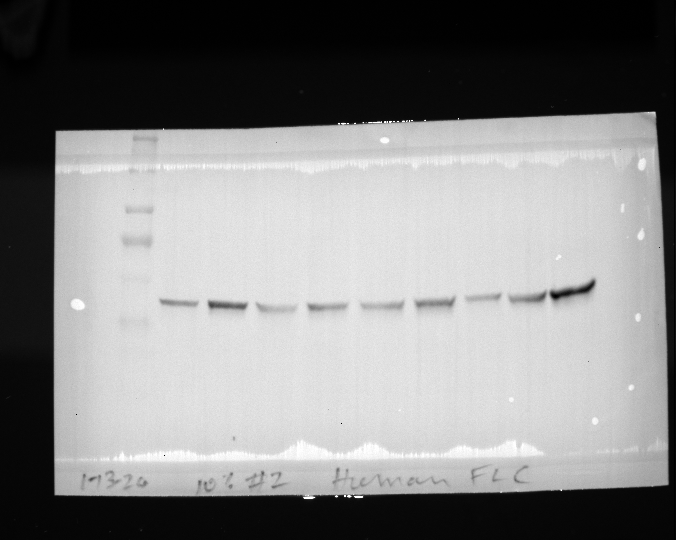

Supplement: Figure 3—source data 6. [file elife-69521-fig3-data6.zip › 3F/Figure 3E Actin Raw.tif]

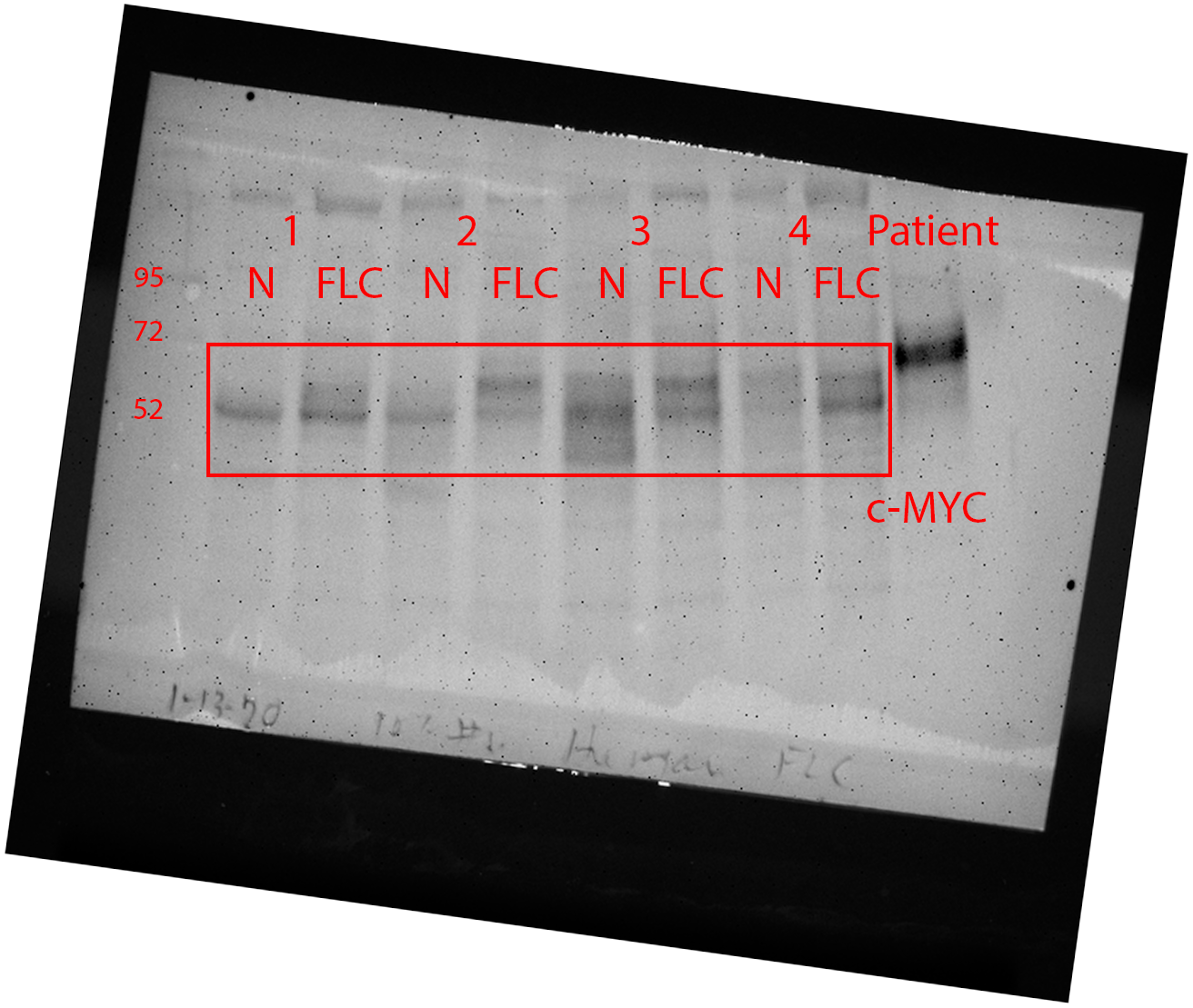

Supplement: Figure 3—source data 6. [file elife-69521-fig3-data6.zip › 3F/Figure 3E c-MYC Labelled.tif]

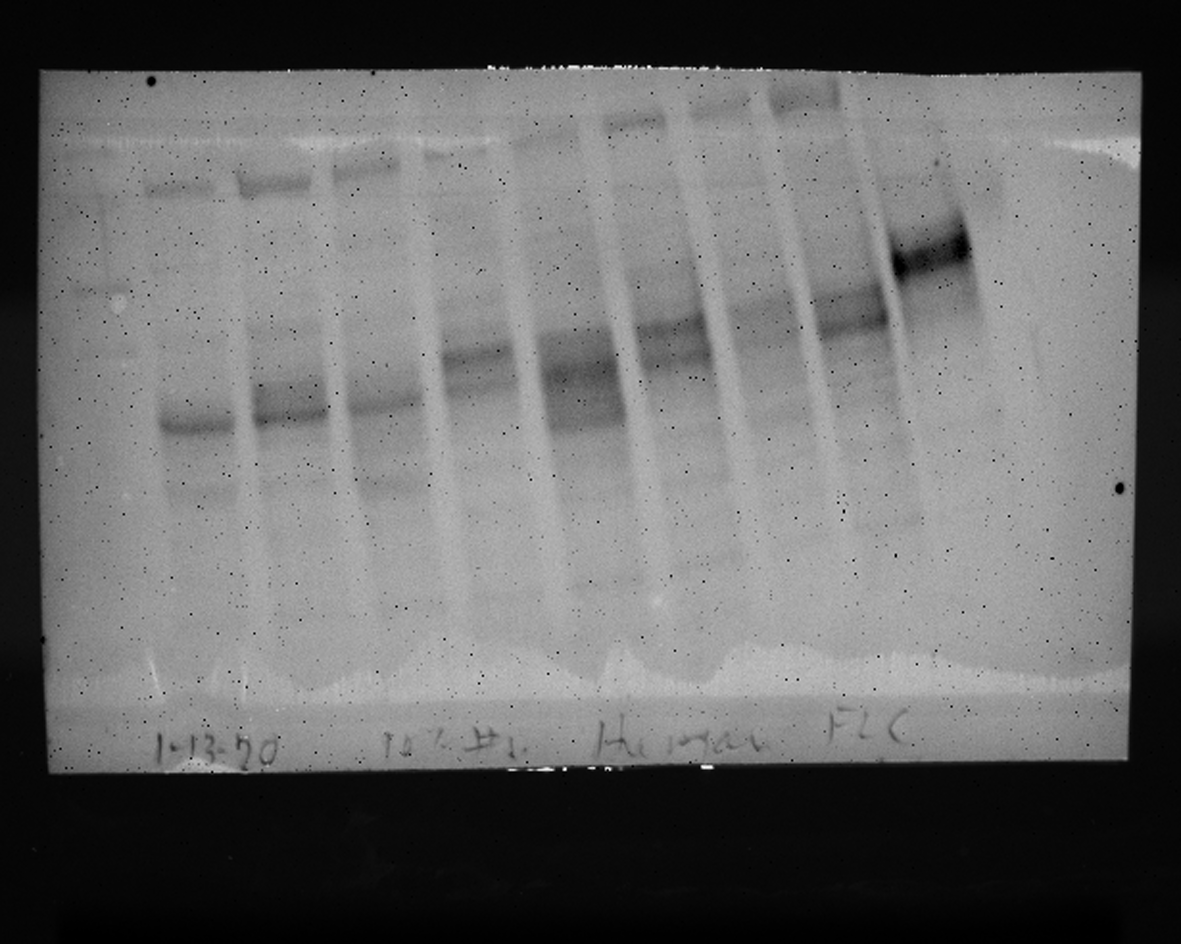

Supplement: Figure 3—source data 6. [file elife-69521-fig3-data6.zip › 3F/Figure 3E c-MYC Raw.tif]

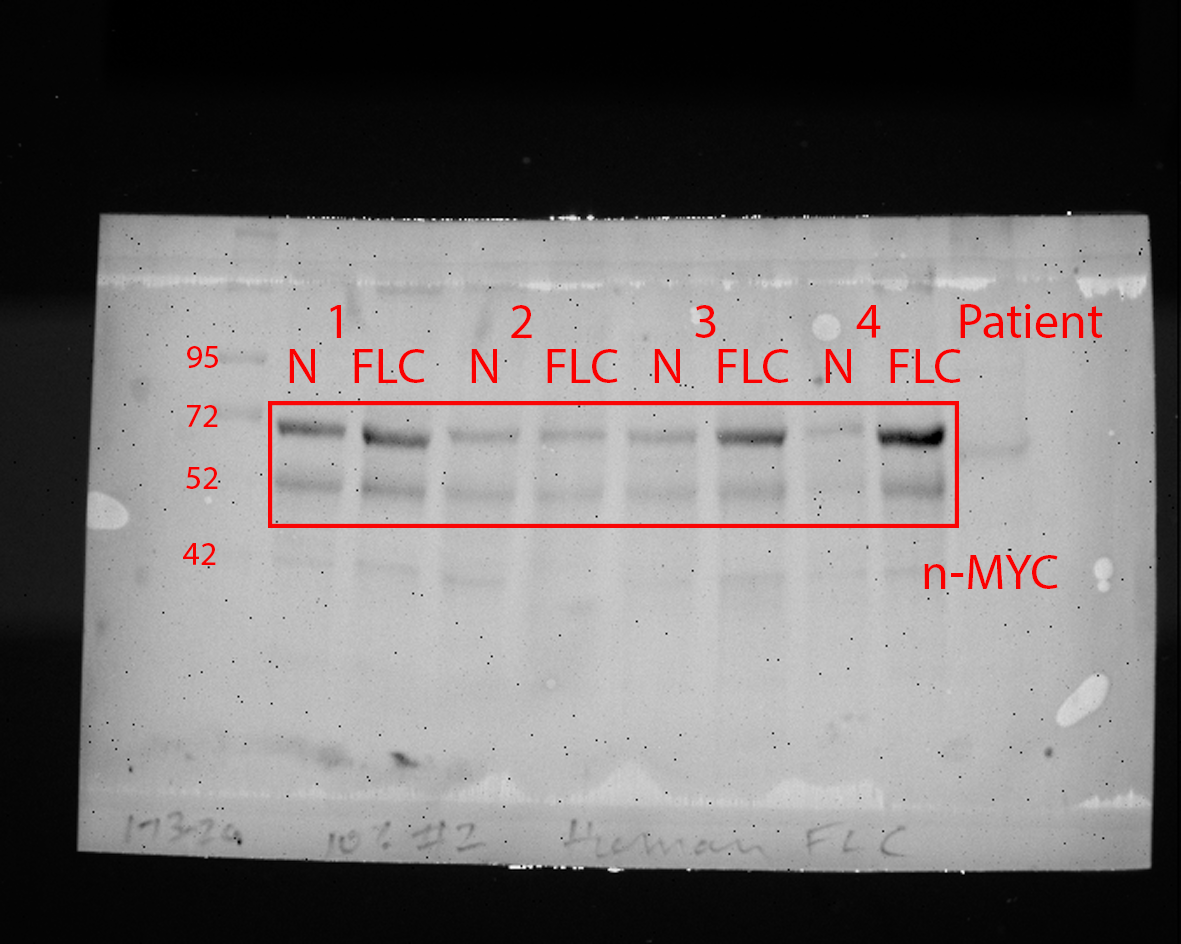

Supplement: Figure 3—source data 6. [file elife-69521-fig3-data6.zip › 3F/Figure 3E n-MYC Labelled.tif]

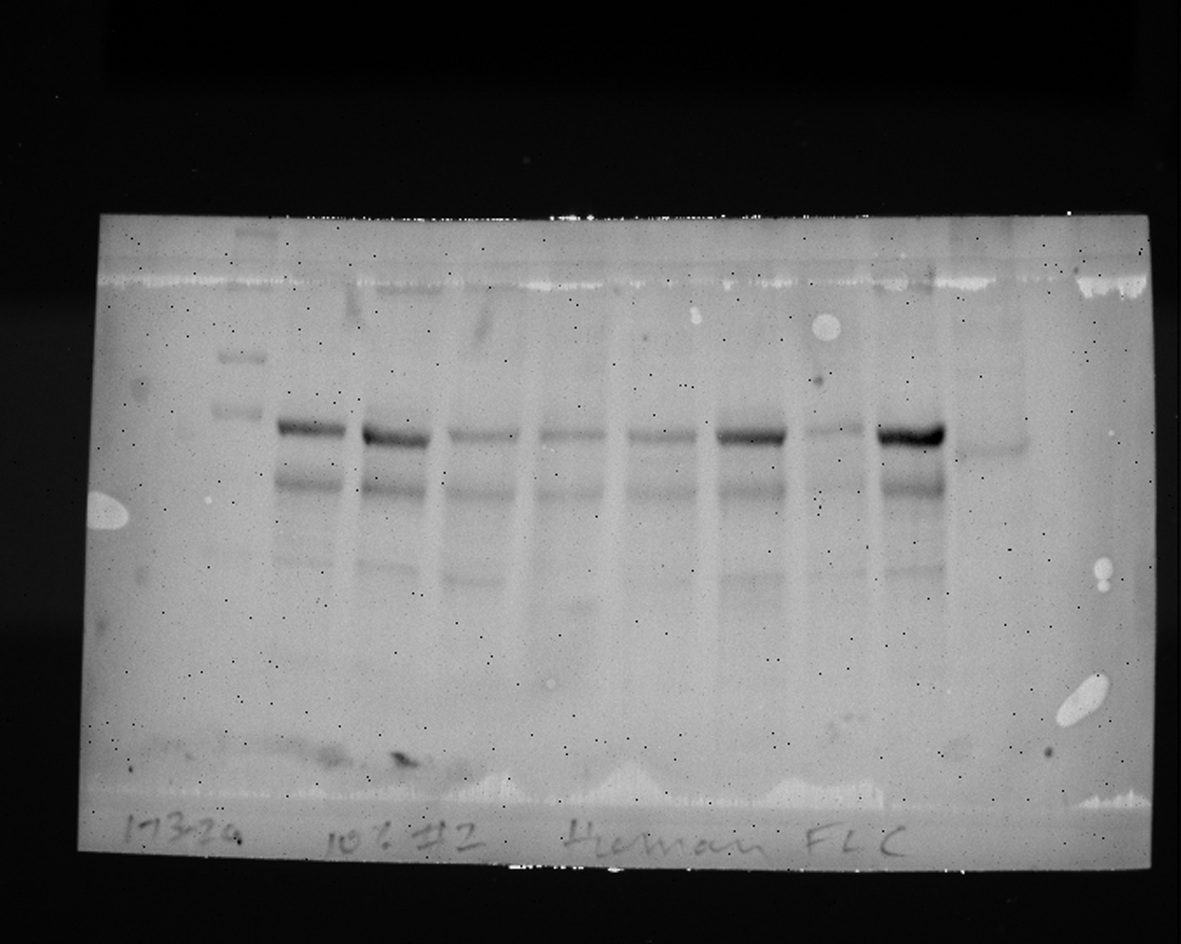

Supplement: Figure 3—source data 6. [file elife-69521-fig3-data6.zip › 3F/Figure 3E n-MYC Raw.tif]

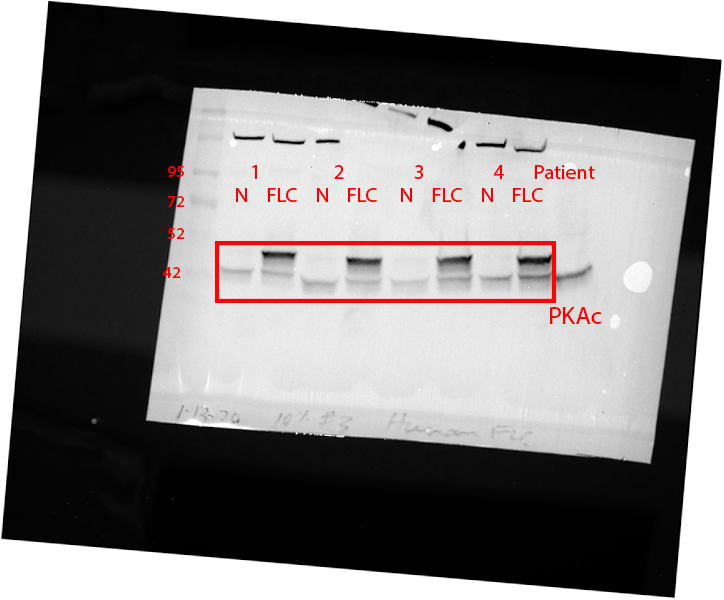

Supplement: Figure 3—source data 6. [file elife-69521-fig3-data6.zip › 3F/Figure 3E PKAc Labelled.tif]

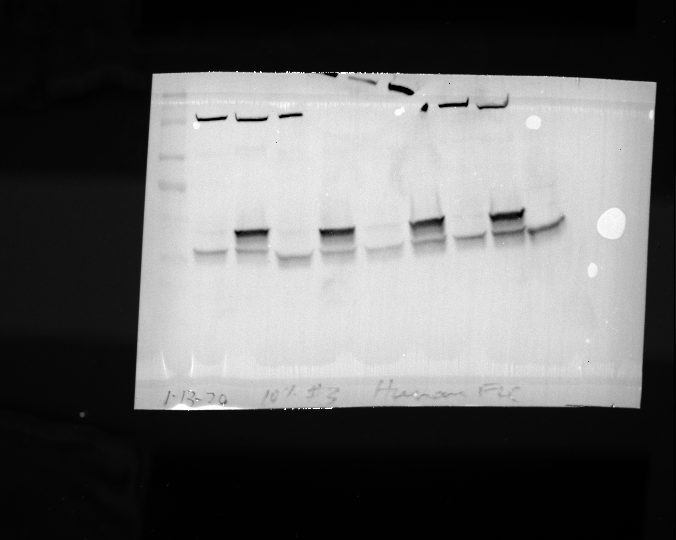

Supplement: Figure 3—source data 6. [file elife-69521-fig3-data6.zip › 3F/Figure 3E PKAc Raw.tif]

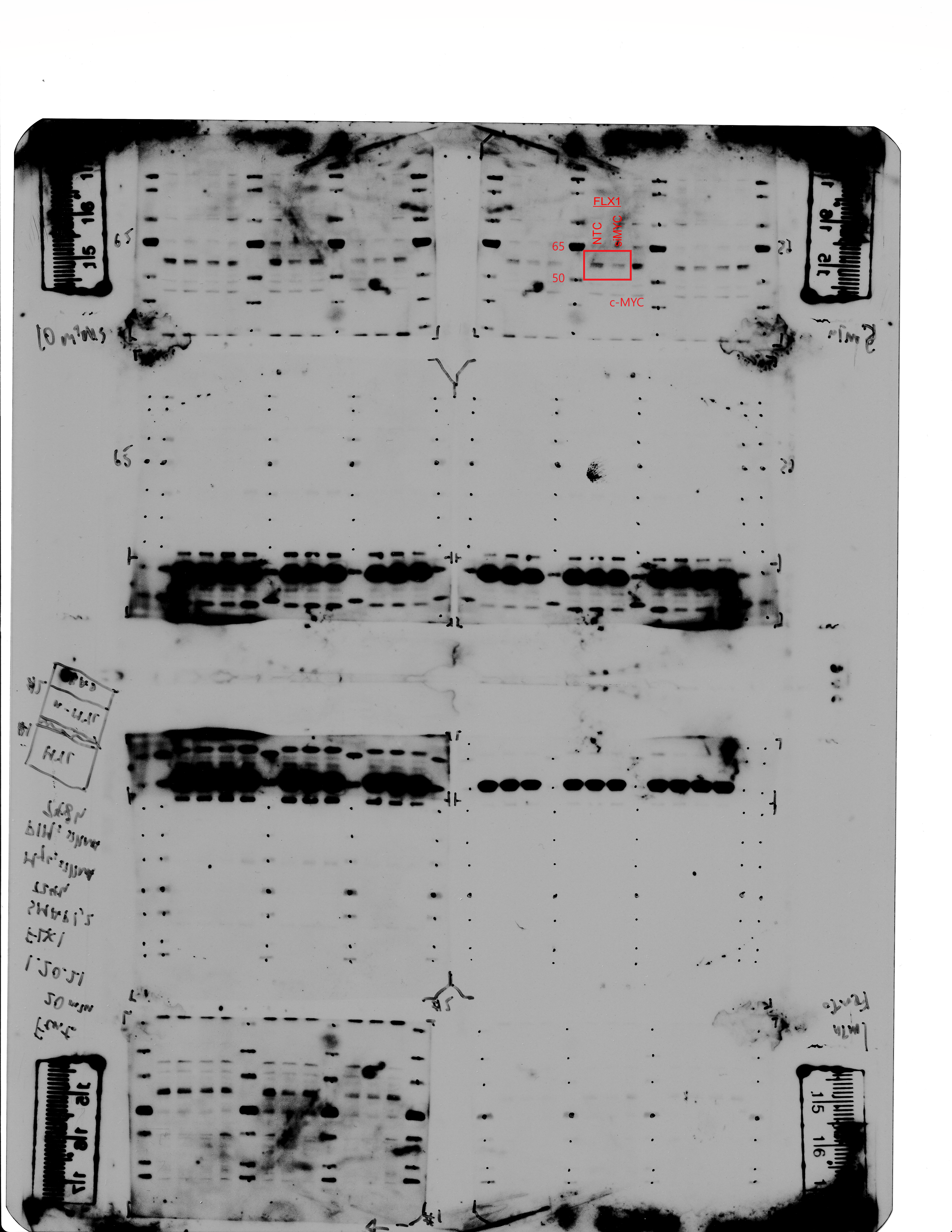

Supplement: Figure 4—source data 6. [file elife-69521-fig4-data6.zip › Figure 4E FLX1 c-MYC Labelled.tif]

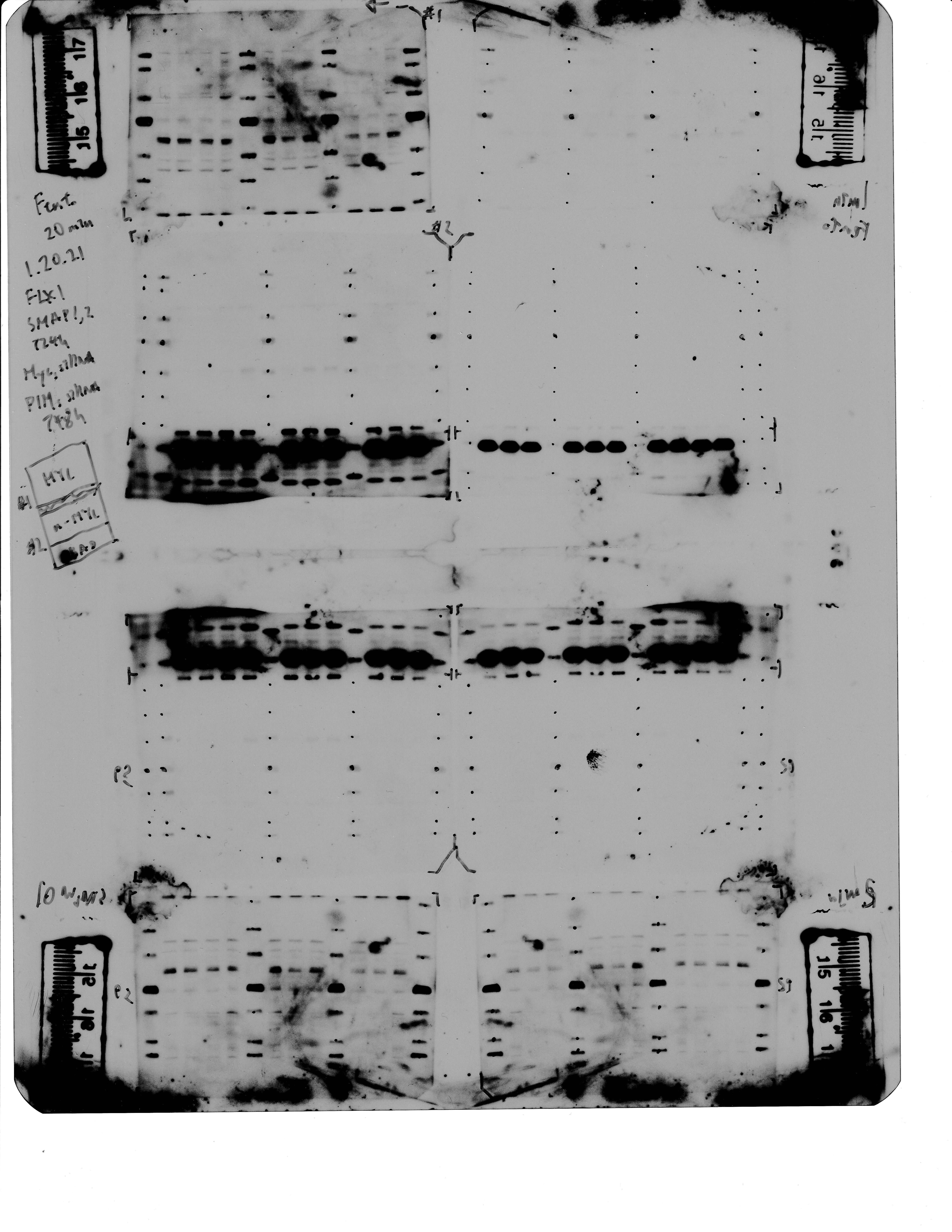

Supplement: Figure 4—source data 6. [file elife-69521-fig4-data6.zip › Figure 4E FLX1 c-MYC Raw.tif]

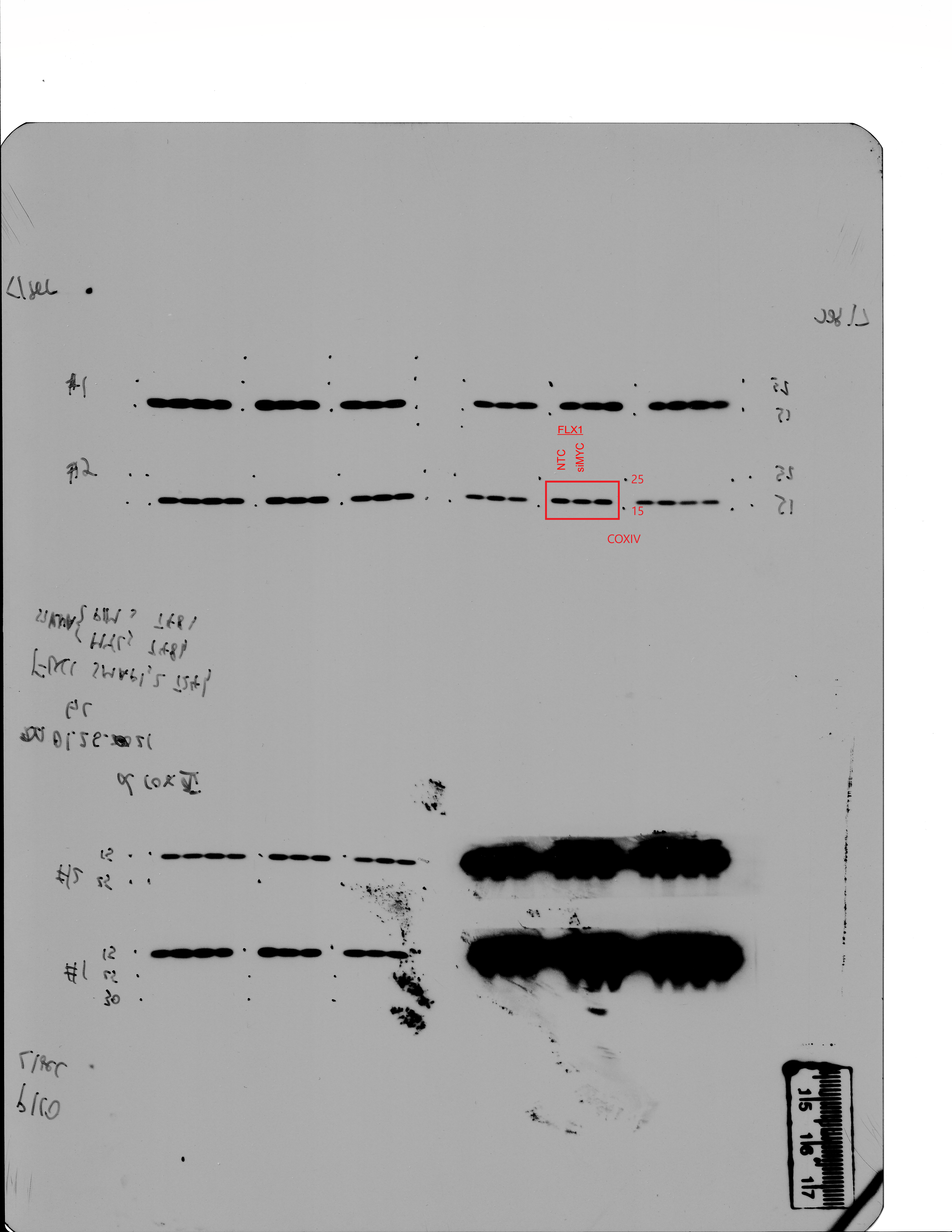

Supplement: Figure 4—source data 6. [file elife-69521-fig4-data6.zip › Figure 4E FLX1 COXIV Labelled.tif]

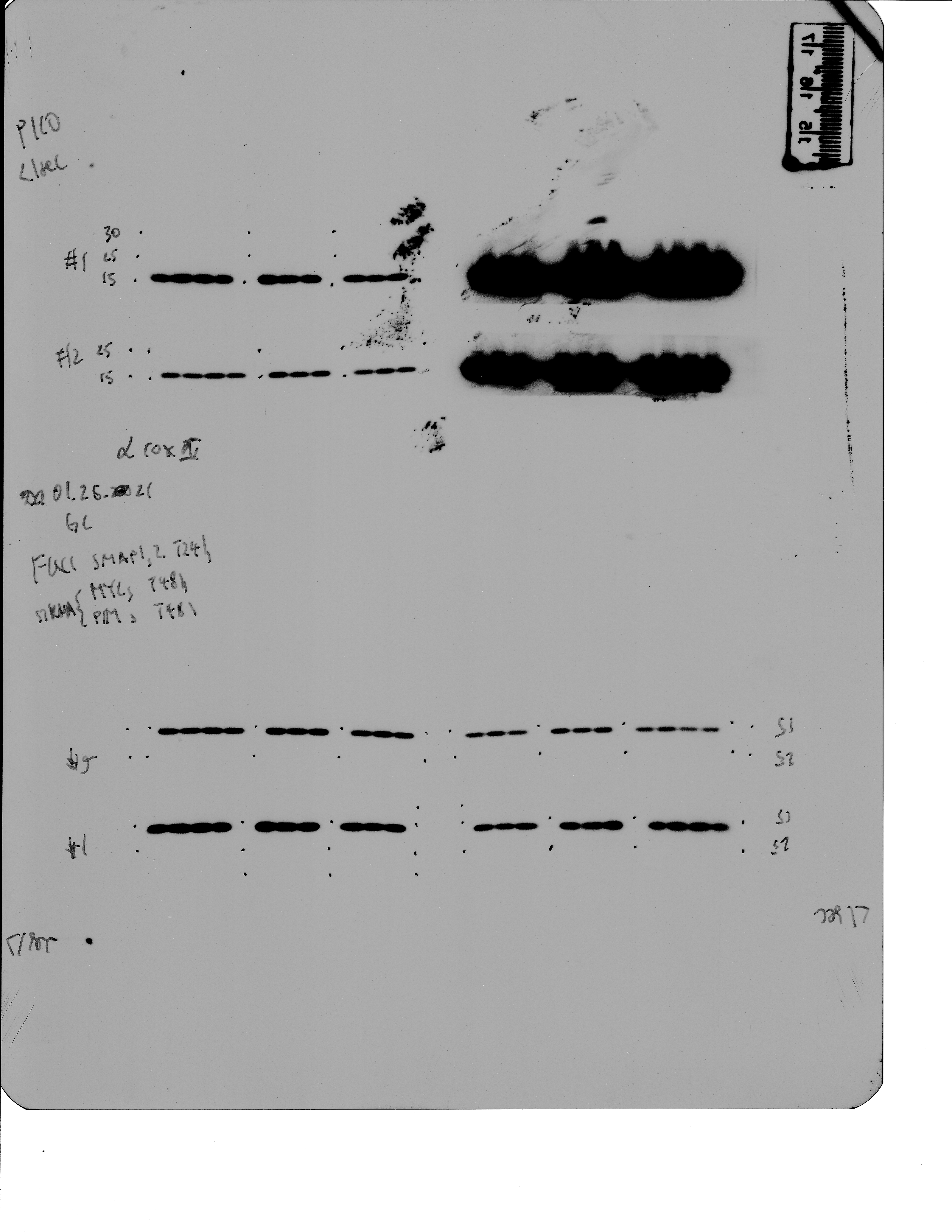

Supplement: Figure 4—source data 6. [file elife-69521-fig4-data6.zip › Figure 4E FLX1 COXIV Raw.tif]

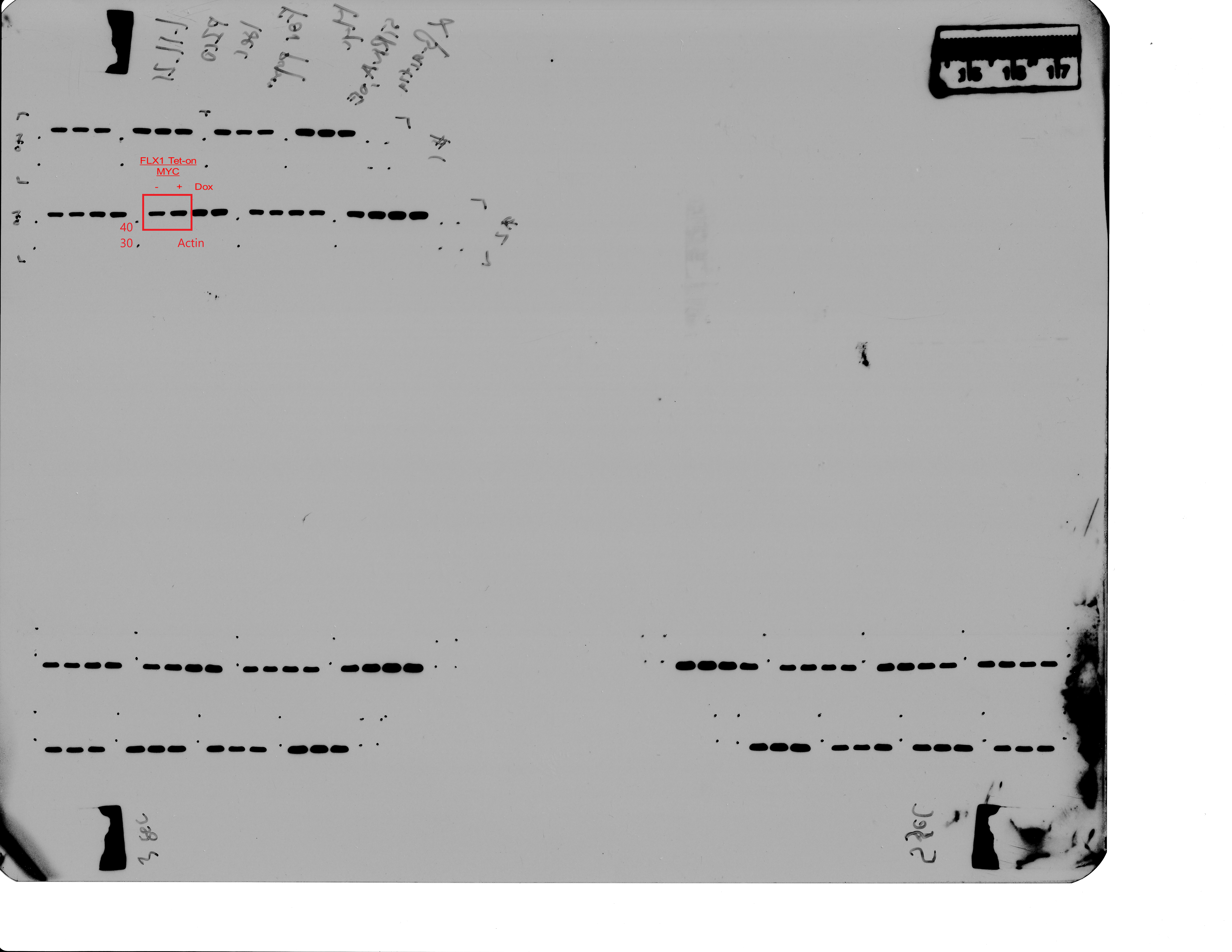

Supplement: Figure 4—source data 9. [file elife-69521-fig4-data9.zip › Figure 4G FLX1 actin Labelled.tif]

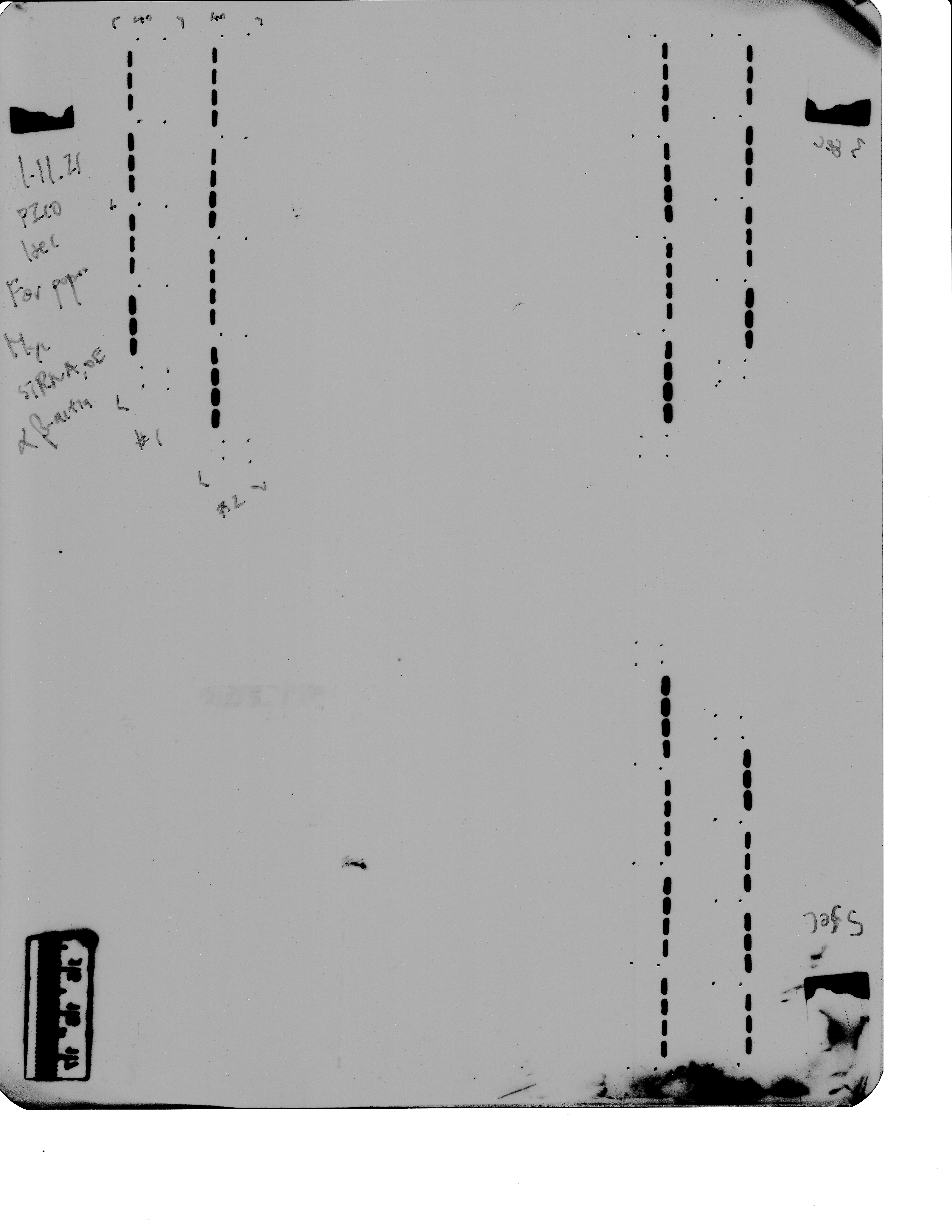

Supplement: Figure 4—source data 9. [file elife-69521-fig4-data9.zip › Figure 4G FLX1 actin Raw.tif]

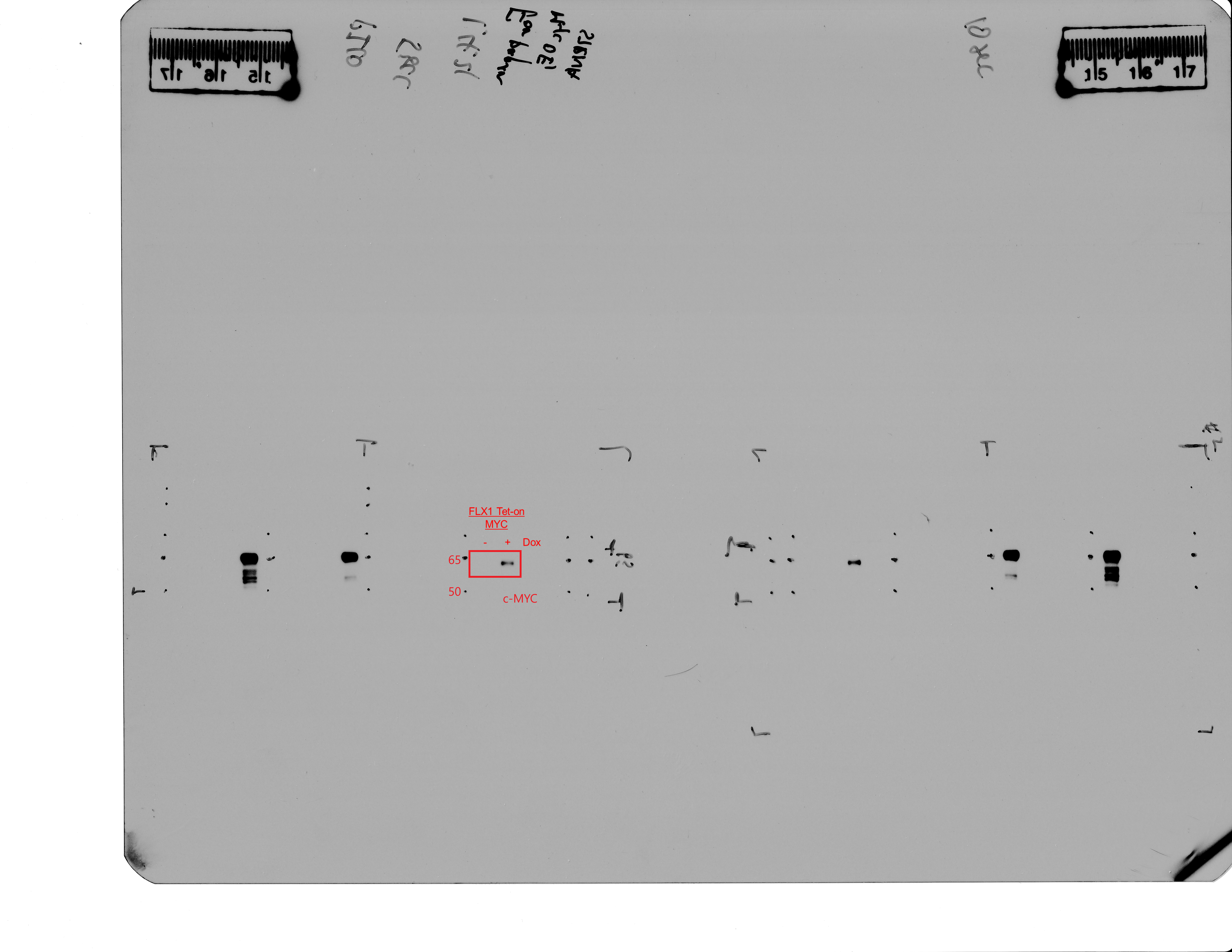

Supplement: Figure 4—source data 9. [file elife-69521-fig4-data9.zip › Figure 4G FLX1 c-MYC Labelled.tif]

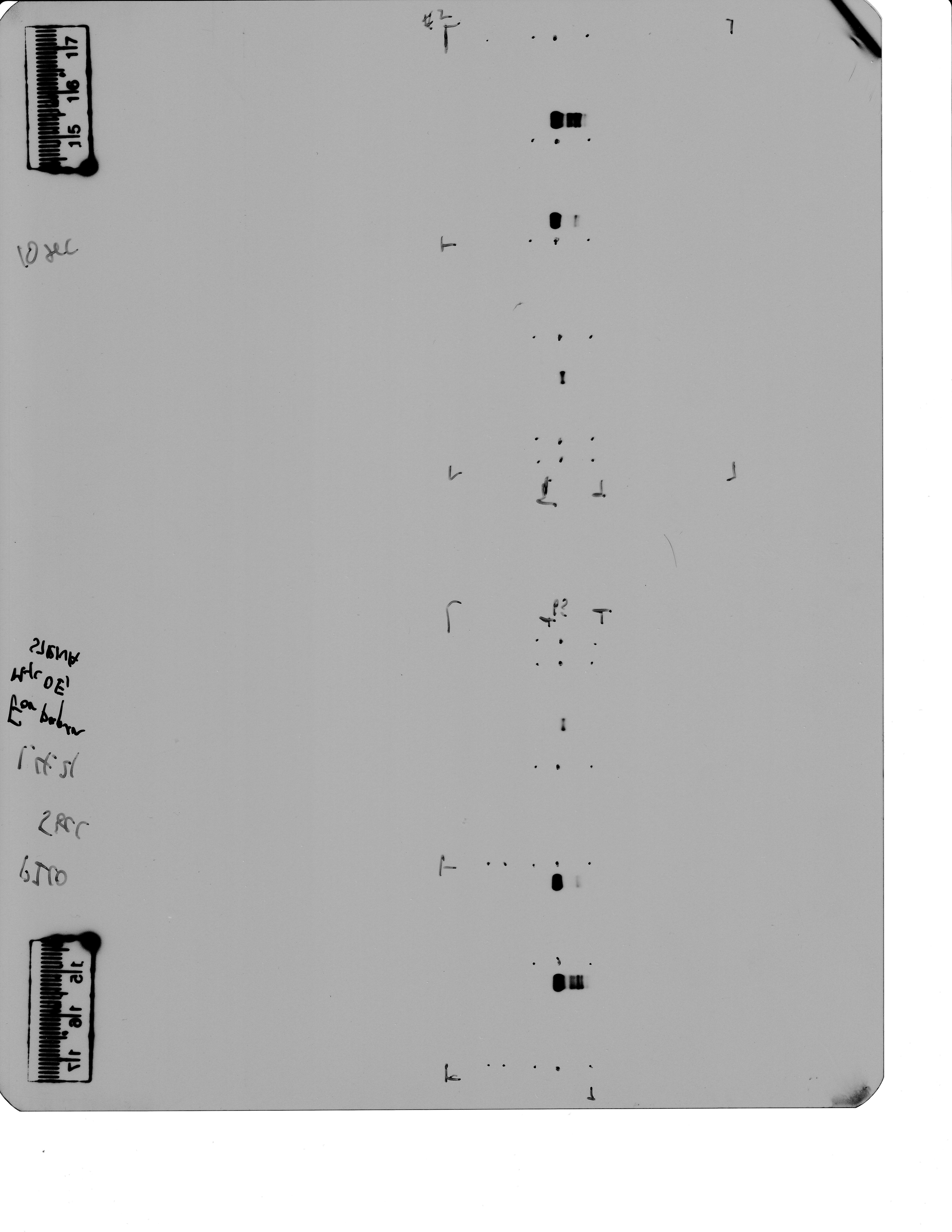

Supplement: Figure 4—source data 9. [file elife-69521-fig4-data9.zip › Figure 4G FLX1 c-MYC Raw.tif]

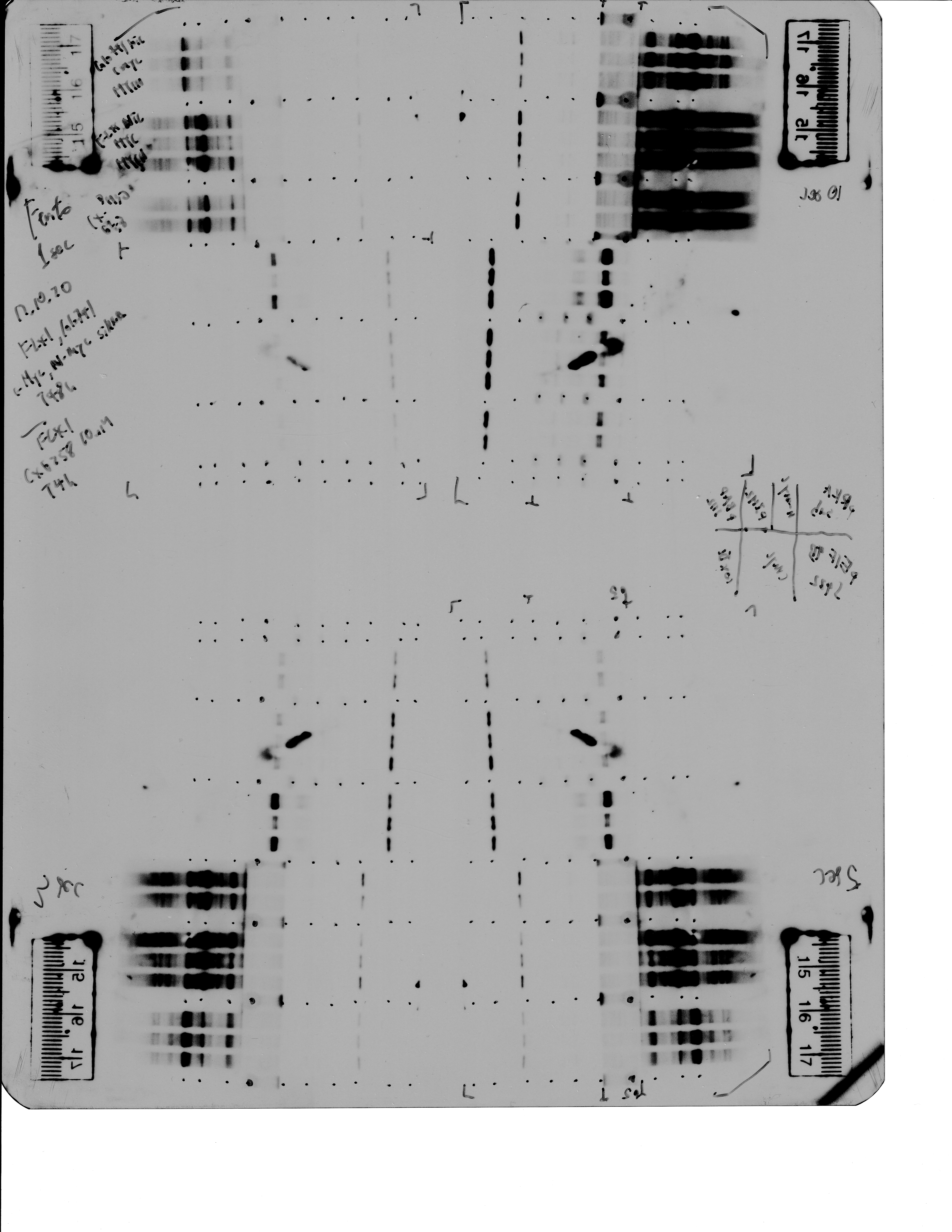

Supplement: Figure 4—figure supplement 1—source data 3. [file elife-69521-fig4-figsupp1-data3.zip › Figure 3D Colo741 c-MYC, COXIV Raw.tif]

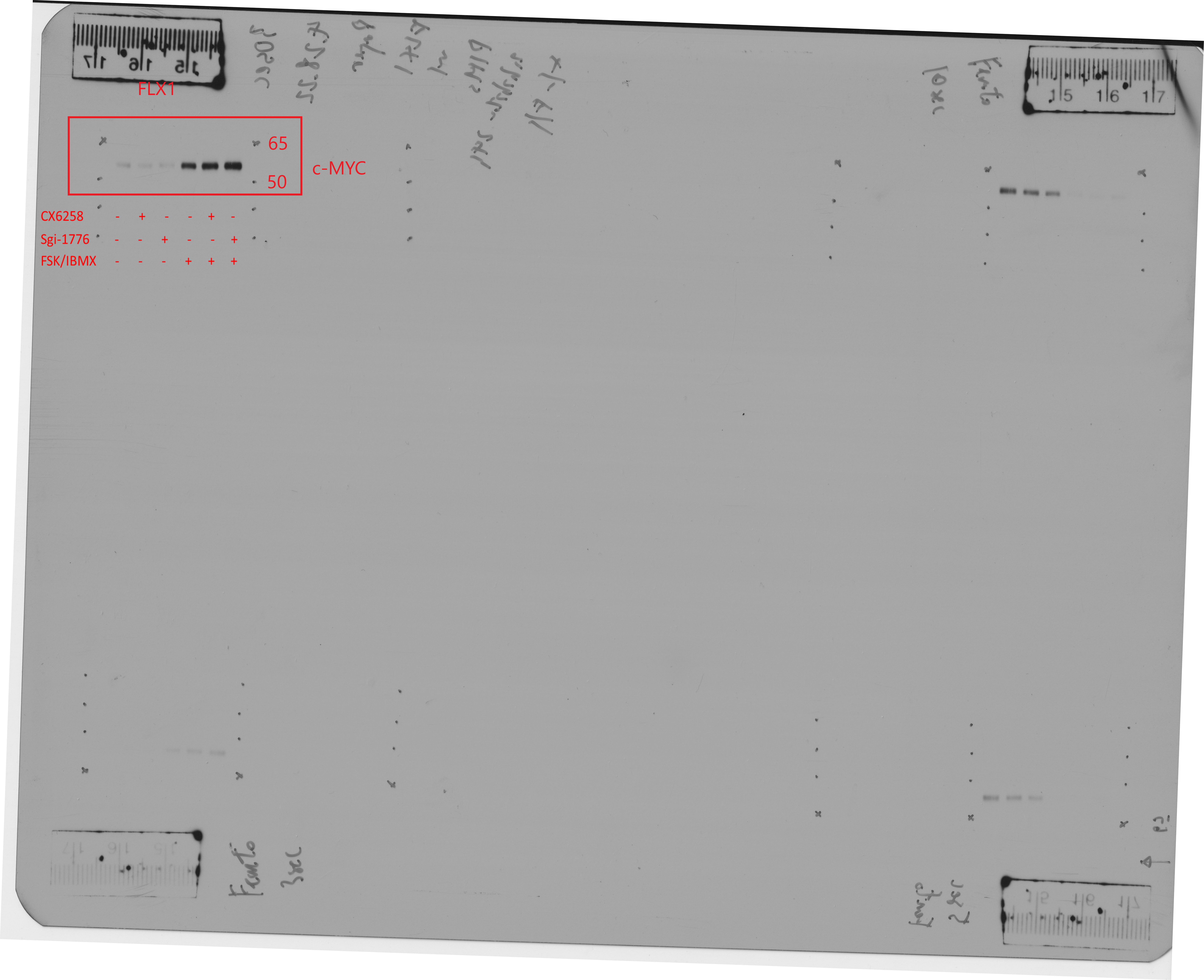

Supplement: Figure 5—source data 6. [file elife-69521-fig5-data6.zip › 5F/Figure 5F FLX1 c-MYC Labelled.tiff]

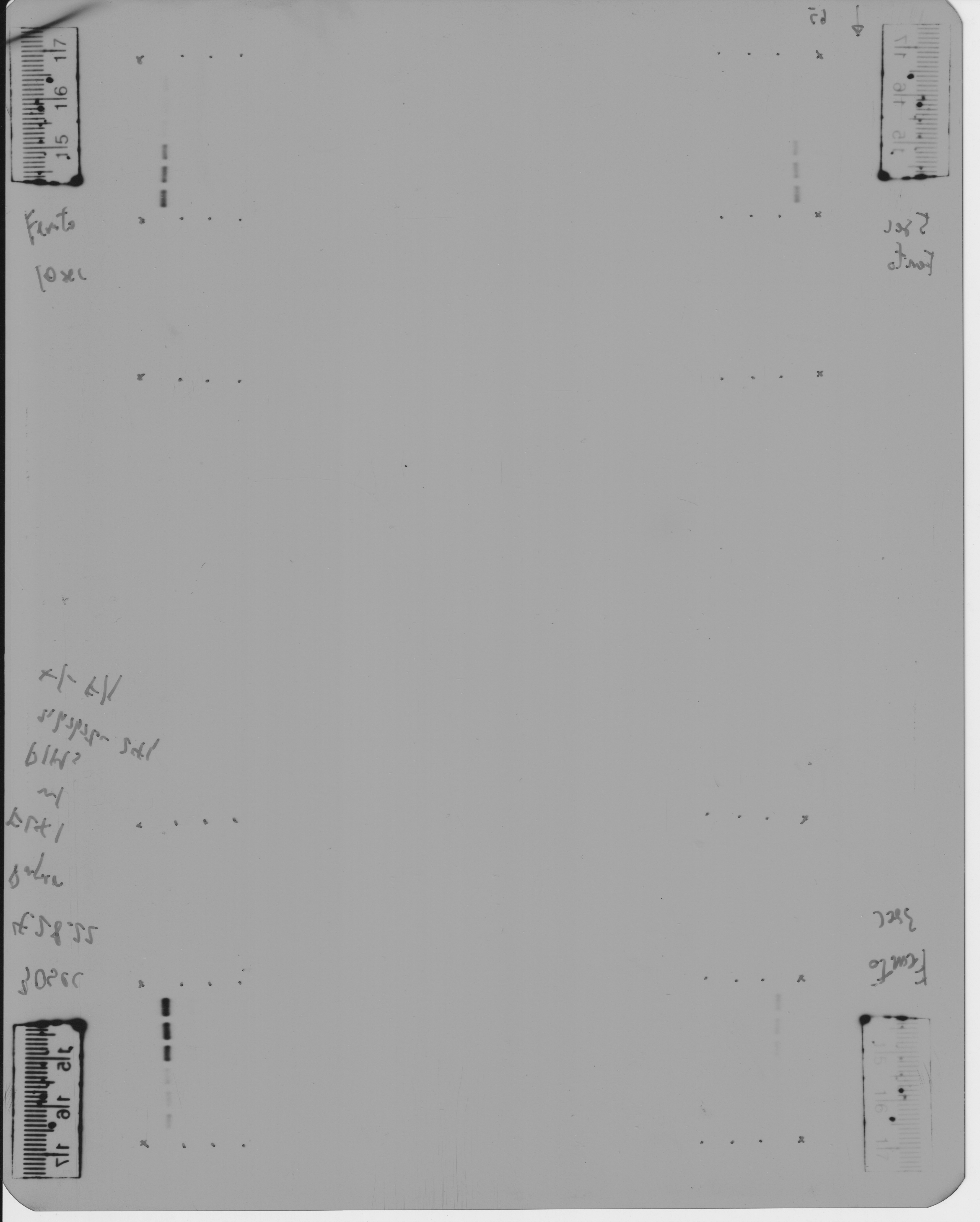

Supplement: Figure 5—source data 6. [file elife-69521-fig5-data6.zip › 5F/Figure 5F FLX1 c-MYC Raw.tiff]

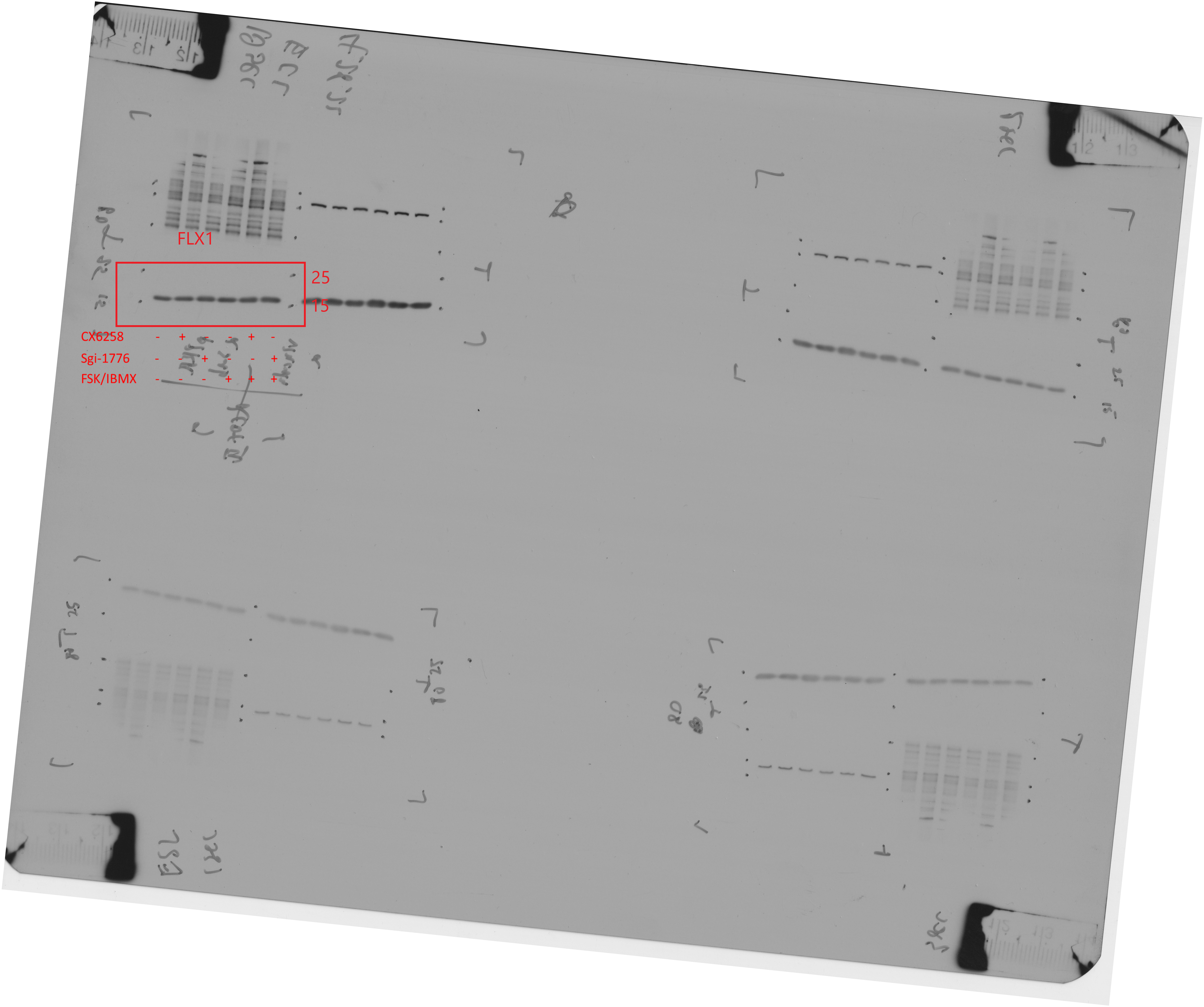

Supplement: Figure 5—source data 6. [file elife-69521-fig5-data6.zip › 5F/Figure 5F FLX1 COXIV Labelled.tiff]

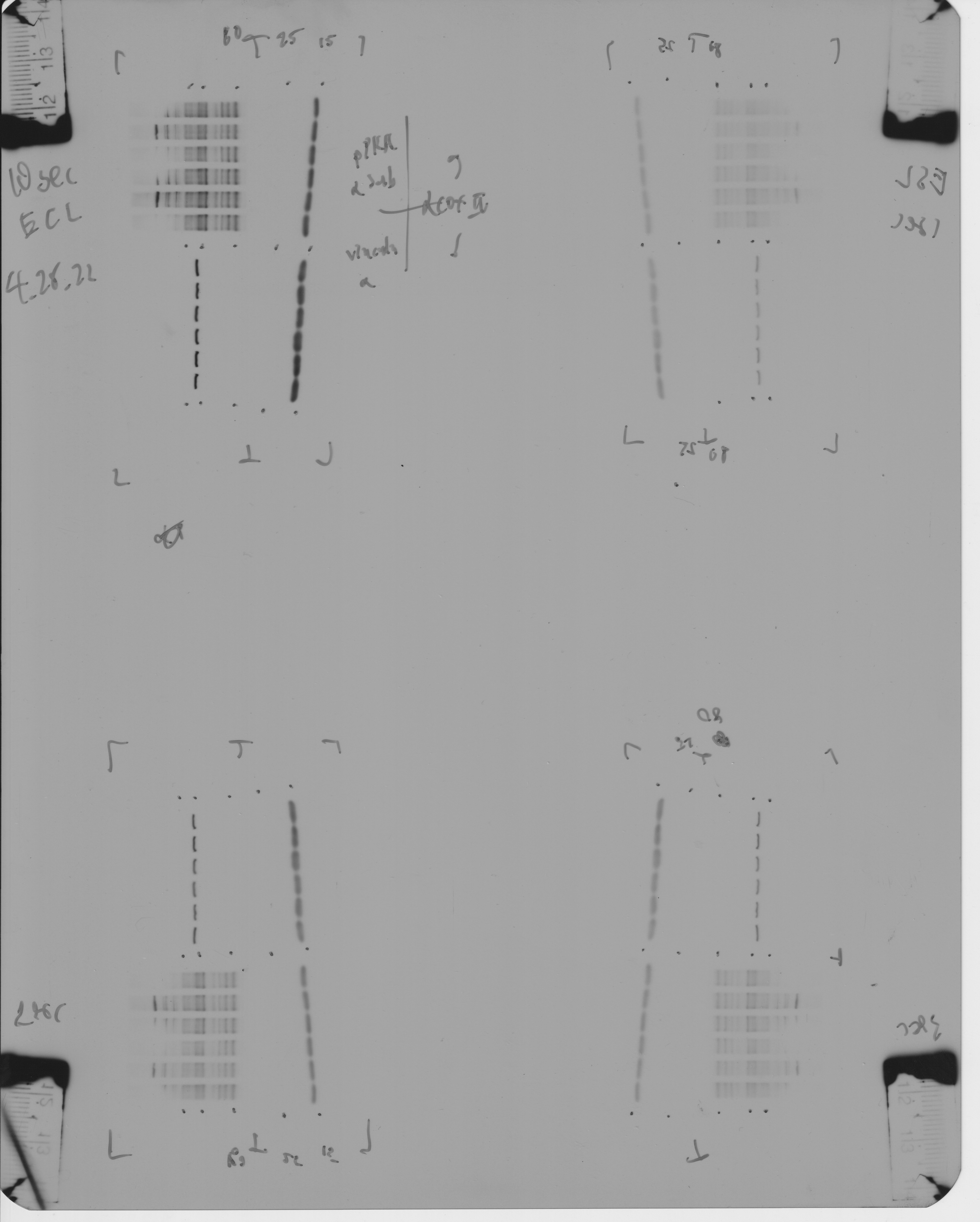

Supplement: Figure 5—source data 6. [file elife-69521-fig5-data6.zip › 5F/Figure 5F FLX1 COXIV Raw.tiff]

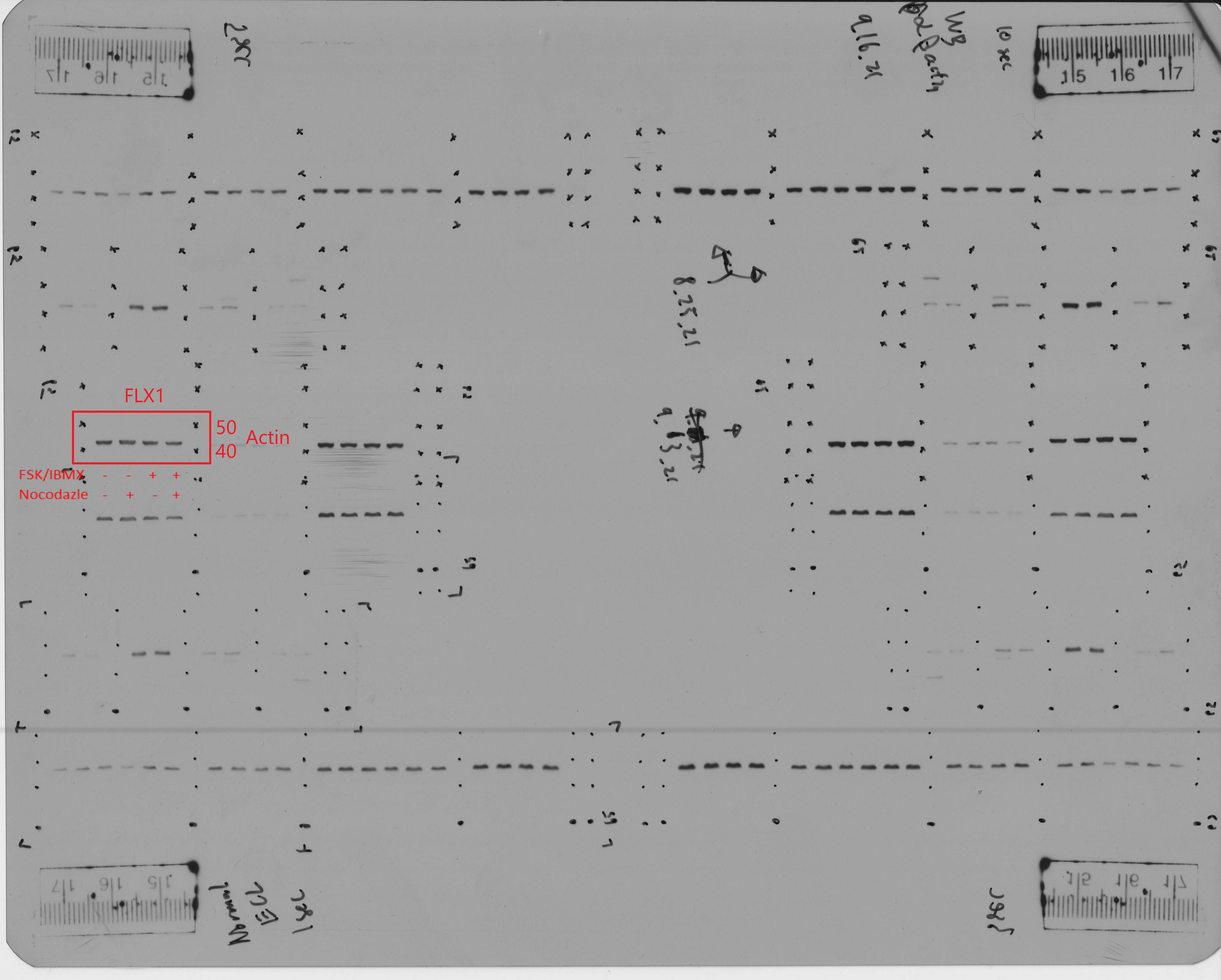

Supplement: Figure 5—figure supplement 1—source data 2. [file elife-69521-fig5-figsupp1-data2.zip › Figure S2B/Figure S2B FLX1 Actin Labelled.tiff]

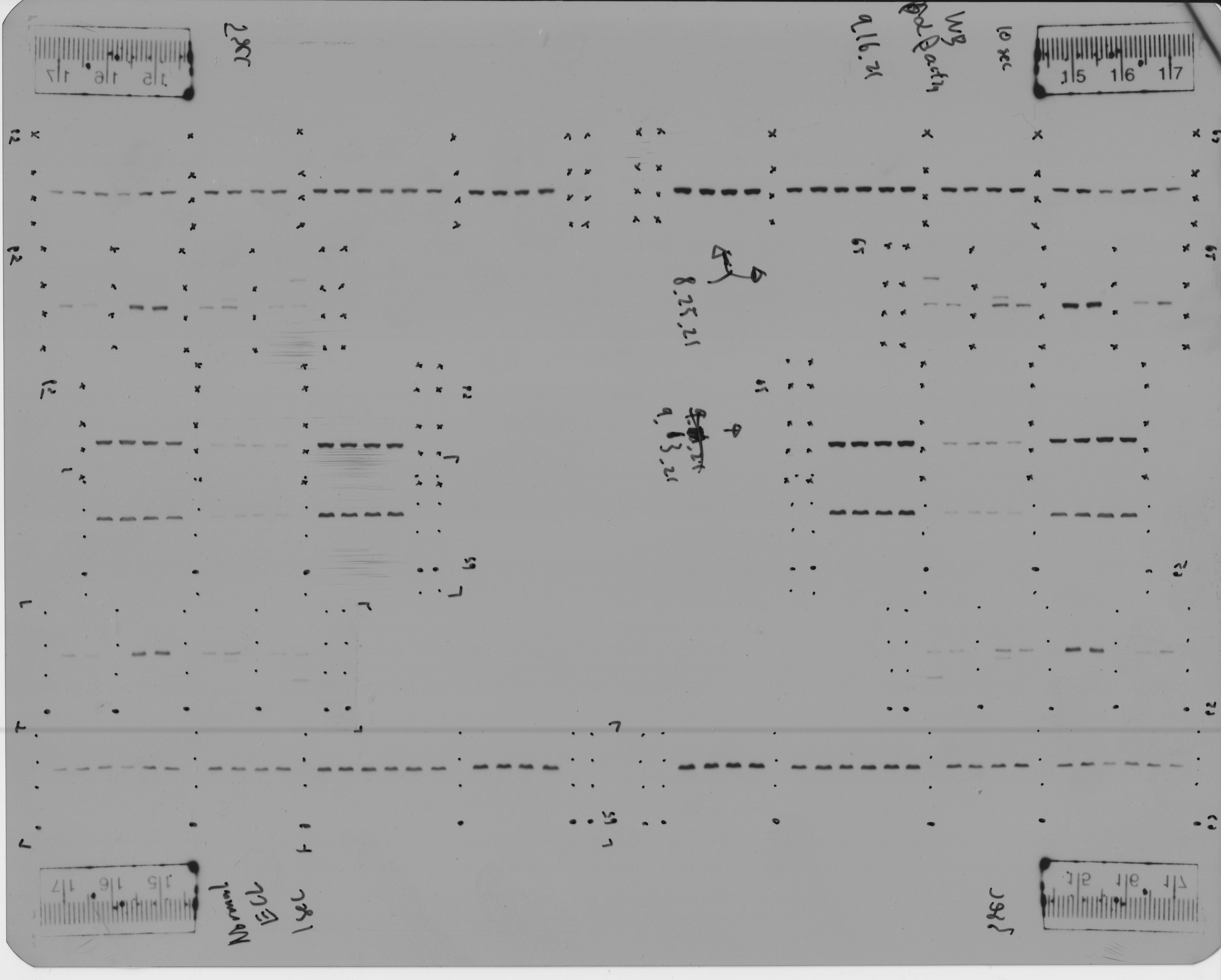

Supplement: Figure 5—figure supplement 1—source data 2. [file elife-69521-fig5-figsupp1-data2.zip › Figure S2B/Figure S2B FLX1 Actin Raw.tiff]

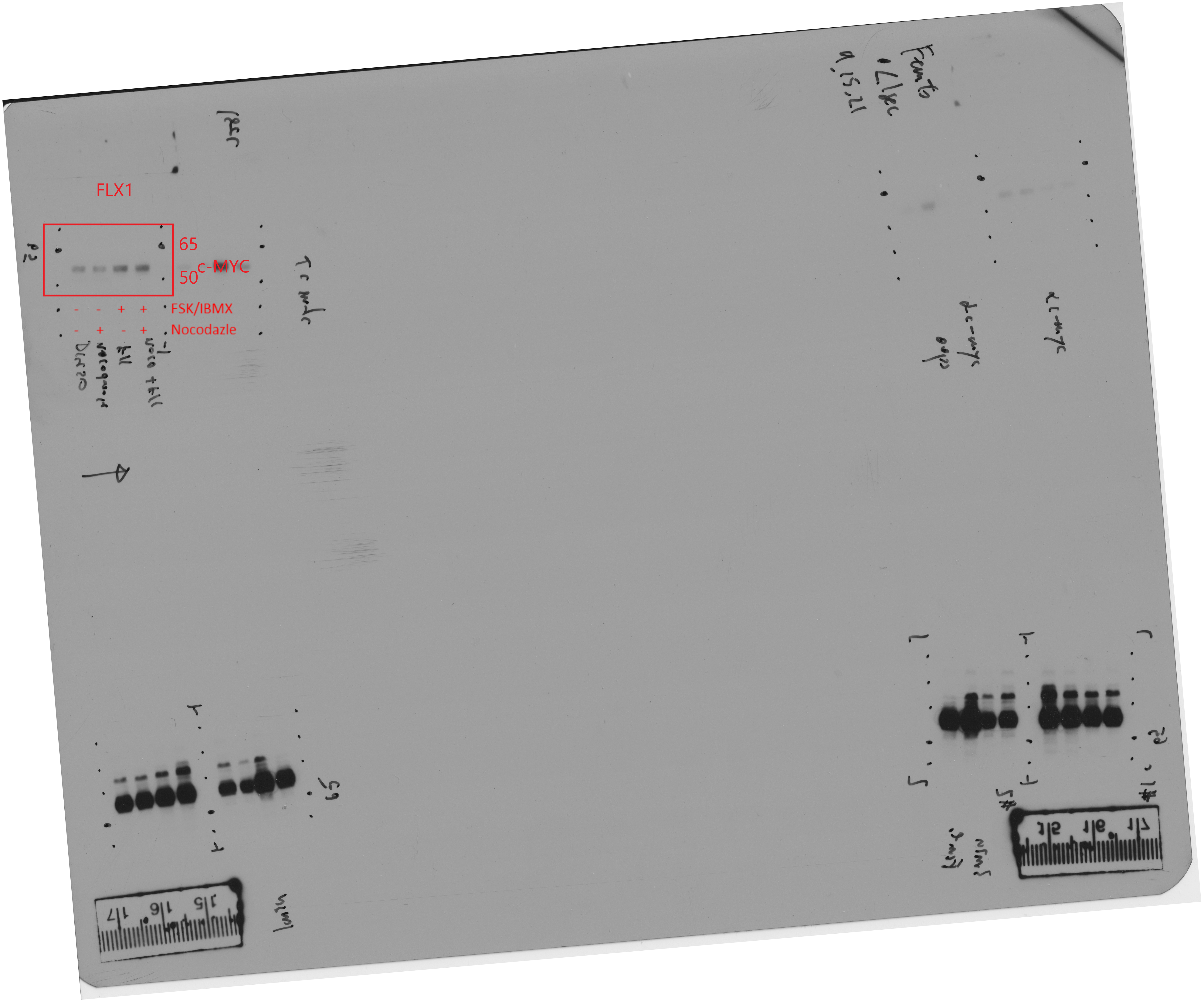

Supplement: Figure 5—figure supplement 1—source data 2. [file elife-69521-fig5-figsupp1-data2.zip › Figure S2B/Figure S2B FLX1 c-MYC Labelled.tiff]

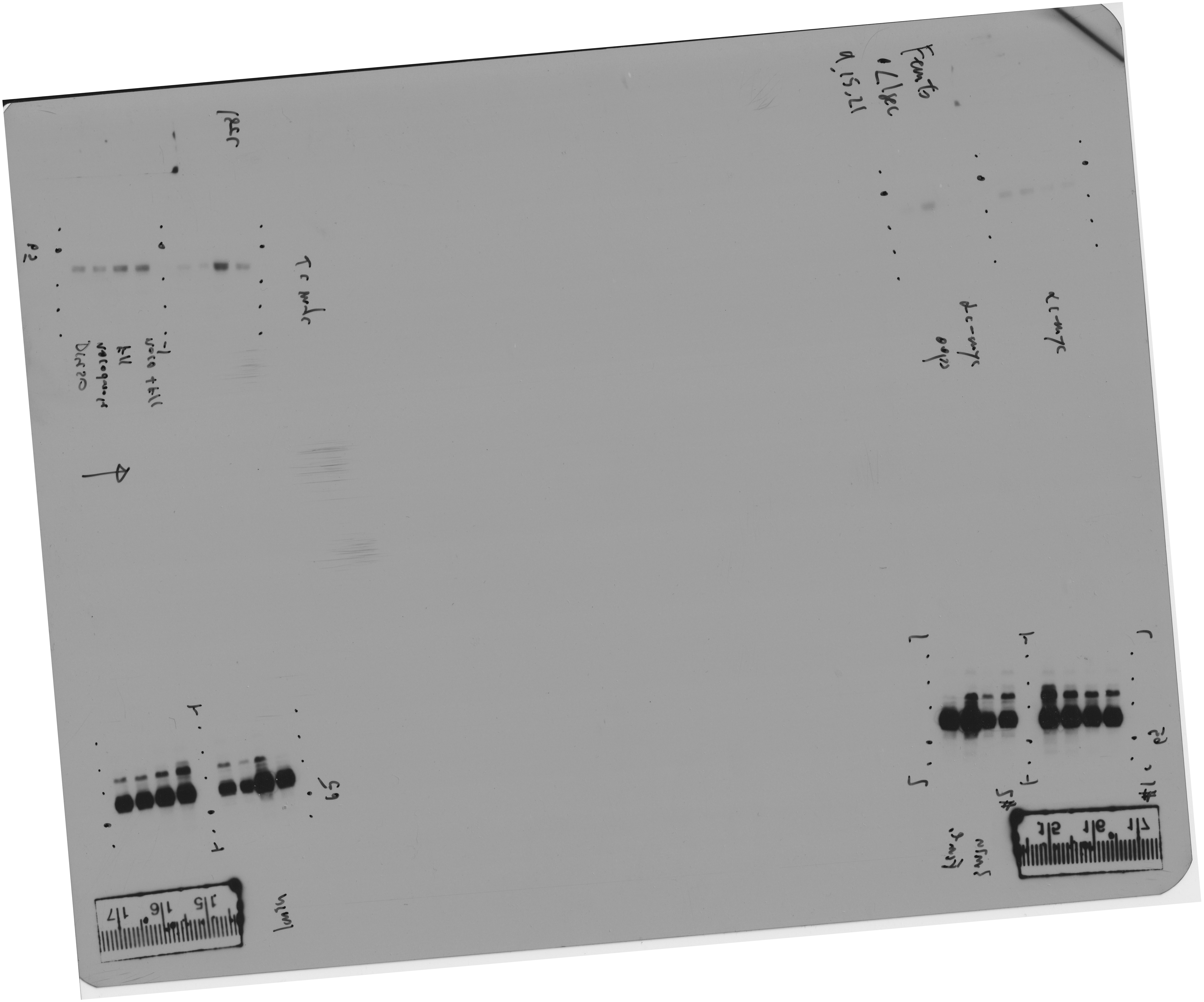

Supplement: Figure 5—figure supplement 1—source data 2. [file elife-69521-fig5-figsupp1-data2.zip › Figure S2B/Figure S2B FLX1 c-MYC Raw.tiff]

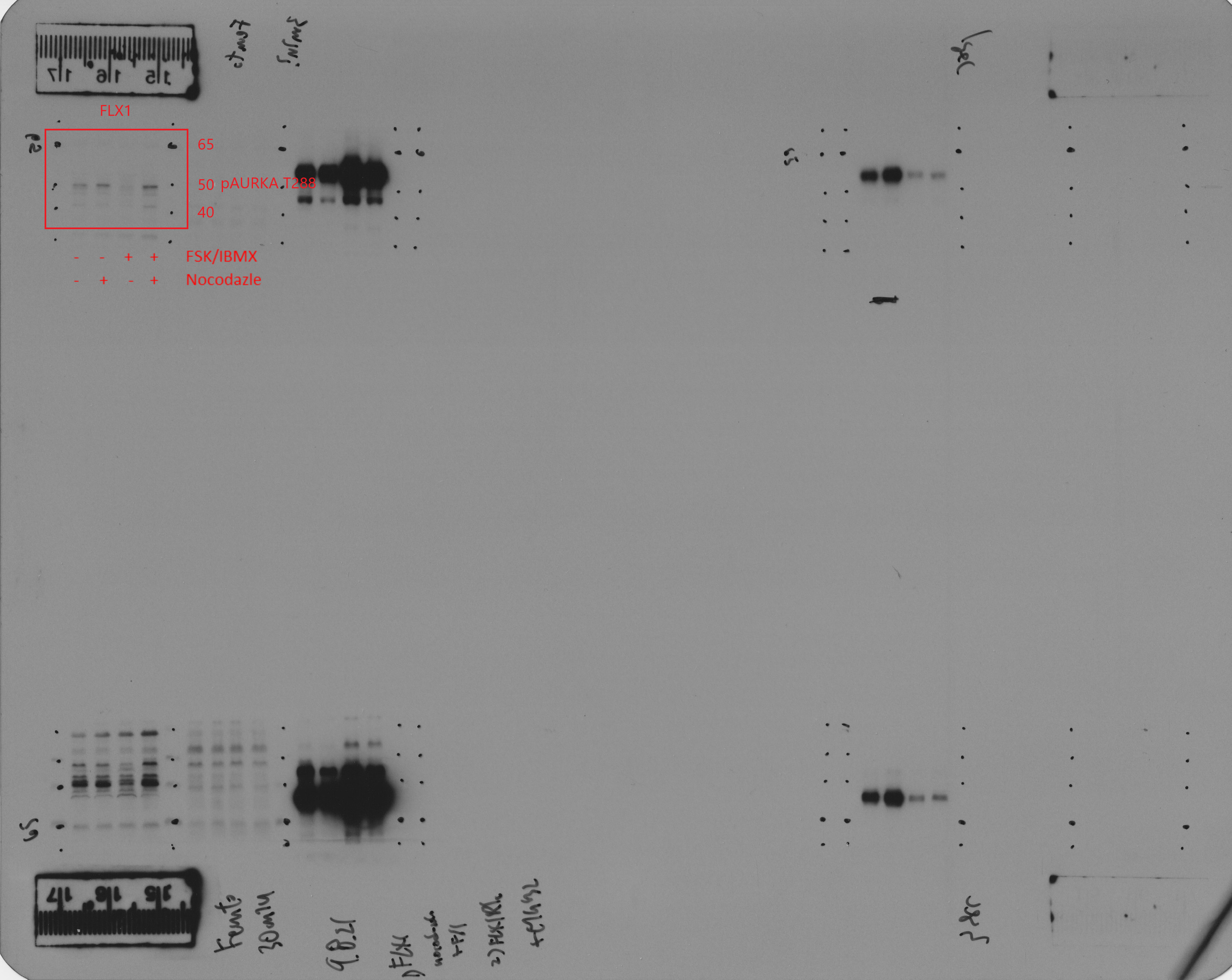

Supplement: Figure 5—figure supplement 1—source data 2. [file elife-69521-fig5-figsupp1-data2.zip › Figure S2B/Figure S2B FLX1 pAURKAT288 Labelled.tiff]

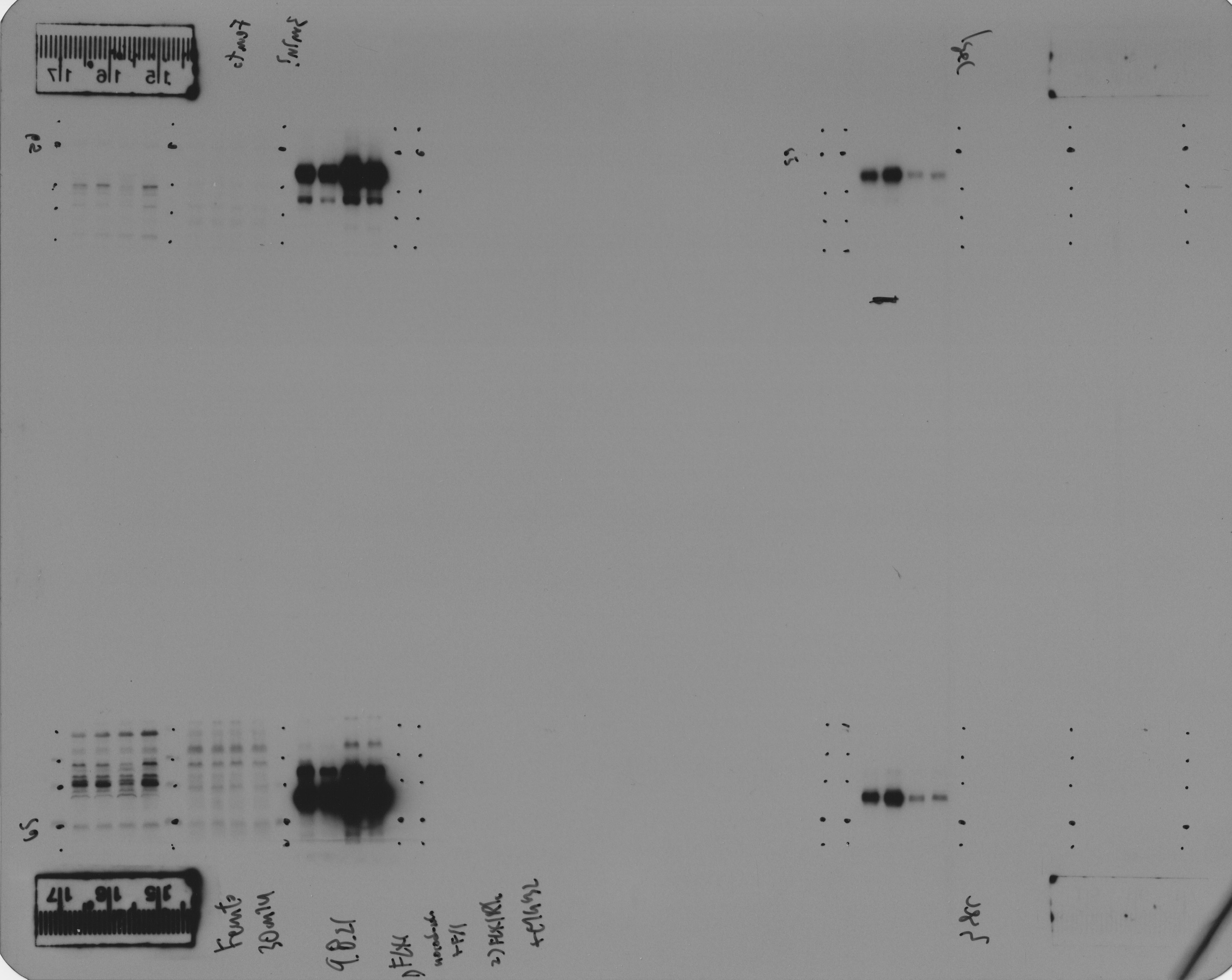

Supplement: Figure 5—figure supplement 1—source data 2. [file elife-69521-fig5-figsupp1-data2.zip › Figure S2B/Figure S2B FLX1 pAURKAT288 Raw.tiff]

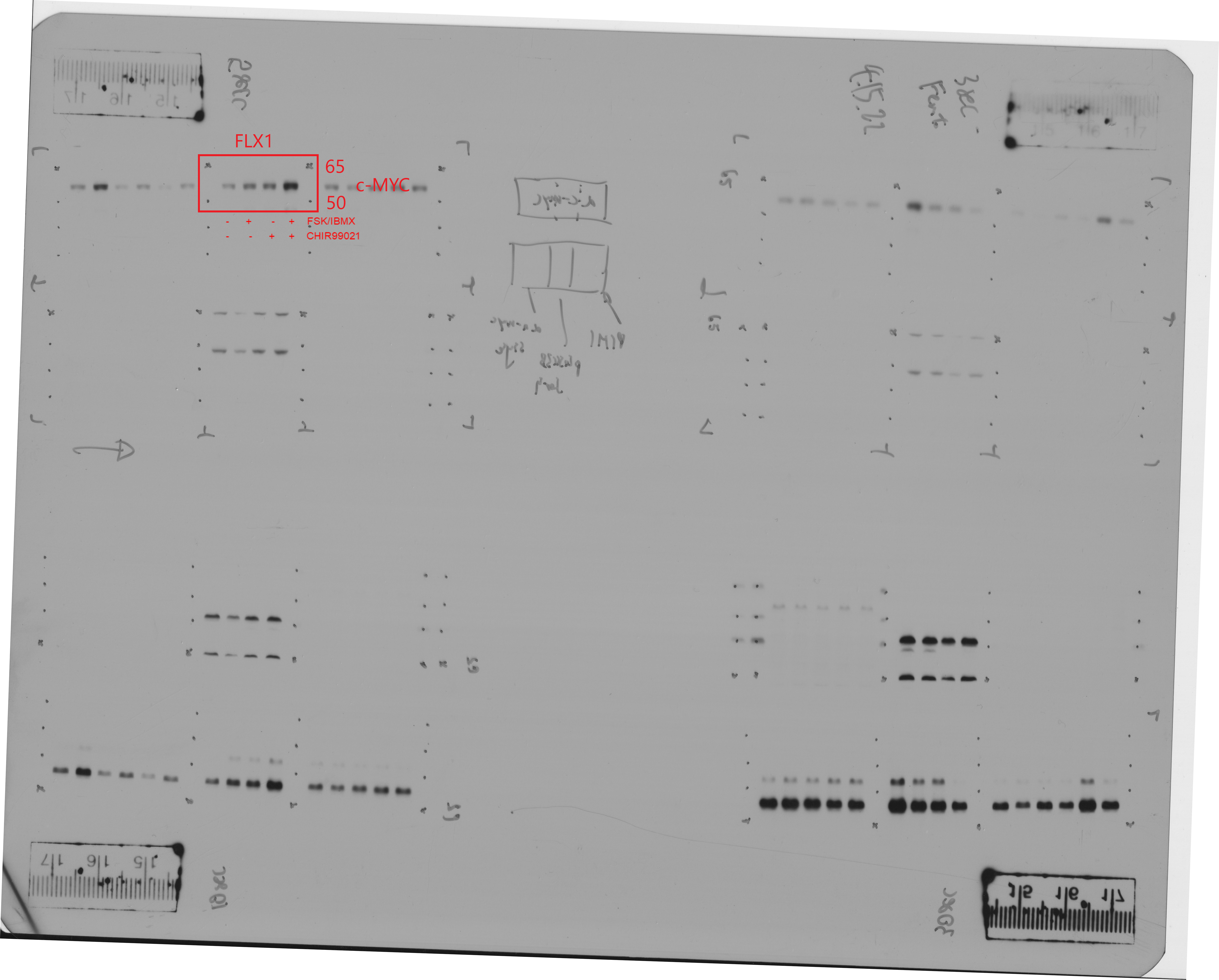

Supplement: Figure 5—figure supplement 1—source data 3. [file elife-69521-fig5-figsupp1-data3.zip › Figure S2C/Figure S2C FLX1 c-MYC Labelled.tiff]

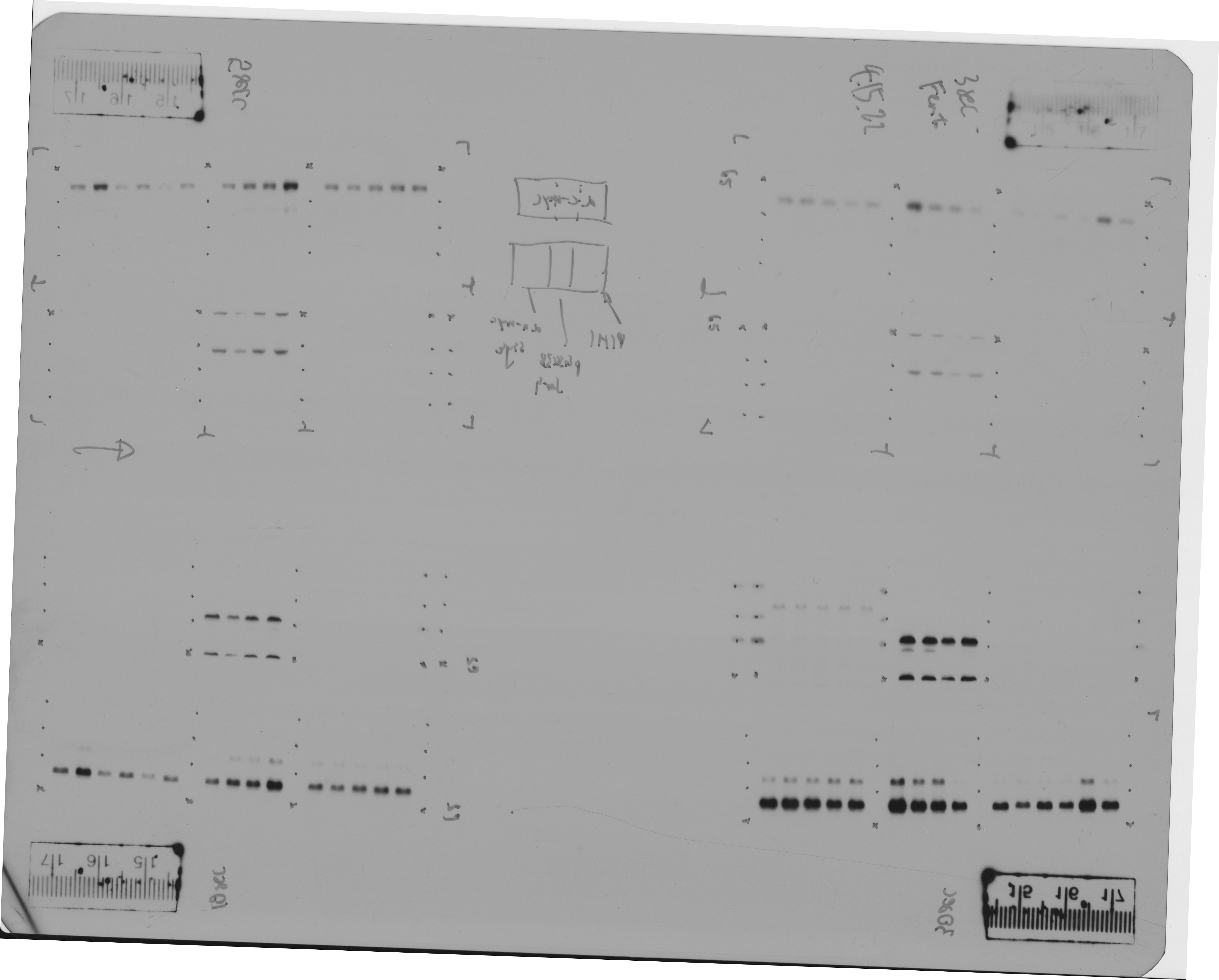

Supplement: Figure 5—figure supplement 1—source data 3. [file elife-69521-fig5-figsupp1-data3.zip › Figure S2C/Figure S2C FLX1 c-MYC Raw.tiff]

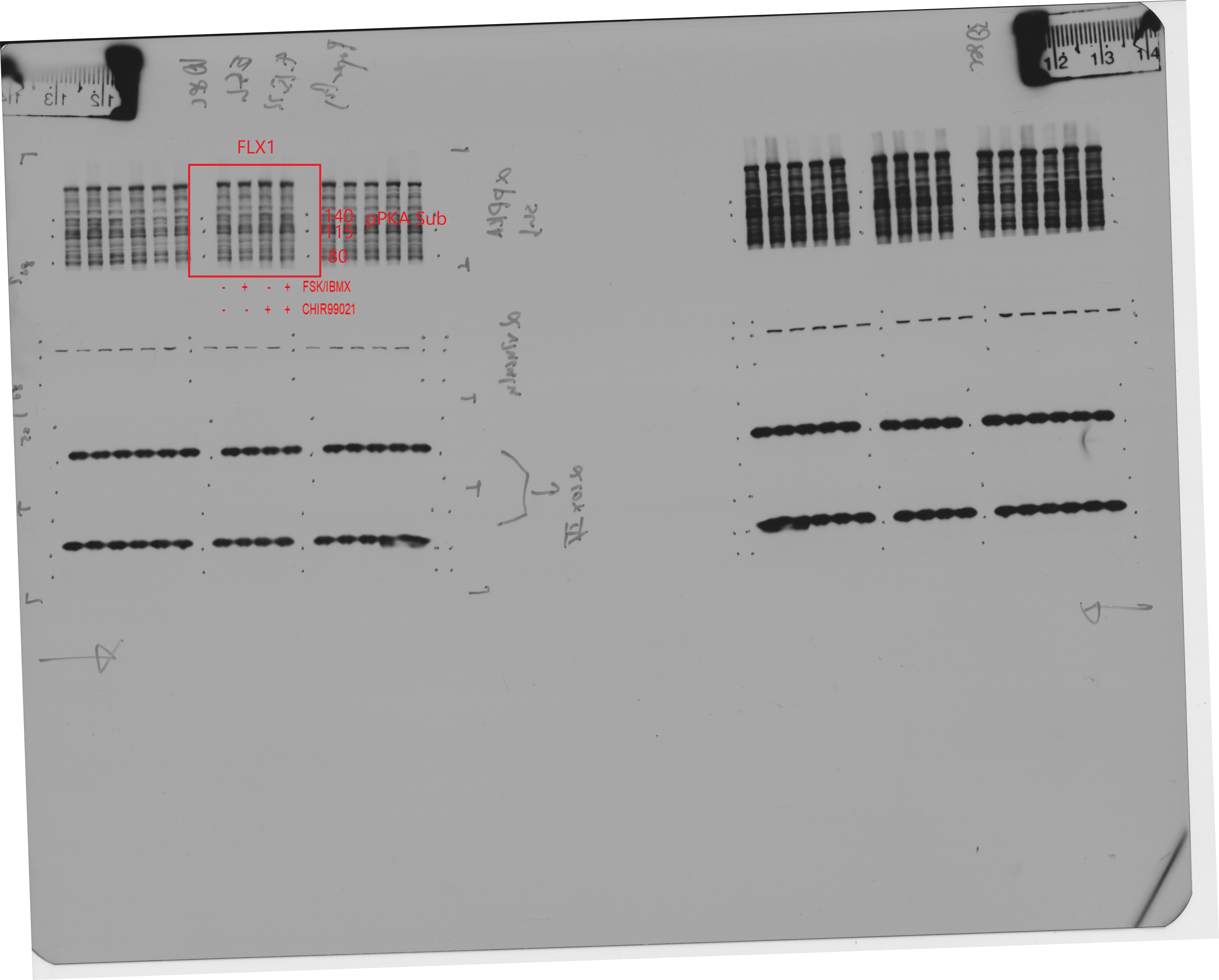

Supplement: Figure 5—figure supplement 1—source data 3. [file elife-69521-fig5-figsupp1-data3.zip › Figure S2C/Figure S2C FLX1 pPKA-sub Labelled.tif]

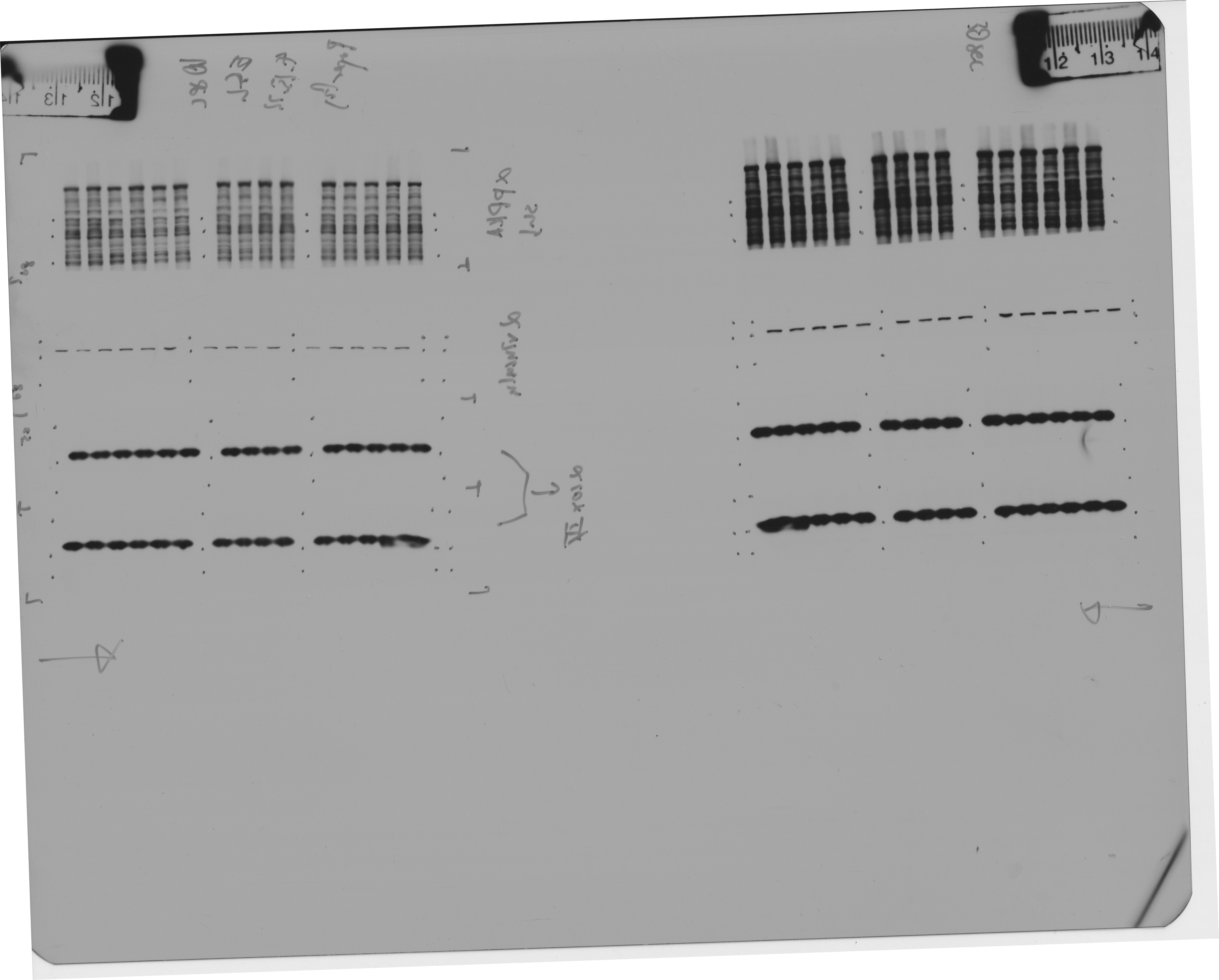

Supplement: Figure 5—figure supplement 1—source data 3. [file elife-69521-fig5-figsupp1-data3.zip › Figure S2C/Figure S2C FLX1 pPKA-sub Raw.tif]

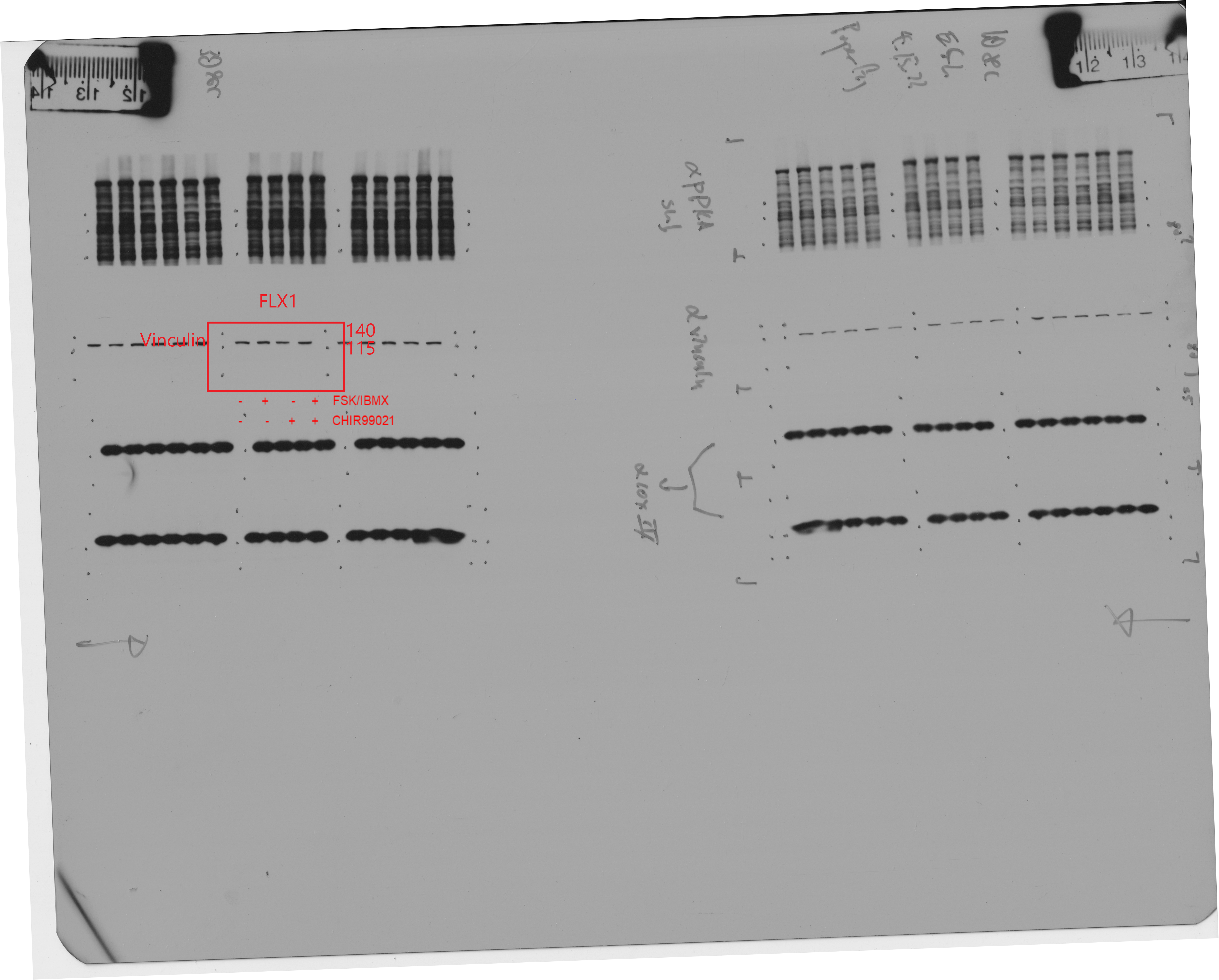

Supplement: Figure 5—figure supplement 1—source data 3. [file elife-69521-fig5-figsupp1-data3.zip › Figure S2C/Figure S2C FLX1 vinculin Labelled.tiff]

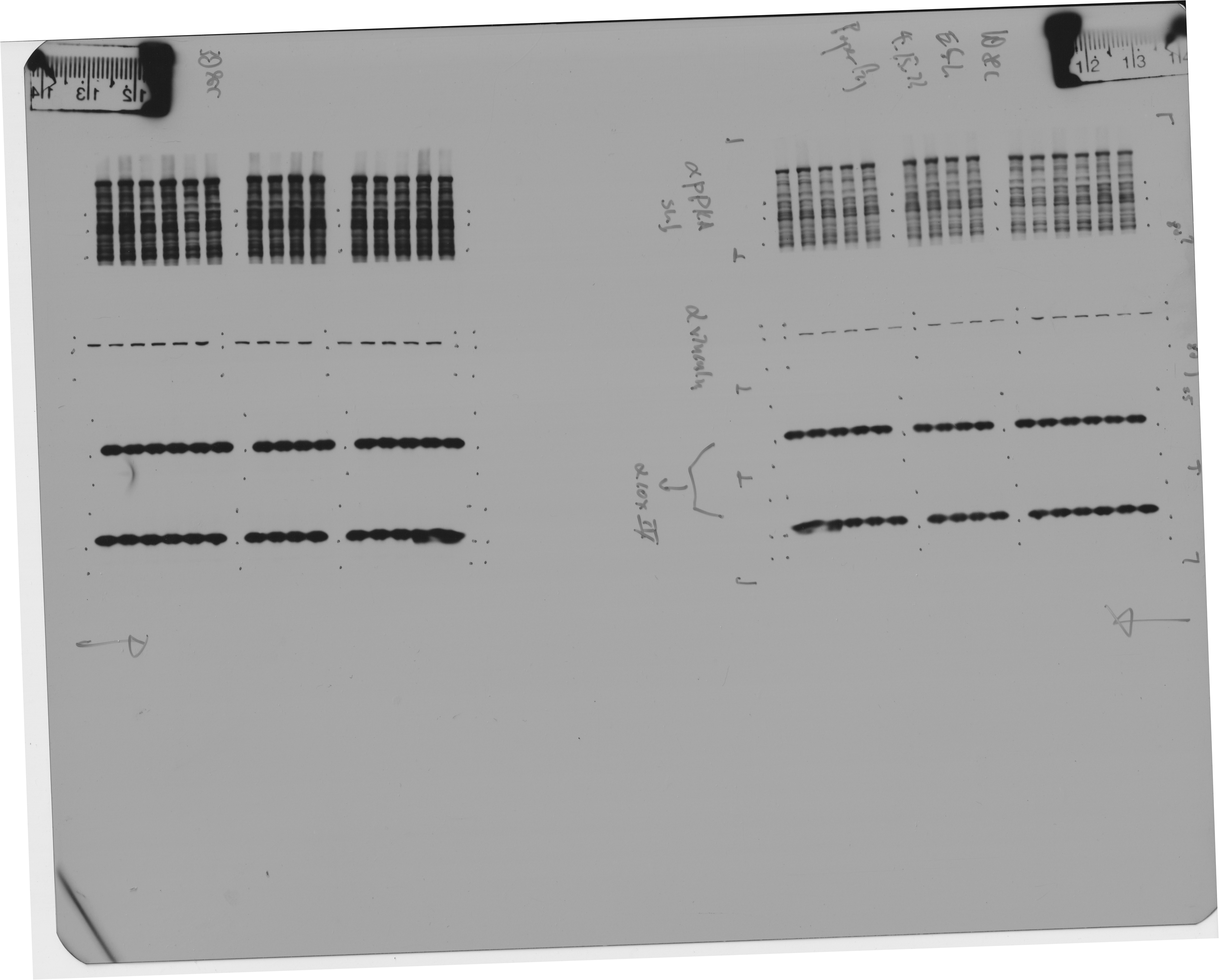

Supplement: Figure 5—figure supplement 1—source data 3. [file elife-69521-fig5-figsupp1-data3.zip › Figure S2C/Figure S2C FLX1 vinculin Raw.tiff]

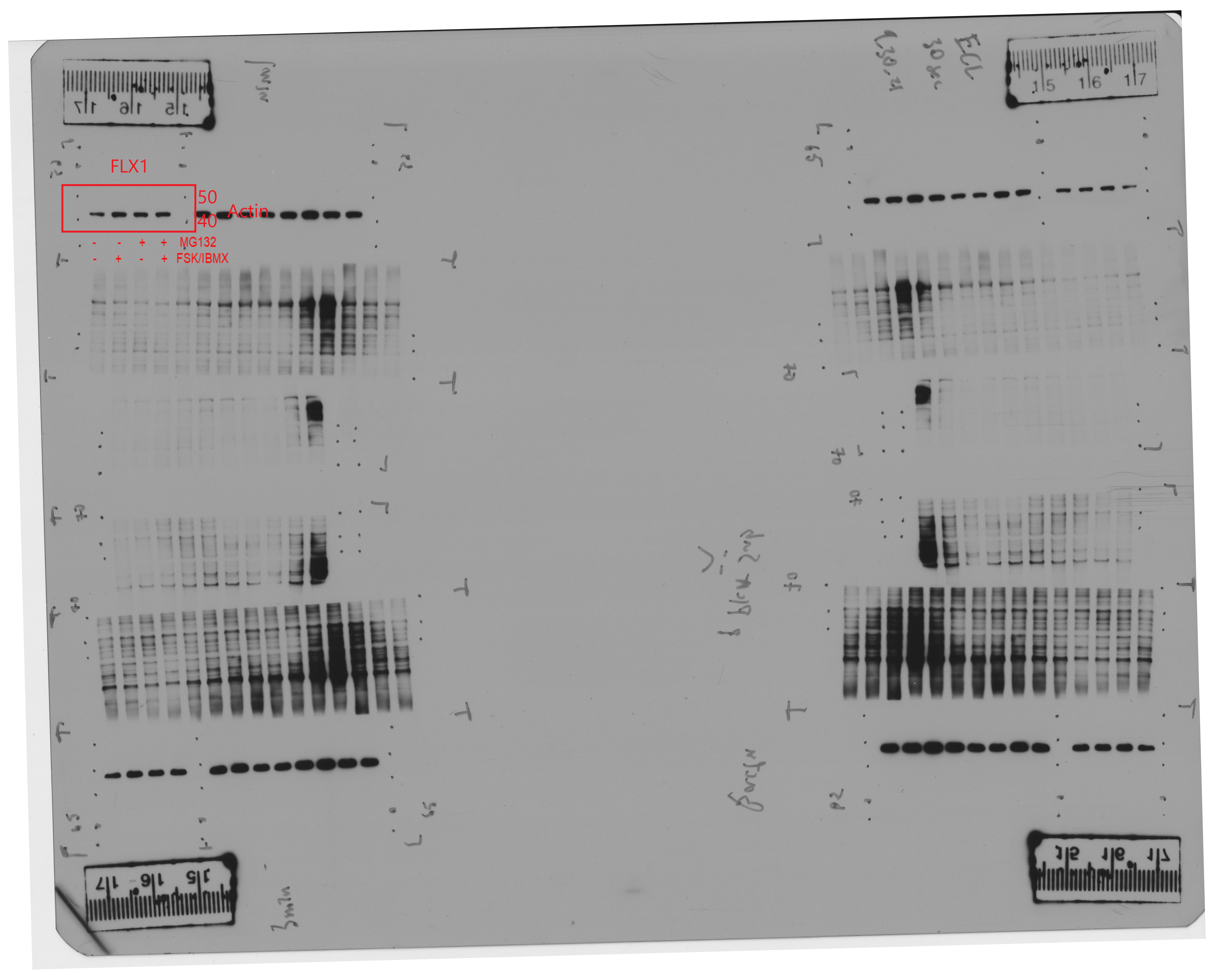

Supplement: Figure 5—figure supplement 2—source data 1. [file elife-69521-fig5-figsupp2-data1.zip › Figure S3A/Figure S3A Actin Labelled.tiff]

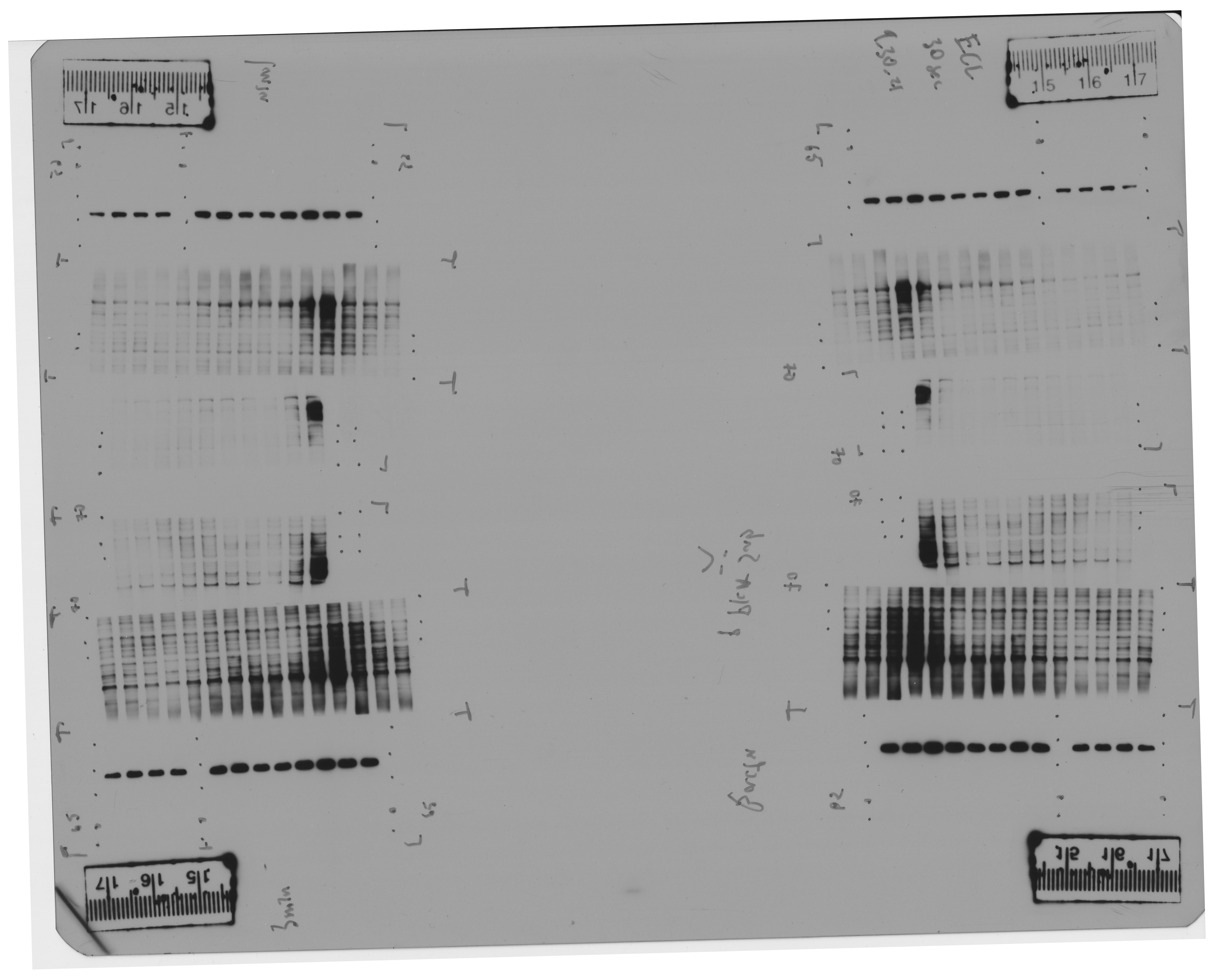

Supplement: Figure 5—figure supplement 2—source data 1. [file elife-69521-fig5-figsupp2-data1.zip › Figure S3A/Figure S3A Actin Raw.tiff]

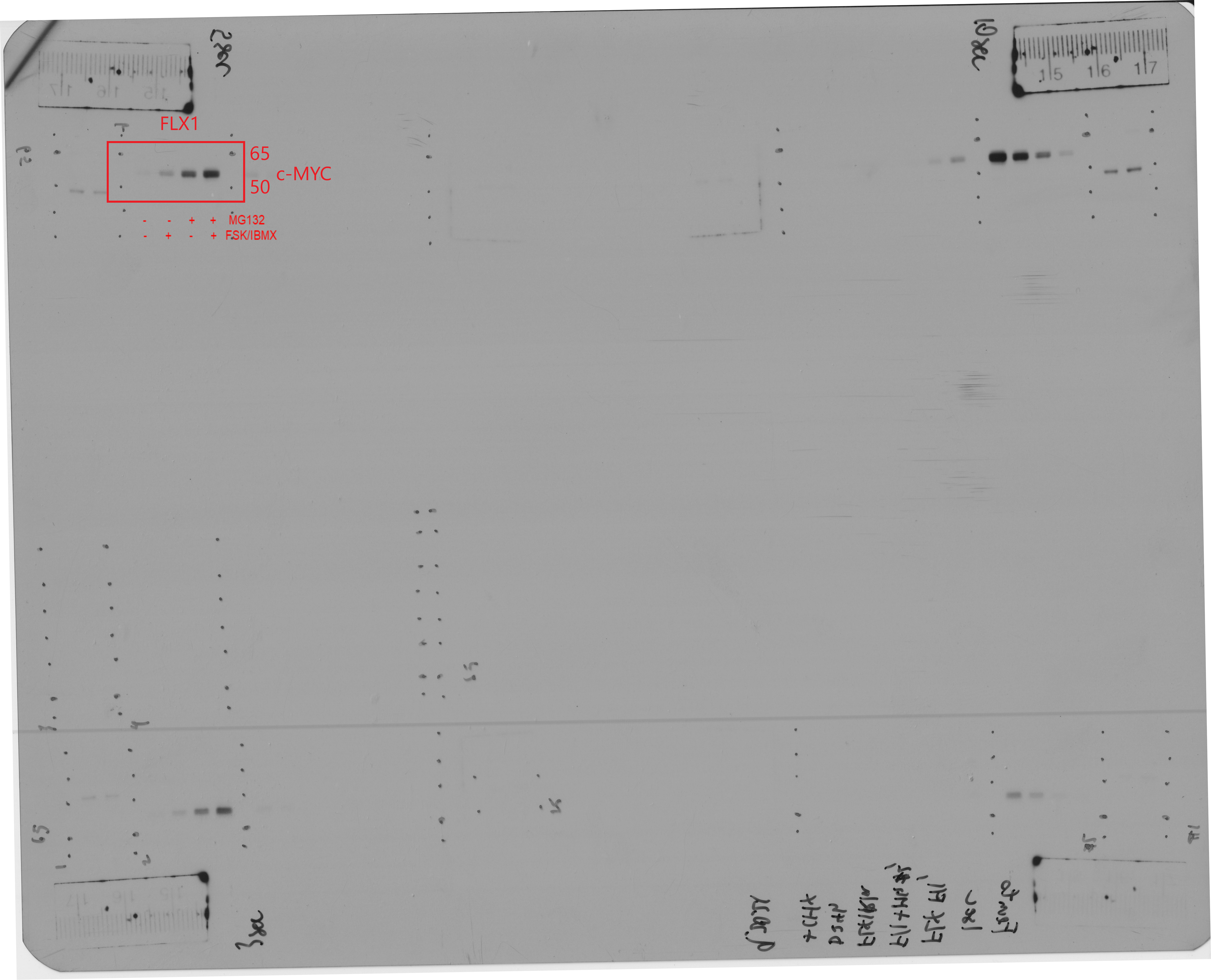

Supplement: Figure 5—figure supplement 2—source data 1. [file elife-69521-fig5-figsupp2-data1.zip › Figure S3A/Figure S3A c-MYC Labelled.tiff]

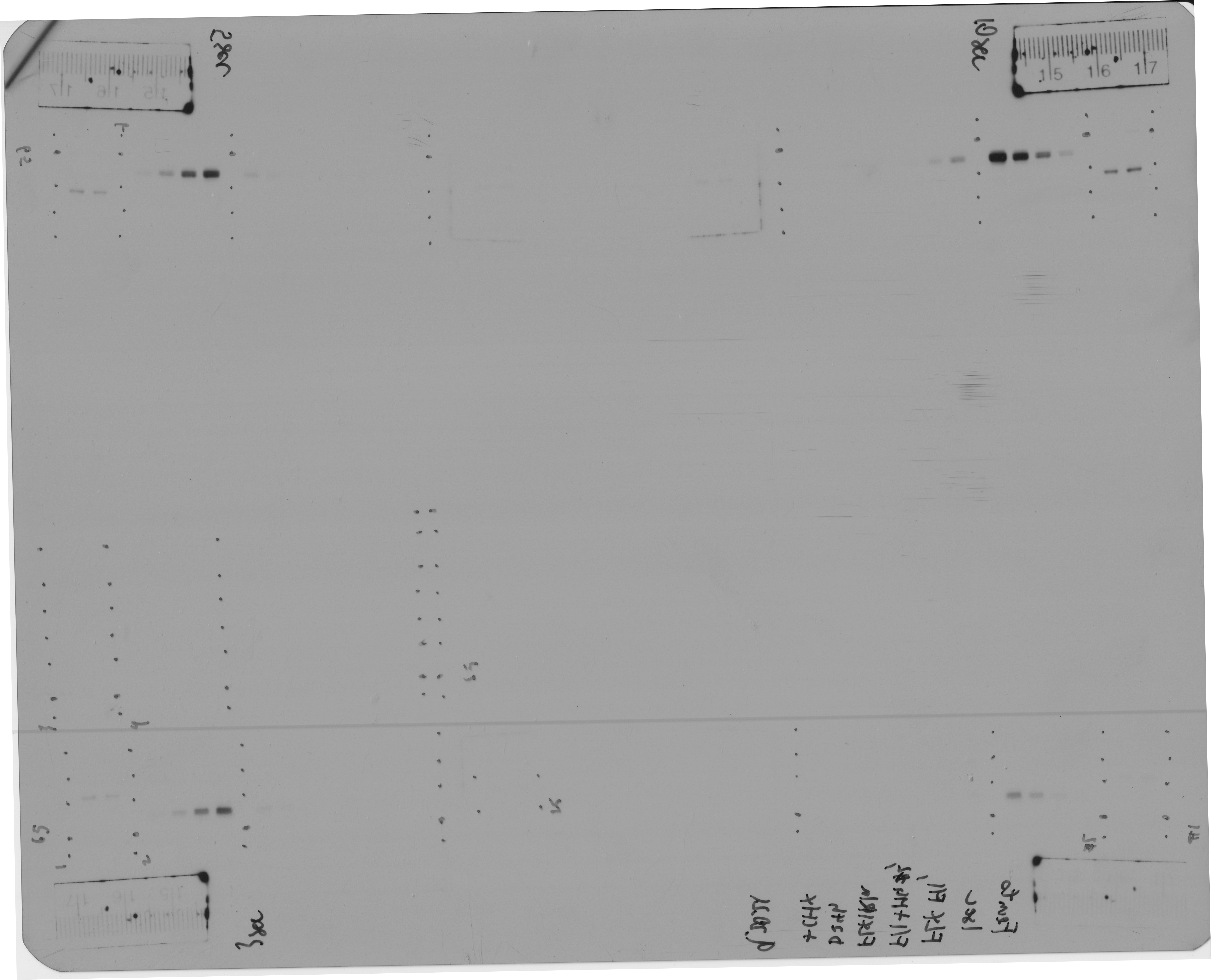

Supplement: Figure 5—figure supplement 2—source data 1. [file elife-69521-fig5-figsupp2-data1.zip › Figure S3A/Figure S3A c-MYC Raw.tiff]

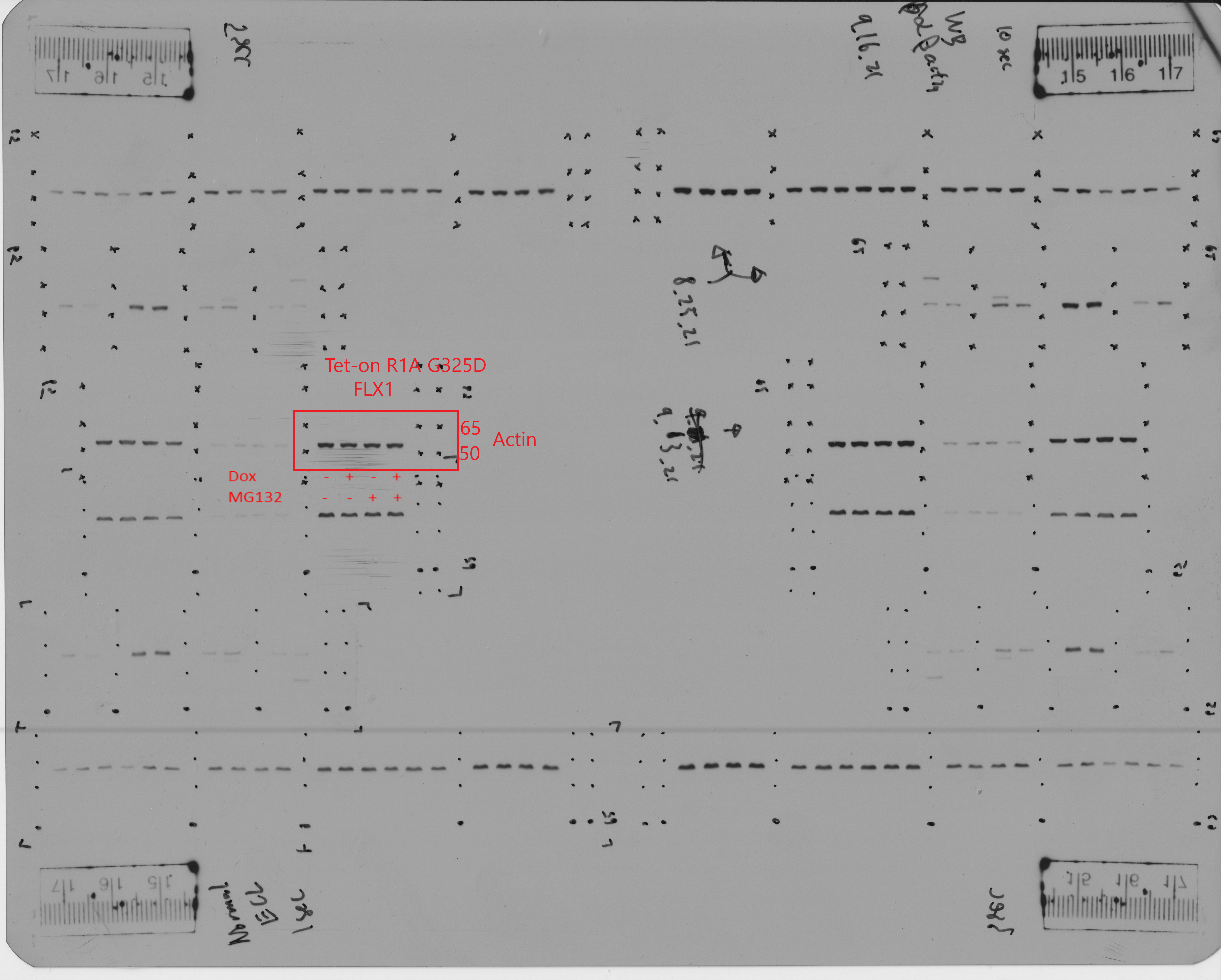

Supplement: Figure 5—figure supplement 2—source data 2. [file elife-69521-fig5-figsupp2-data2.zip › Figure S3B/Figure S3B FLX1 R1a Actin Labelled.tiff]

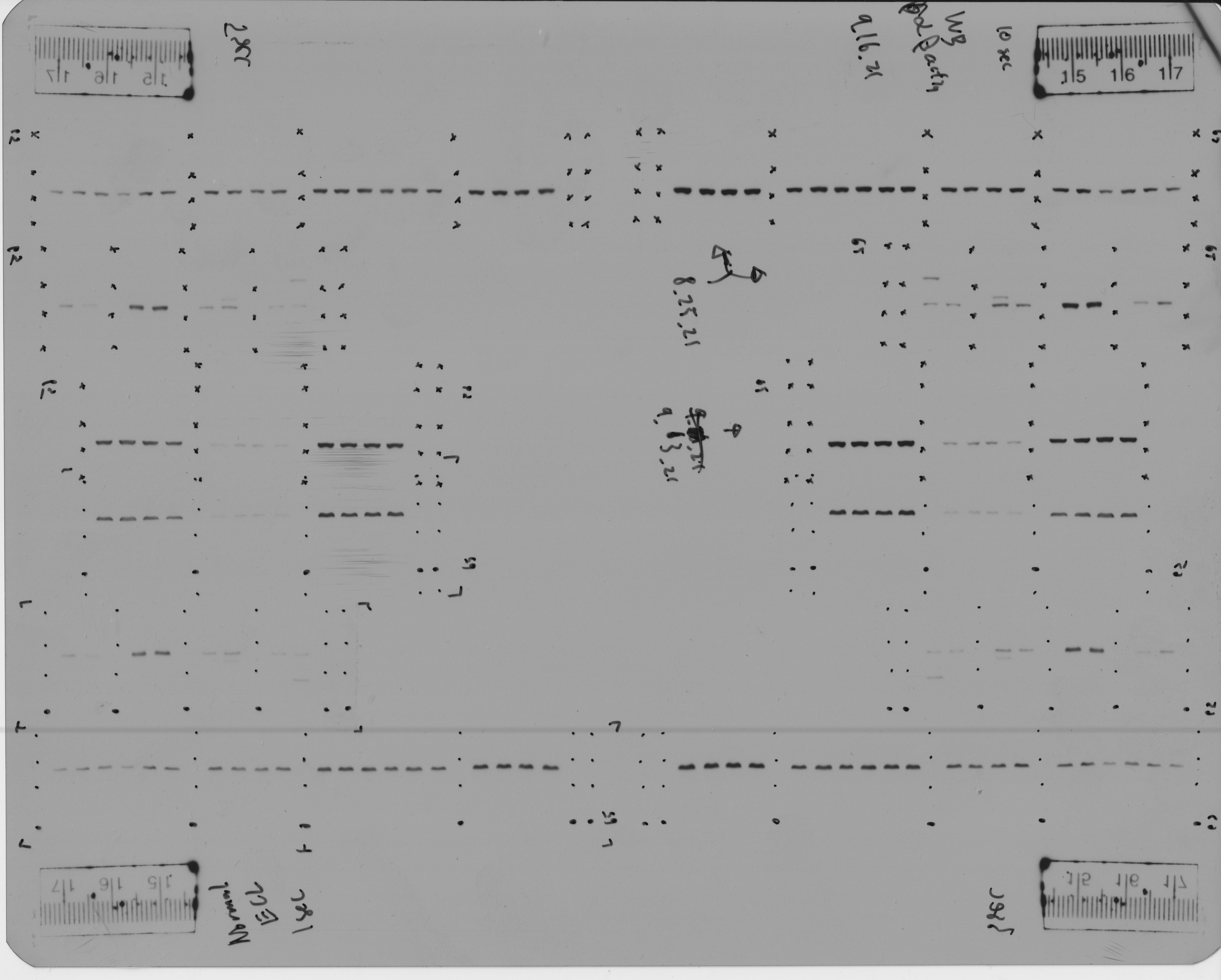

Supplement: Figure 5—figure supplement 2—source data 2. [file elife-69521-fig5-figsupp2-data2.zip › Figure S3B/Figure S3B FLX1 R1a Actin Raw.tiff]

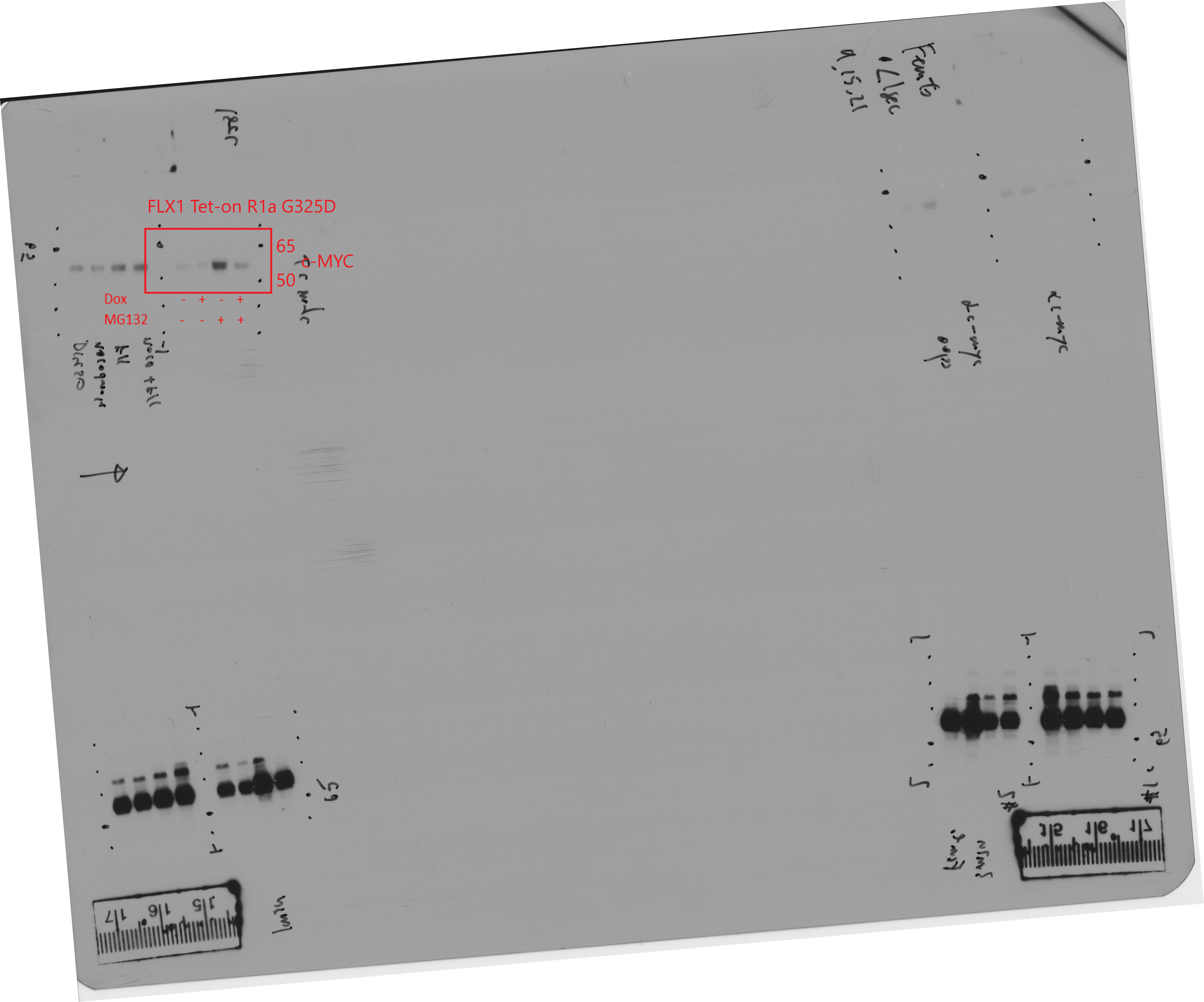

Supplement: Figure 5—figure supplement 2—source data 2. [file elife-69521-fig5-figsupp2-data2.zip › Figure S3B/Figure S3B FLX1 R1a c-MYC Labelled.tiff]

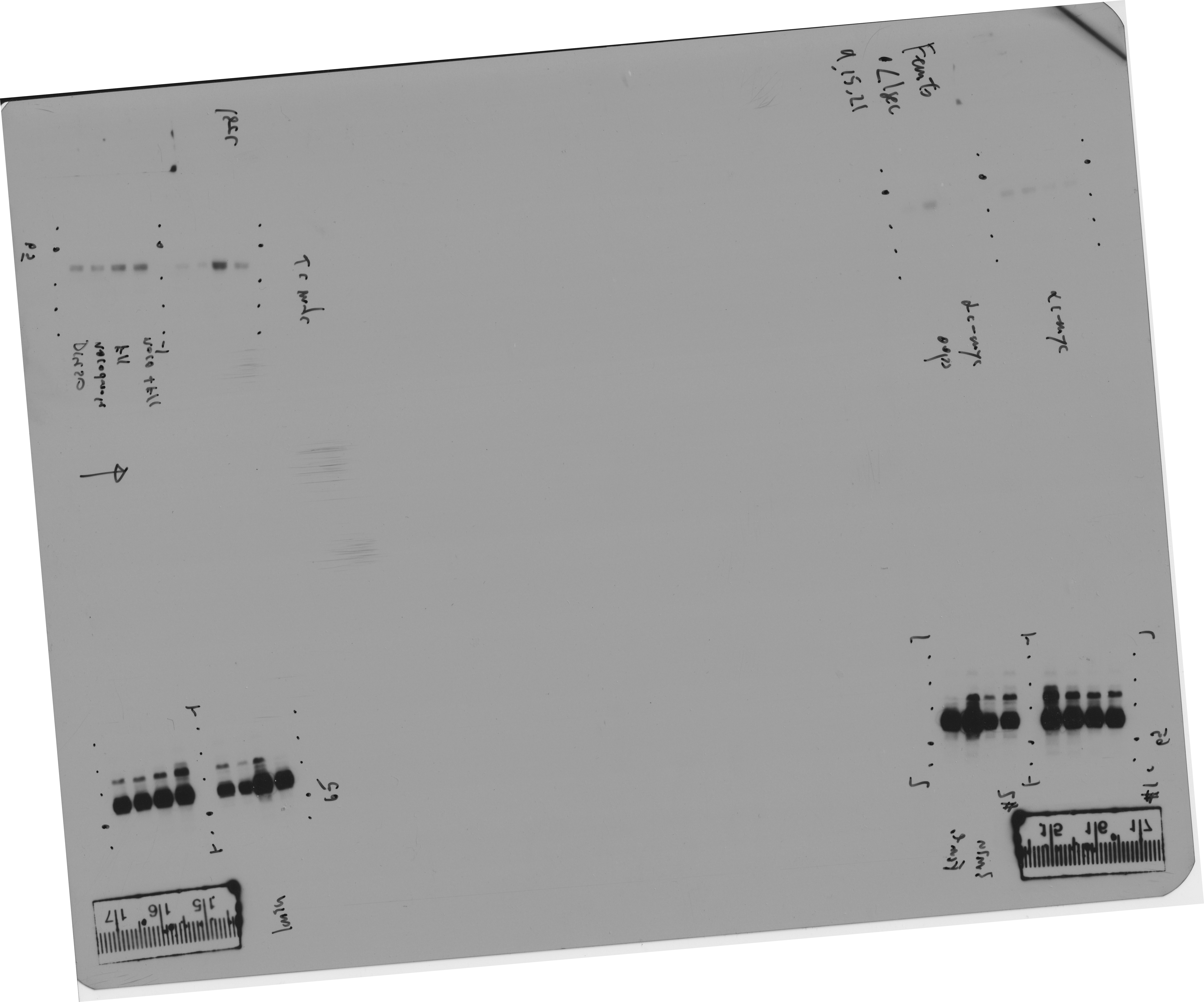

Supplement: Figure 5—figure supplement 2—source data 2. [file elife-69521-fig5-figsupp2-data2.zip › Figure S3B/Figure S3B FLX1 R1a c-MYC Raw.tiff]

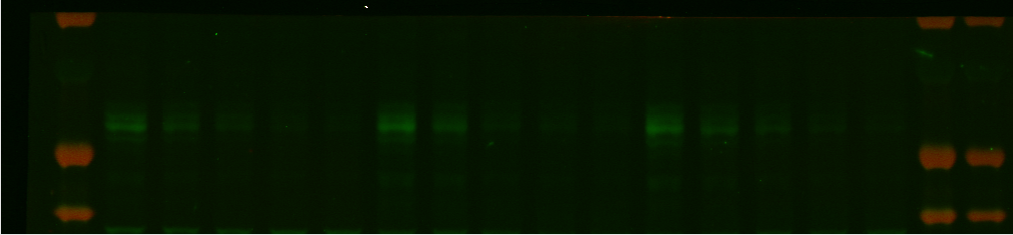

Supplement: Figure 5—figure supplement 2—source data 3. [file elife-69521-fig5-figsupp2-data3.zip › Figure S3C/Figure S3C cMYC.tif]

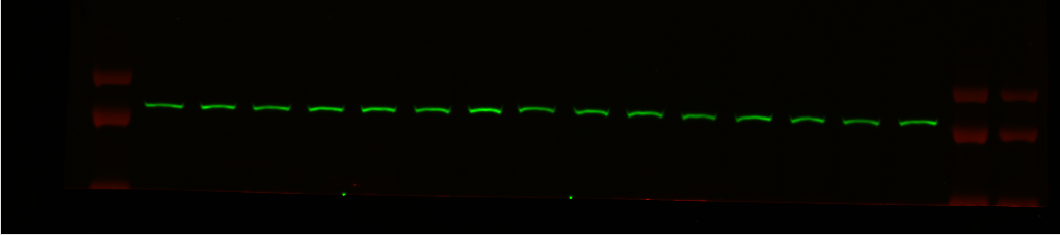

Supplement: Figure 5—figure supplement 2—source data 3. [file elife-69521-fig5-figsupp2-data3.zip › Figure S3C/Figure S3C Vinculin.tif]

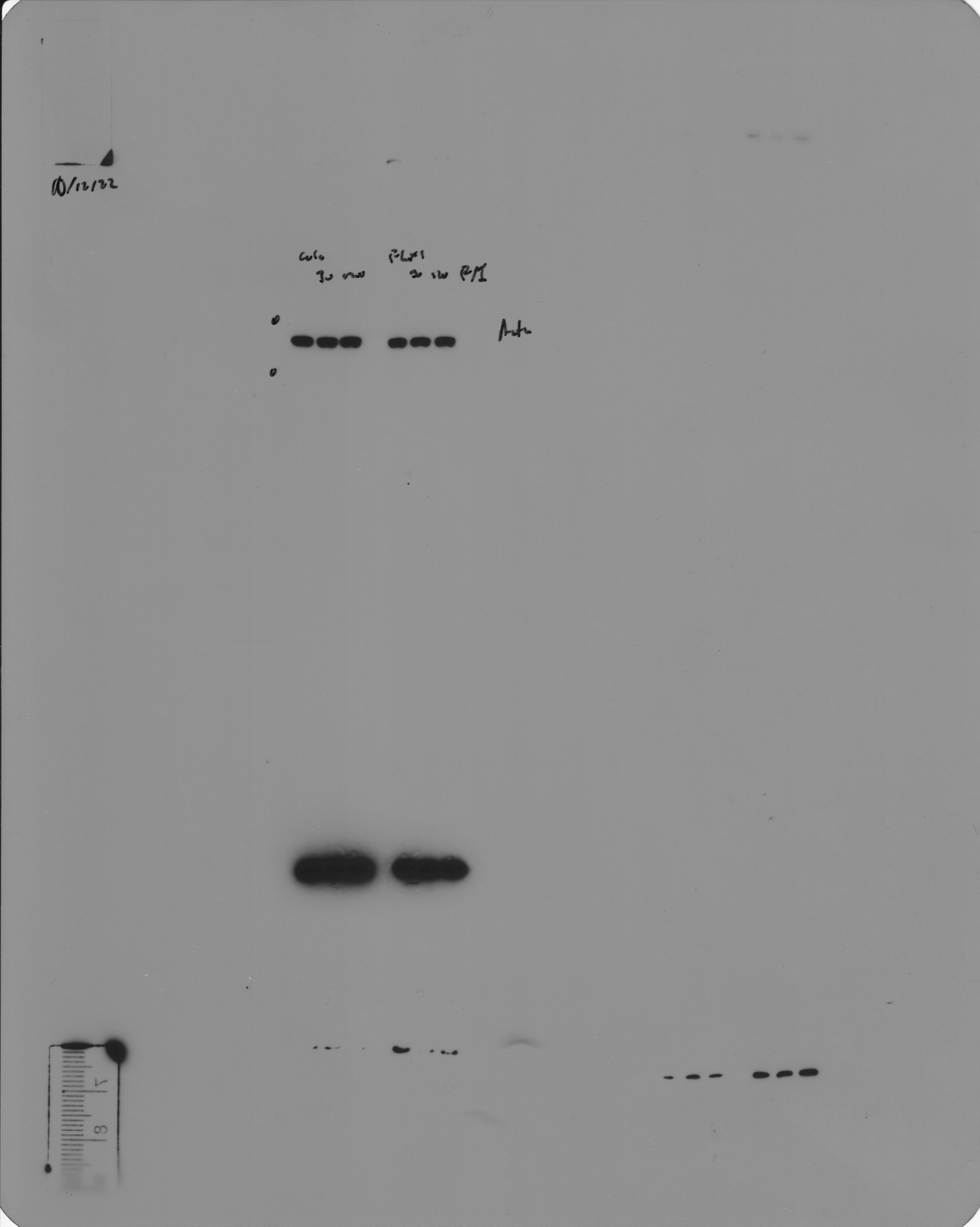

Supplement: Figure 6—source data 2. [file elife-69521-fig6-data2.zip › 6B/actin raw.tif]

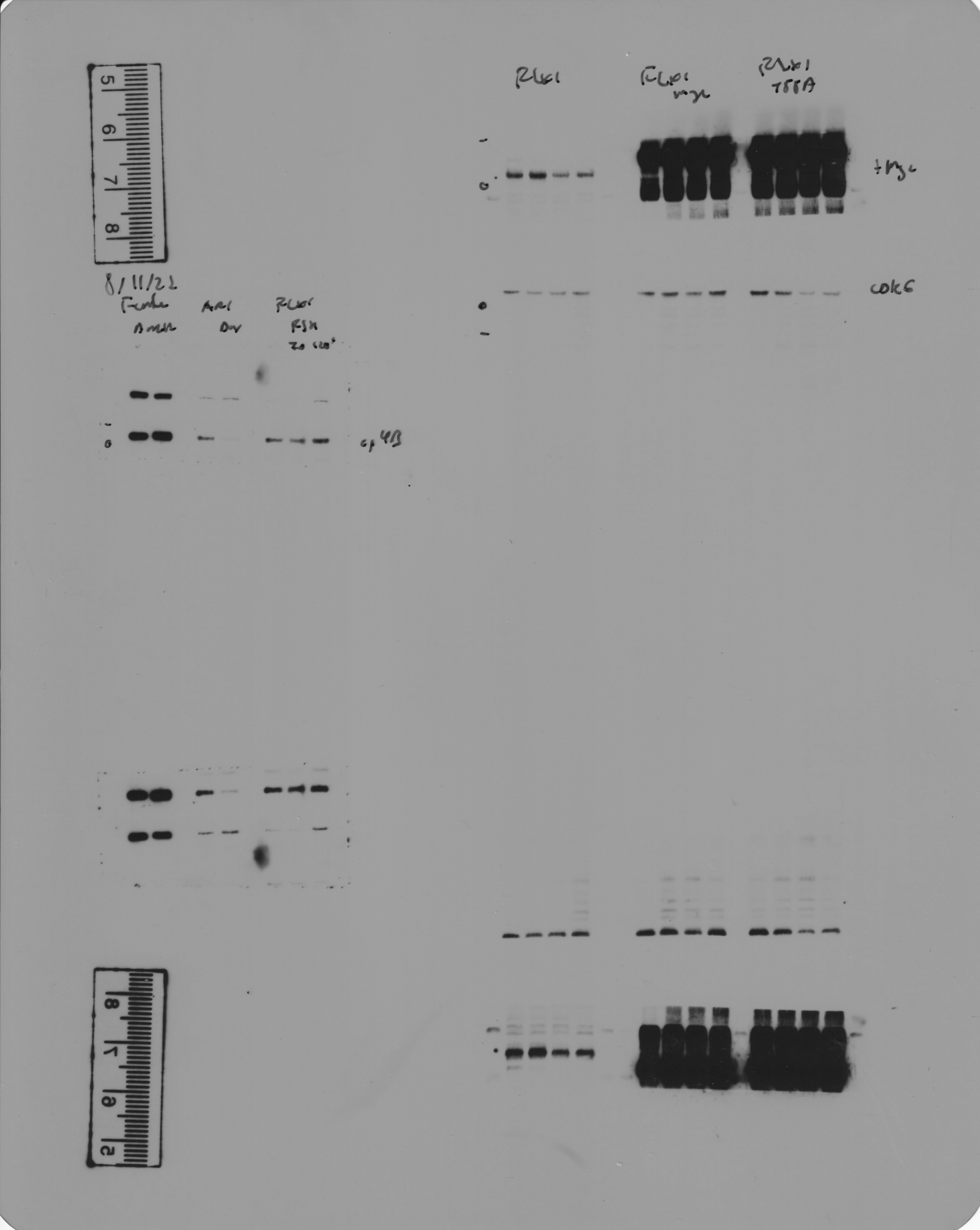

Supplement: Figure 6—source data 3. [file elife-69521-fig6-data3.zip › 6C/6C p4B raw.tif]

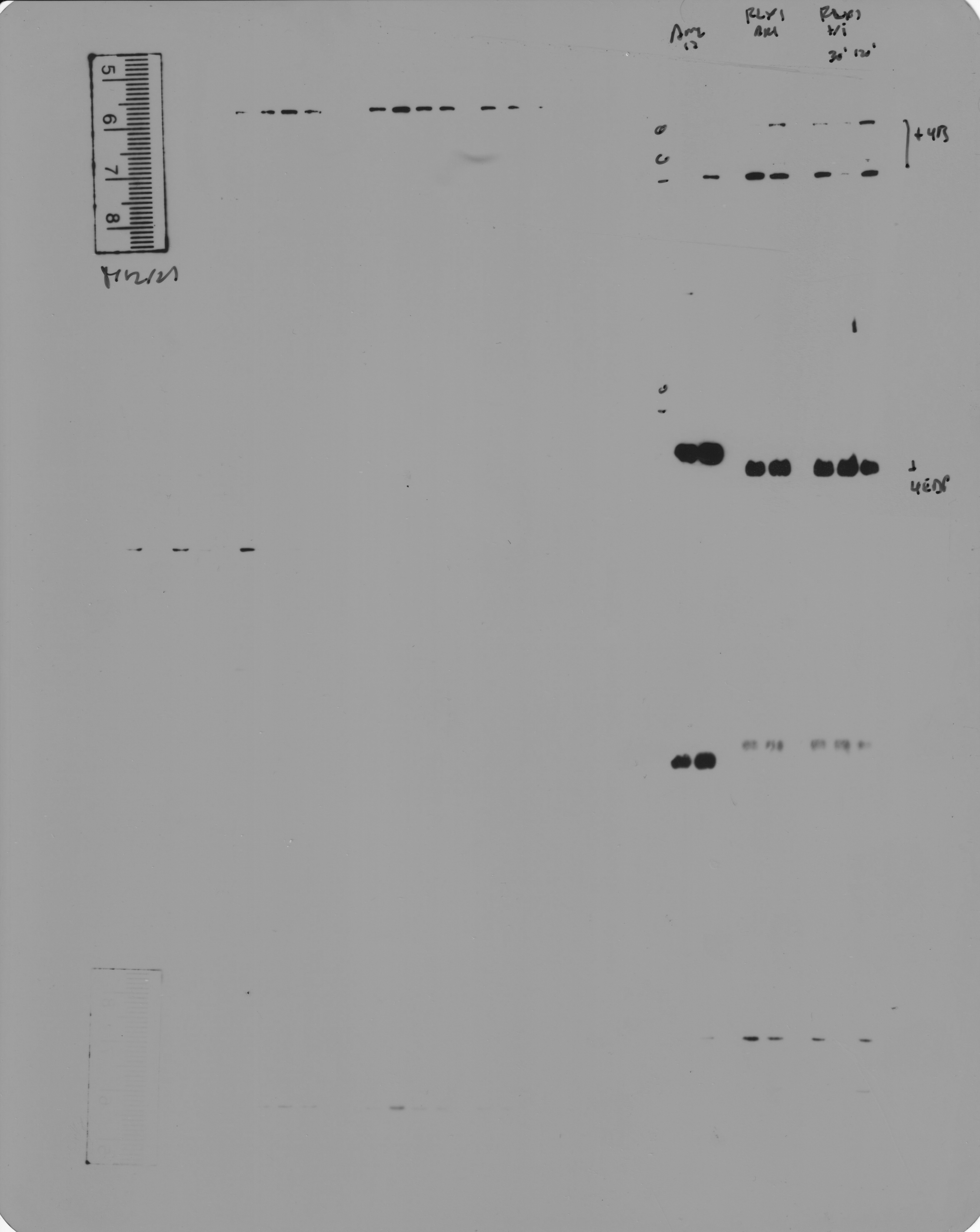

Supplement: Figure 6—source data 3. [file elife-69521-fig6-data3.zip › 6C/6C t4B raw.tif]

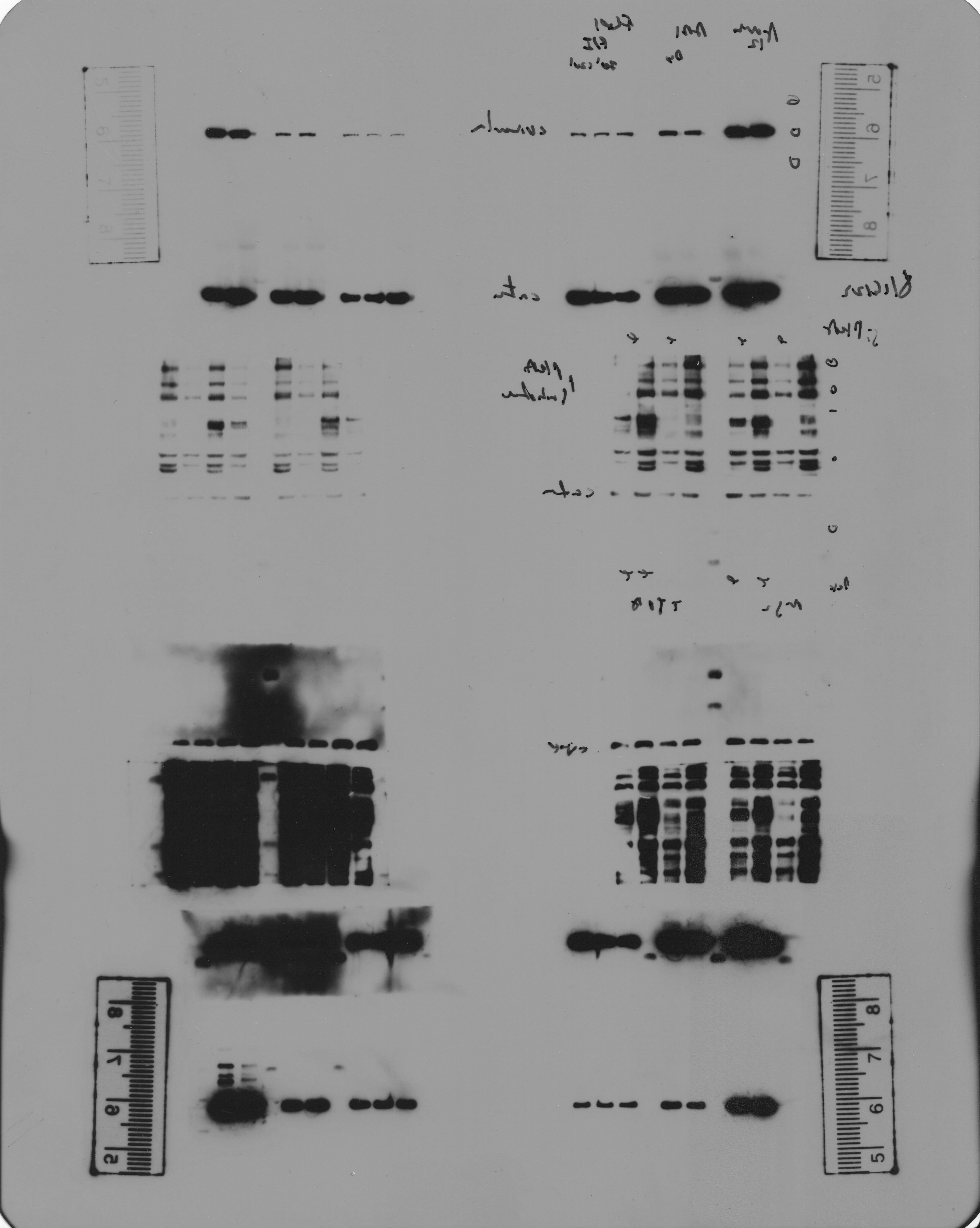

Supplement: Figure 6—source data 3. [file elife-69521-fig6-data3.zip › 6C/6C vinculin raw.tif]

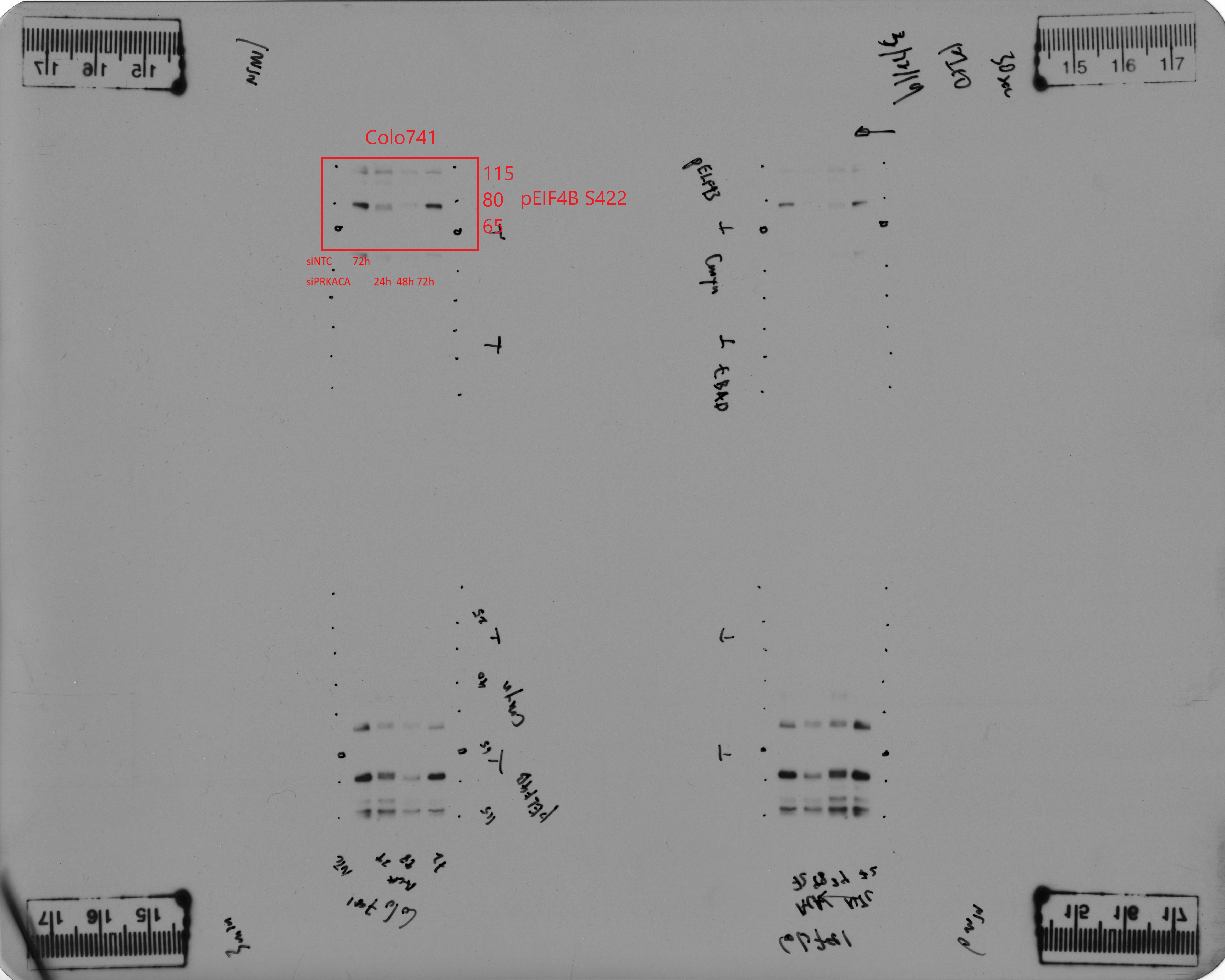

Supplement: Figure 6—source data 4. [file elife-69521-fig6-data4.zip › Colo741 pElF4BS422 Labelled.tif]

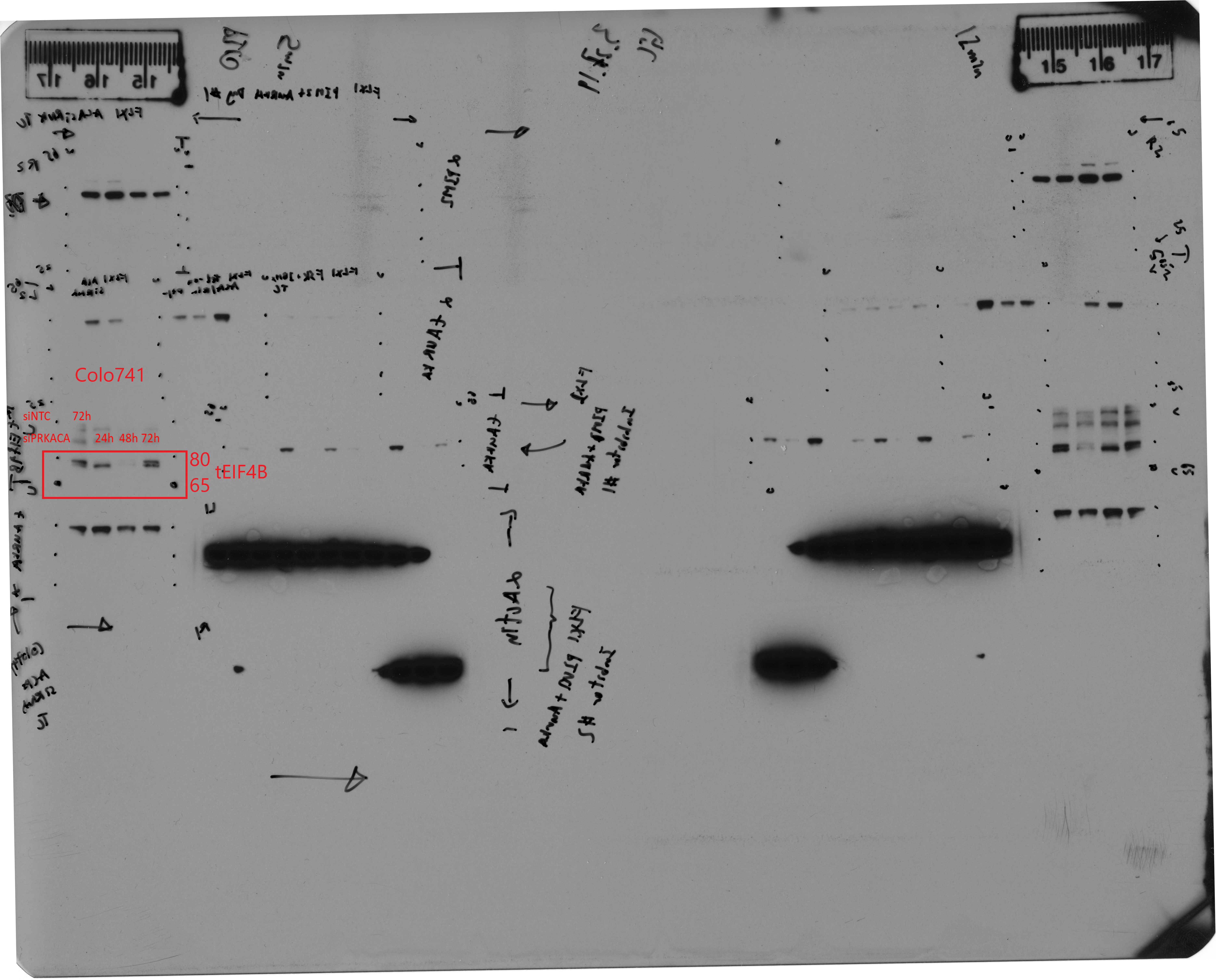

Supplement: Figure 6—source data 4. [file elife-69521-fig6-data4.zip › Colo741 tEIF4B Labelled.tif]

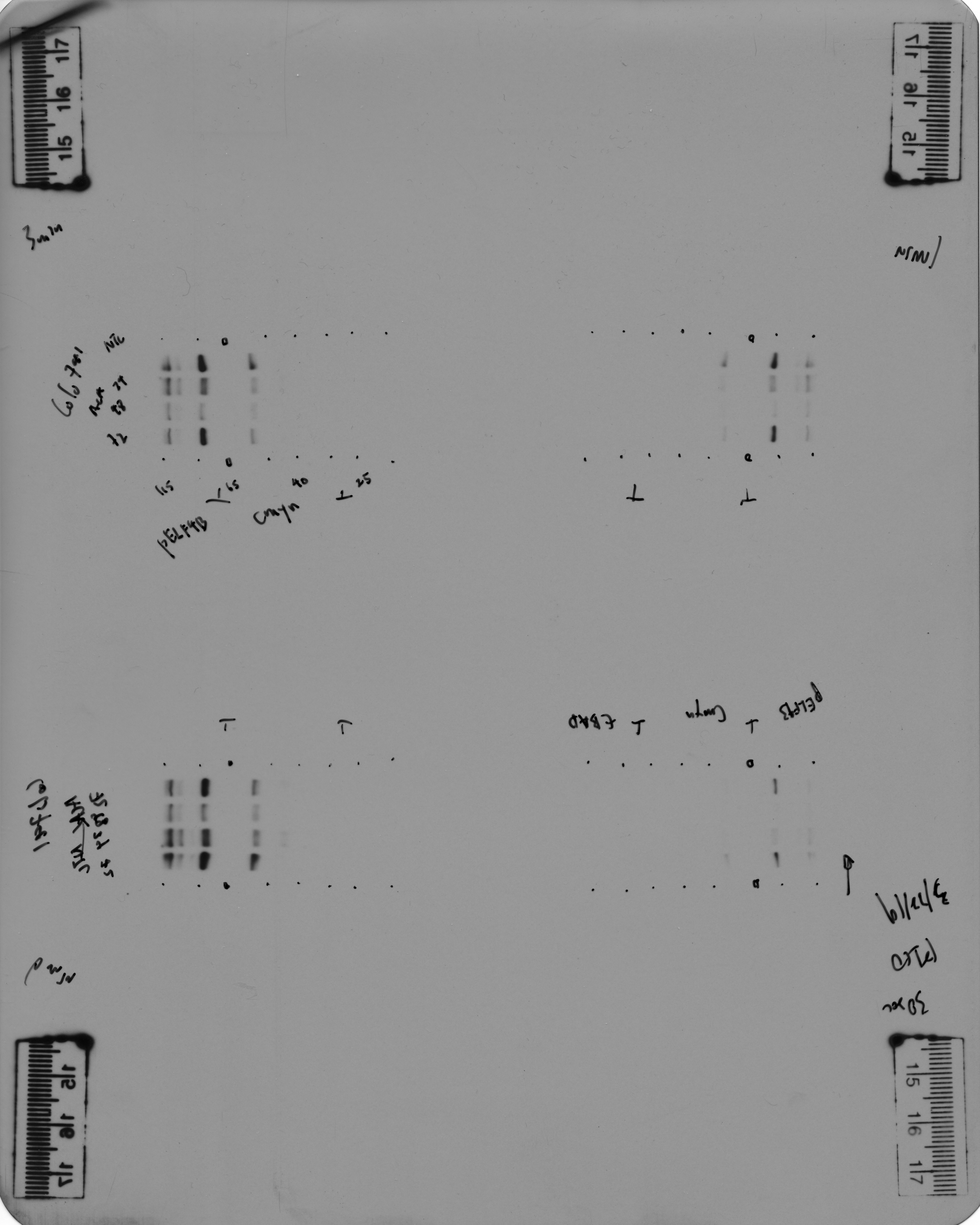

Supplement: Figure 6—source data 5. [file elife-69521-fig6-data5.zip › Figure 6D Colo741 c-MYC Raw.tif]

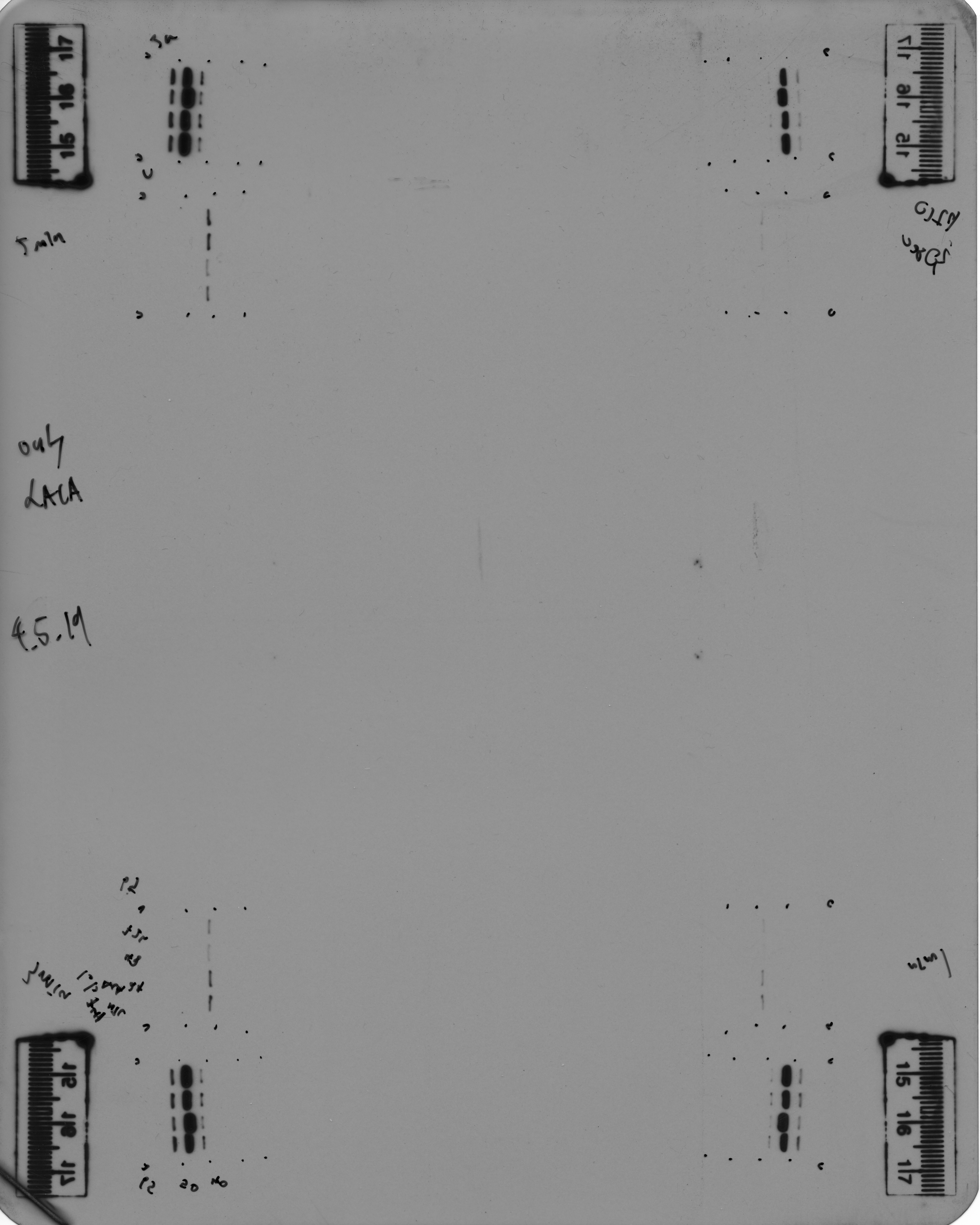

Supplement: Figure 6—source data 5. [file elife-69521-fig6-data5.zip › Figure 6D Colo741 PKAc Raw.tif]

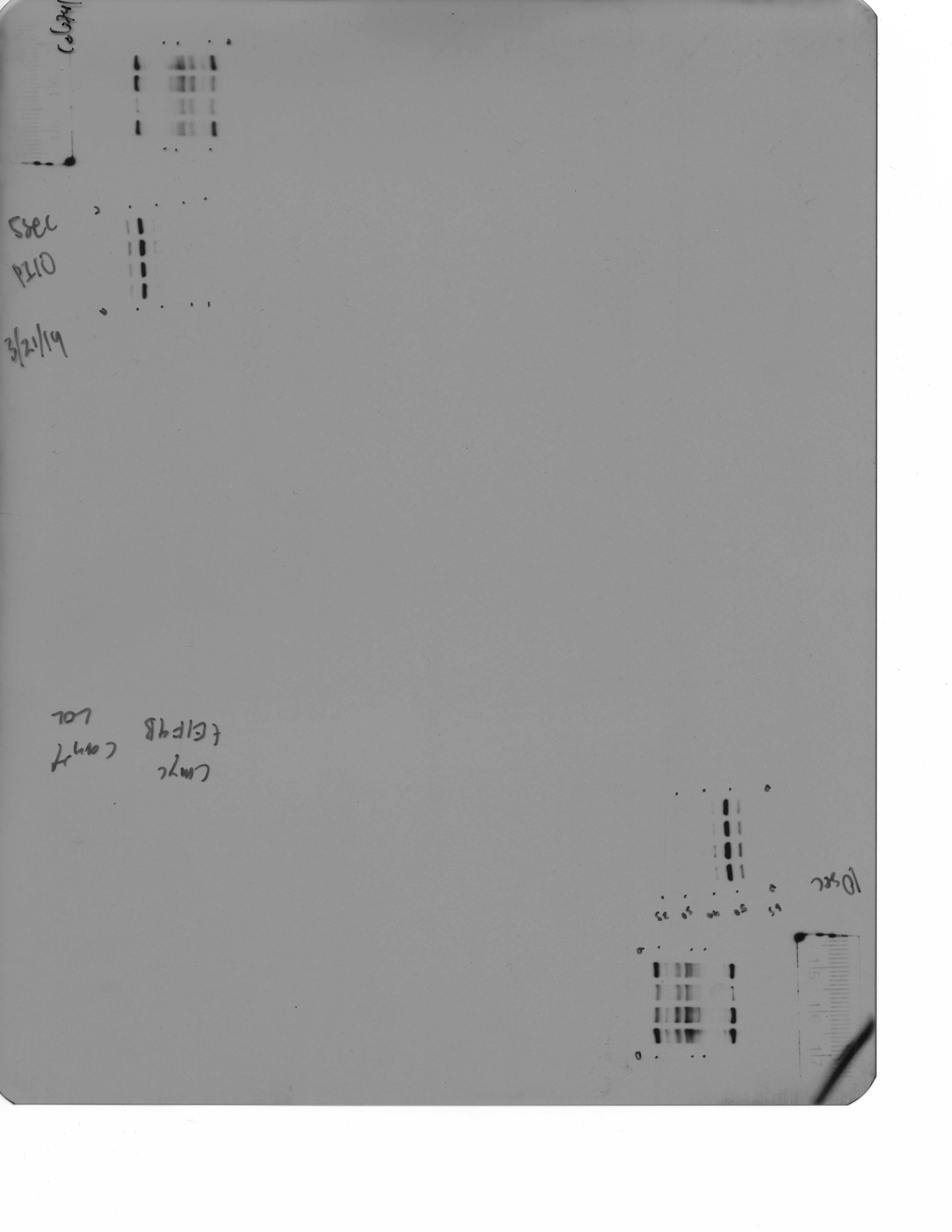

Supplement: Figure 6—source data 5. [file elife-69521-fig6-data5.zip › Figure 6D Colo741 pPKA Substrate Raw.tif]

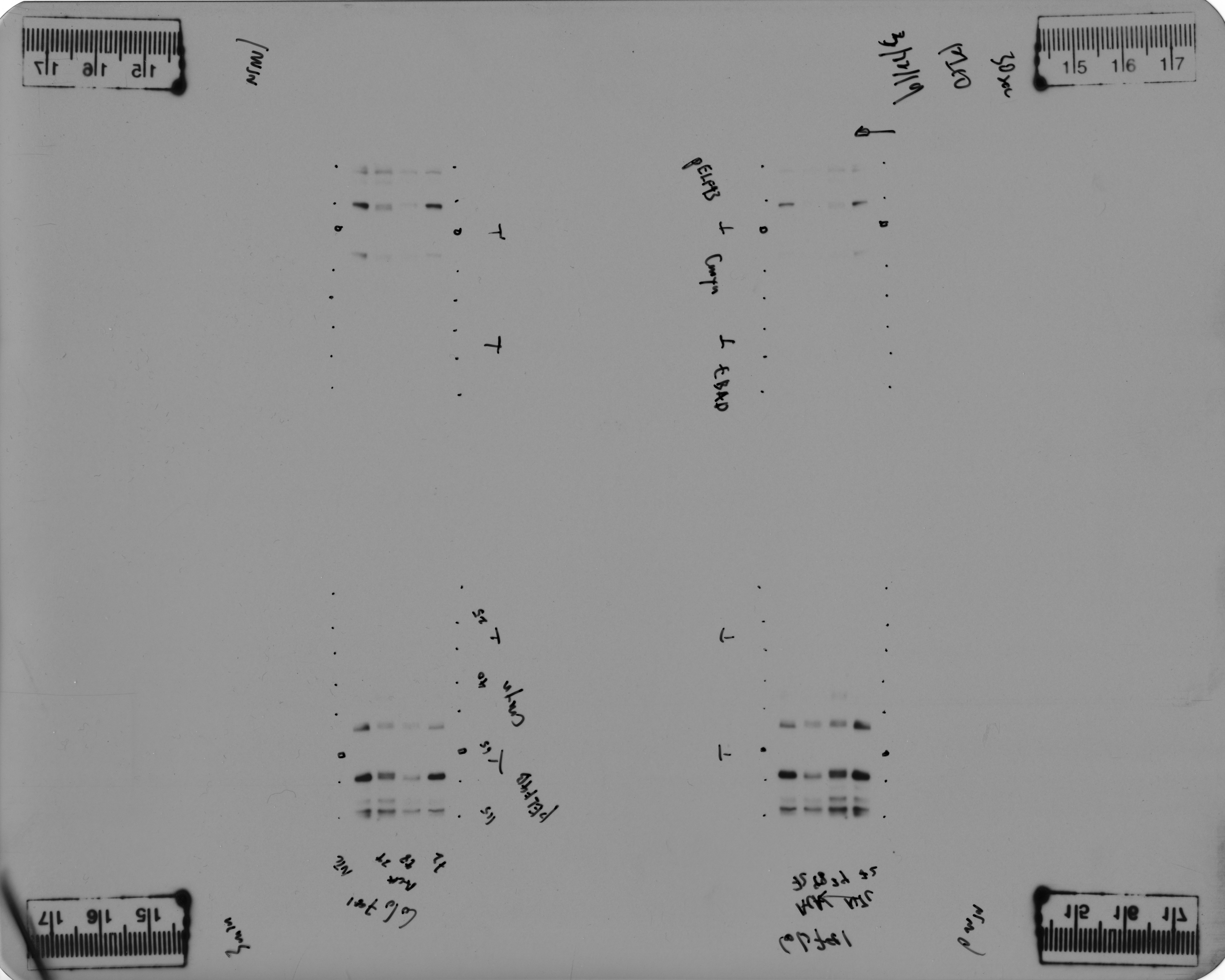

Supplement: Figure 6—source data 5. [file elife-69521-fig6-data5.zip › Colo741 pElF4BS422 Raw.tif]

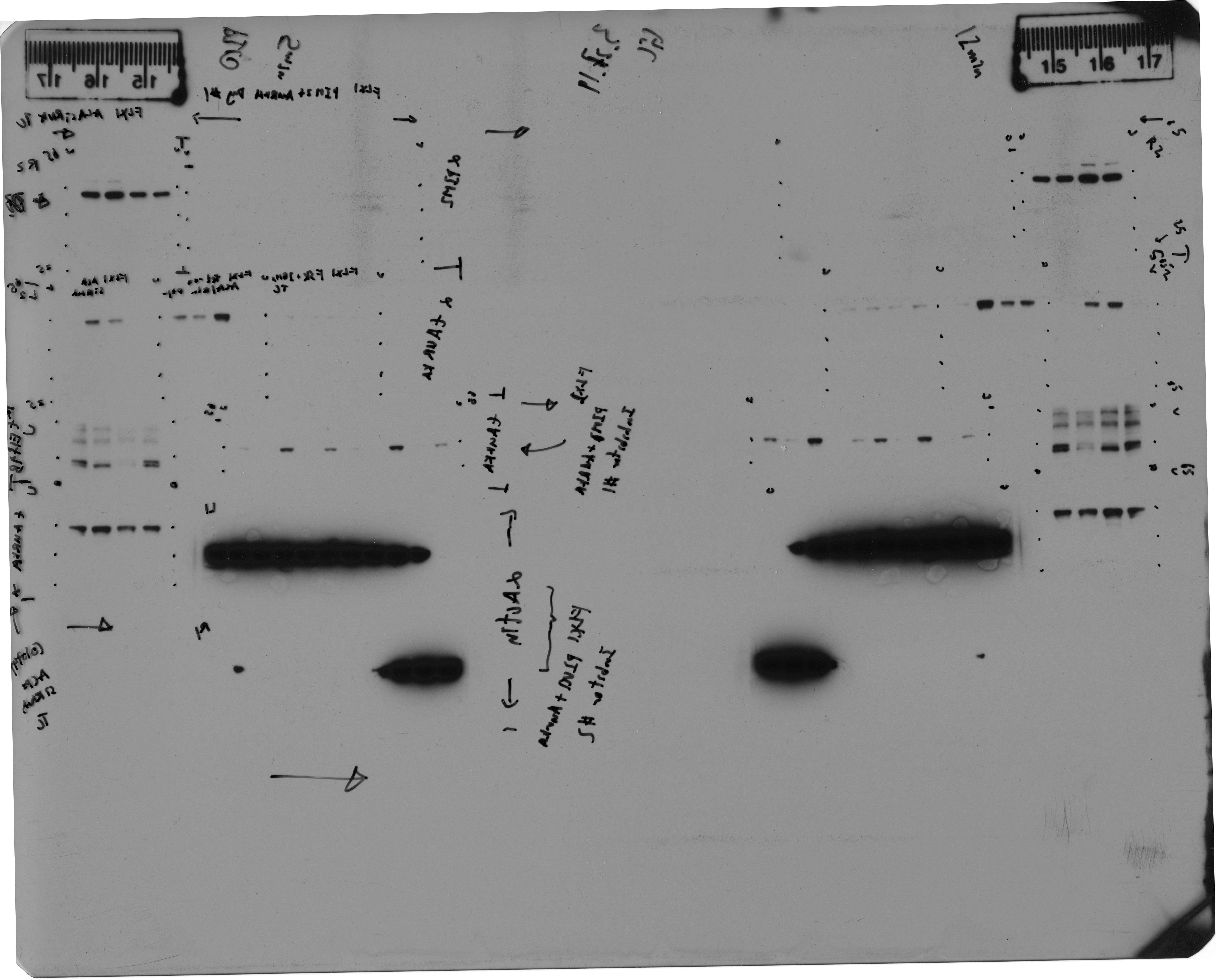

Supplement: Figure 6—source data 5. [file elife-69521-fig6-data5.zip › Colo741 tEIF4B Raw.tif]

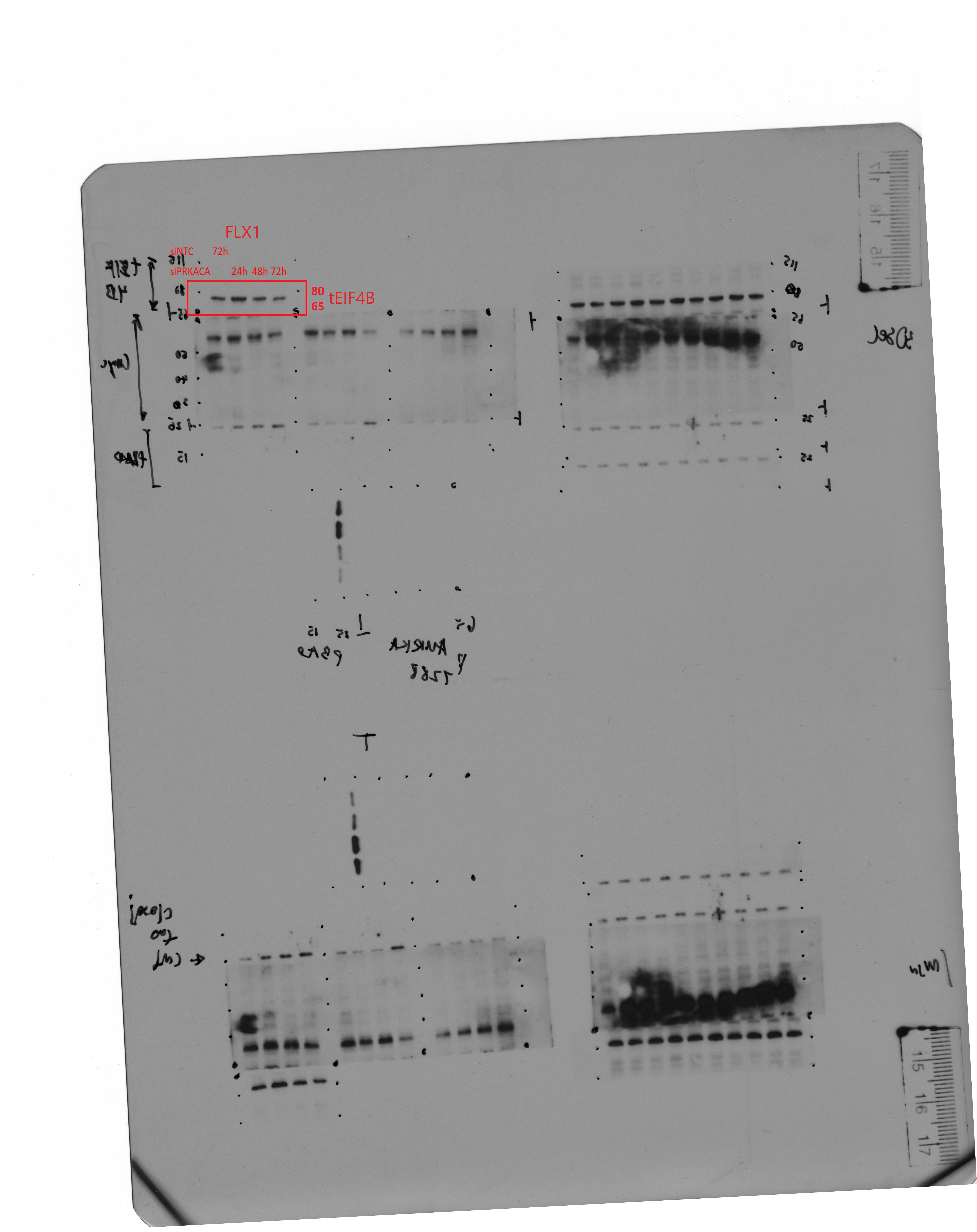

Supplement: Figure 6—source data 6. [file elife-69521-fig6-data6.zip › FLX1 tElF4B Labelled.tif]

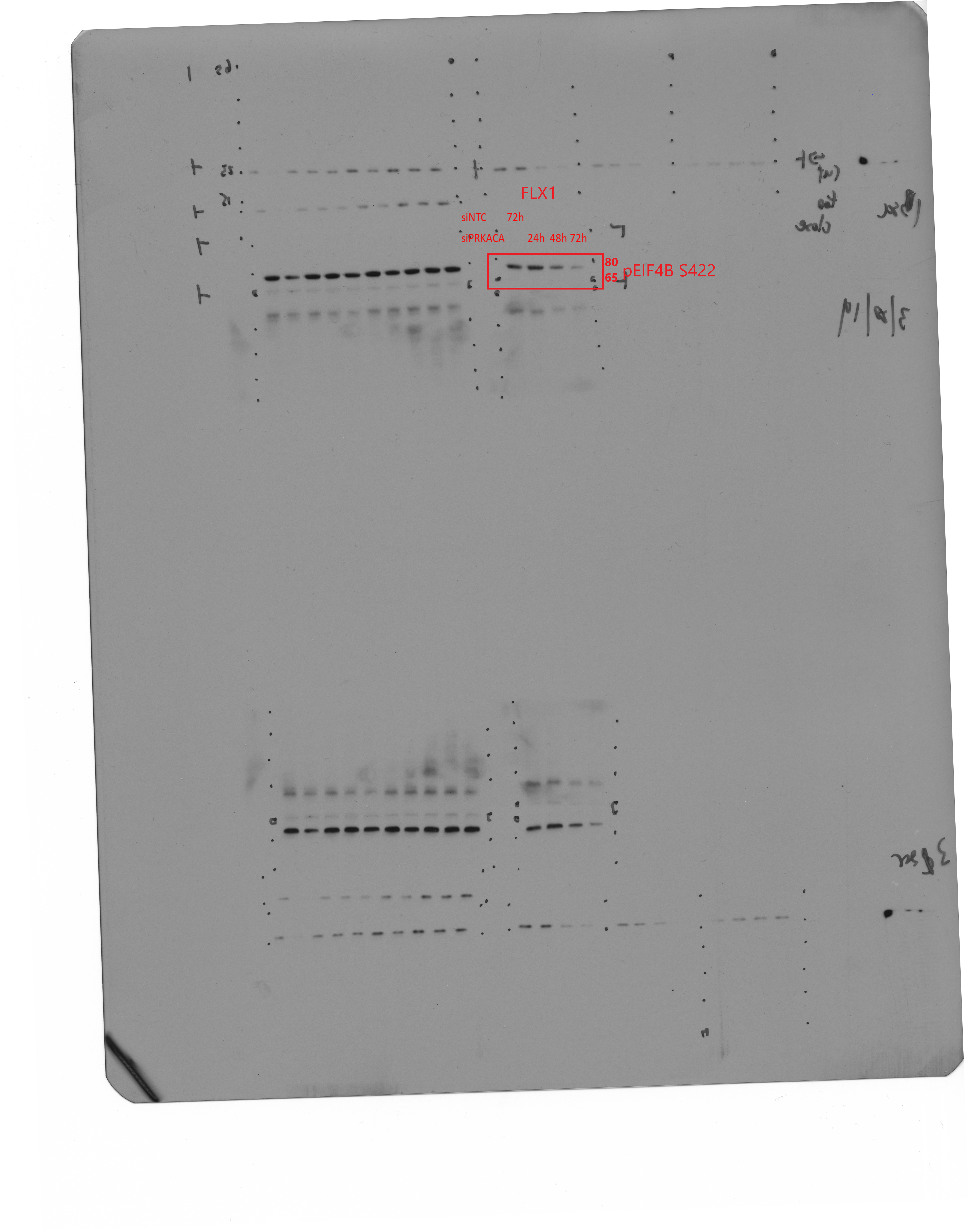

Supplement: Figure 6—source data 6. [file elife-69521-fig6-data6.zip › FLX1 pElF4BS422 Labelled.tif]

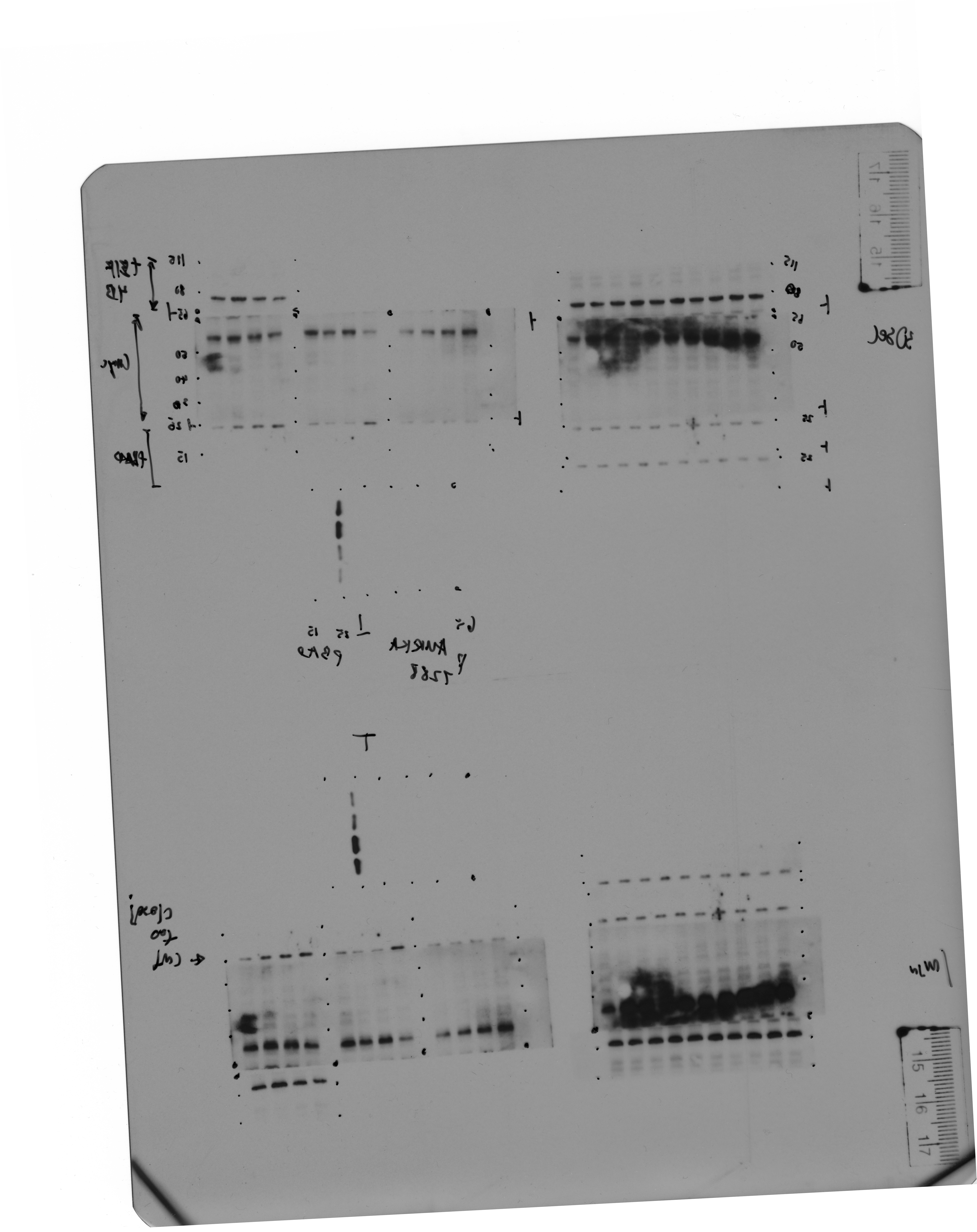

Supplement: Figure 6—source data 7. [file elife-69521-fig6-data7.zip › FLX1 tElF4B Raw.tif]

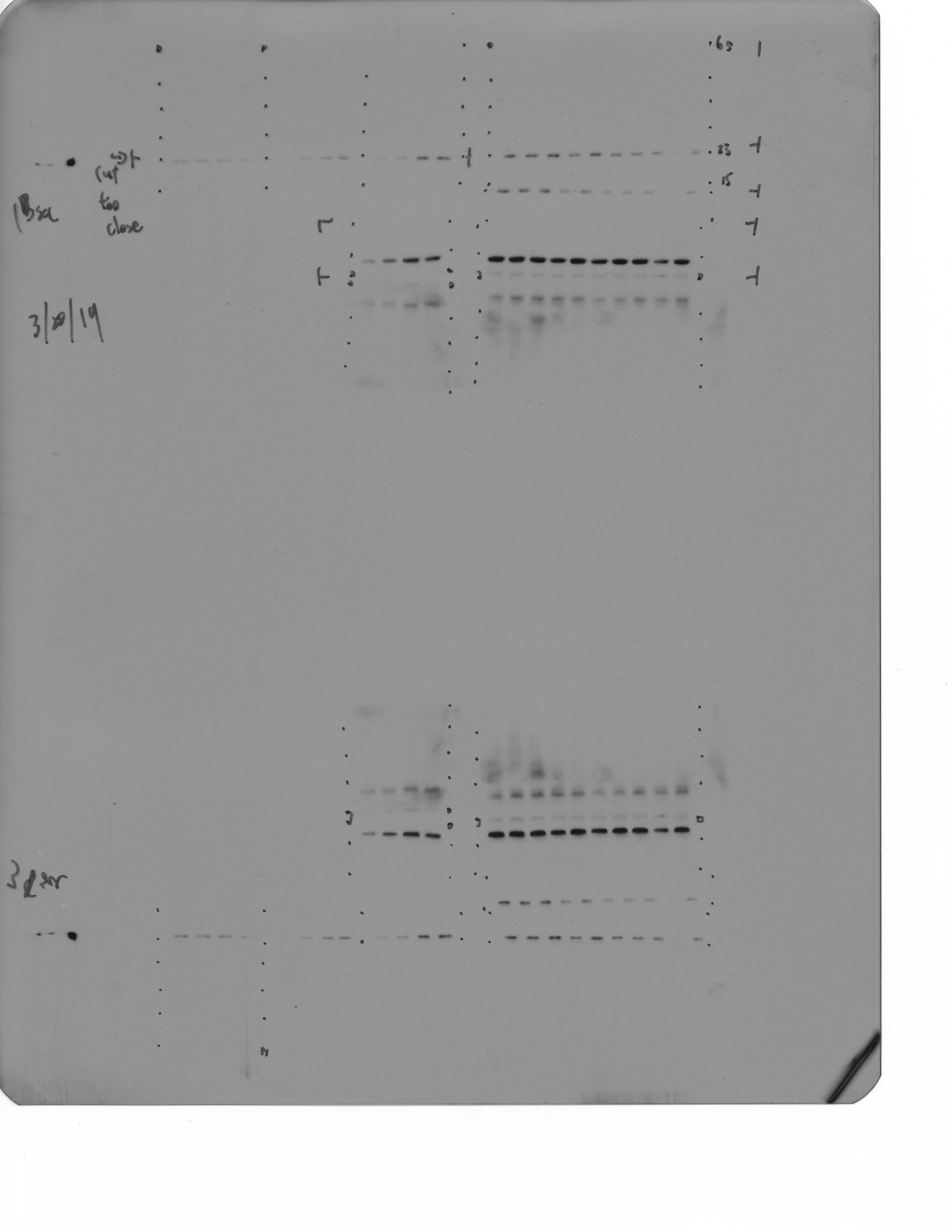

Supplement: Figure 6—source data 7. [file elife-69521-fig6-data7.zip › Figure 6D FLX1 c-MYC Raw.tif]

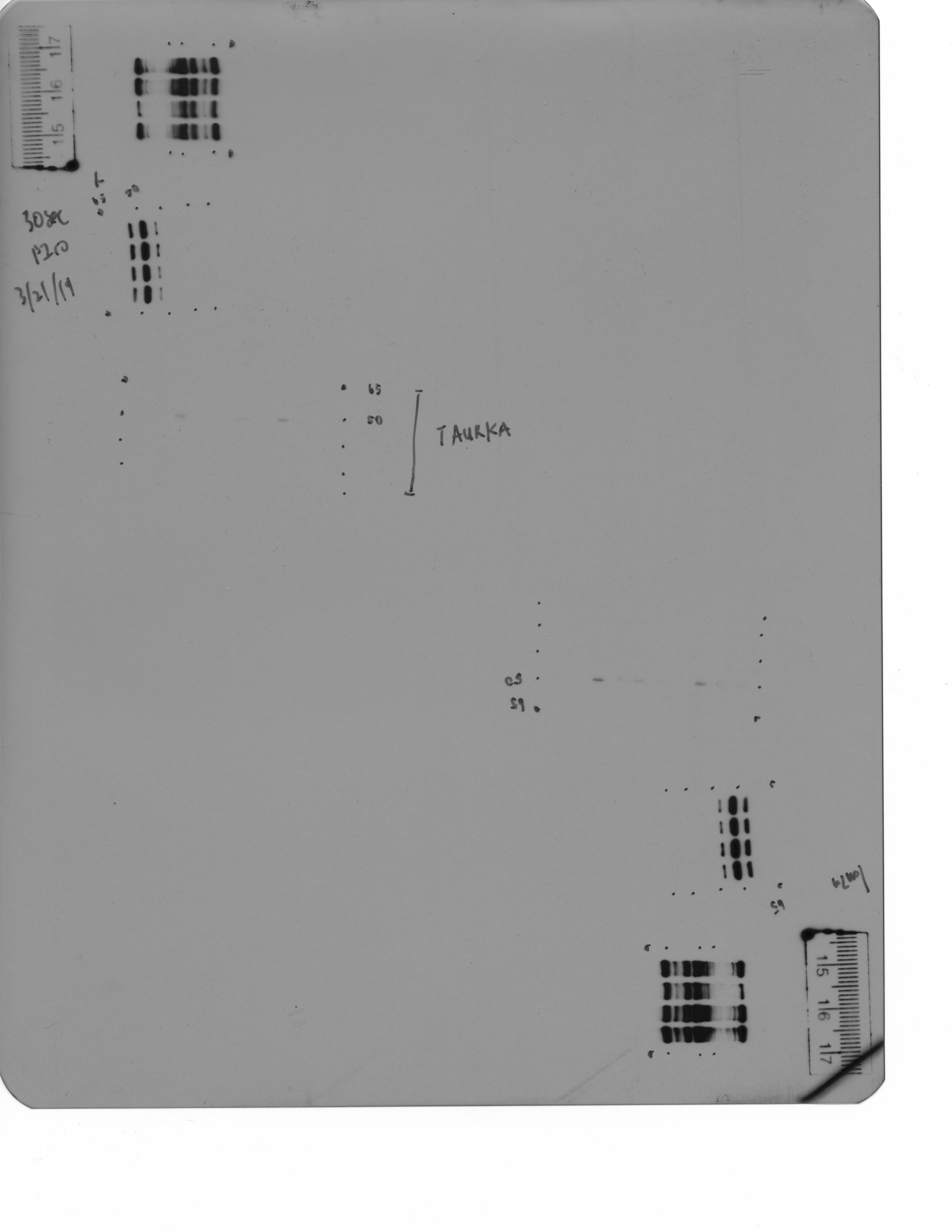

Supplement: Figure 6—source data 7. [file elife-69521-fig6-data7.zip › Figure 6D FLX1 PKAc Long Raw.tif]

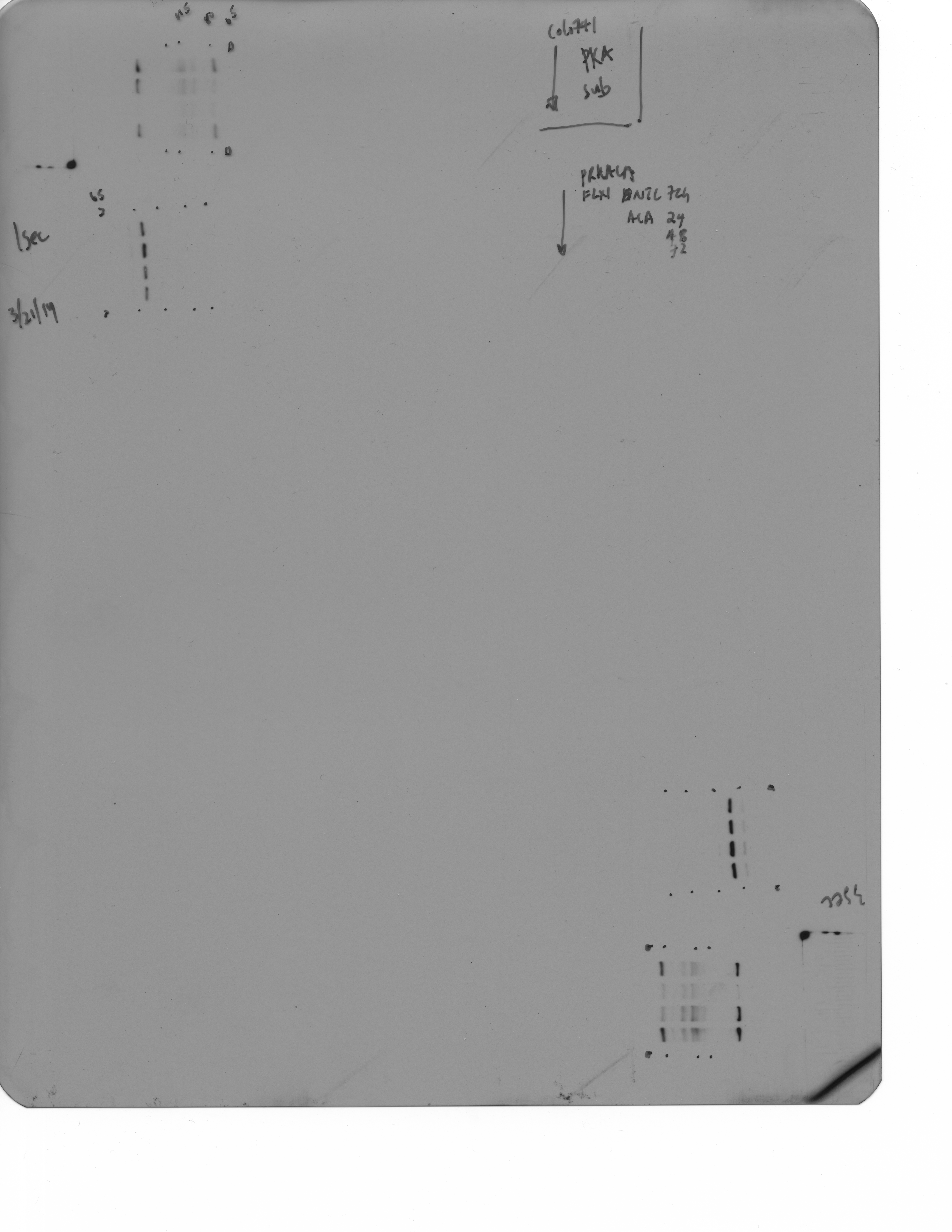

Supplement: Figure 6—source data 7. [file elife-69521-fig6-data7.zip › Figure 6D FLX1 PKAc Short Raw.tif]

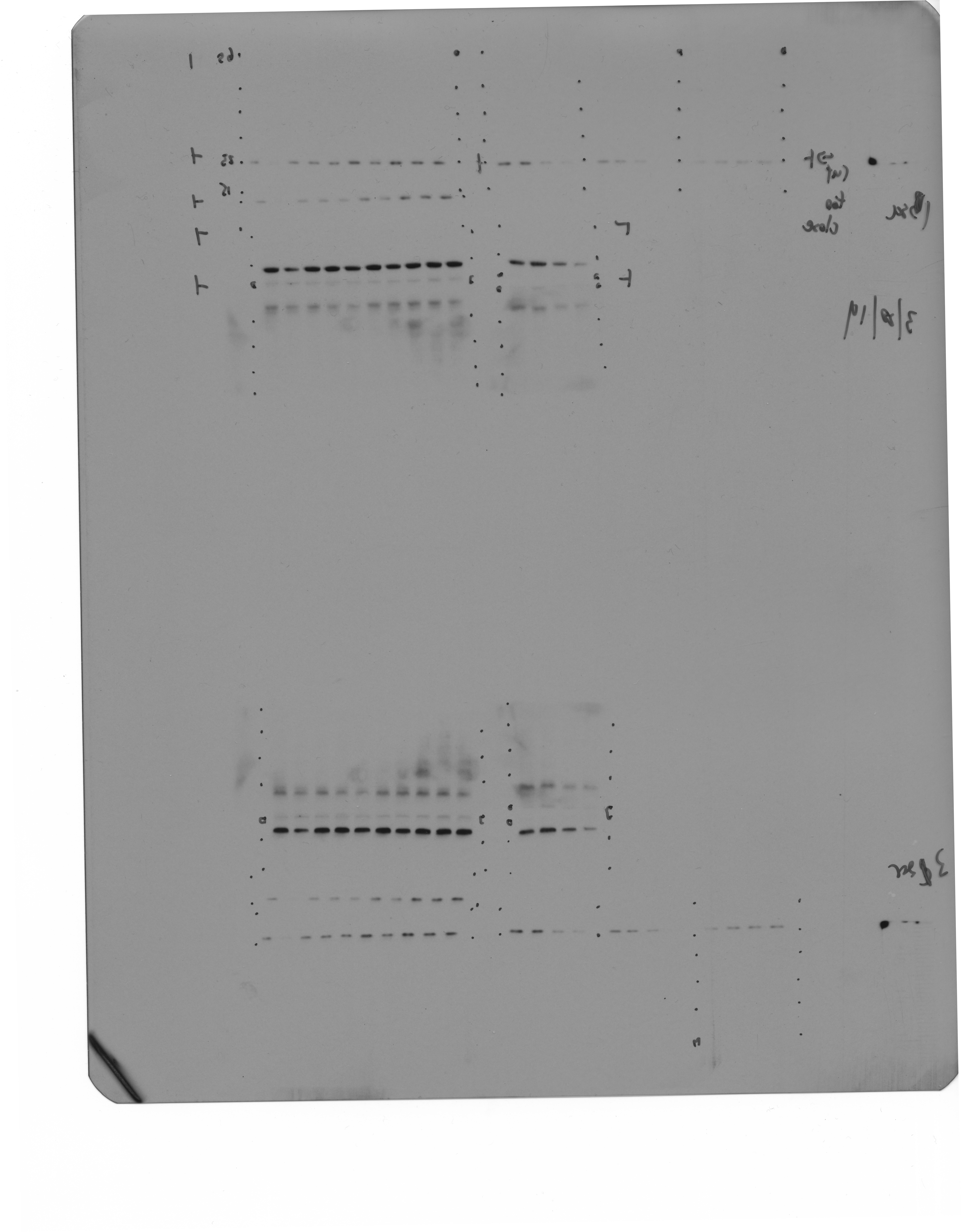

Supplement: Figure 6—source data 7. [file elife-69521-fig6-data7.zip › FLX1 pElF4BS422 Raw.tif]

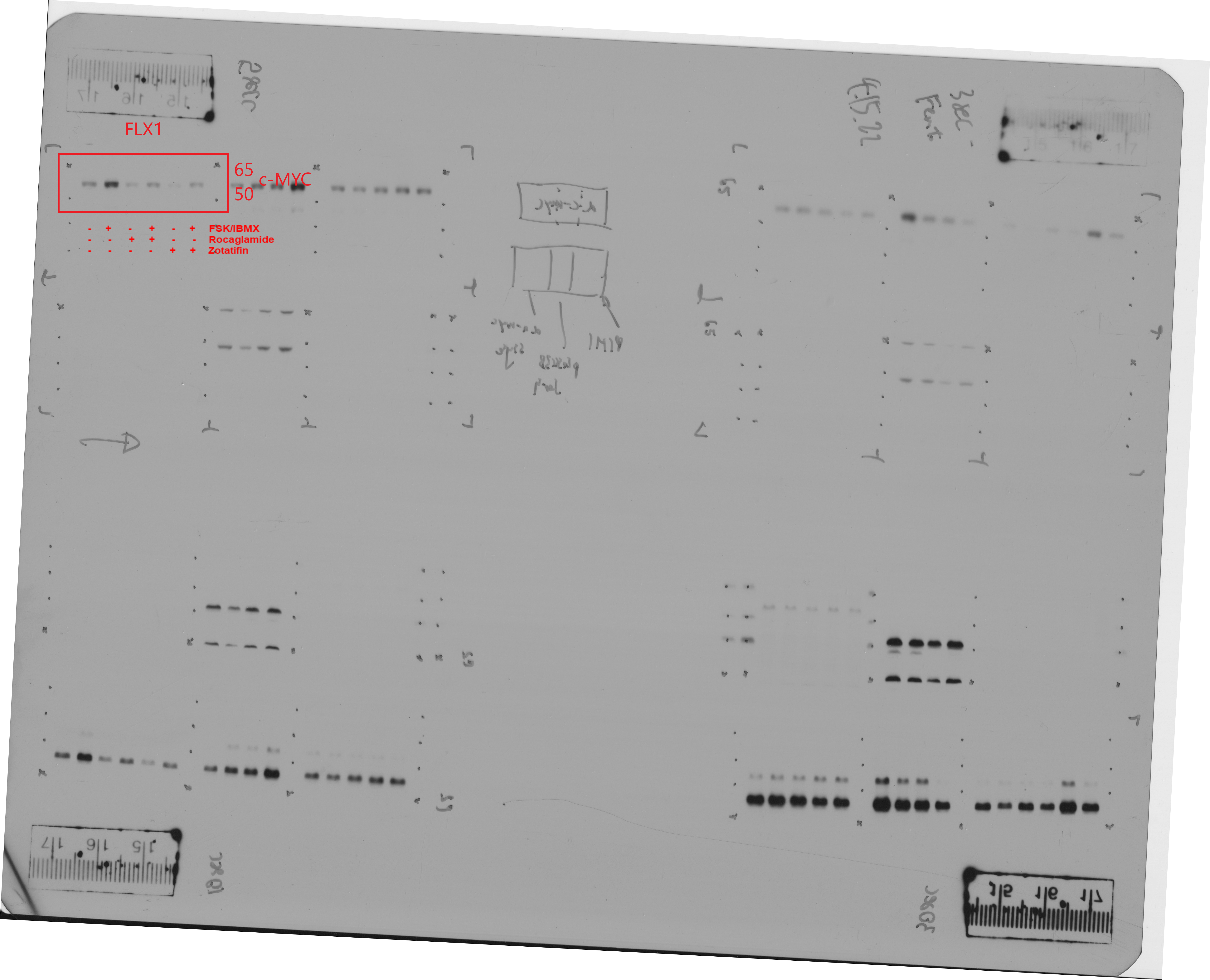

Supplement: Figure 7—source data 1. [file elife-69521-fig7-data1.zip › 7A/Figure 7A FLX1 c-MYC Labelled.tiff]

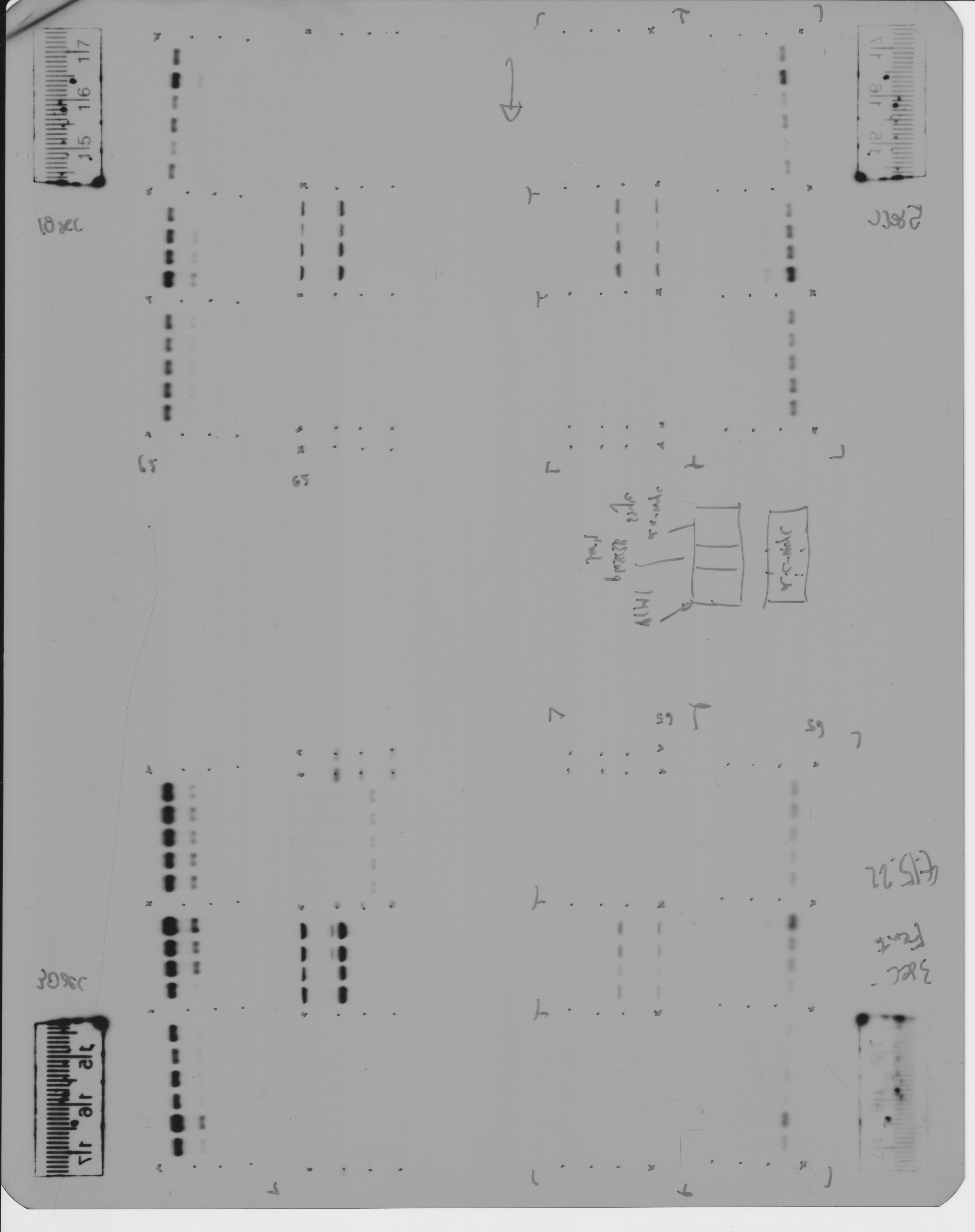

Supplement: Figure 7—source data 1. [file elife-69521-fig7-data1.zip › 7A/Figure 7A FLX1 c-MYC Raw.tiff]

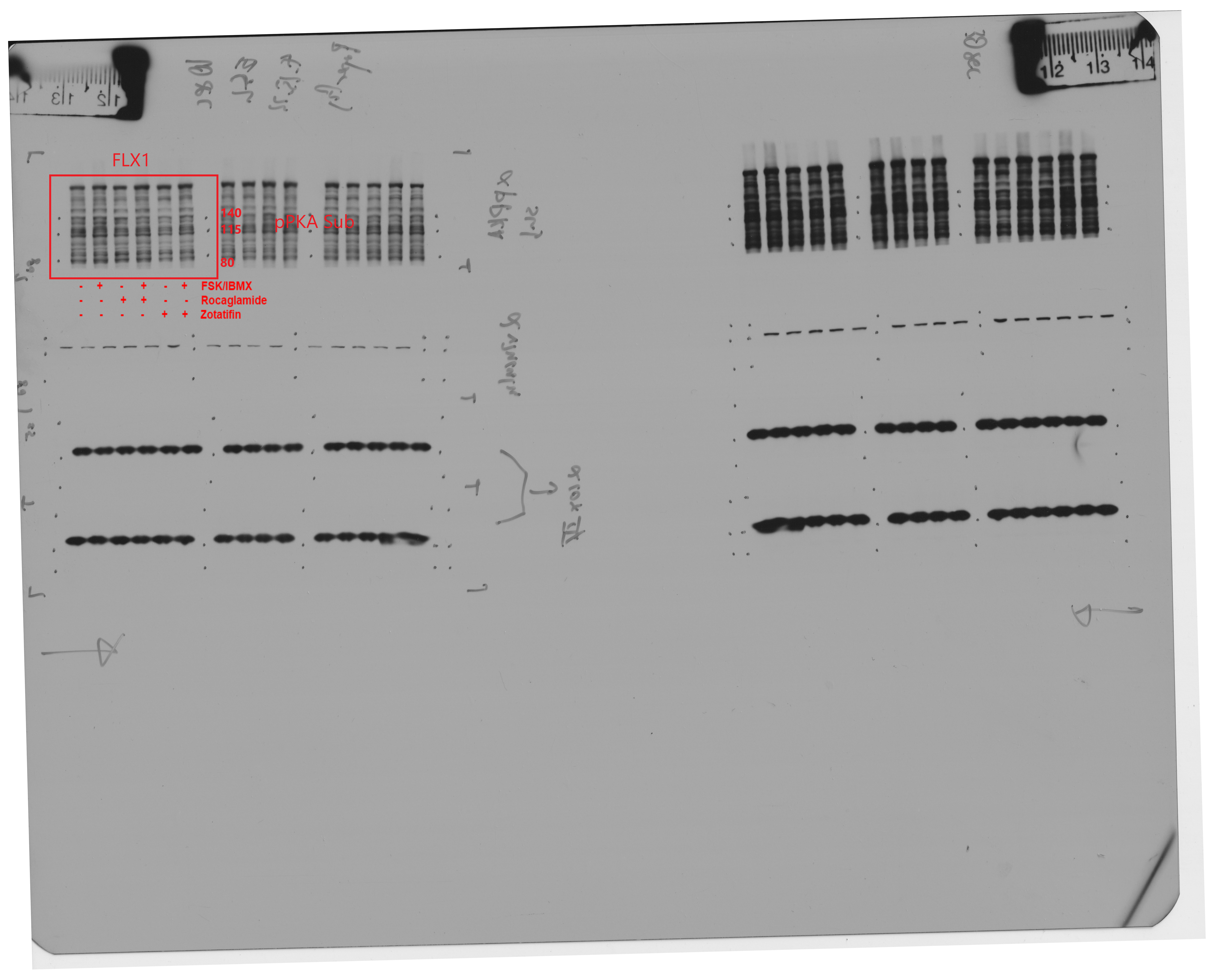

Supplement: Figure 7—source data 1. [file elife-69521-fig7-data1.zip › 7A/Figure 7A FLX1 pPKA-sub Labelled.tiff]

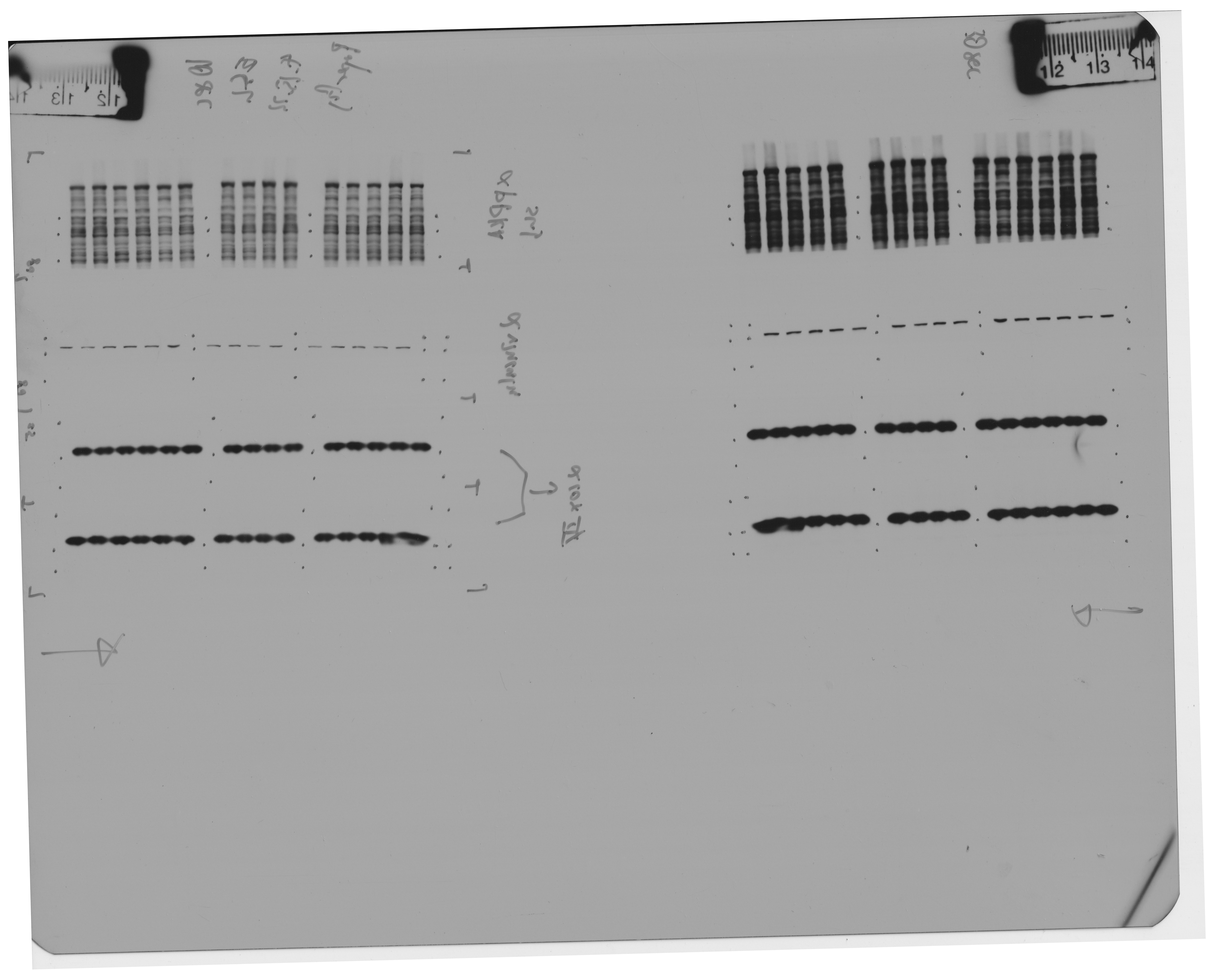

Supplement: Figure 7—source data 1. [file elife-69521-fig7-data1.zip › 7A/Figure 7A FLX1 pPKA-sub Raw.tiff]

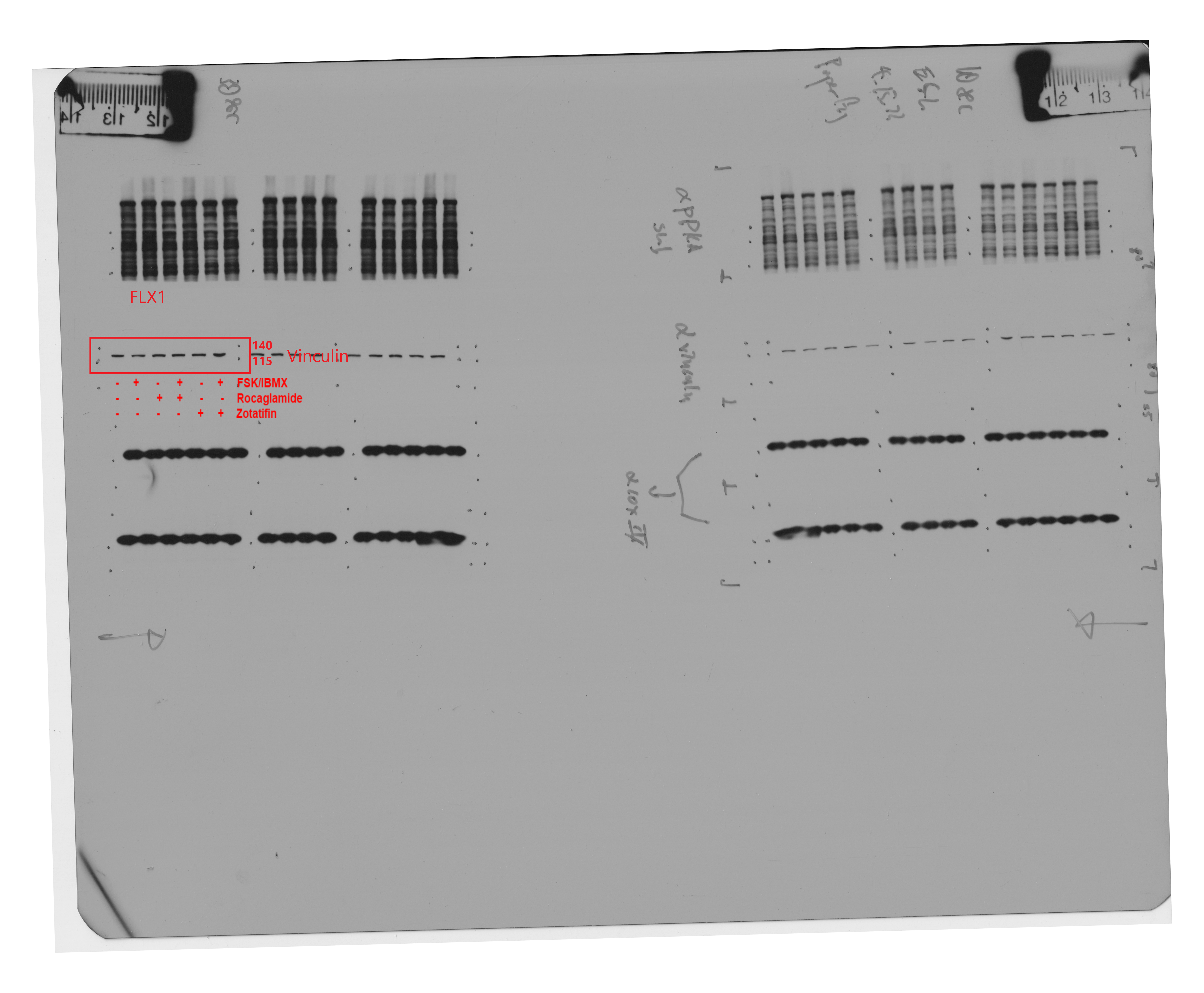

Supplement: Figure 7—source data 1. [file elife-69521-fig7-data1.zip › 7A/Figure 7A FLX1 Vinculin Labelled.tiff]

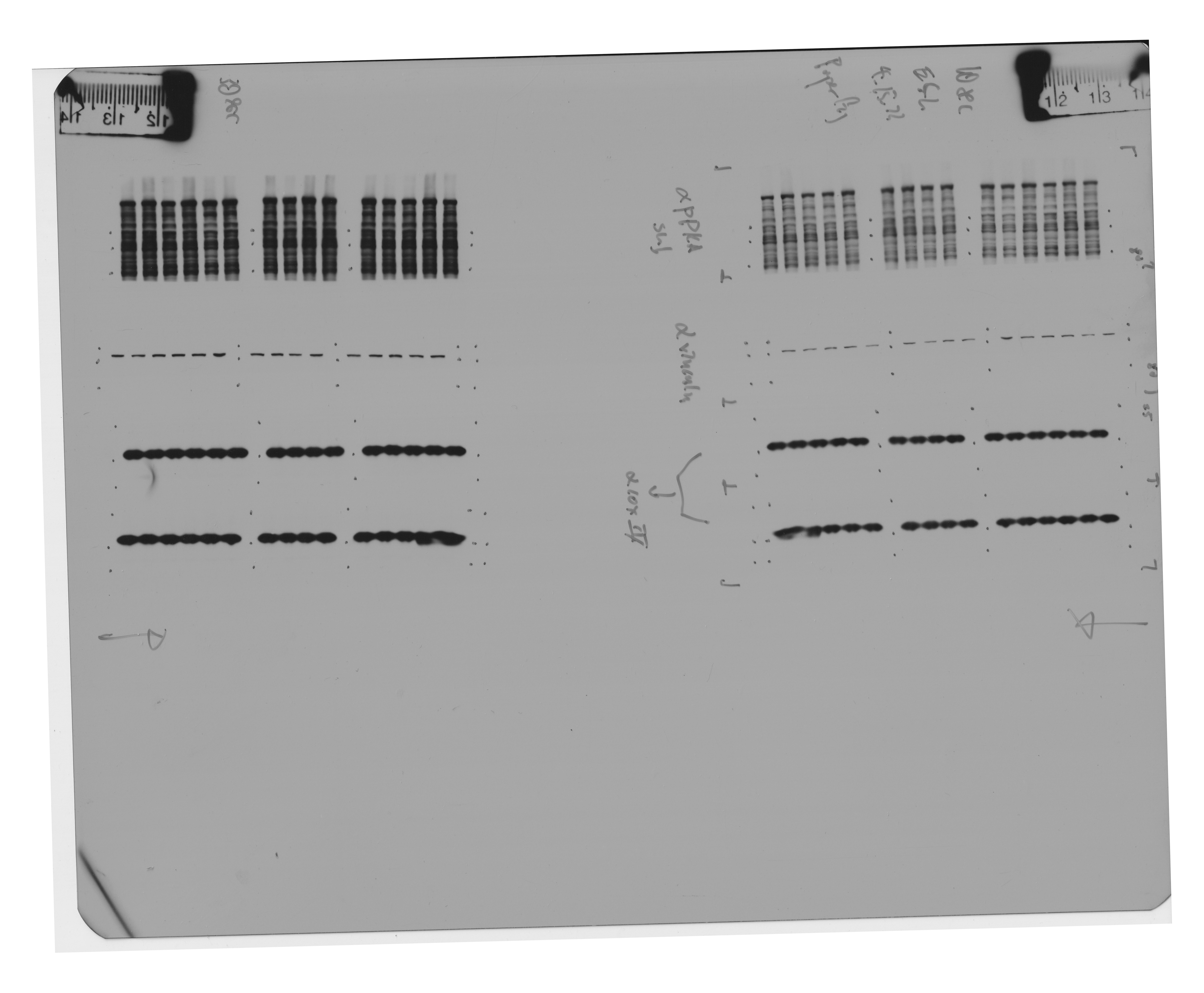

Supplement: Figure 7—source data 1. [file elife-69521-fig7-data1.zip › 7A/Figure 7A FLX1 Vinculin Raw.tiff]

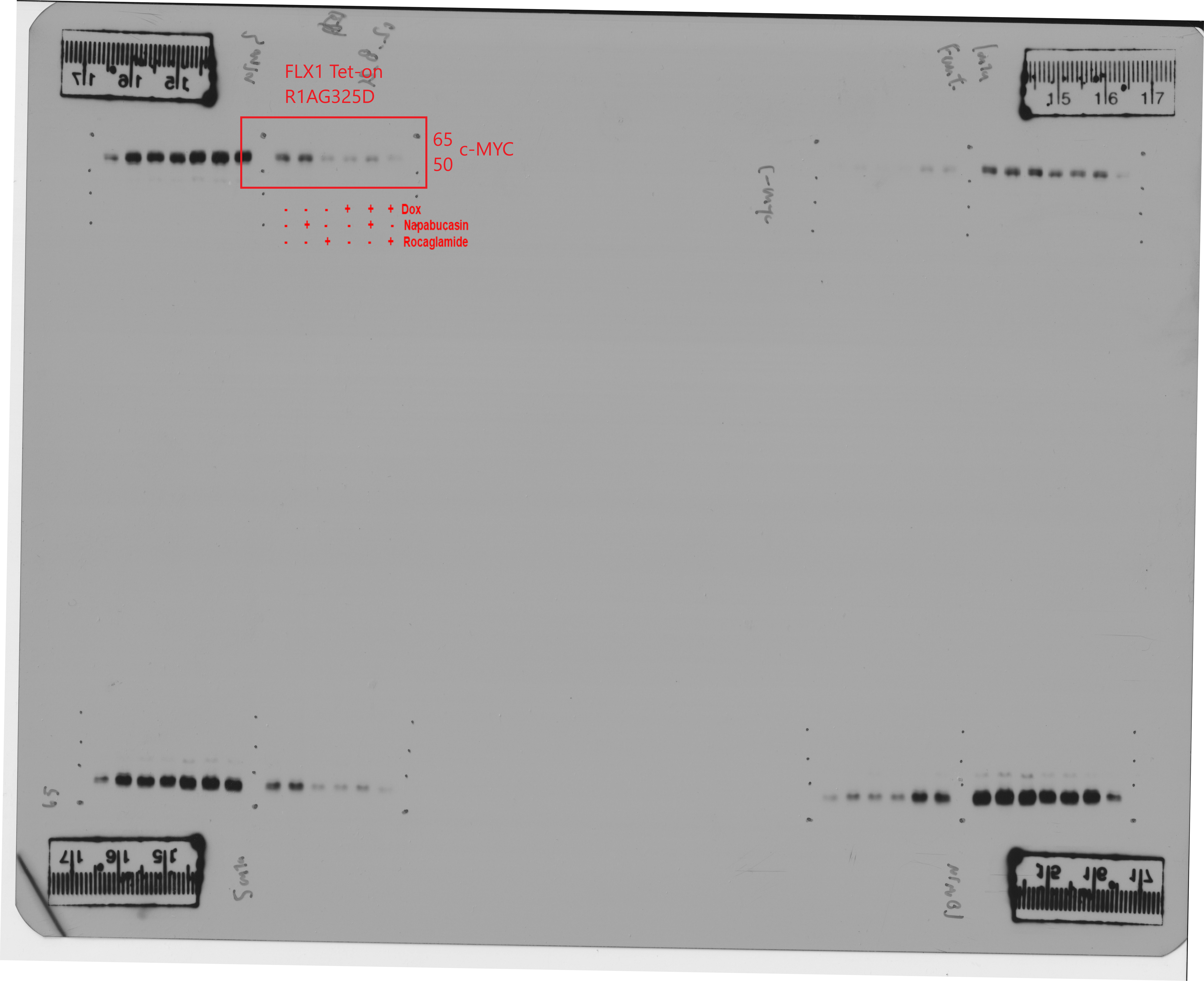

Supplement: Figure 7—source data 2. [file elife-69521-fig7-data2.zip › 7B/Figure 7B FLX1 R1A c-MYC Labelled.tiff]
