# Supplementary material for: Oncogenic PKA signaling increases c-MYC protein expression through multiple targetable mechanisms
Source: eLife. 2023 Jan 24;12:e69521. doi: 10.7554/eLife.69521 (PMC9925115; doi:10.7554/eLife.69521)
Supplement: Figure 2—source data 6. [file elife-69521-fig2-data6.zip › 2F/Fig2 marked up.pdf]

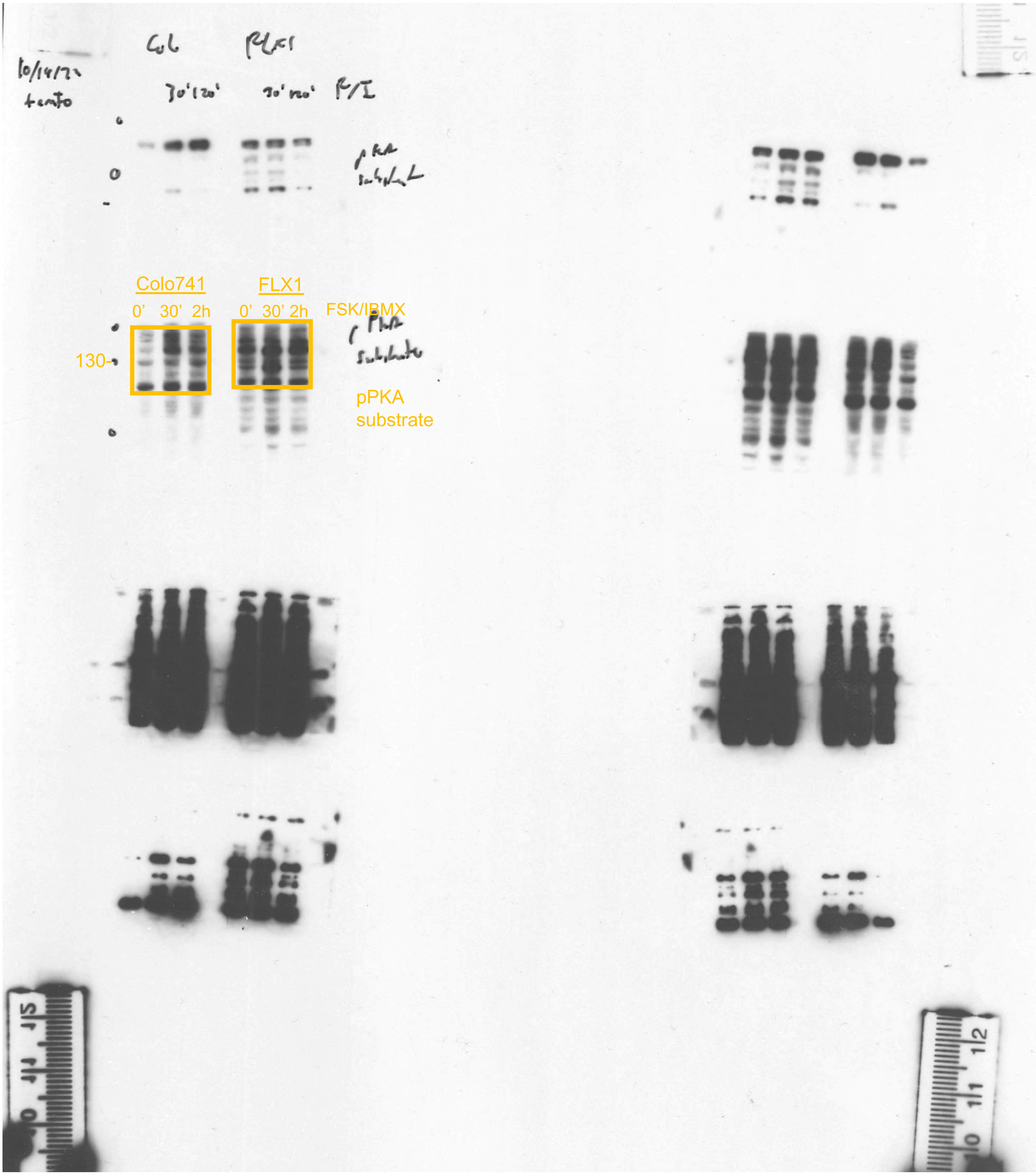
Colo741
FLX1
0' 30' 2h
0' 30' 2h
FSK/IBMX
130-
pPKA
substrate

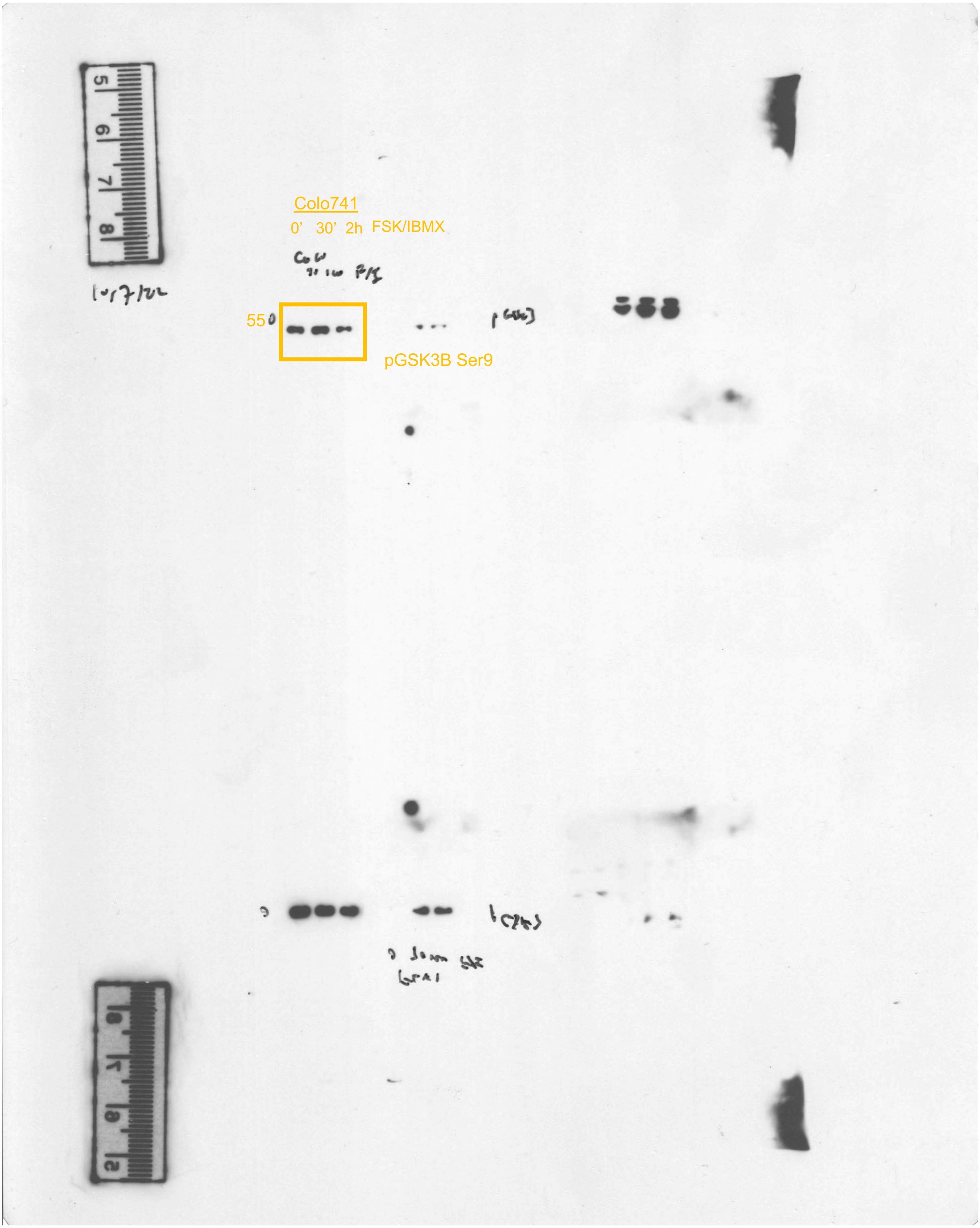
Colo741
0’ 30’ 2h FSK/IBMX
55
pGSK3B Ser9

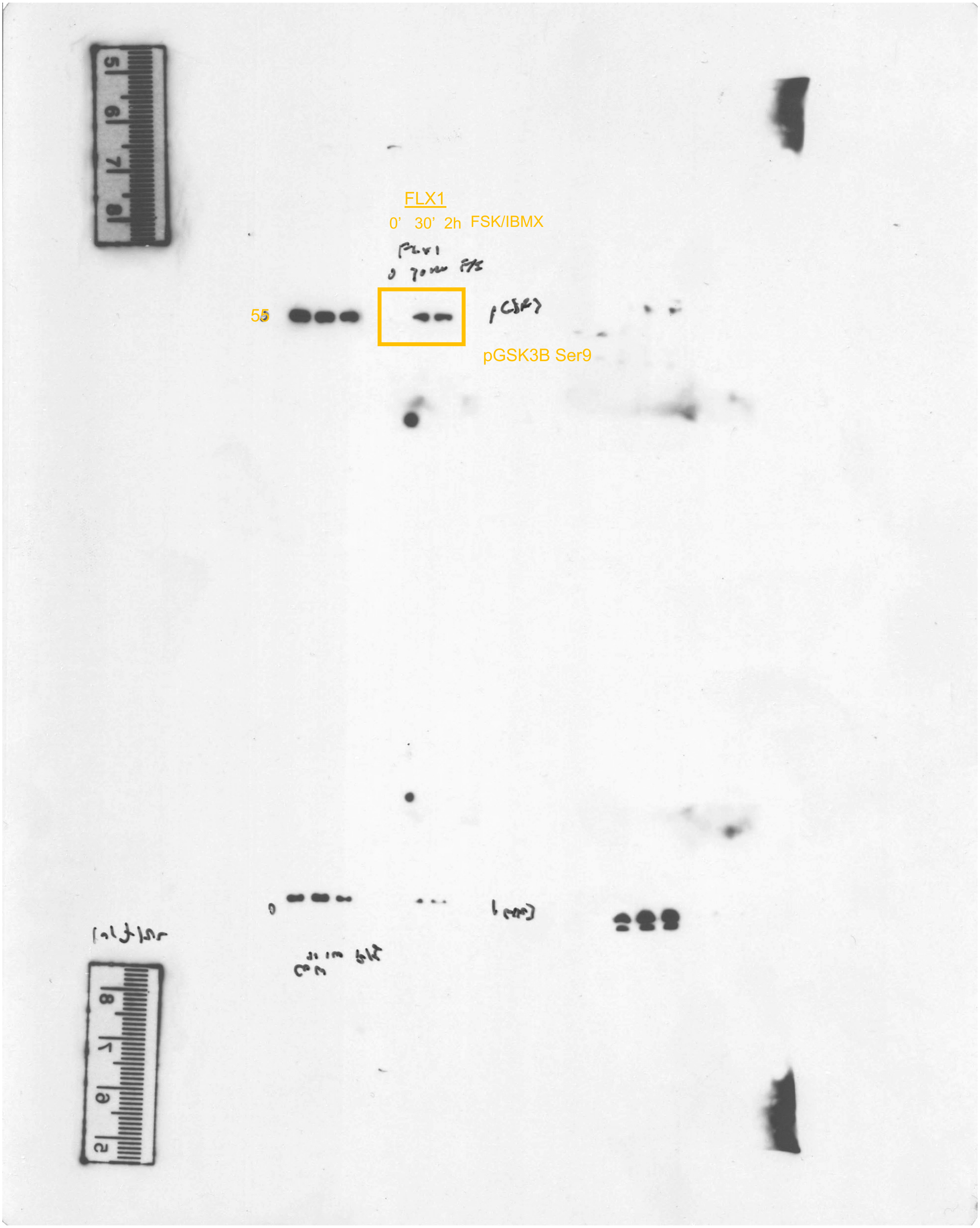

FLX1
0'
30'
2h
FSK/IBMX
55
pGSK3B Ser9

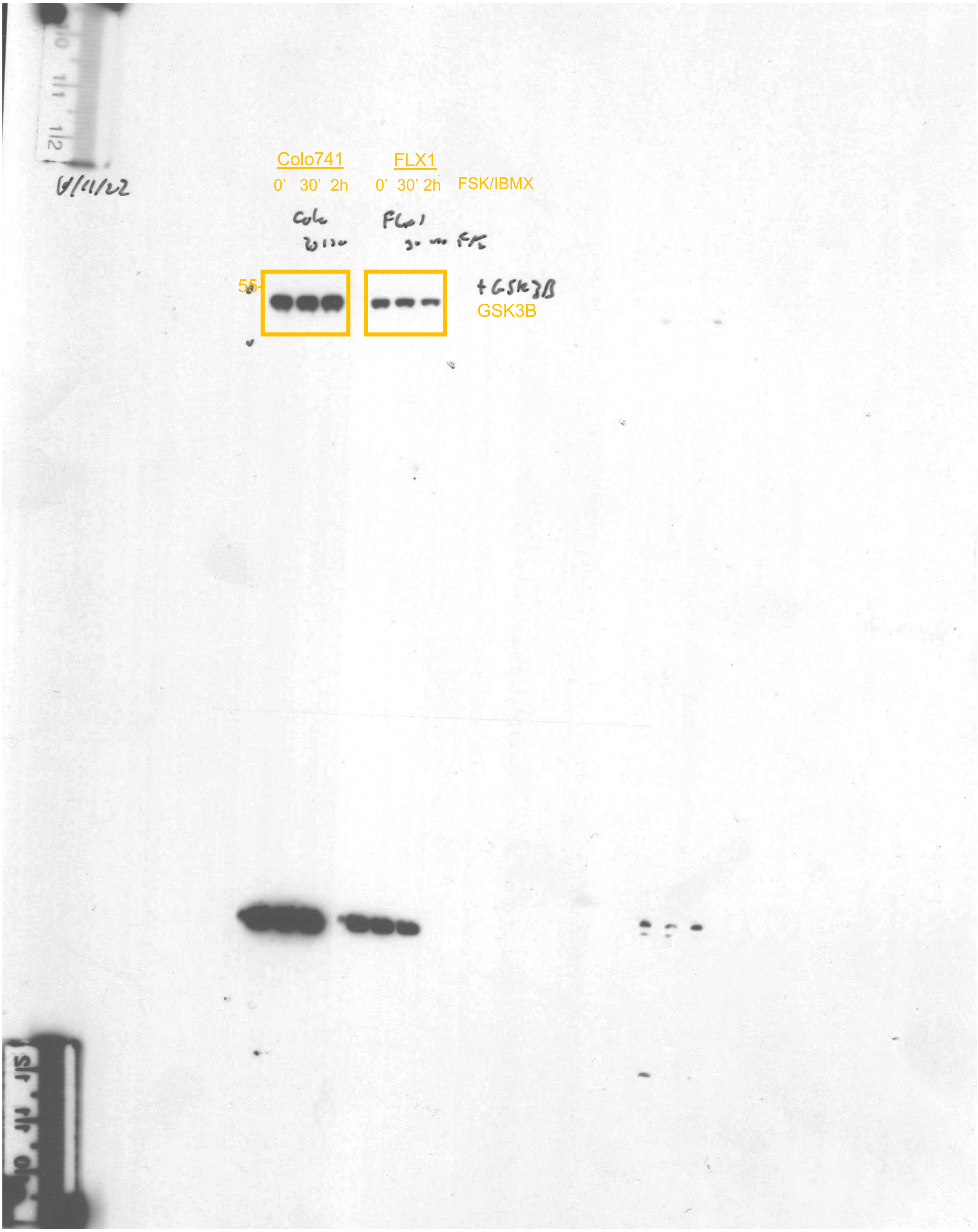
Colo741
0' 30' 2h
FLX1
0' 30' 2h
FSK/IBMX
55
GSK3B

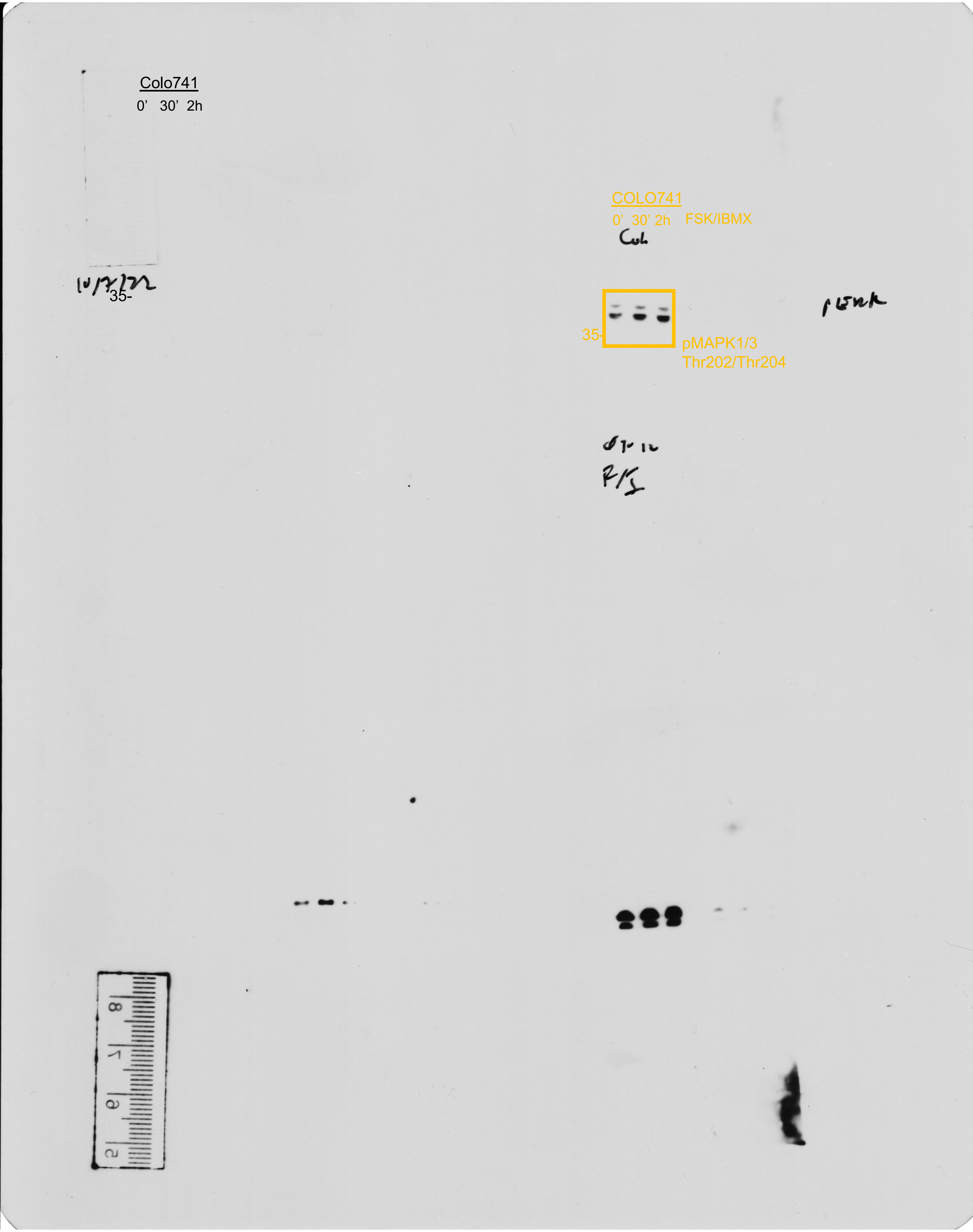
Colo741
0’ 30’ 2h
35-
COLO741
0’ 30’ 2h FSK/IBMX
35-
pMAPK1/3
Thr202/Thr204

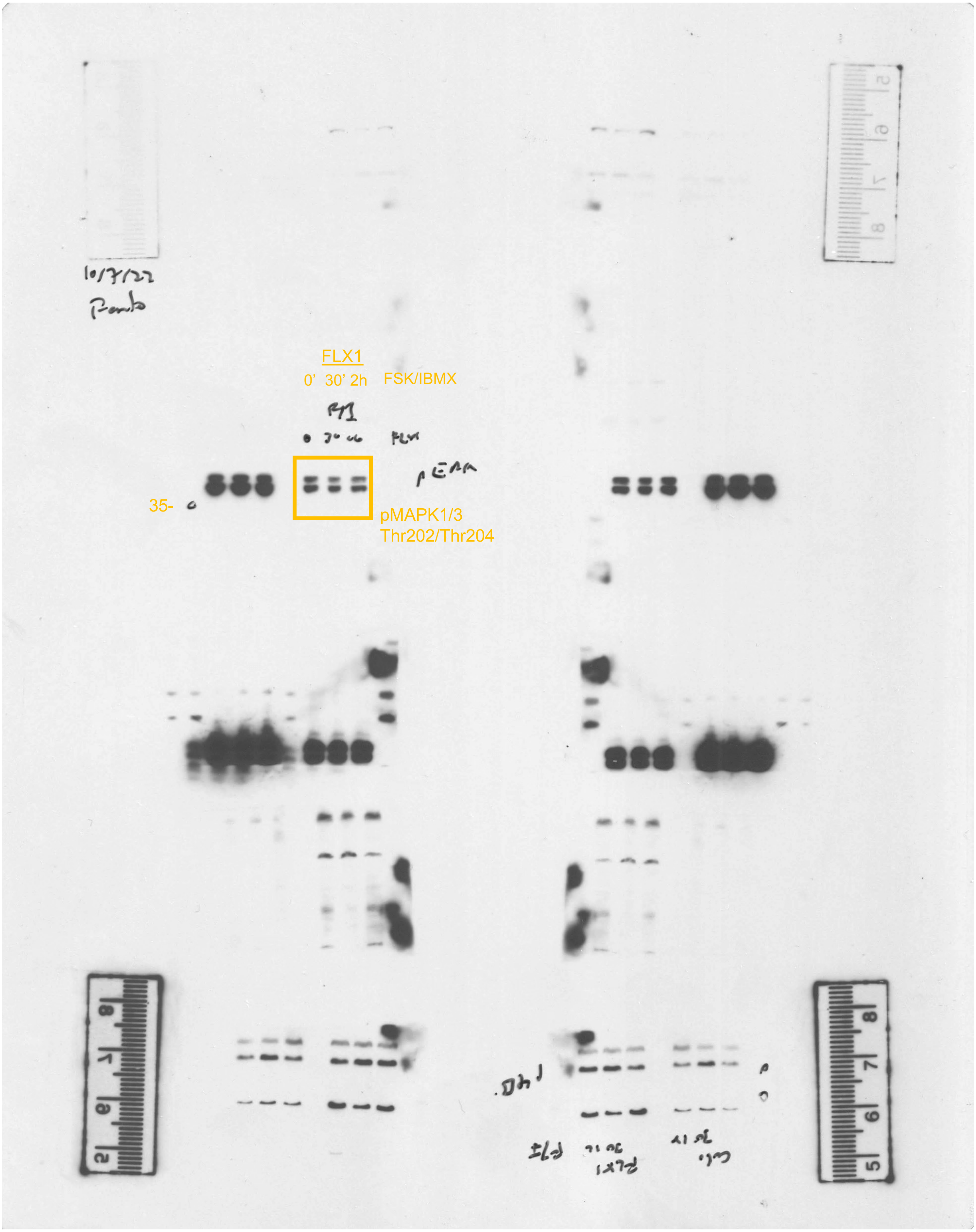
FLX1
0' 30' 2h
FSK/IBMX
35-
pMAPK1/3
Thr202/Thr204

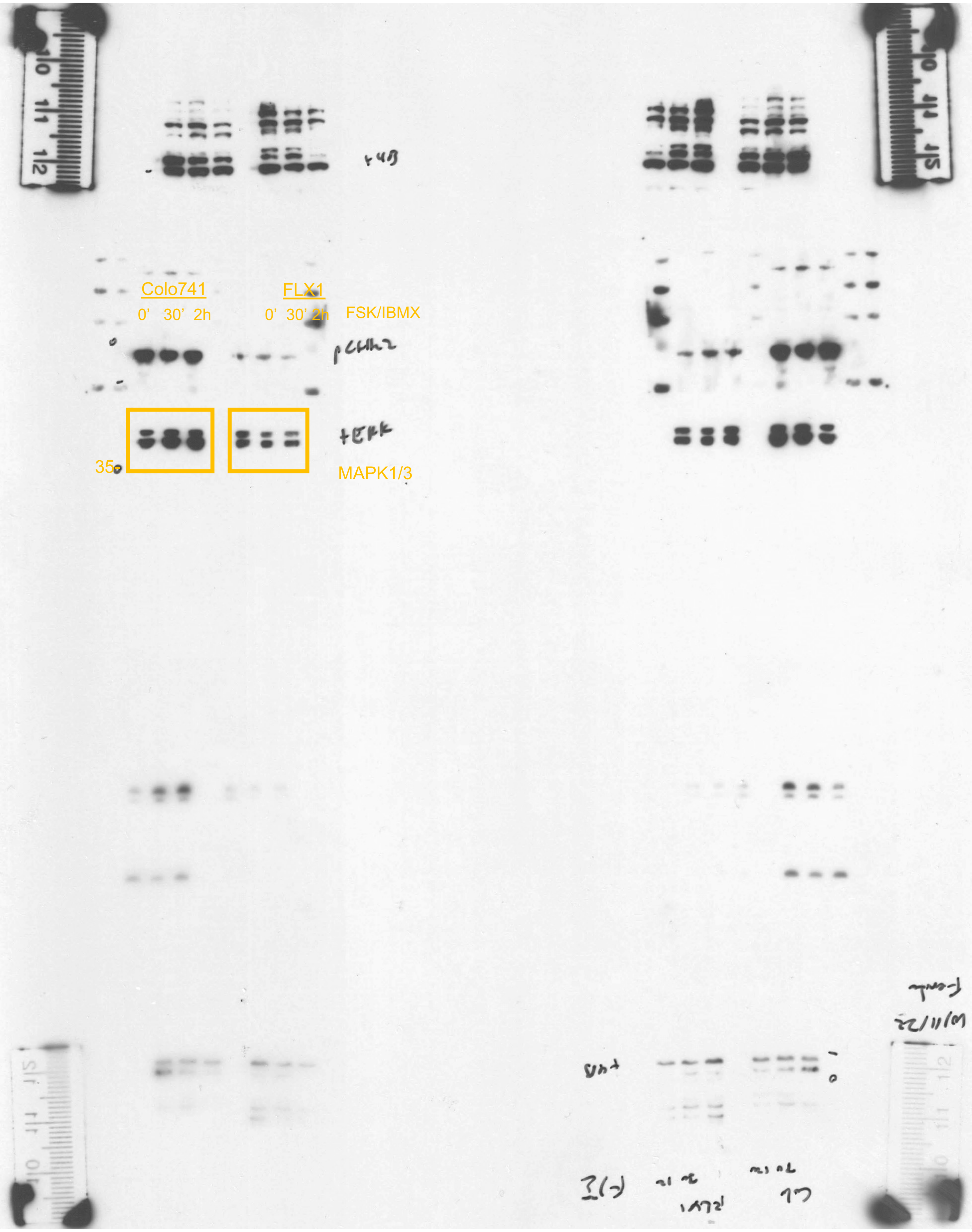
Colo741
FLX1
0' 30' 2h
0' 30' 2h
FSK/IBMX
35
MAPK1/3

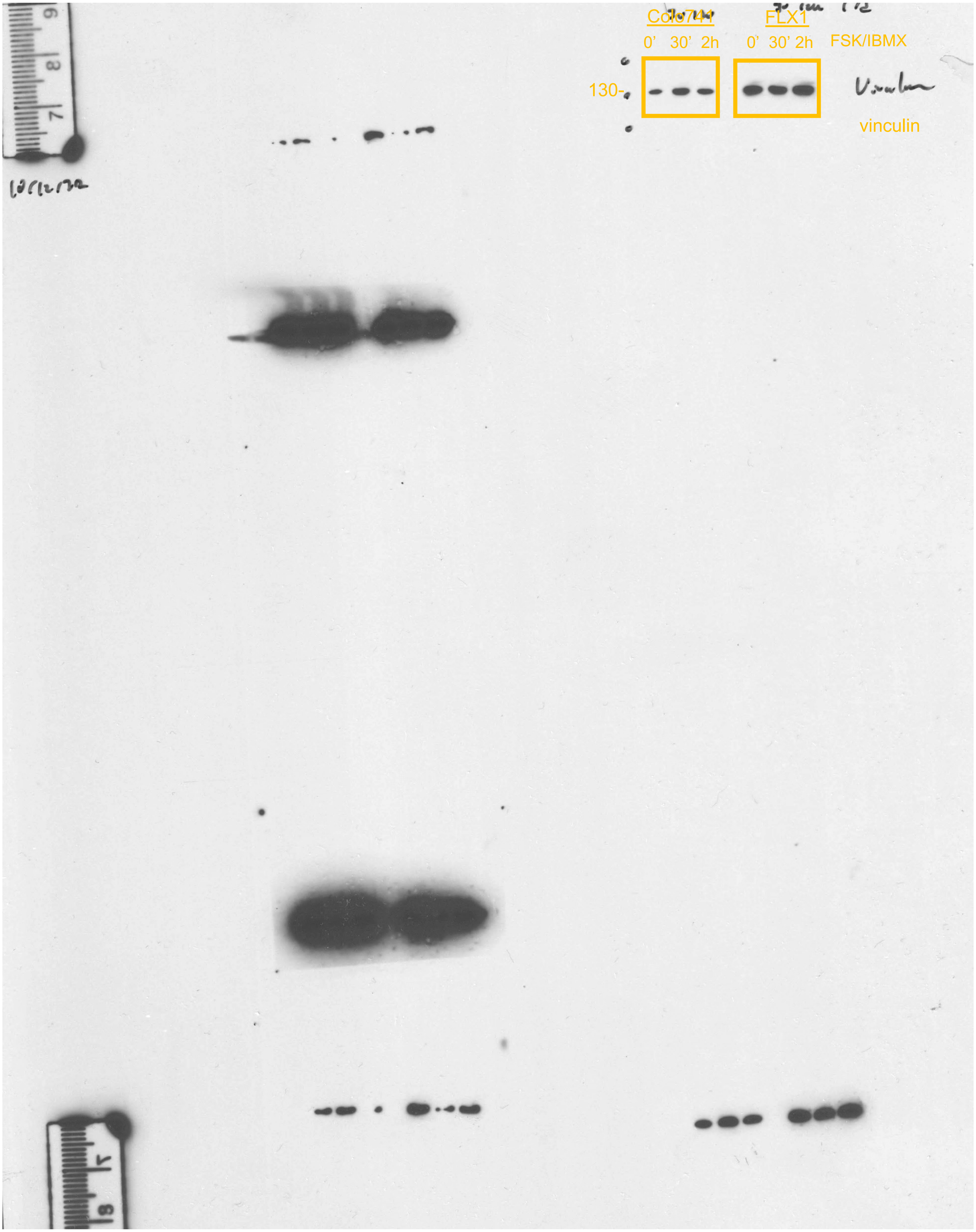
Colo741
FLX1
0' 30' 2h
0' 30' 2h
FSK/IBMX
130-
vinculin
