# Supplementary material for: Oncogenic PKA signaling increases c-MYC protein expression through multiple targetable mechanisms
Source: eLife. 2023 Jan 24;12:e69521. doi: 10.7554/eLife.69521 (PMC9925115; doi:10.7554/eLife.69521)
Supplement: Figure 3—source data 5. [file elife-69521-fig3-data5.zip › 3E/3E markup.pdf]

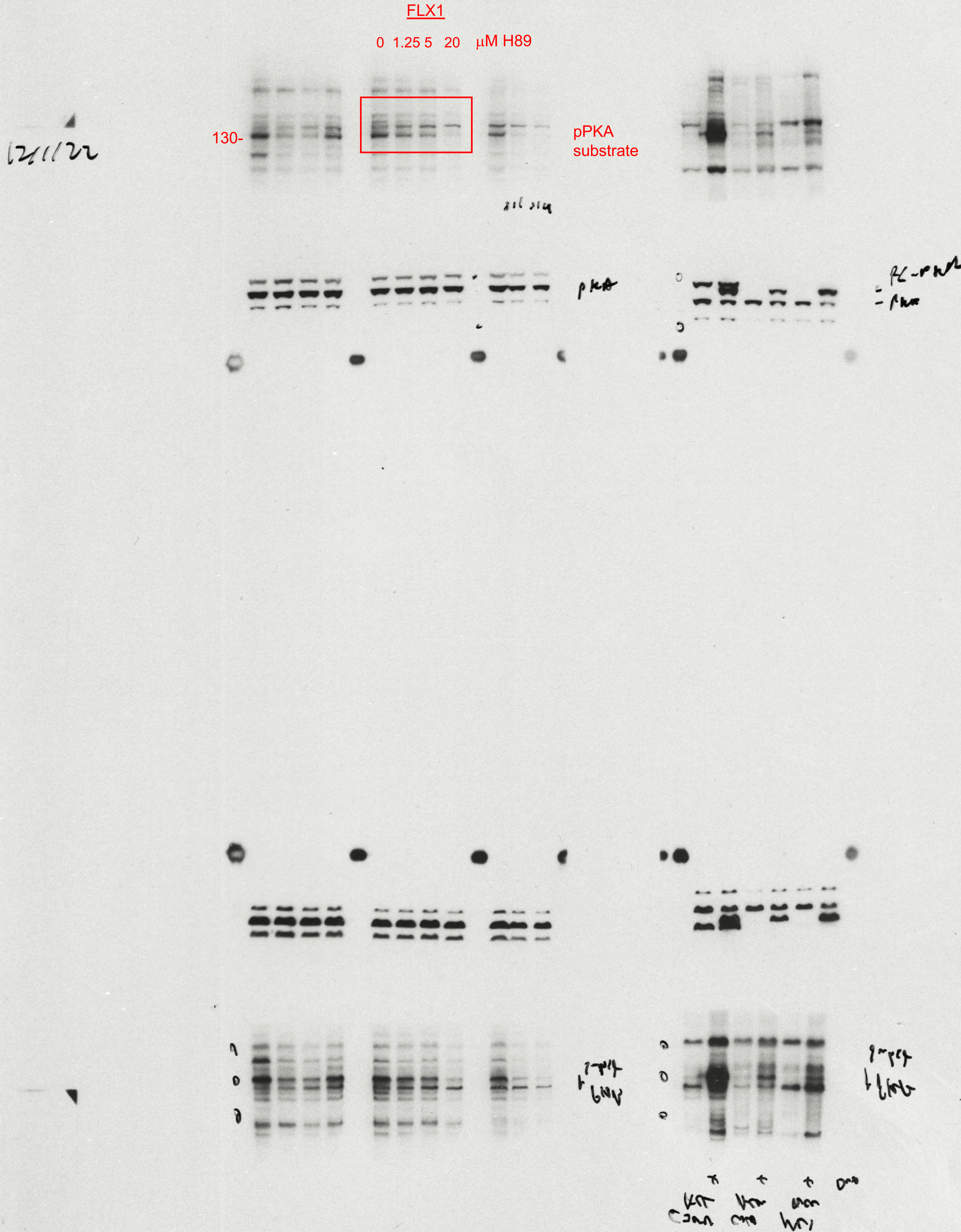
12/11/22
FLX1
0 1.25 5 20 µM H89
130-
pPKA
substrate
pKA

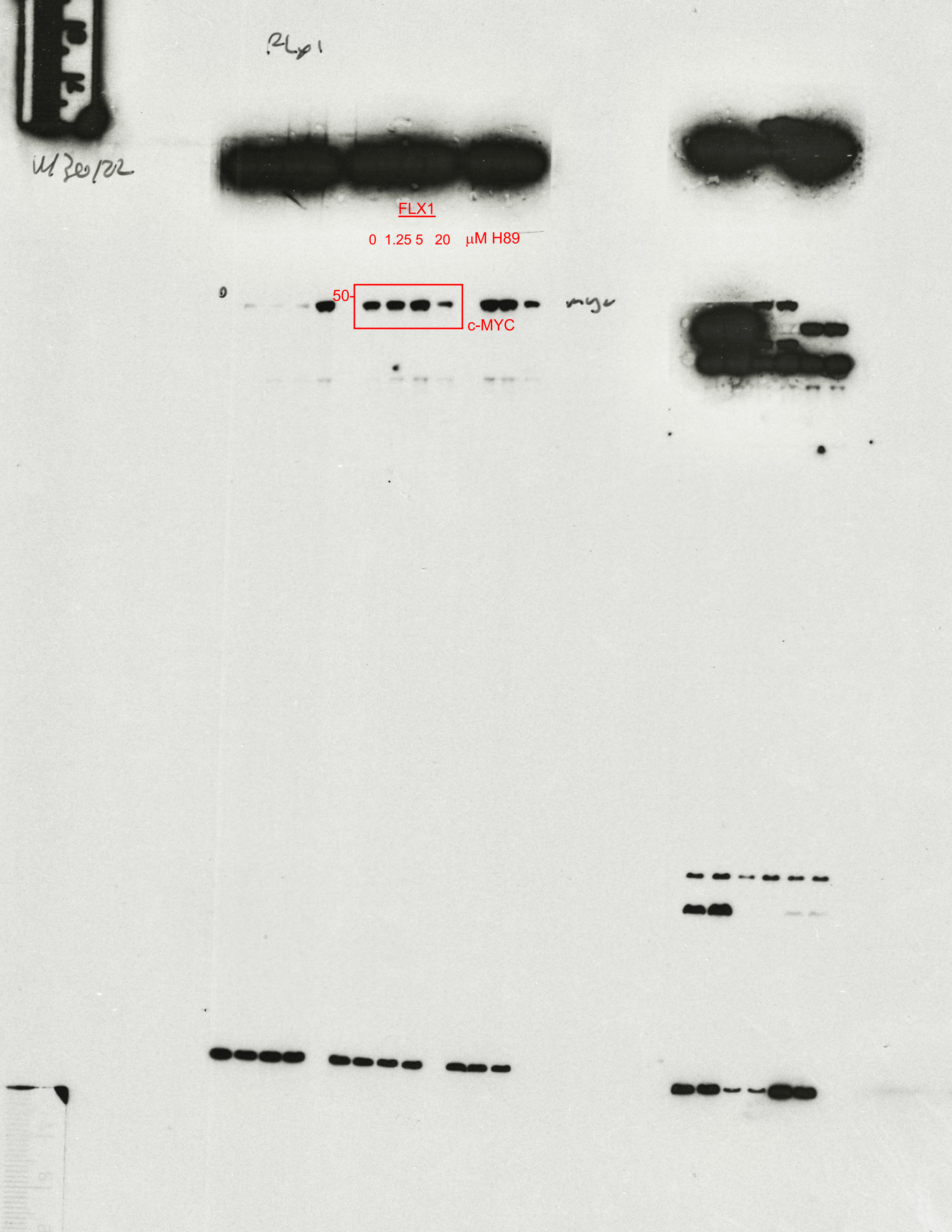
FLX1
0 1.25 5 20 μM H89
50-
c-MYC

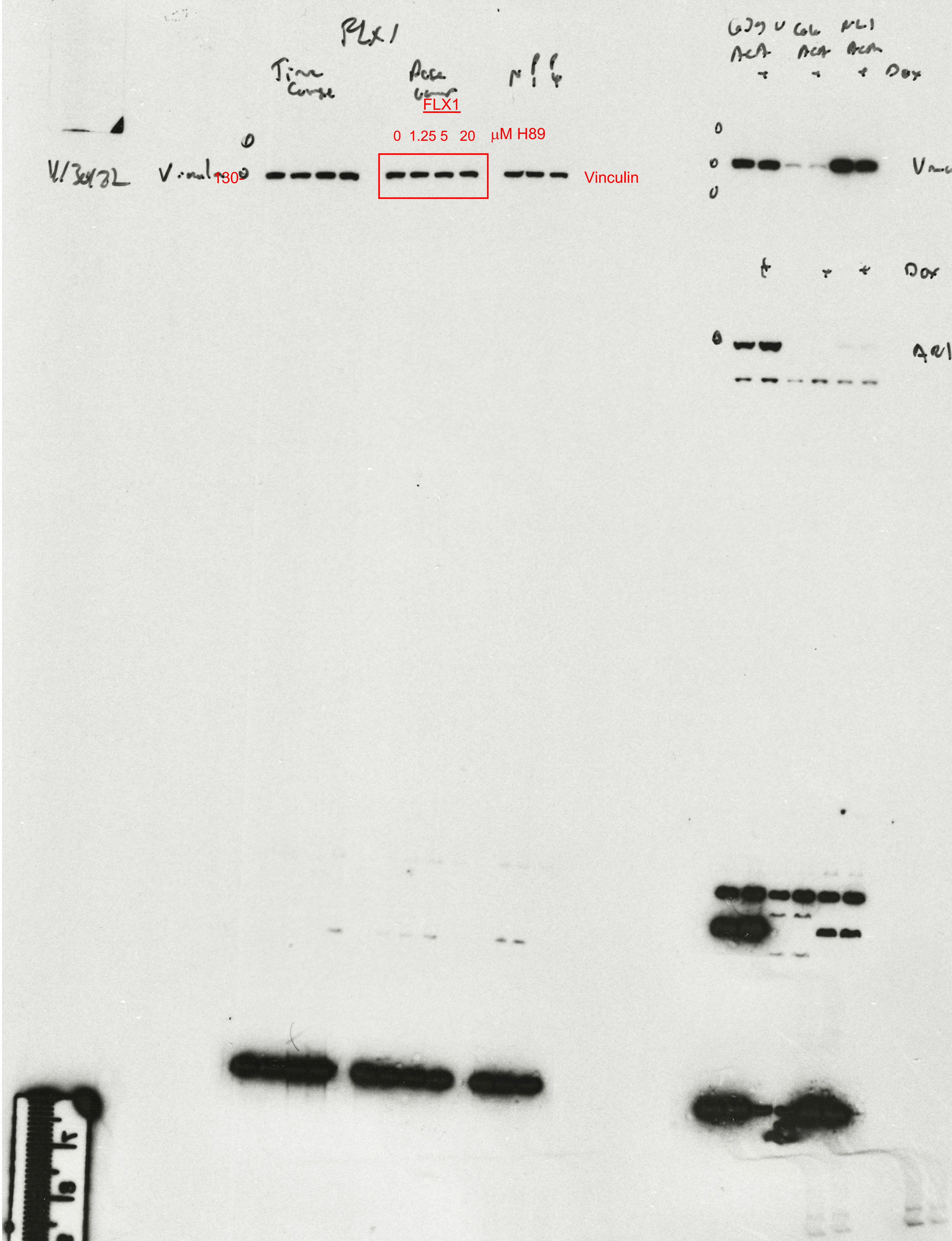
FLX1
Time Course
Dose
FLX1
0 1.25 5 20 µM H89
130-
Vinculin
Dox
Dox
