# Supplementary material for: Oncogenic PKA signaling increases c-MYC protein expression through multiple targetable mechanisms
Source: eLife. 2023 Jan 24;12:e69521. doi: 10.7554/eLife.69521 (PMC9925115; doi:10.7554/eLife.69521)
Supplement: Figure 6—source data 2. [file elife-69521-fig6-data2.zip › 6B/6B markup.pdf]

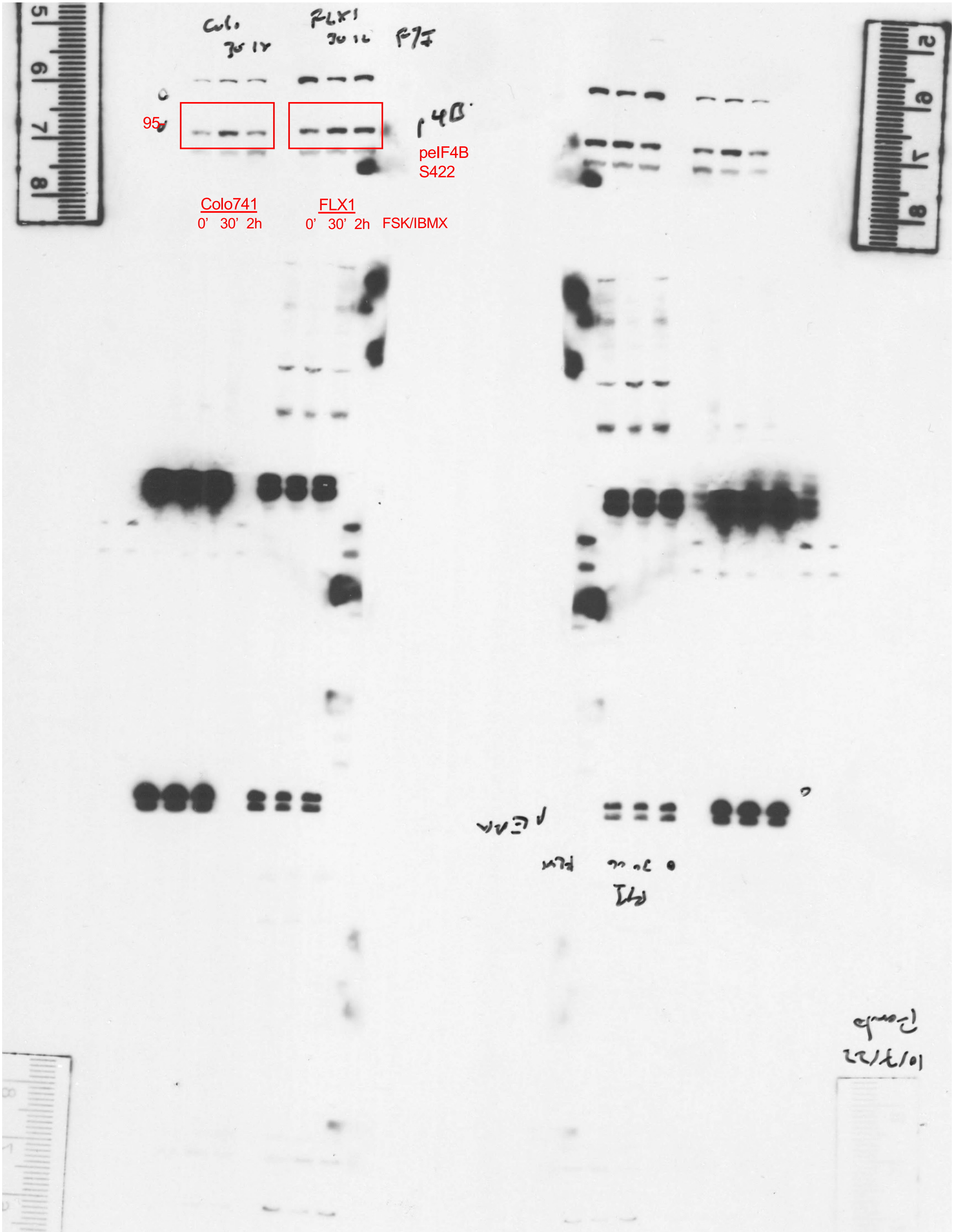
95
peIF4B
S422
Colo741
0' 30' 2h
FLX1
0' 30' 2h
FSK/IBMX

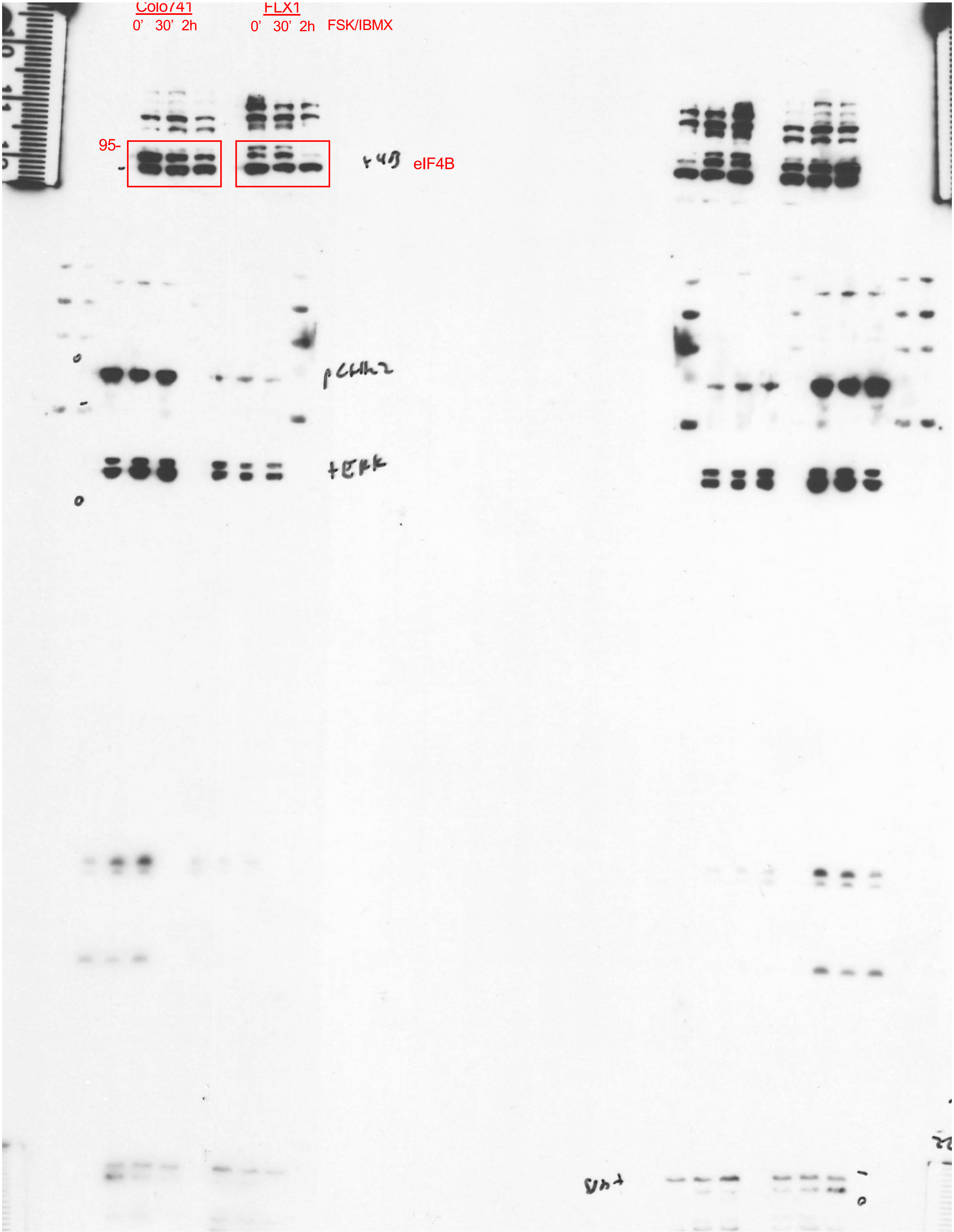
Colo741
0' 30' 2h
FLX1
0' 30' 2h FSK/IBMX
95-
eIF4B

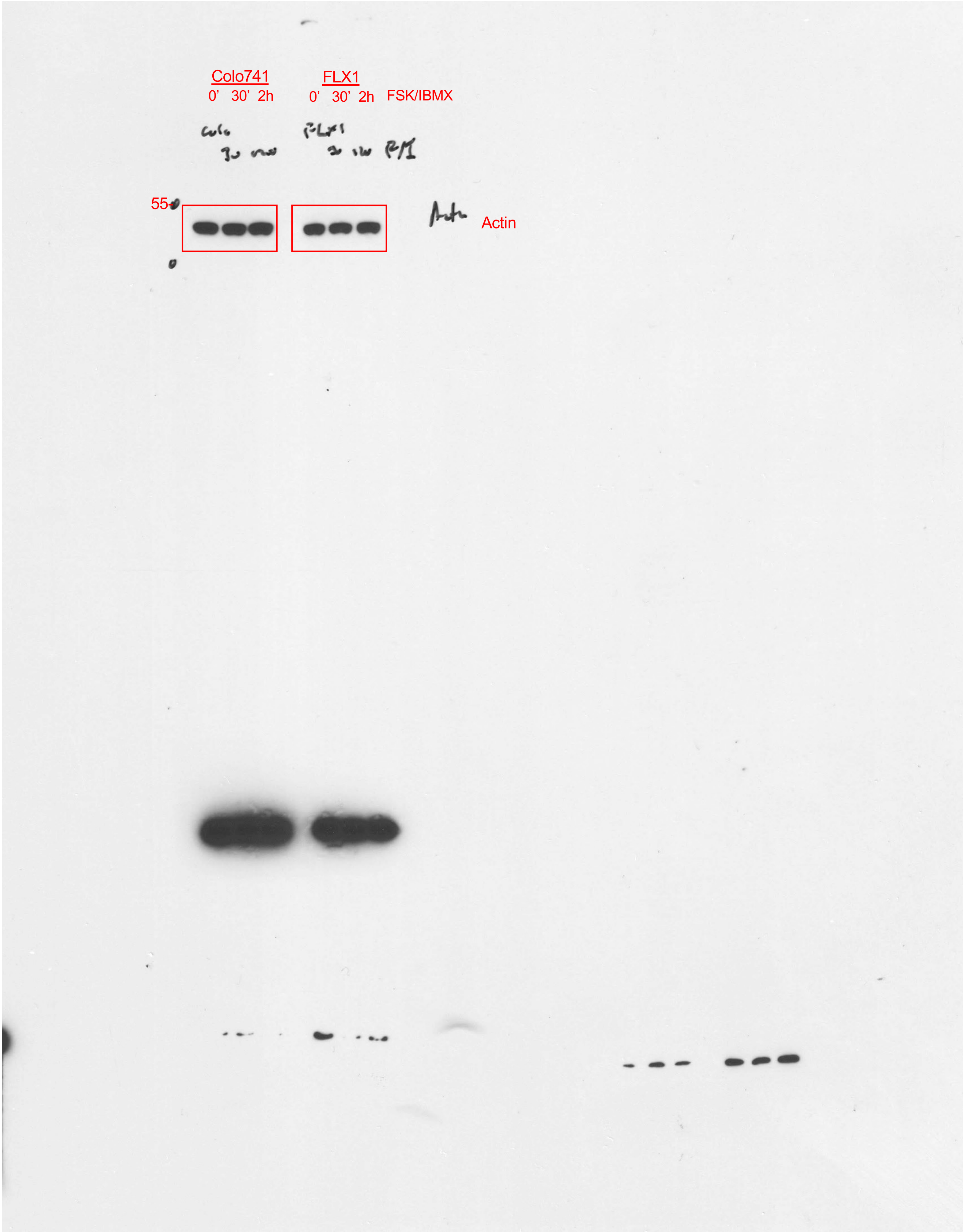
Colo741
0' 30' 2h
FLX1
0' 30' 2h
FSK/IBMX
55
Actin
