# Supplementary material for: Oncogenic PKA signaling increases c-MYC protein expression through multiple targetable mechanisms
Source: eLife. 2023 Jan 24;12:e69521. doi: 10.7554/eLife.69521 (PMC9925115; doi:10.7554/eLife.69521)
Supplement: Figure 6—source data 3. [file elife-69521-fig6-data3.zip › 6C/6c markup.pdf]

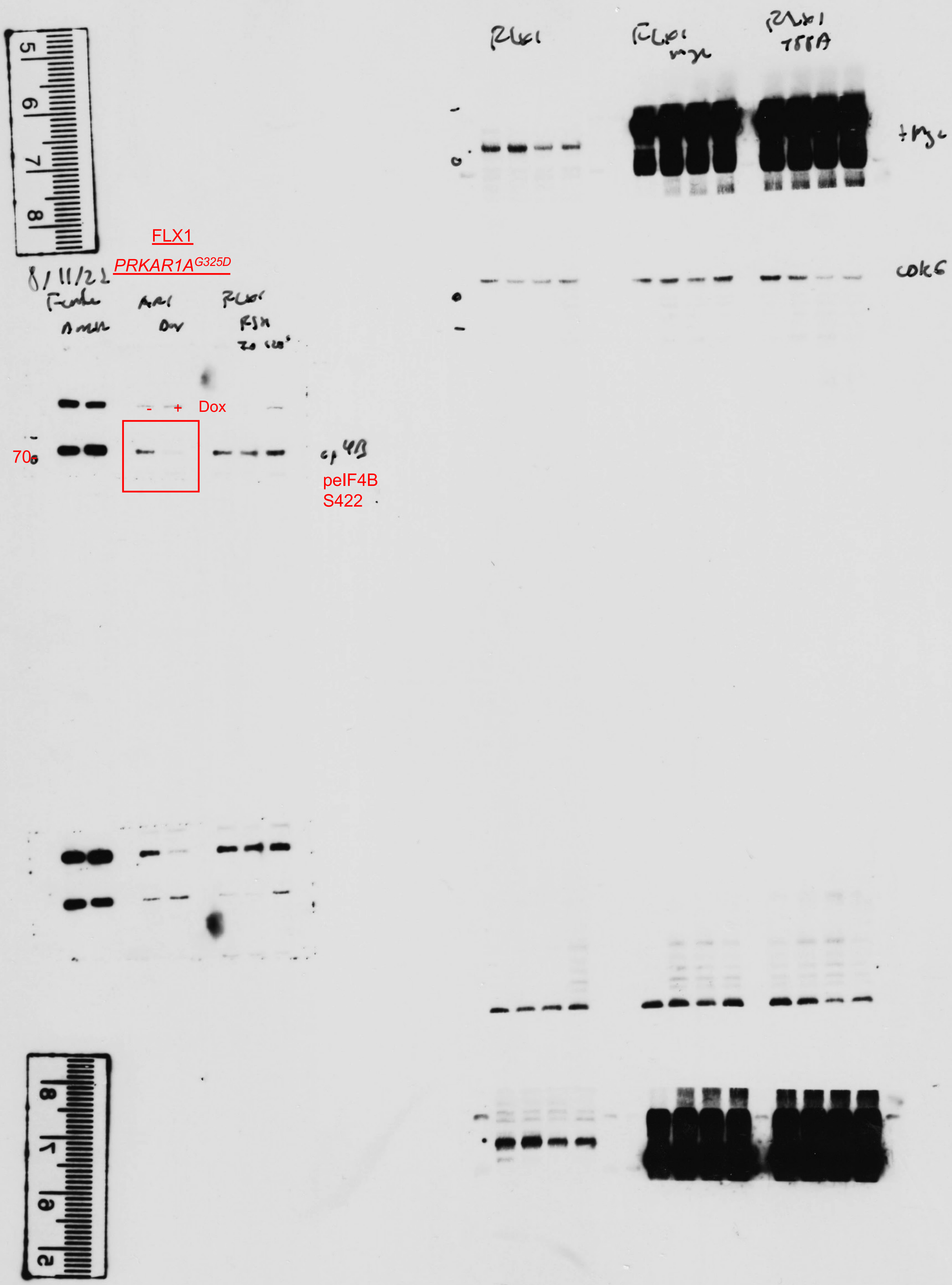
FLX1
PRKAR1A G325D
- + Dox
70
peIF4B
S422

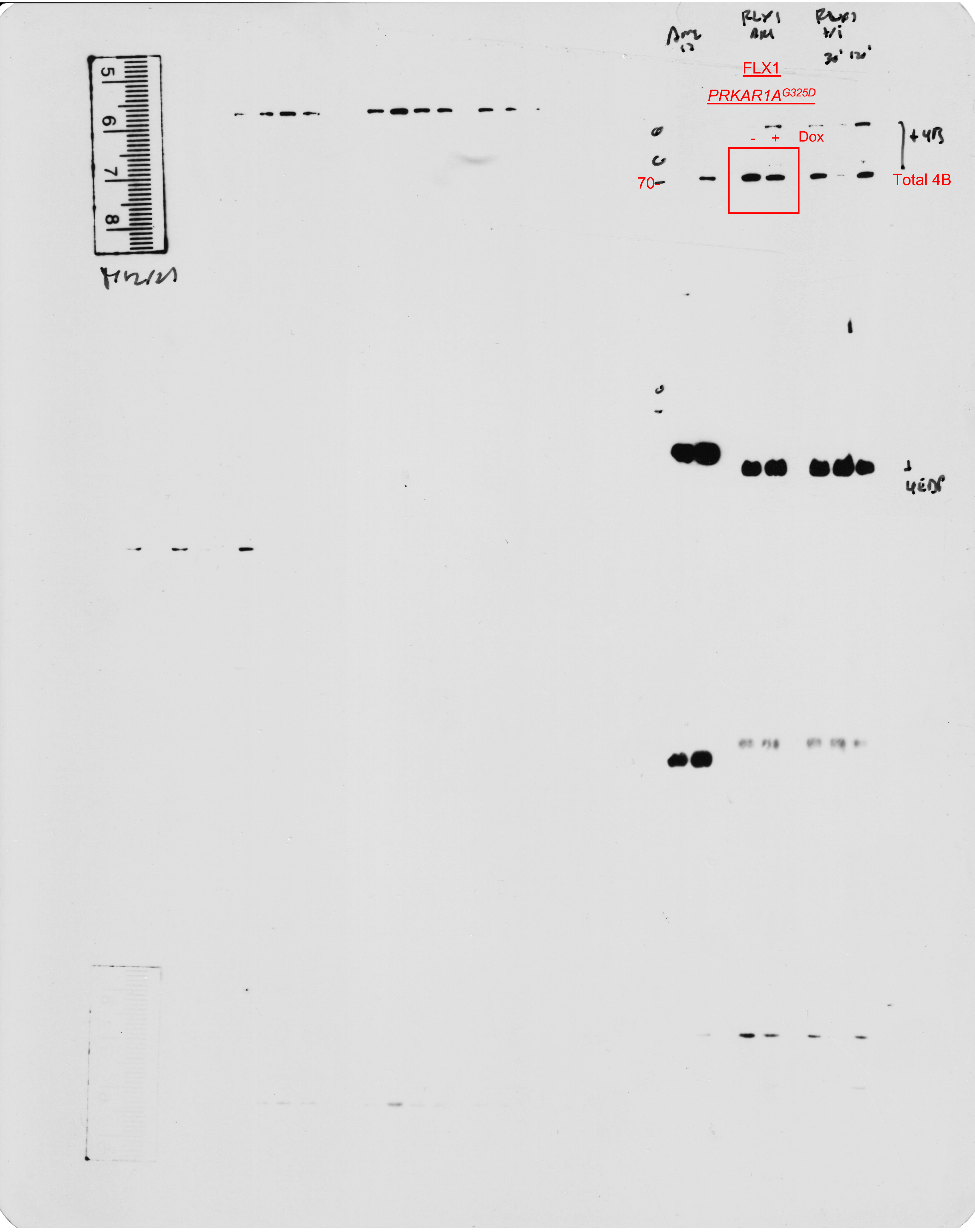

FLX1
PRKAR1A^G325D
- + Dox
70-
Total 4B

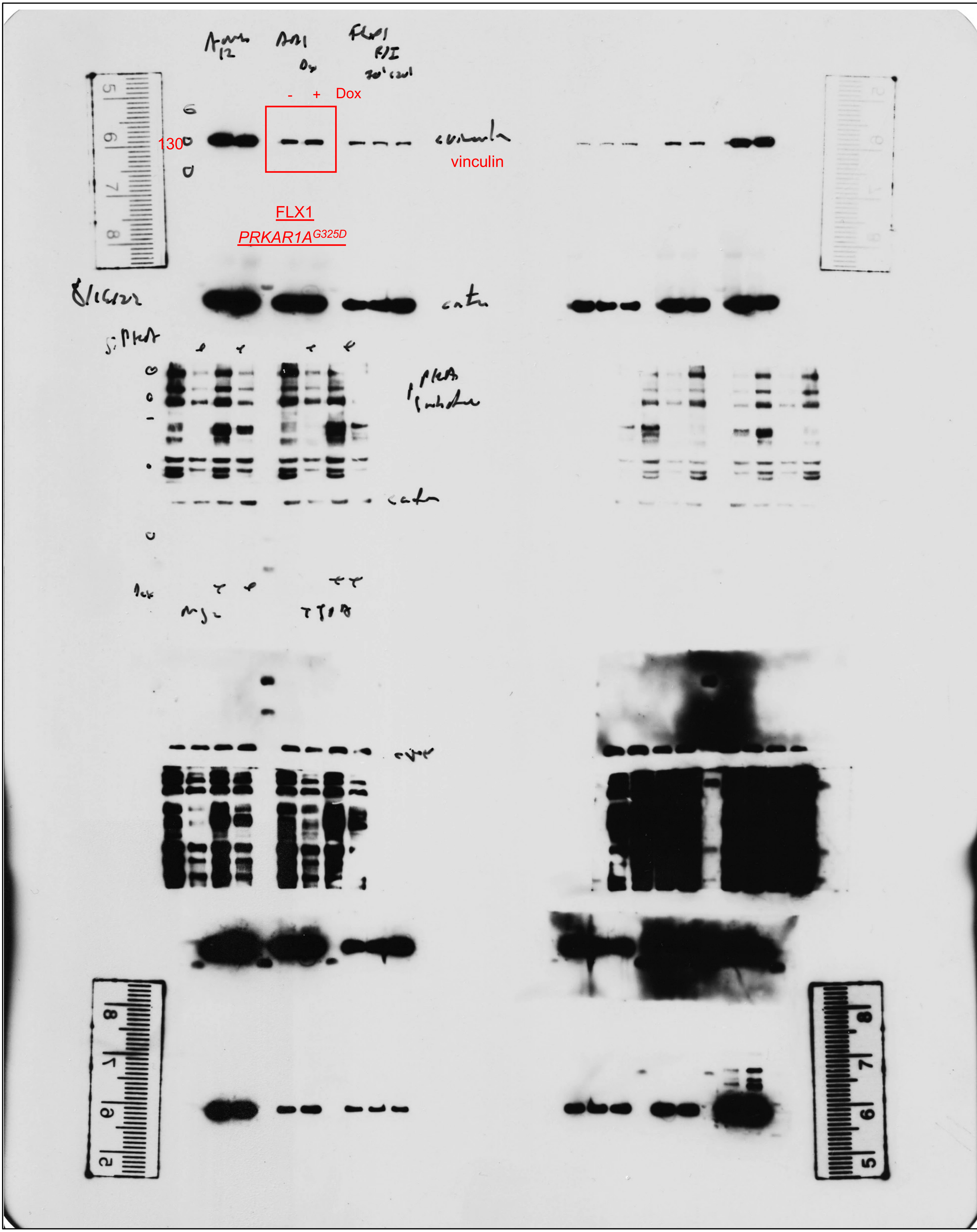
- + Dox
130
vinculin
FLX1
PRKAR1A G325D
