# Supplementary figures and images for: Oncogenic PKA signaling increases c-MYC protein expression through multiple targetable mechanisms (part 2 of 2)
Source: eLife. 2023 Jan 24;12:e69521. doi: 10.7554/eLife.69521 (PMC9925115; doi:10.7554/eLife.69521)

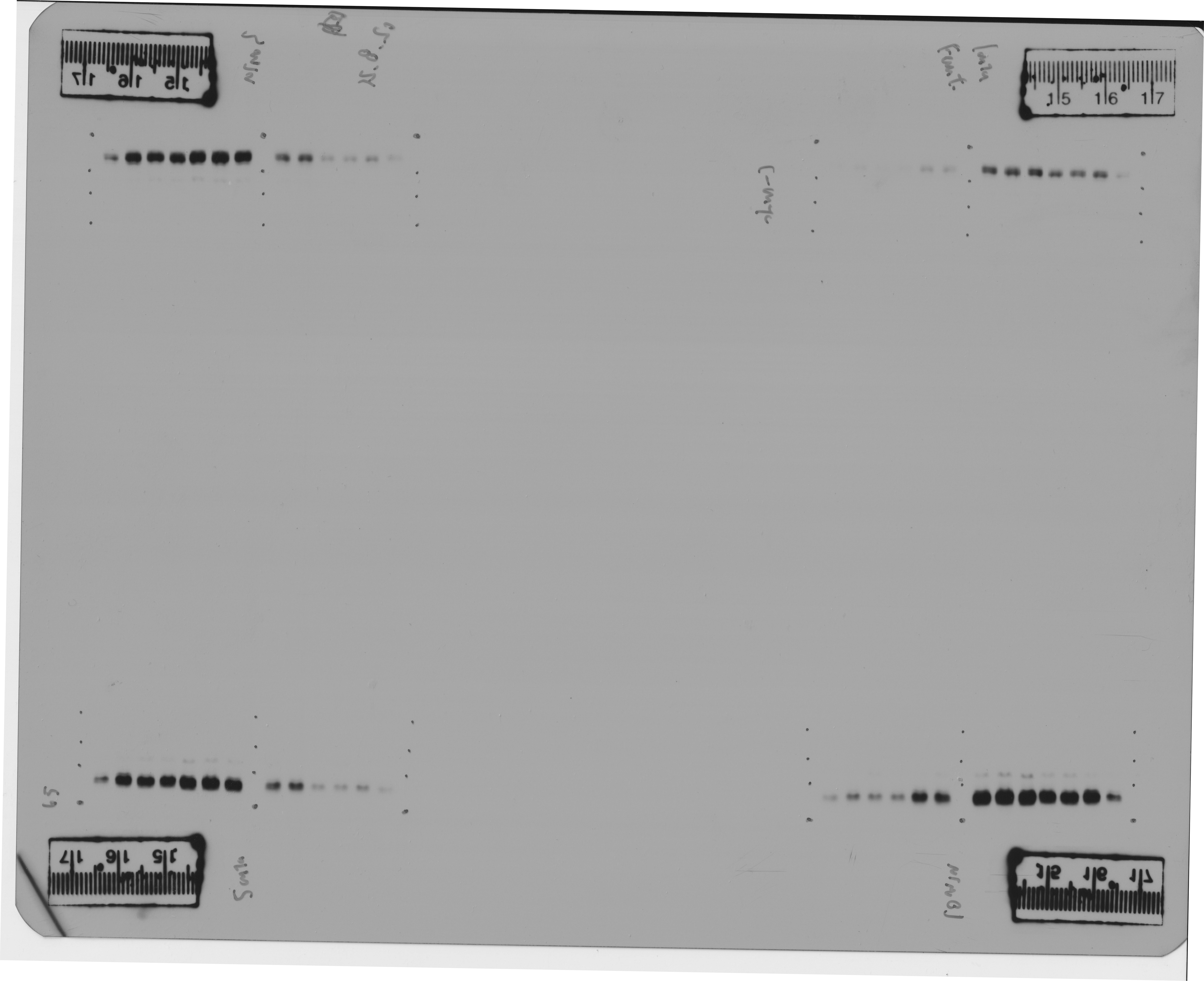

Supplement: Figure 7—source data 2. [file elife-69521-fig7-data2.zip › 7B/Figure 7B FLX1 R1A c-MYC Raw.tiff]

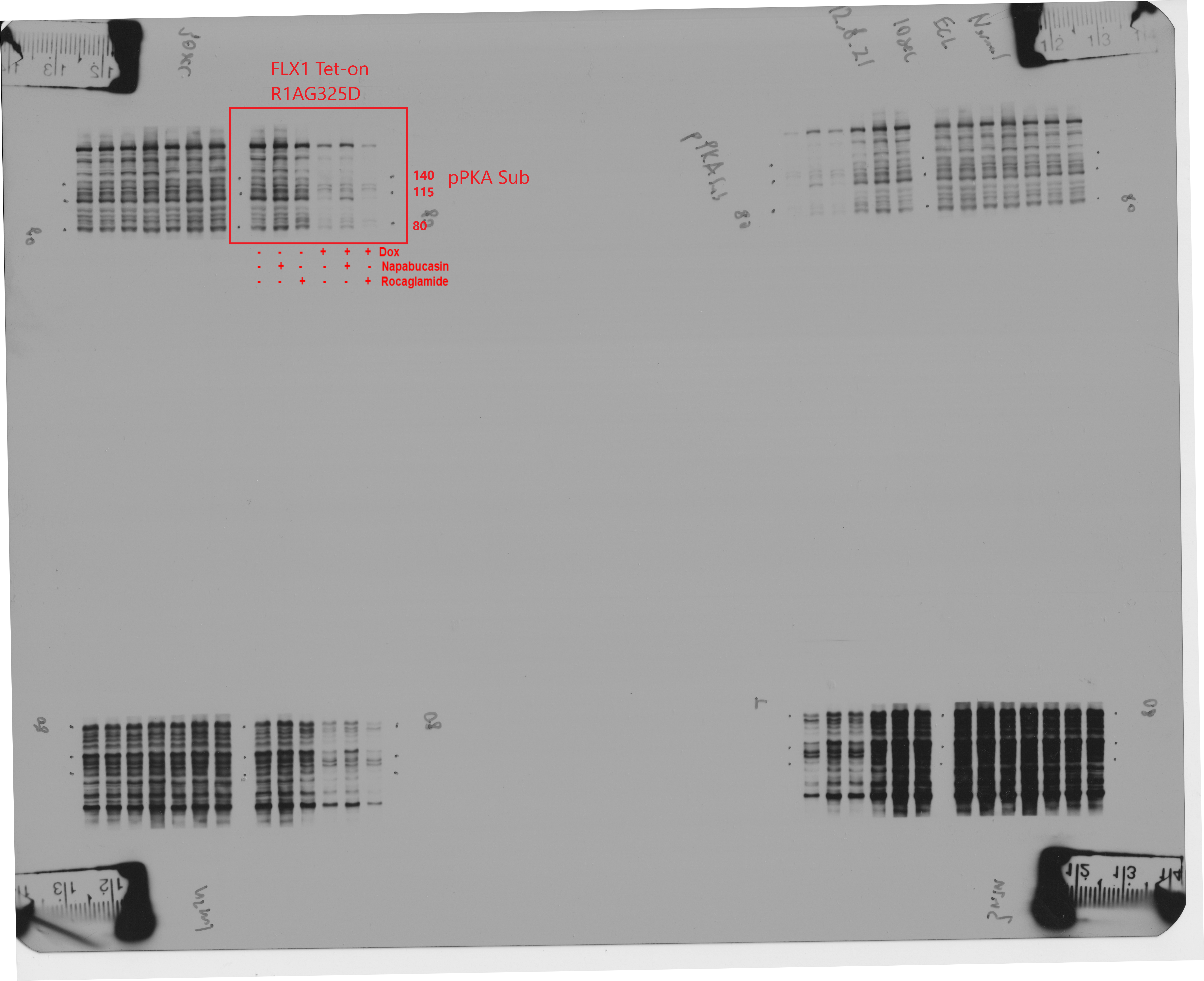

Supplement: Figure 7—source data 2. [file elife-69521-fig7-data2.zip › 7B/Figure 7B FLX1 R1A pPKAsub Labelled.tiff]

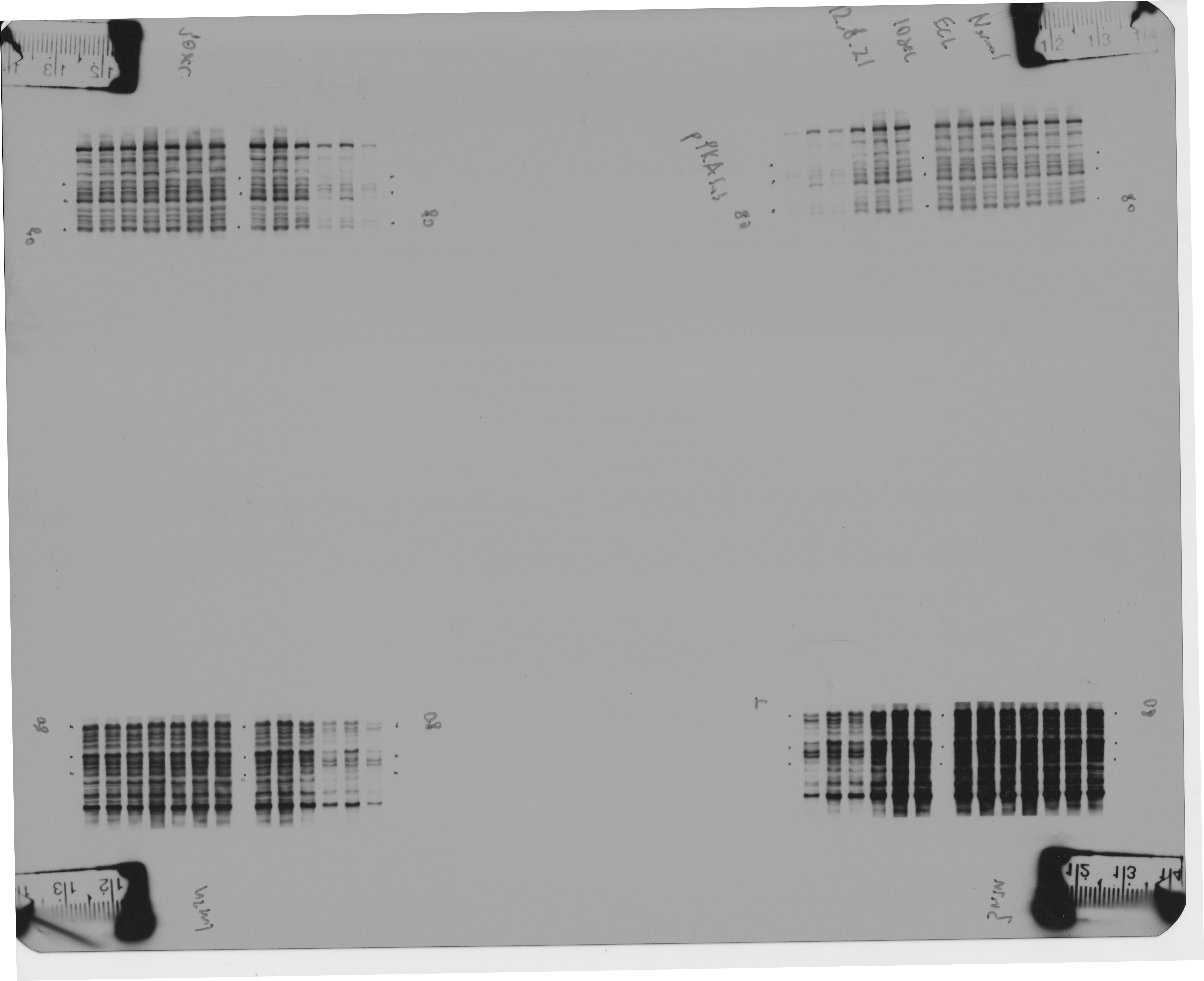

Supplement: Figure 7—source data 2. [file elife-69521-fig7-data2.zip › 7B/Figure 7B FLX1 R1A pPKAsub Raw.tiff]

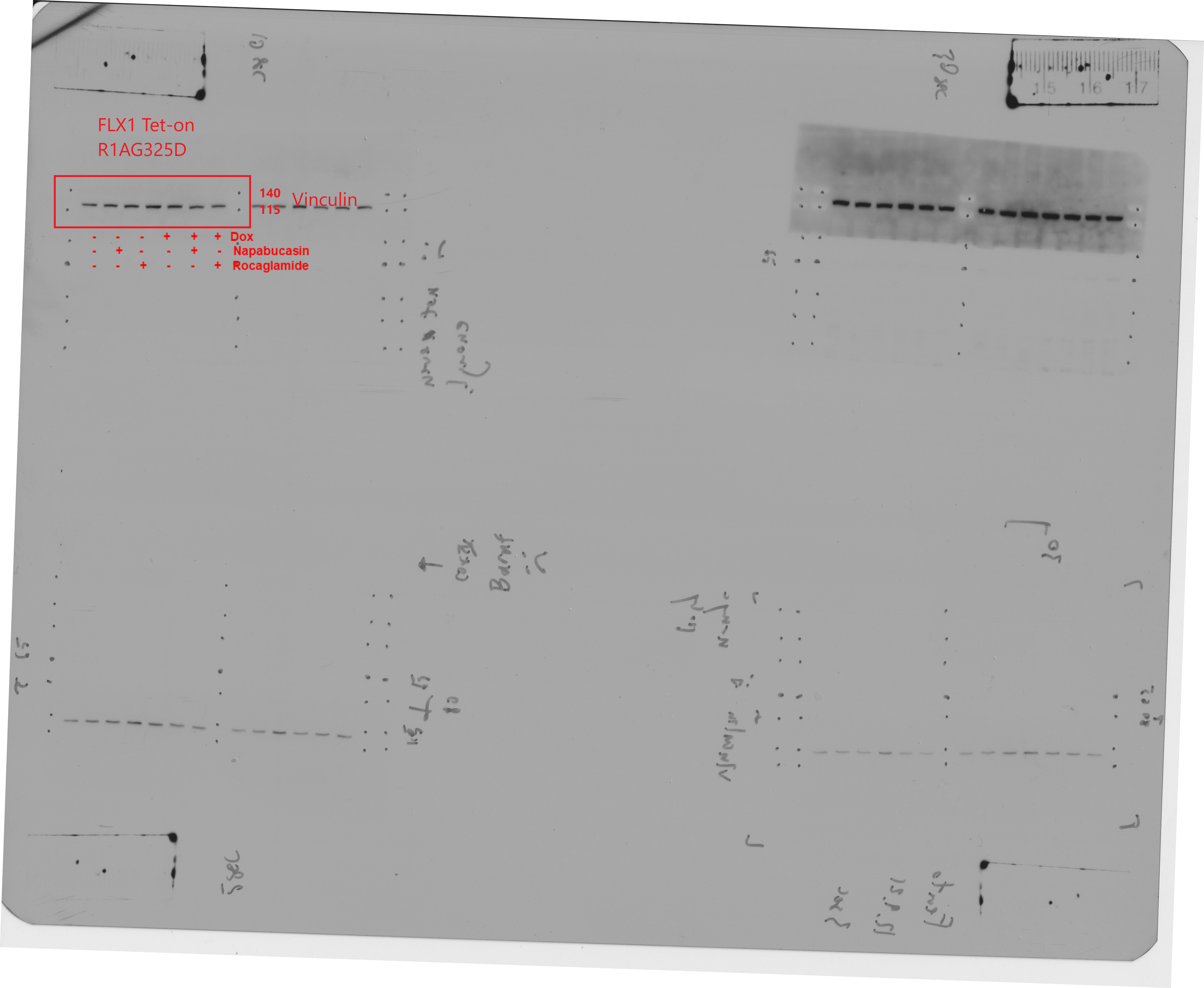

Supplement: Figure 7—source data 2. [file elife-69521-fig7-data2.zip › 7B/Figure 7B FLX1 R1A Vinculin Labelled.tiff]

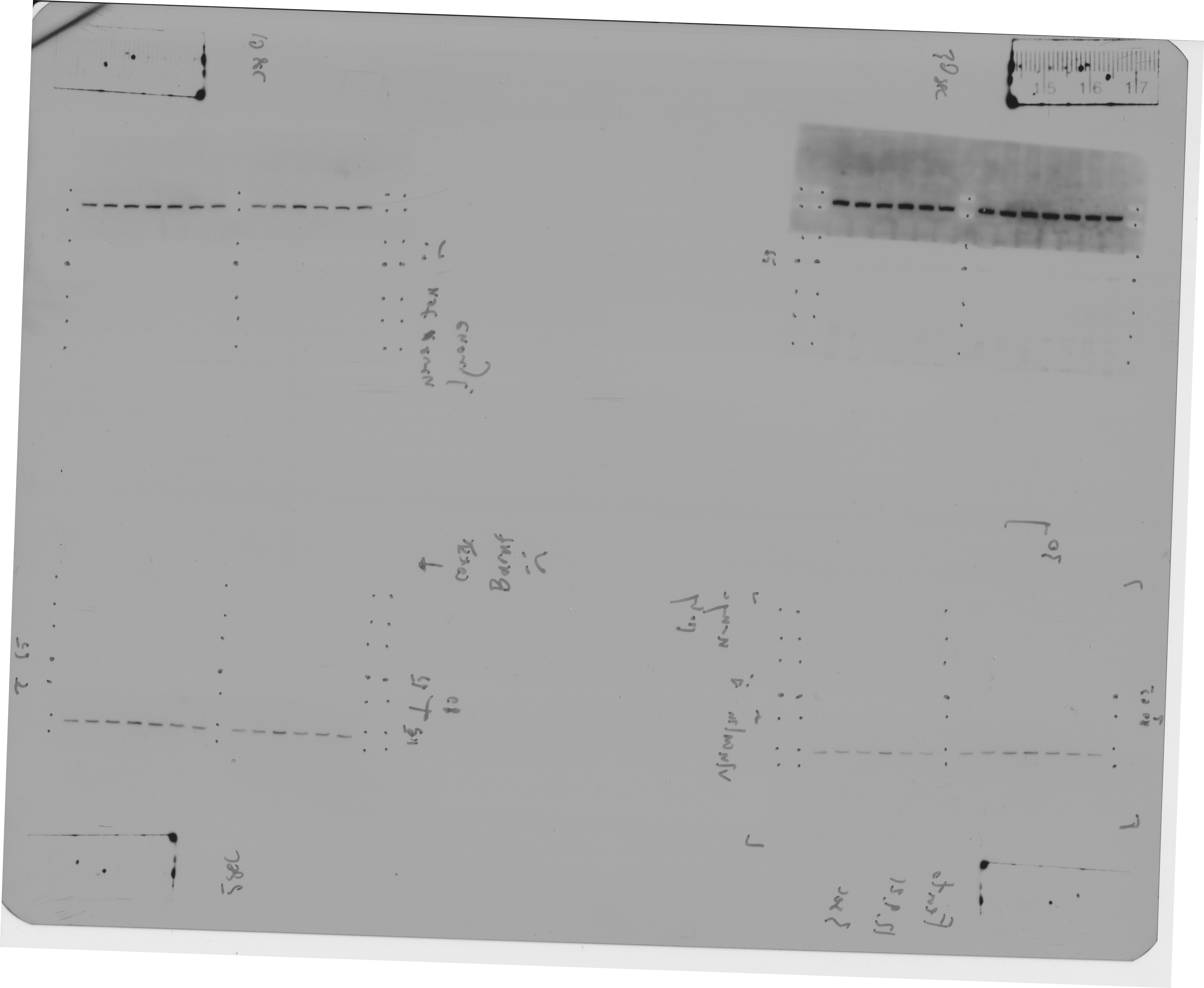

Supplement: Figure 7—source data 2. [file elife-69521-fig7-data2.zip › 7B/Figure 7B FLX1 R1A Vinculin Raw.tiff]

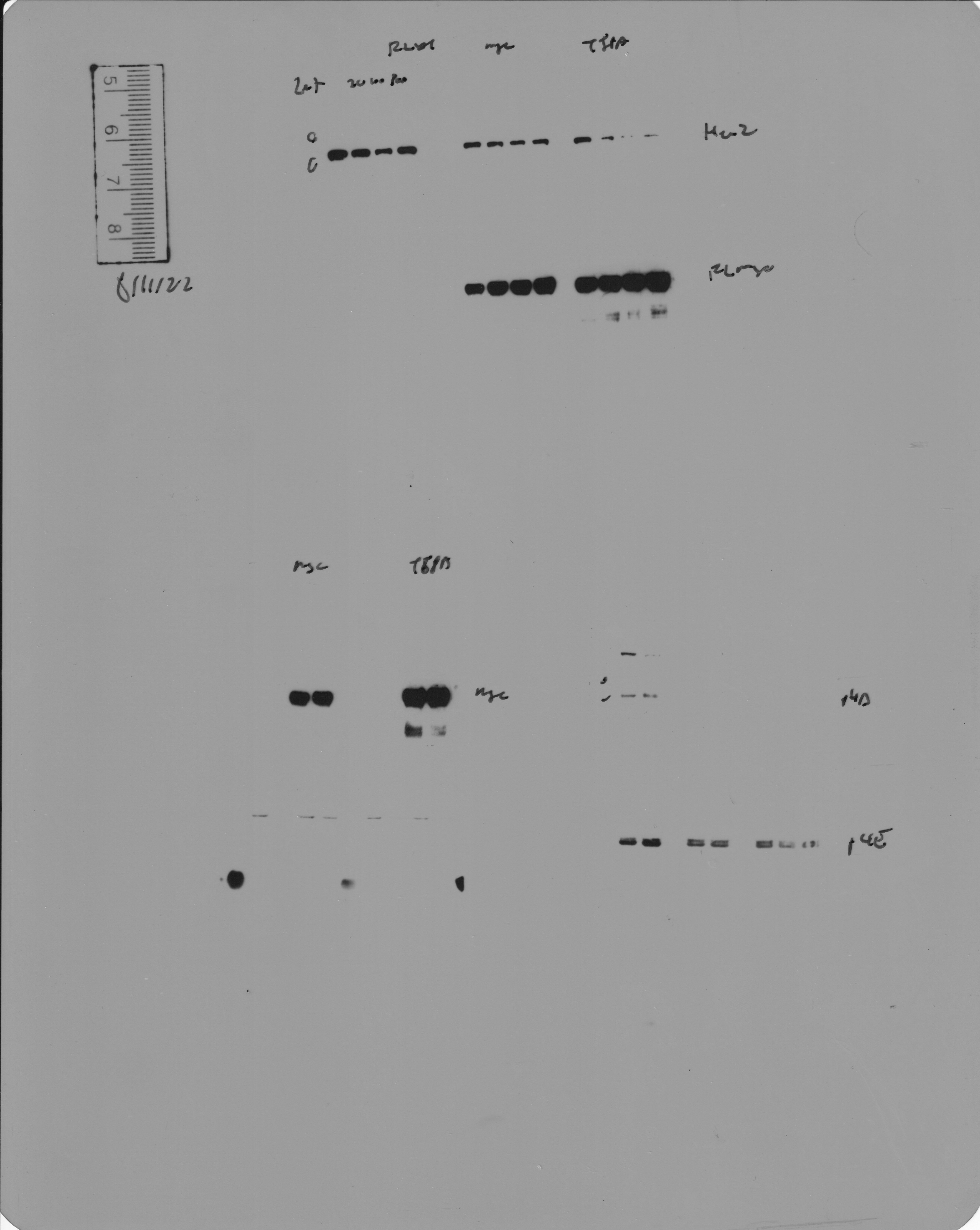

Supplement: Figure 7—source data 3. [file elife-69521-fig7-data3.zip › 7C/7C MYC overexpressed raw.tif]

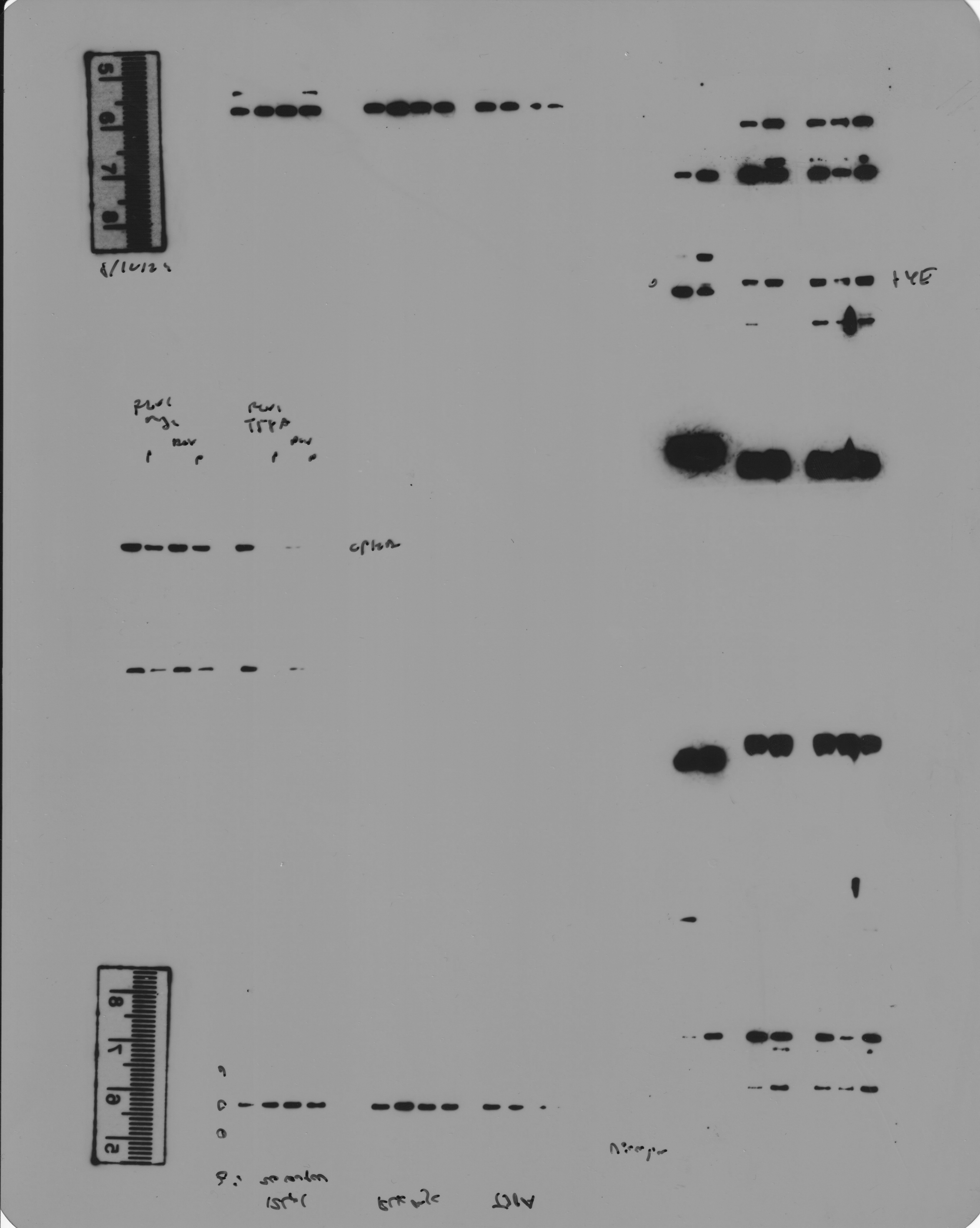

Supplement: Figure 7—source data 3. [file elife-69521-fig7-data3.zip › 7C/7E vinculin raw.tif]

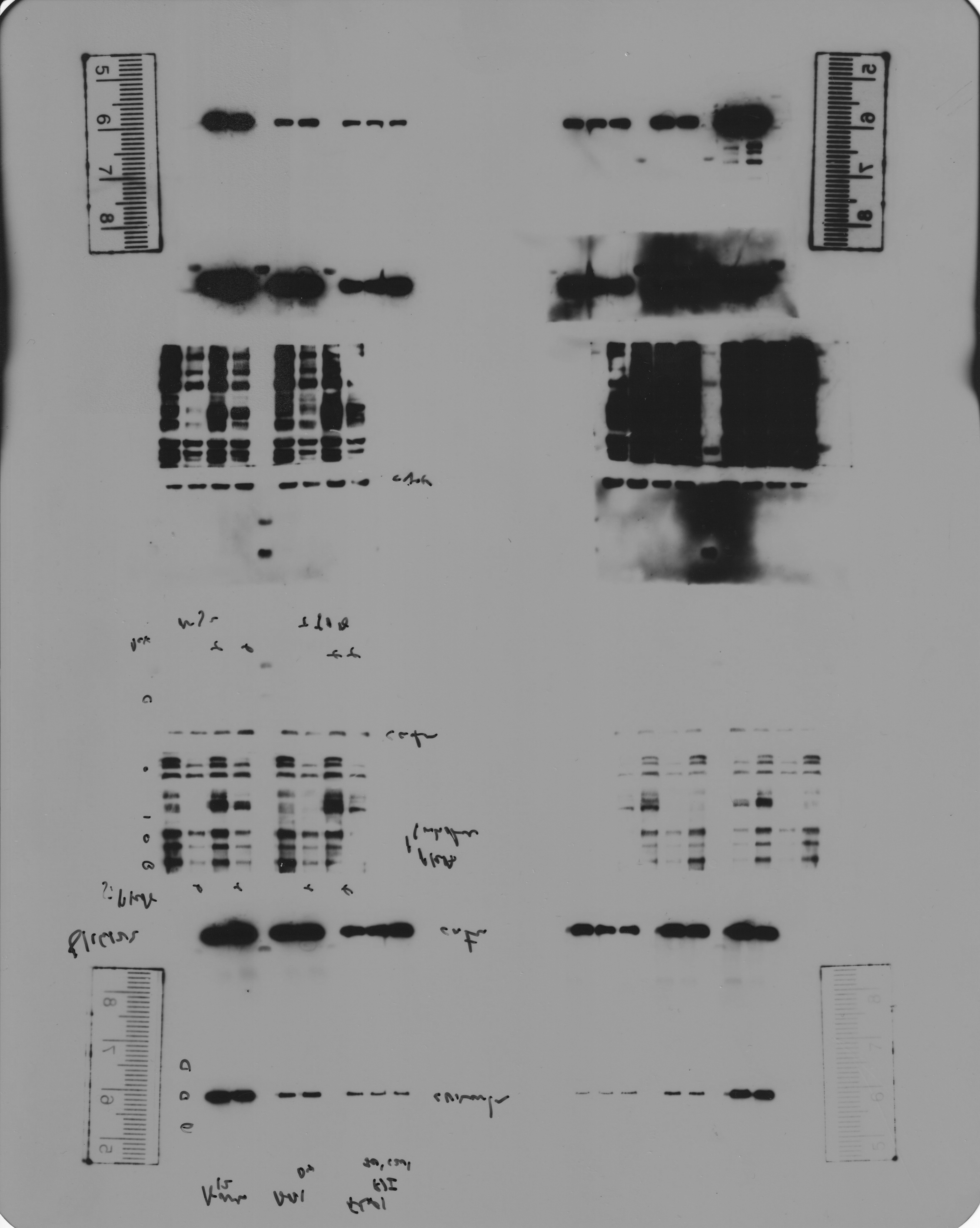

Supplement: Figure 7—figure supplement 1—source data 1. [file elife-69521-fig7-figsupp1-data1.zip › S4A/S4A actin raw.tif]

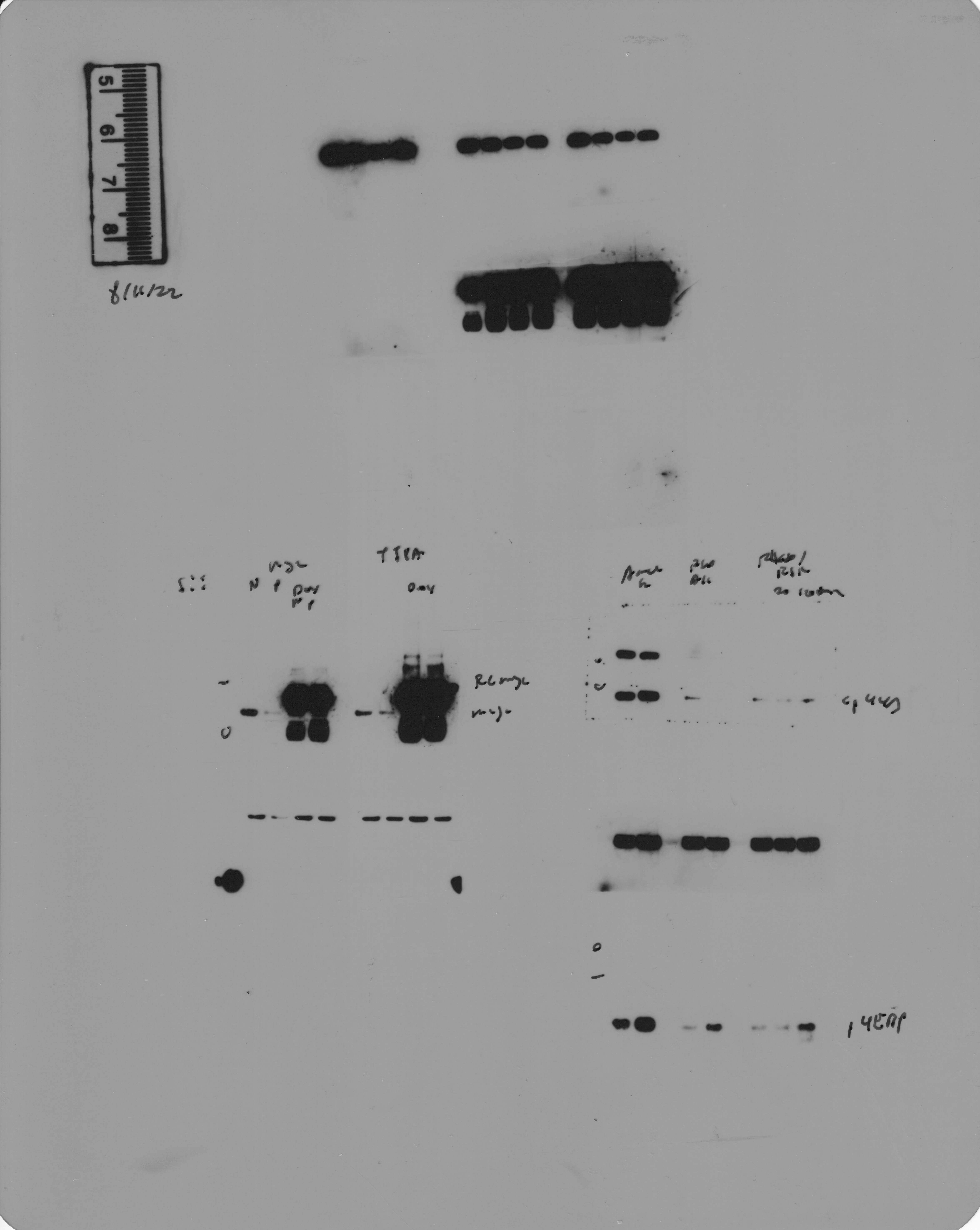

Supplement: Figure 7—figure supplement 1—source data 1. [file elife-69521-fig7-figsupp1-data1.zip › S4A/S4A MYC raw.tif]

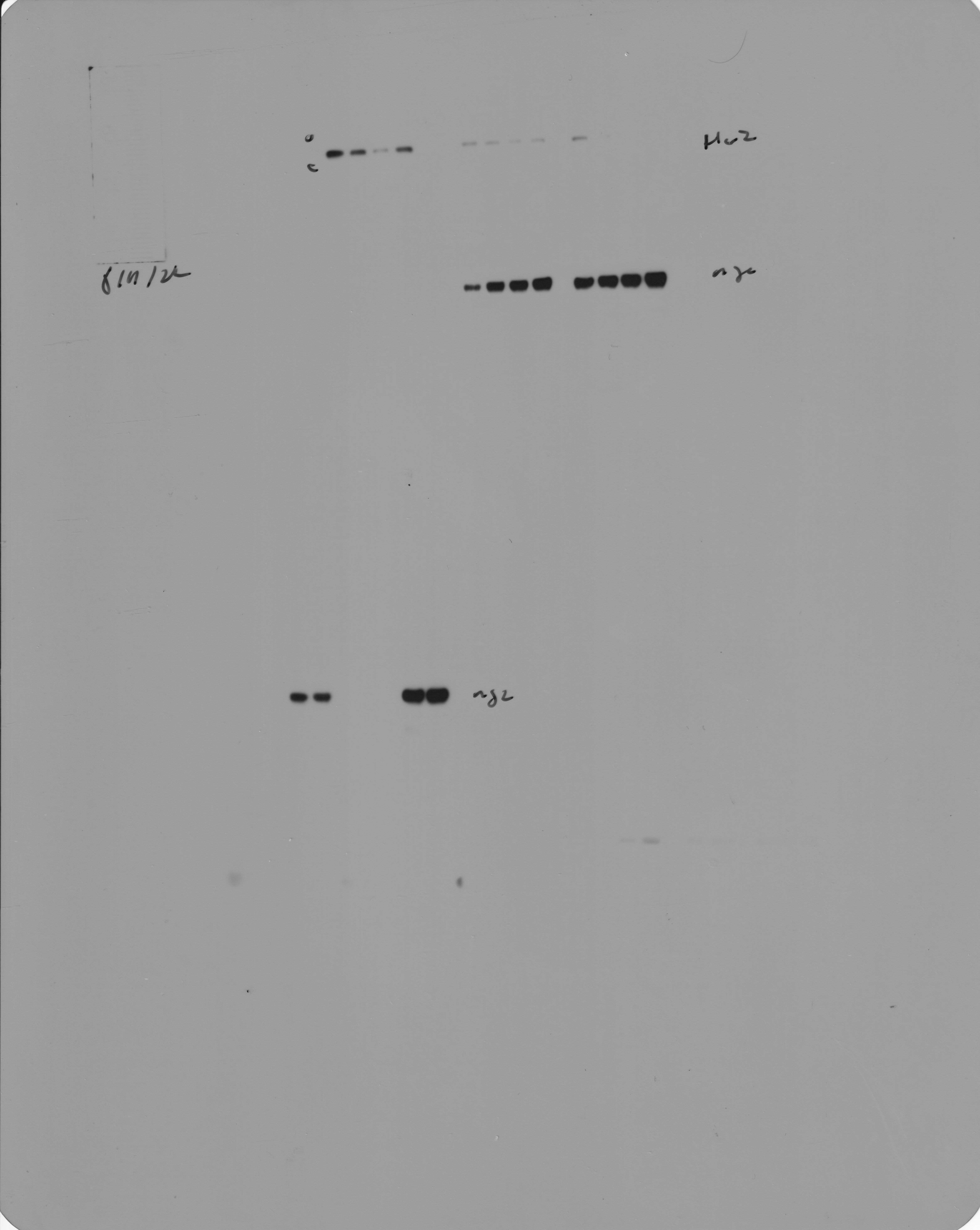

Supplement: Figure 7—figure supplement 1—source data 1. [file elife-69521-fig7-figsupp1-data1.zip › S4A/S4A MYC short.tif]

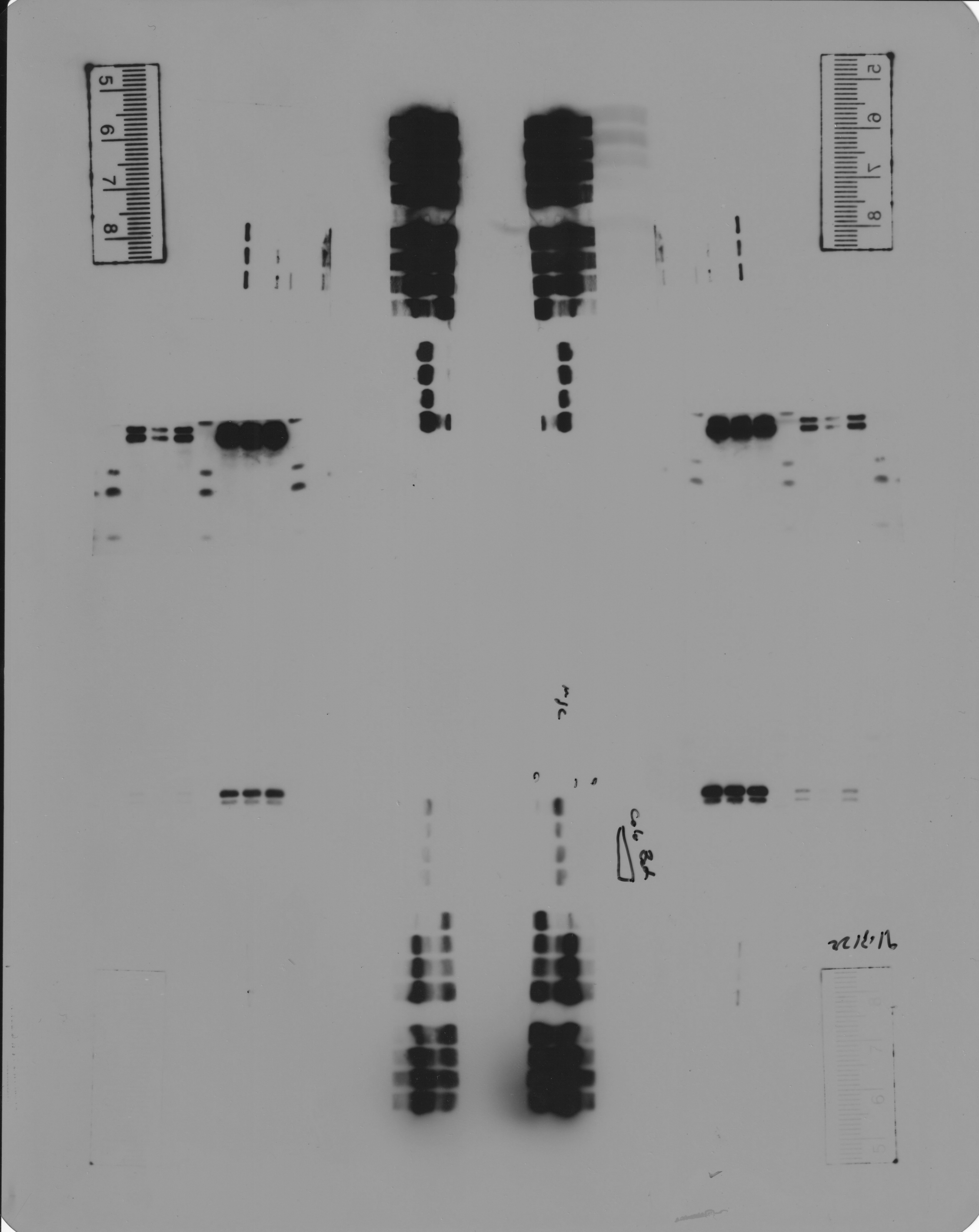

Supplement: Figure 7—figure supplement 1—source data 2. [file elife-69521-fig7-figsupp1-data2.zip › S4B/S4B endogenous MYC raw.tif]

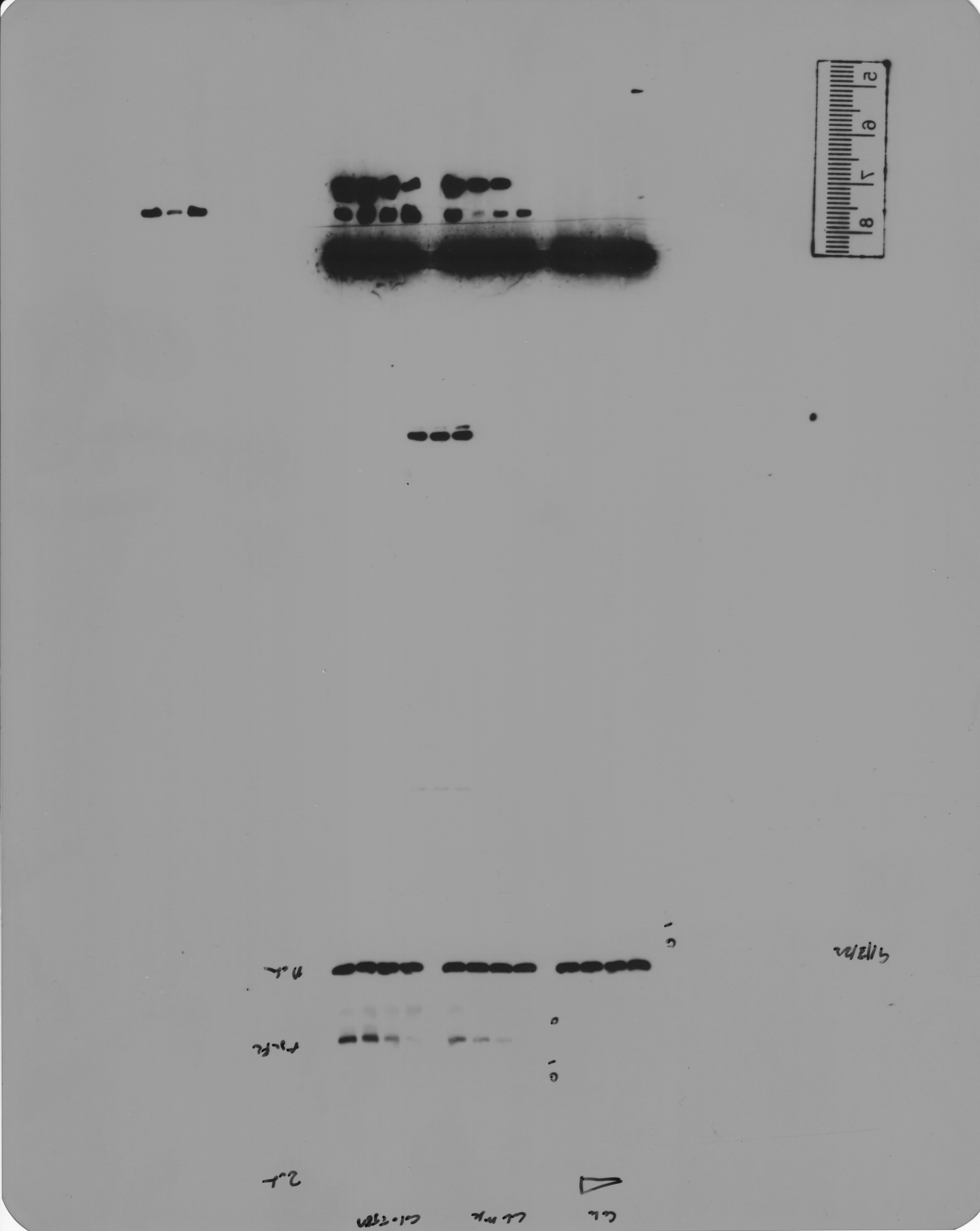

Supplement: Figure 7—figure supplement 1—source data 2. [file elife-69521-fig7-figsupp1-data2.zip › S4B/S4B oe MYC actin raw.tif]
