# Supplementary material for: Oncogenic PKA signaling increases c-MYC protein expression through multiple targetable mechanisms
Source: eLife. 2023 Jan 24;12:e69521. doi: 10.7554/eLife.69521 (PMC9925115; doi:10.7554/eLife.69521)
Supplement: Figure 7—source data 3. [file elife-69521-fig7-data3.zip › 7C/7C markup.pdf]

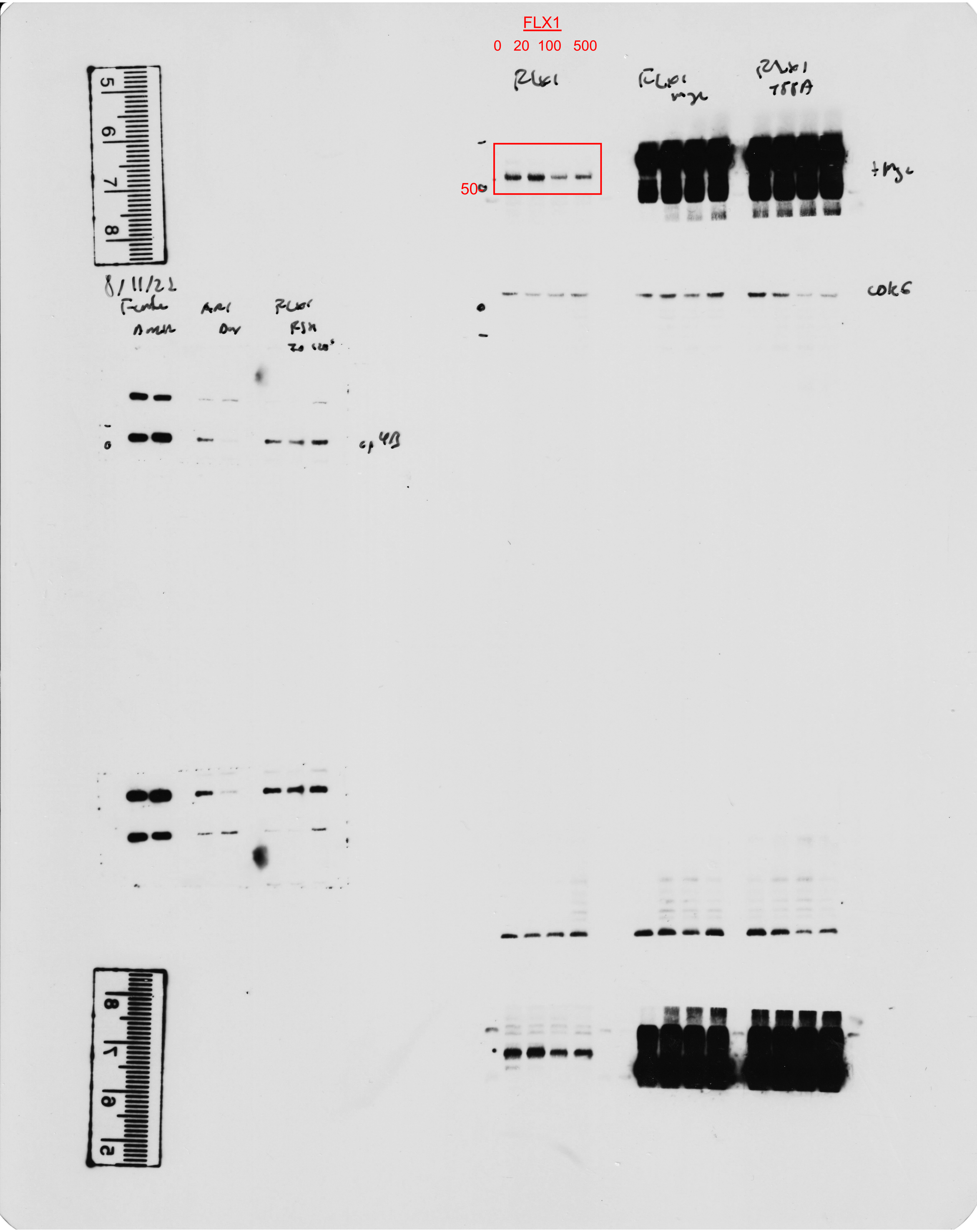
FLX1
0 20 100 500
50
8/11/21
T88A

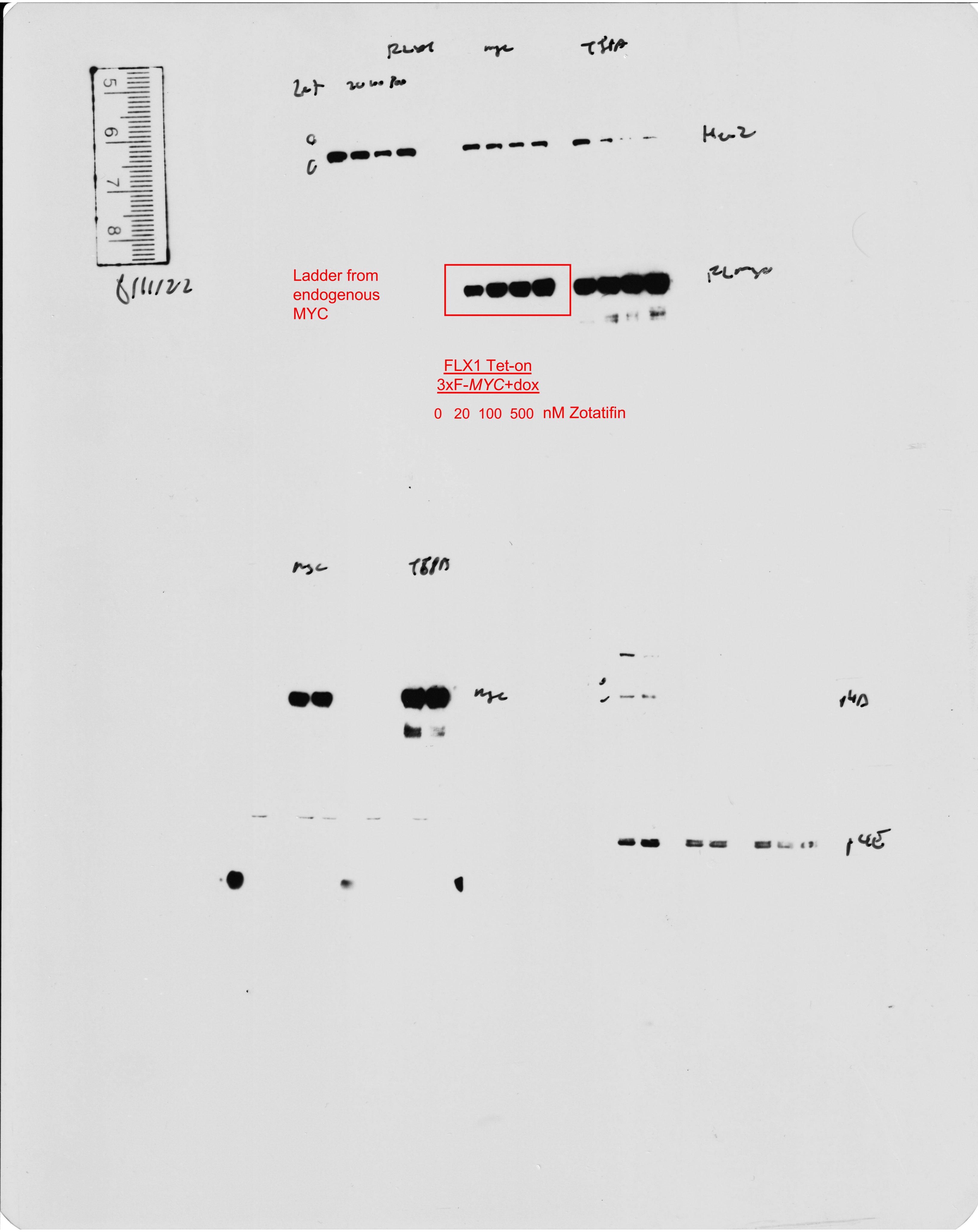
Ladder from endogenous MYC
FLX1 Tet-on 3xF-MYC+dox
0 20 100 500 nM Zotatifin

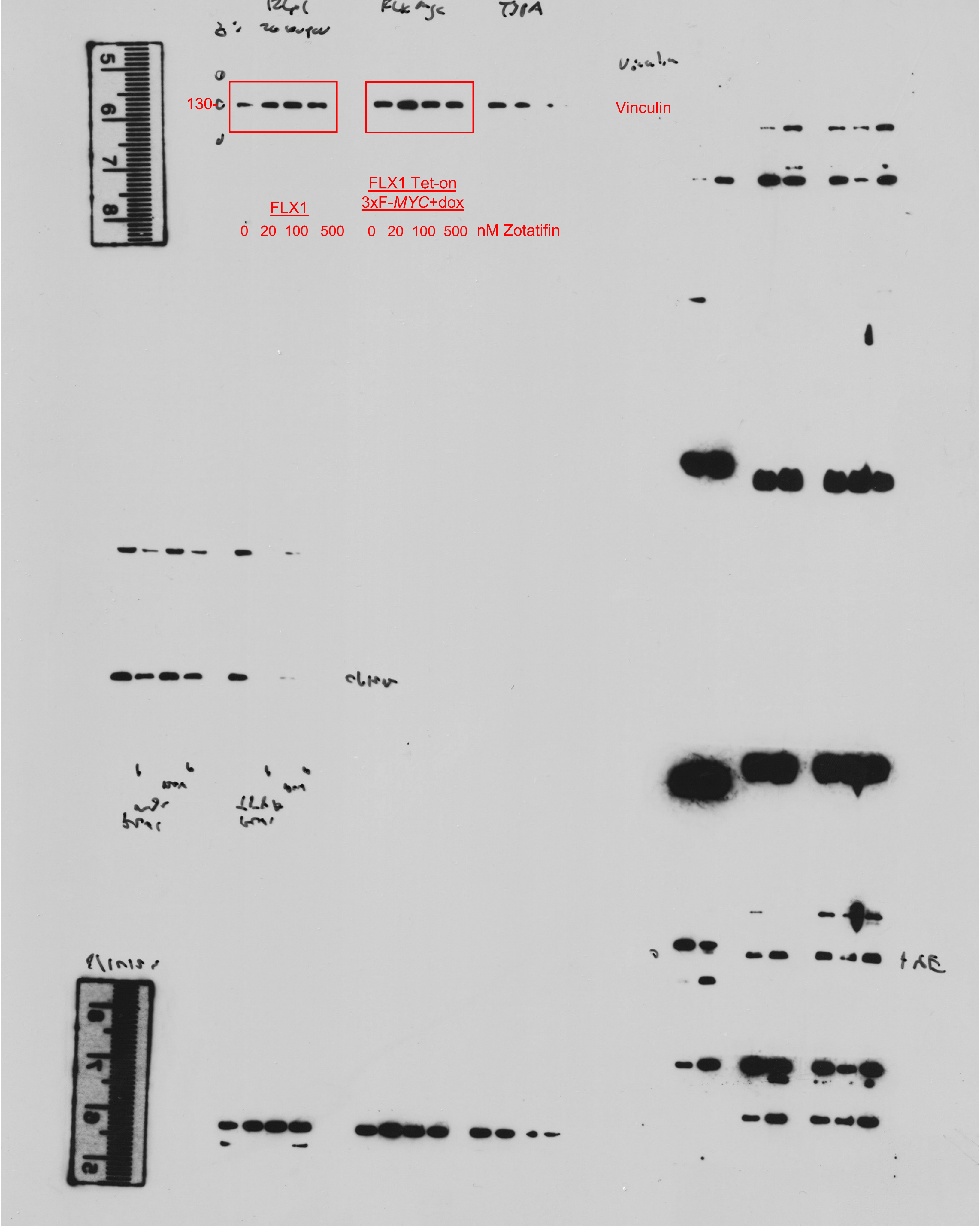
130
Vinculin
FLX1
FLX1 Tet-on
3xF-MYC+dox
0 20 100 500
0 20 100 500 nM Zotatifin
