# Supplementary material for: Oncogenic PKA signaling increases c-MYC protein expression through multiple targetable mechanisms
Source: eLife. 2023 Jan 24;12:e69521. doi: 10.7554/eLife.69521 (PMC9925115; doi:10.7554/eLife.69521)
Supplement: Figure 7—figure supplement 1—source data 1. [file elife-69521-fig7-figsupp1-data1.zip › S4A/S4a markup.pdf]

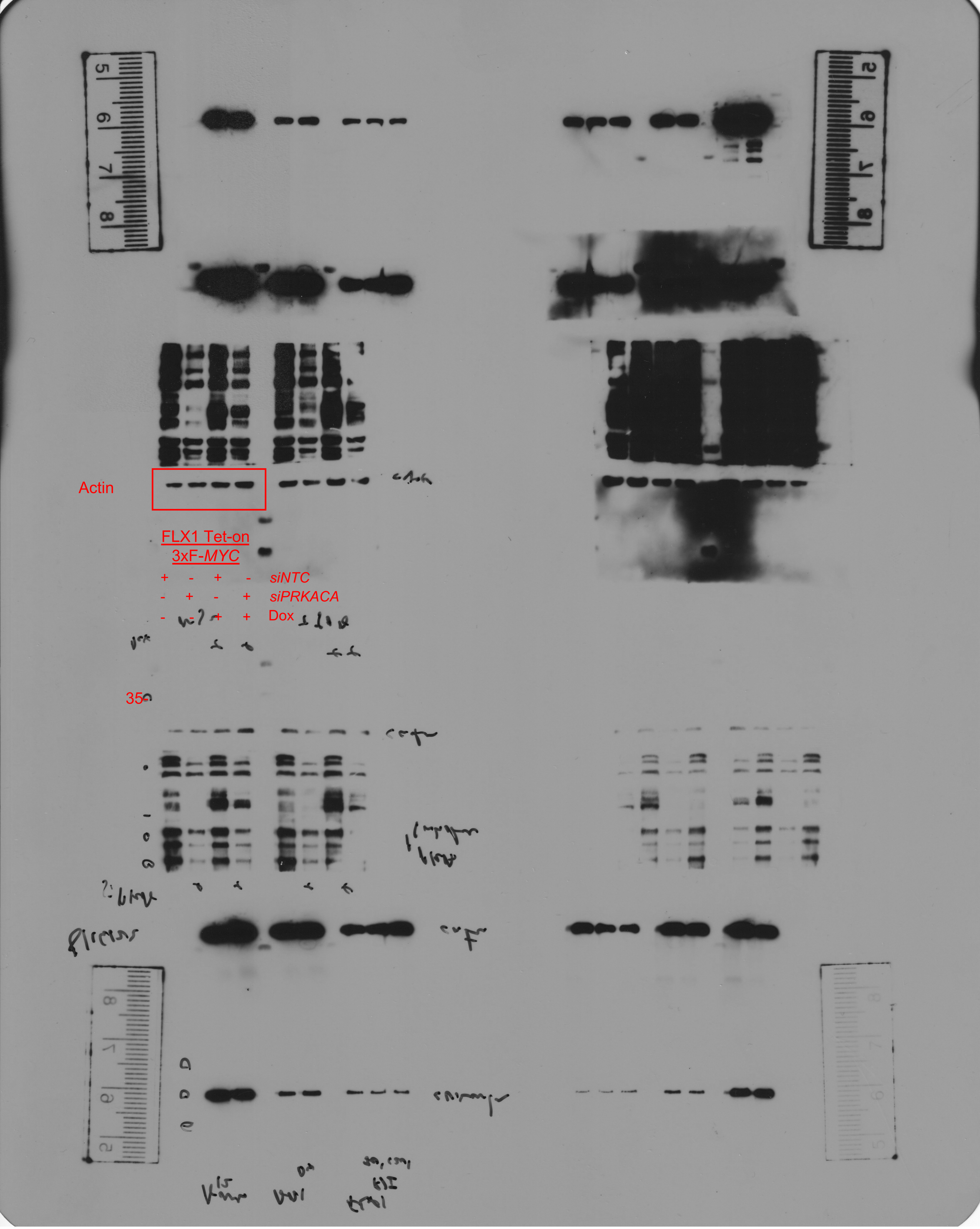
Actin
FLX1 Tet-on
3xF-MYC
+ - + - siNTC
- + - + siPRKACA
- - + + Dox
35

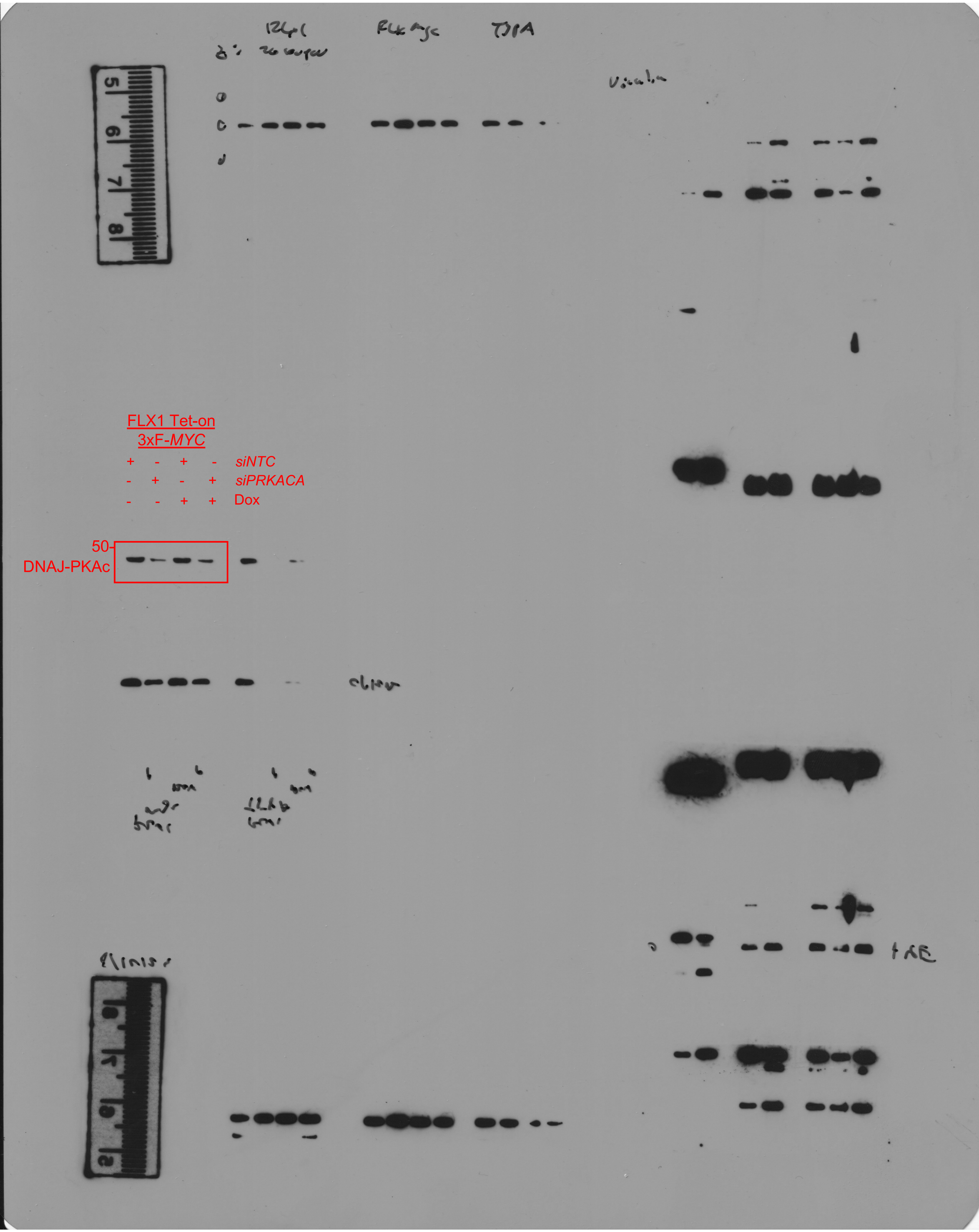
FLX1 Tet-on
3xF-MYC
+ - + - siNTC
- + - + siPRKACA
- - + + Dox
50-
DNAJ-PKAc

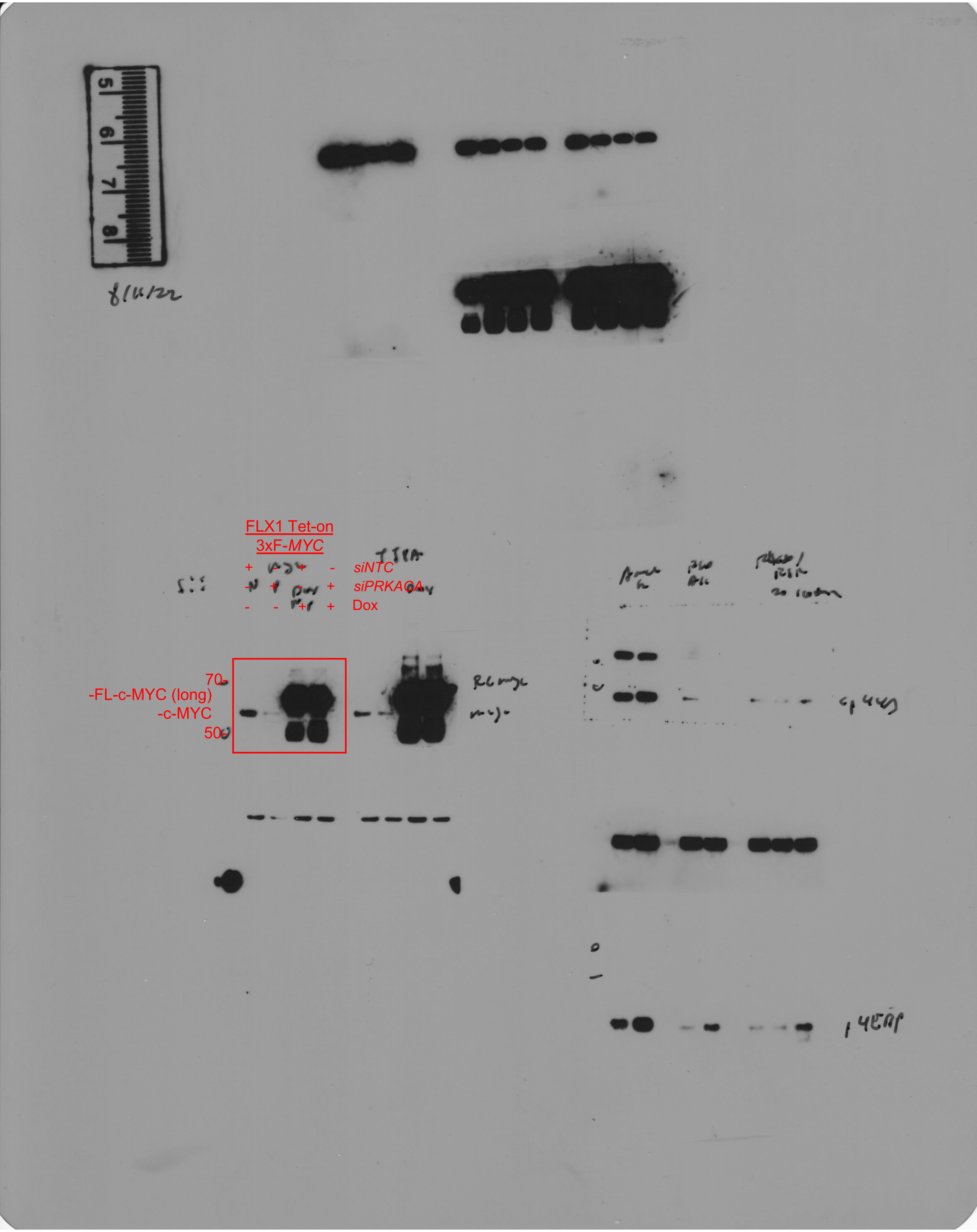

8/11/22
FLX1 Tet-on
3xF-MYC
+ + - siNTC
- - + siPRKACA
- - + + Dox
70-
-FL-c-MYC (long)
-c-MYC
50-

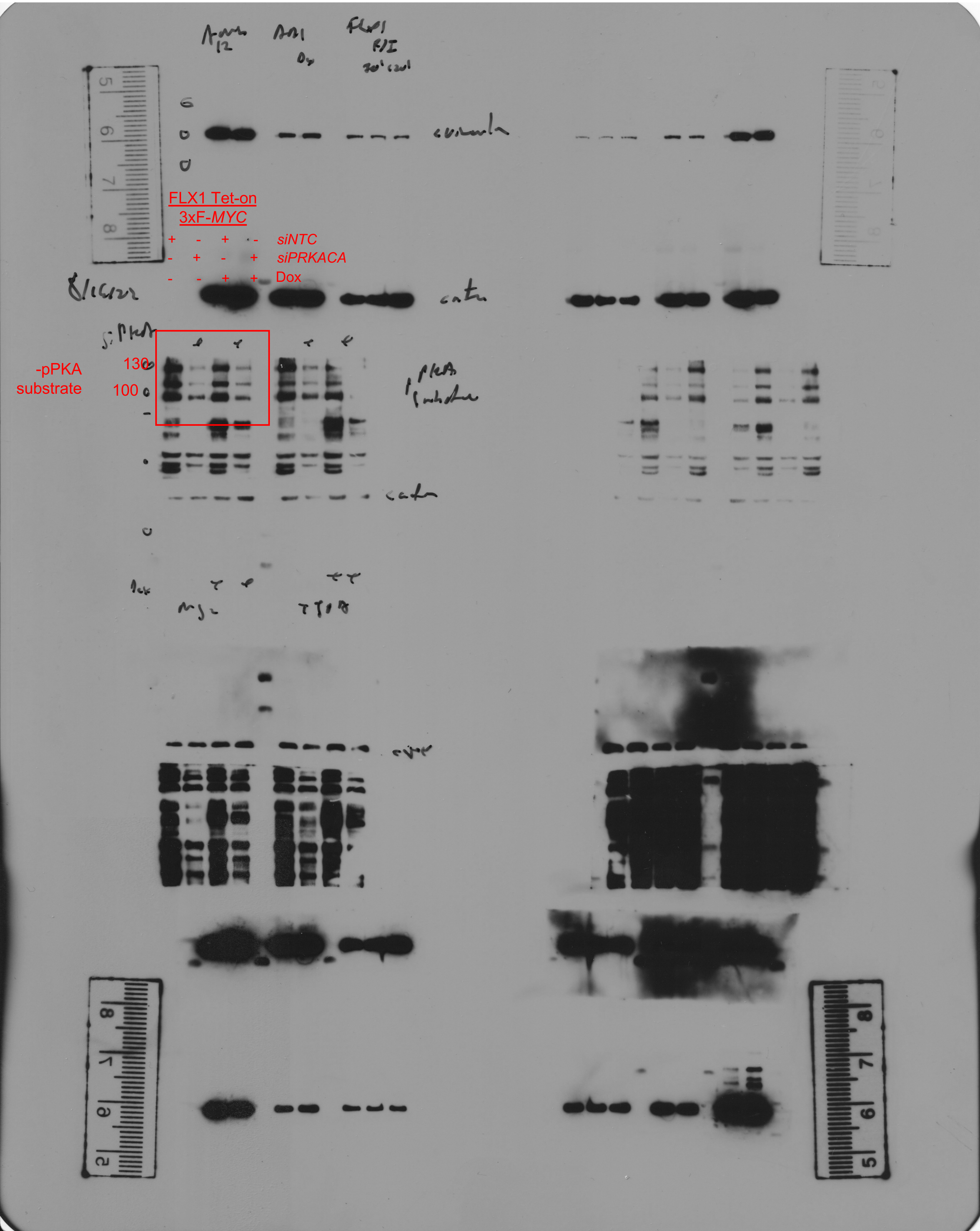
FLX1 Tet-on
3xF-MYC
+ - + - siNTC
- + - + siPRKACA
- - + + Dox
-pPKA
substrate
130
100

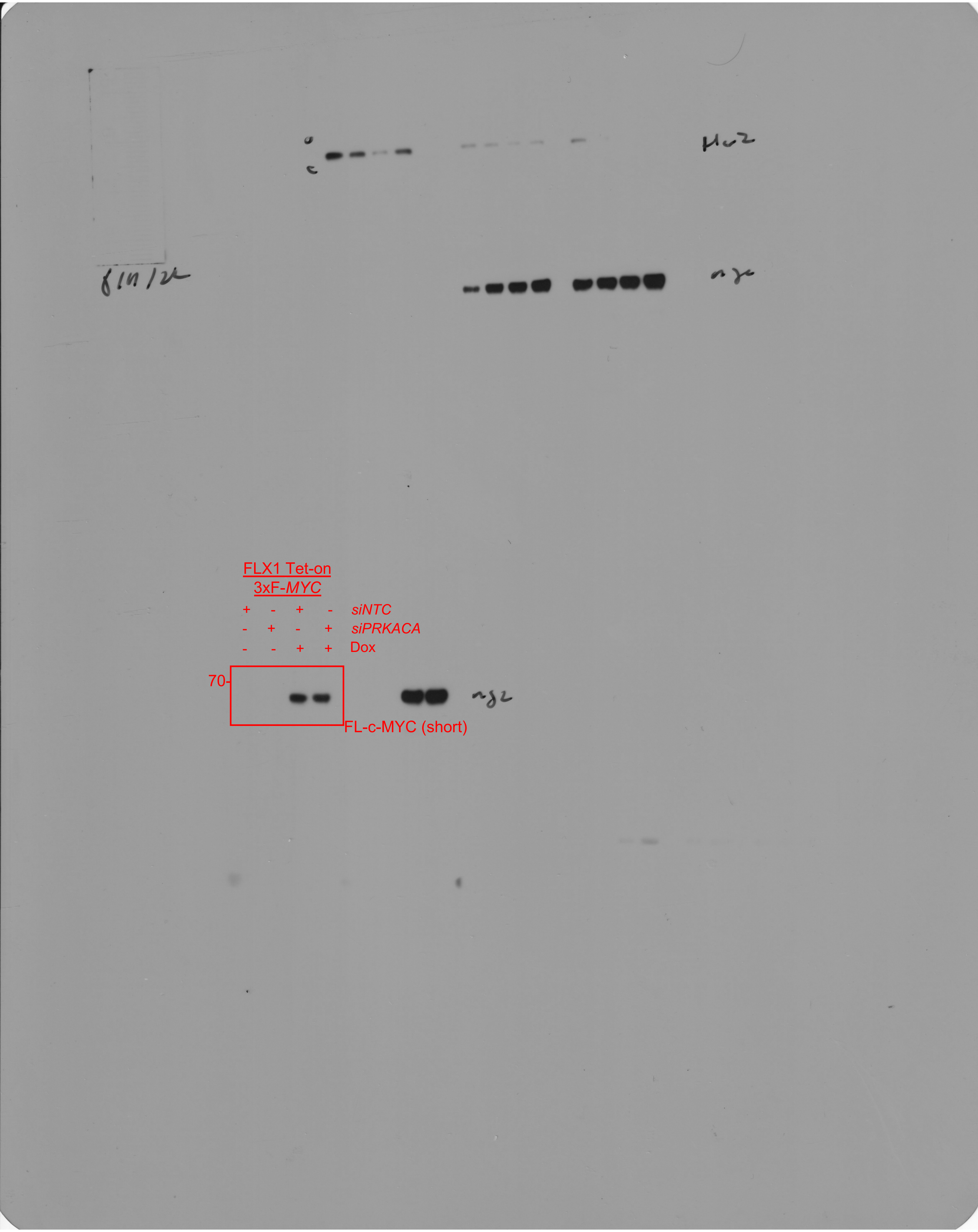
FLX1 Tet-on
3xF-MYC
+ - + - siNTC
- + - + siPRKACA
- - + + Dox
70-
FL-c-MYC (short)
