# Supplementary material for: Oncogenic PKA signaling increases c-MYC protein expression through multiple targetable mechanisms
Source: eLife. 2023 Jan 24;12:e69521. doi: 10.7554/eLife.69521 (PMC9925115; doi:10.7554/eLife.69521)
Supplement: Figure 7—figure supplement 1—source data 2. [file elife-69521-fig7-figsupp1-data2.zip › S4B/S4b markup.pdf]

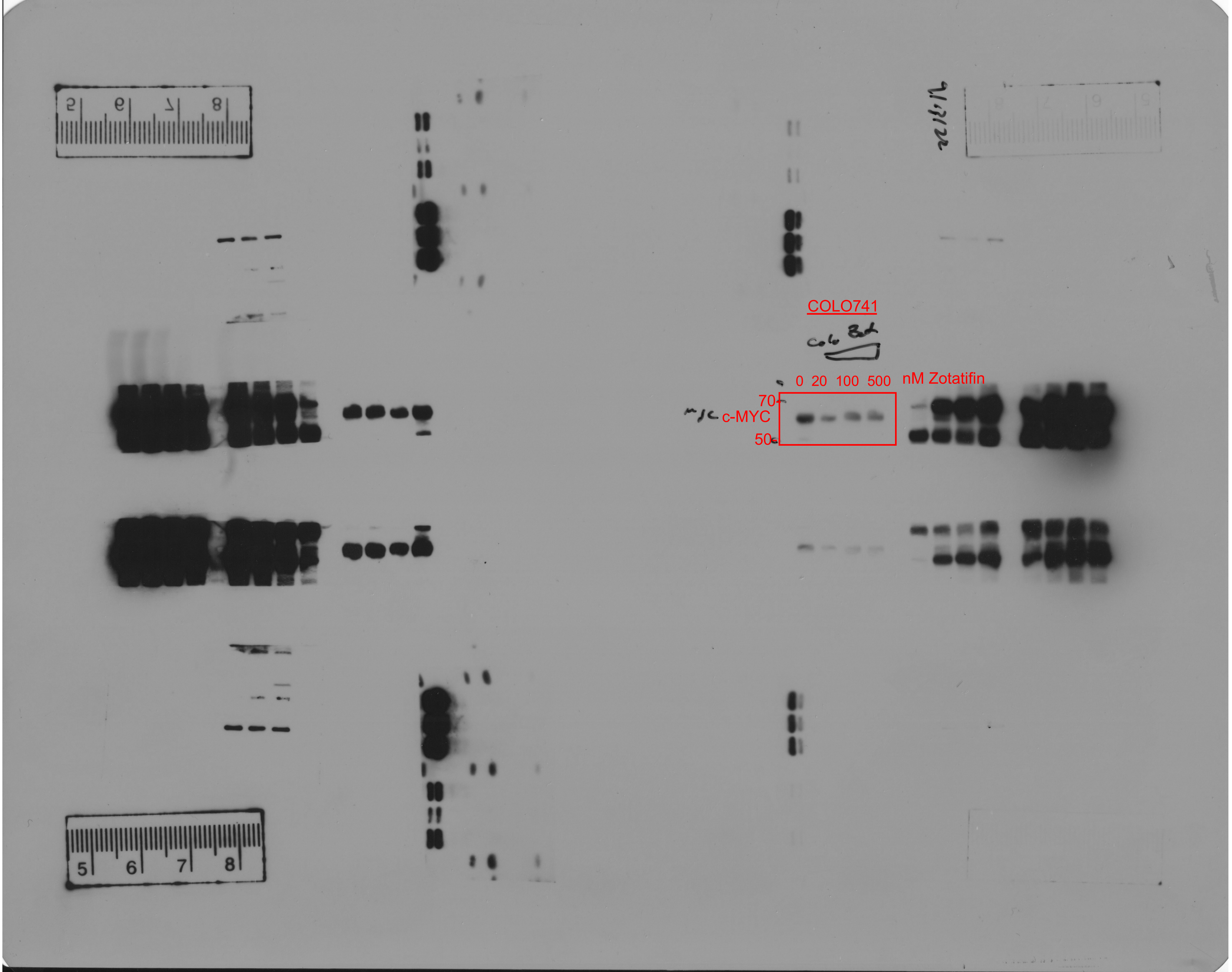
COLO741
0 20 100 500 nM Zotatifin
70
c-MYC
50
5 6 7 8

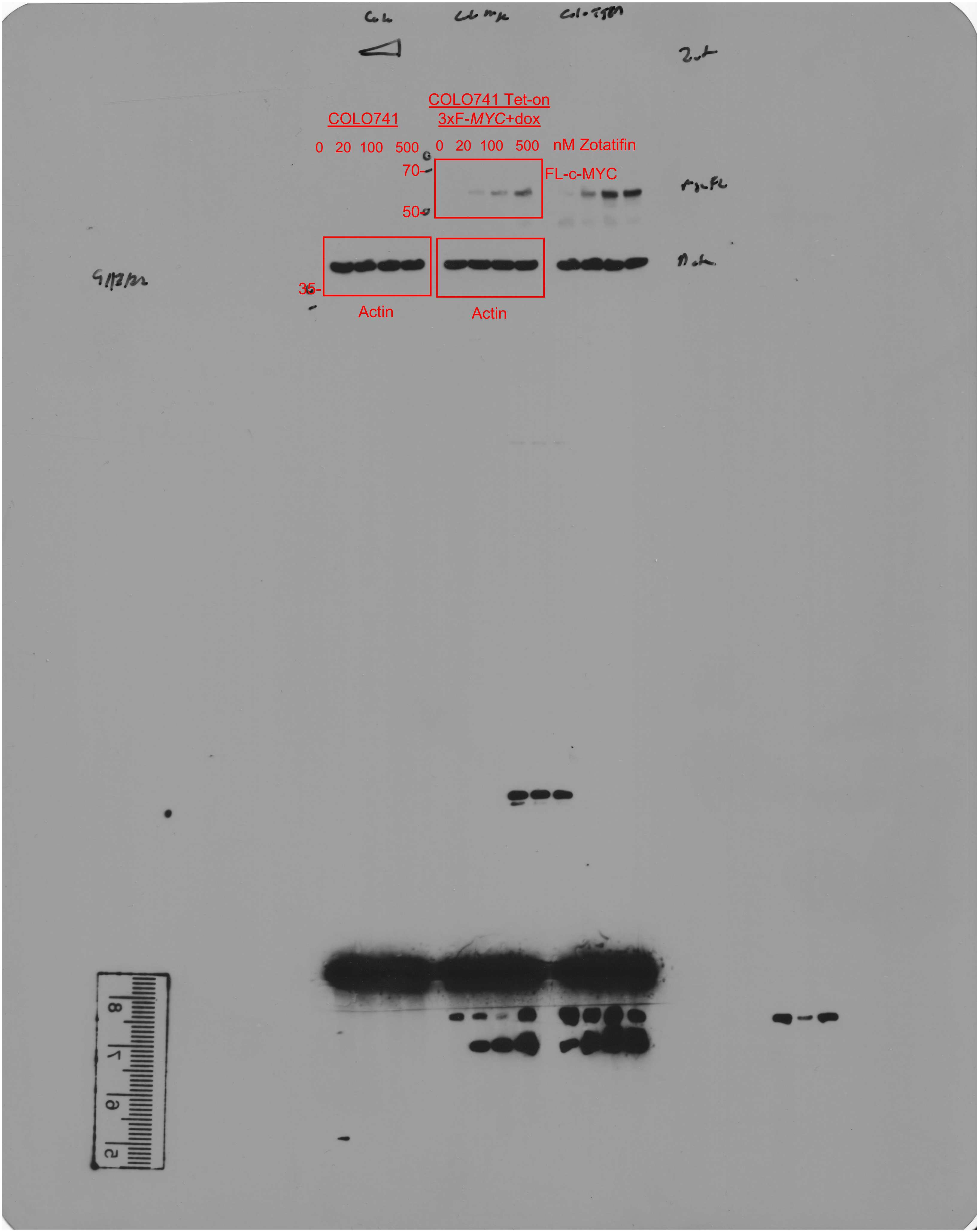
COLO741
COLO741 Tet-on
3xF-*MYC*+dox
0 20 100 500
0 20 100 500 nM Zotatifin
70-
50-
FL-c-MYC
35-
Actin
Actin
